# Stable Associations Masked by Temporal Variability in the Marine Copepod Microbiome

**DOI:** 10.1371/journal.pone.0138967

**Published:** 2015-09-22

**Authors:** Pia H. Moisander, Andrew D. Sexton, Meaghan C. Daley

**Affiliations:** 1 Department of Biology, University of Massachusetts Dartmouth, North Dartmouth, Massachusetts, United States of America; 2 Department of Biological Sciences, University of New Hampshire, Durham, New Hampshire, United States of America; Stazione Zoologica Anton Dohrn, Naples, ITALY

## Abstract

Copepod-bacteria interactions include permanent and transient epi- and endobiotic associations that may play roles in copepod health, transfer of elements in the food web, and biogeochemical cycling. Microbiomes of three temperate copepod species (*Acartia longiremis*, *Centropages hamatus*, and *Calanus finmarchicus*) from the Gulf of Maine were investigated during the early summer season using high throughput amplicon sequencing. The most prominent stable component of the microbiome included several taxa within Gammaproteobacteria, with *Pseudoalteromonas* spp. especially abundant across copepod species. These Gammaproteobacteria appear to be promoted by the copepod association, likely benefitting from nutrient enriched microenvironments on copepods, and forming a more important part of the copepod-associated community than *Vibrio* spp. during the cold-water season in this temperate system. Taxon-specific associations included an elevated relative abundance of Piscirickettsiaceae and Colwelliaceae on *Calanus*, and *Marinomonas* sp. in *Centropages*. The communities in full and voided gut copepods had distinct characteristics, thus the presence of a food-associated microbiome was evident, including higher abundance of Rhodobacteraceae and chloroplast sequences in the transient communities. The observed variability was partially explained by collection date that may be linked to factors such as variable time since molting, gender differences, and changes in food availability and type over the study period. While some taxon-specific and stable associations were identified, temporal changes in environmental conditions, including food type, appear to be key in controlling the composition of bacterial communities associated with copepods in this temperate coastal system during the early summer.

## Introduction

Microbial associations have been reported with numerous marine animals, including invertebrates and crustaceans [1, 2], but there is a limited understanding on the stability and function of such associations at the microbial community level. Stable symbioses with microbes form in many terrestrial insects and ticks [3], where the symbiotic bacteria may aid the host in digestion, uptake of nutrients, reproduction, immune response, and other defenses [4]. In terrestrial insect arthropods and some marine animal symbioses, the symbionts are often passed to the next generation vertically (maternally), and the host and symbiont commonly undergo co-evolution [5–7], as evidence of the importance of the permanent co-existence. Stable chemolithoautotrophic symbioses are common in deep-sea invertebrates [2, 8], but little is known about potential symbioses in copepods, marine pelagic crustaceans (Arthropoda). In addition to stable symbioses, insects and invertebrates hosting symbionts also contain microbes that are present only temporarily either as ecto- or endobionts or simply passing through the gut [9].

Copepods are known to host microbial communities [1] that appear to attach at greatest abundances near the copepod mouth and anus, and egg sacs of females. Cultivation-based studies have suggested an abundance of Gammaproteobacteria, especially *Vibrio* spp. both in the surfaces and guts of copepods, while other groups such as *Flavobacterium*, *Cytophaga*, and *Pseudomonas* have also been reported [10–13]. To date only few cultivation-independent studies using high-throughput sequencing methods have been conducted on the marine copepod microbiome [14, 15], and little is known about temporal variability of the microbiome and factors controlling it.

Microbial communities in ecto- and endobiotic associations of copepods could potentially be linked with the surrounding environmental conditions, especially the availability and type of food [16]. Much of the microbiome could be passively recruited from the environment, thus environmental controls should play an important role in controlling the composition and function. Additionally, copepods could contain host-specific associations, or symbioses, and a natural “core microbiome” dependent on the copepod environment that is independent of food. It could be argued that a majority of the copepod-associated microorganisms are mostly from food and random recruitment from the environment, and that the relationship with the copepod is either neutral or parasitic. However, microscopic and cultivation data indicate that copepods also contain intestinal, presumably non-transient flora [17], suggesting the community in copepods is not purely under environmental control and might form an important ecological association.

The goal of this study was to address the nature and variability of the microbiome on temperate marine copepods. In the Gulf of Maine (GoM), many fish species rely on copepods as food during their larval stages, thus copepods play an important role in trophic transfer towards commercially important fish. This study focused on three common copepod genera with distinct ecological niches in the Gulf of Maine, *Acartia* spp. (primarily *A*. *longiremis*), *Centropages* sp. (primarily *C*. *hamatus*), and *Calanus finmarchicus*. With a body length of 2–3 mm, *C*. *finmarchicus* is the largest of the three, and is an important food source for fishes and whales in the northwestern Atlantic Ocean [18–20] with its range covering the North Atlantic from coastal waters to the open ocean. The smaller copepods *A*. *longiremis* and *C*. *hamatus* are primarily estuarine and coastal species.

The major goal of this study was to provide a description of the microbiome in these important North Atlantic copepods, to investigate whether there is a “core microbiome” suggesting stable associations, whether such core microbiome varies among these three copepods with different ecological niches, and whether the microbiome experiences temporal changes. The study was conducted over a 3-week period in early summer in the Gulf of Maine located in the Northwestern Atlantic Ocean.

## Materials and Methods

Samples were collected between June 4 and 23, 2011, off the Shoals Marine Laboratory on Appledore Island in the Gulf of Maine (42.9892°N, 70.6150°W) (S1 Table). Sampling was conducted under permits for sampling marine organisms in the Gulf of Maine waters (State of Maine Department of Marine Resources special license ME 2011-56-01 and New Hampshire Fish and Game Department permit No. MFD 1117). No endangered or protected species were sampled in this study. Most of the samples were collected between 9:30 am and noon. Copepods were collected by towing a 200-μm zooplankton net behind a small boat at a slow speed for approximately 10 min. The sample was diluted with surface seawater immediately upon collection and poured into clean polyethylene containers. Surface water temperature was measured using a temperature probe (YSI) and salinity was measured with a refractometer. Upon returning to the lab, the containers were kept at 4°C and copepods picked out of the plankton samples under a dissecting microscope for molecular and microscopic analyses. Copepods were identified to genus level, rinsed with 0.2 μm filtered seawater 2–3 times, then 1–10 live copepods were placed in a tube with a mixture of 0.1—and 0.5—μm diameter sterile glass beads (S1 Table) (these samples were termed ‘full gut copepods’). To study the influence of gut contents on the overall microbiome, some copepods underwent a starvation period for removal of gut contents. Copepods were picked to filtered seawater in petri dishes that were kept at 4°C for 24–48 h, and the live copepods were then picked and preserved into tubes as above (these samples were termed ‘starved copepods’). The relatively low holding temperature minimized secondary bacterial growth during incubation, and was sufficient in keeping the copepods alive. Hereafter these ‘starved’ copepods are referred to with ‘S’ and copepods with ‘full gut’, preserved shortly after sampling, are referred to with ‘F’ (e.g. *Acartia* S and *Acartia* F, respectively). Parallel water samples were collected from the same general area where the zooplankton tows were conducted, by filling a polycarbonate bottle below the surface. The seawater was filtered through a 0.2 μm Supor filter (Pall Gelman, Port Washington, NY) folded into a bead beater tube with sterile glass beads (as above). Copepods and filtered seawater samples (S1 Table) were immediately placed in liquid nitrogen, and kept there until moved to -80°C for long-term storage. Very few *Calanus* individuals were obtained in the samples, and only a set of ‘Full gut’ individuals was included in the study.

DNA was extracted using the Qiagen Blood and Tissue kit with modifications (S1 Text). Polymerase chain reaction was used to amplify the mitochondrial cytochrome oxidase gene (COI) to confirm the identity of copepods, using primers LCO1490F and HCO2198R [21] (S1 Text, S2 Table). The GenBank accession numbers for the COI sequences from this study are KT186356-KT186363.

For characterization of the microbial community, 16S rRNA gene was amplified using a paired end amplicon sequencing procedure for Illumina MiSeq (S1 Text) employing the V3-V4 region of the 16S rRNA gene [22]. The multiplexed sample was sequenced at the Tufts University Core Facility using Illumina MiSeq V3 600 cycles paired end method. The samples for this study were included in two separate sequencing runs, each in a set of a total of 96 samples multiplexed (pooled with samples from other studies). PCR with negative controls with nuclease free water added in place of DNA template were run in parallel and sequenced, although bands were not visible when the amplified products from these controls were run on the gel electrophoresis.

The paired end sequences were first combined and initial downstream analyses conducted in QIIME [23, 24]. Operational taxonomic unit (OTU) in this study was defined as group of sequences that had a 97% or higher identity. OTUs that formed >1% of the sequences in the negatives (no template controls) were removed from sample data, assuming any sequences representing these OTUs in the samples originated from reagent contamination [25, 26] (S1 Text). QIIME was used in the initial analyses for OTU binning and the R software package ‘vegan’ was used to conduct the rarefaction analysis and make a heatmap with a dendrogram (hierarchical clustering based on Bray-Curtis distance). Redundancy analysis (RDA) on the Hellinger transformed data was also run in R, followed by ANOVA with 999 permutations. Multidimensional Scaling (MDS) analysis was conducted, with the Bray-Curtis dissimilarities of the communities tested using ANOSIM in Primer v6 [27]. LefSe analysis was used to investigate which community members were significantly more abundant in each sample type [28]. Illumina sequences from this project have been deposited to the NCBI Sequence Read Archive with accession number SRP059778.

## Results

Water temperature increased steadily from 12.1 to 16°C during the study period while salinity varied between 30–31, except for June 10^th^ when the salinity was 34. Copepods were identified based on visual observations as *Acartia* spp., *Centropages* spp., and *Calanus finmarchicus*, and further species level identification was conducted based on the COI sequence. COI data from copepod samples identified microscopically as *Centropages* spp. suggested their closest match to be *C*. *hamatus* (EU016220.1; 99–100% identity). The closest match for sequences obtained from the samples identified as *Acartia* spp. was *A*. *longiremis* (KC287256.1; 99–100% identity). The 16S rRNA gene sequence yield varied from 6040 to 132576 (62523±31897; ave±stdev) sequences per sample before removal of negatives (S1 Table). The negative controls resulted in a low number of sequences with low diversity (see Methods and S1 Text).

**Fig 1:**
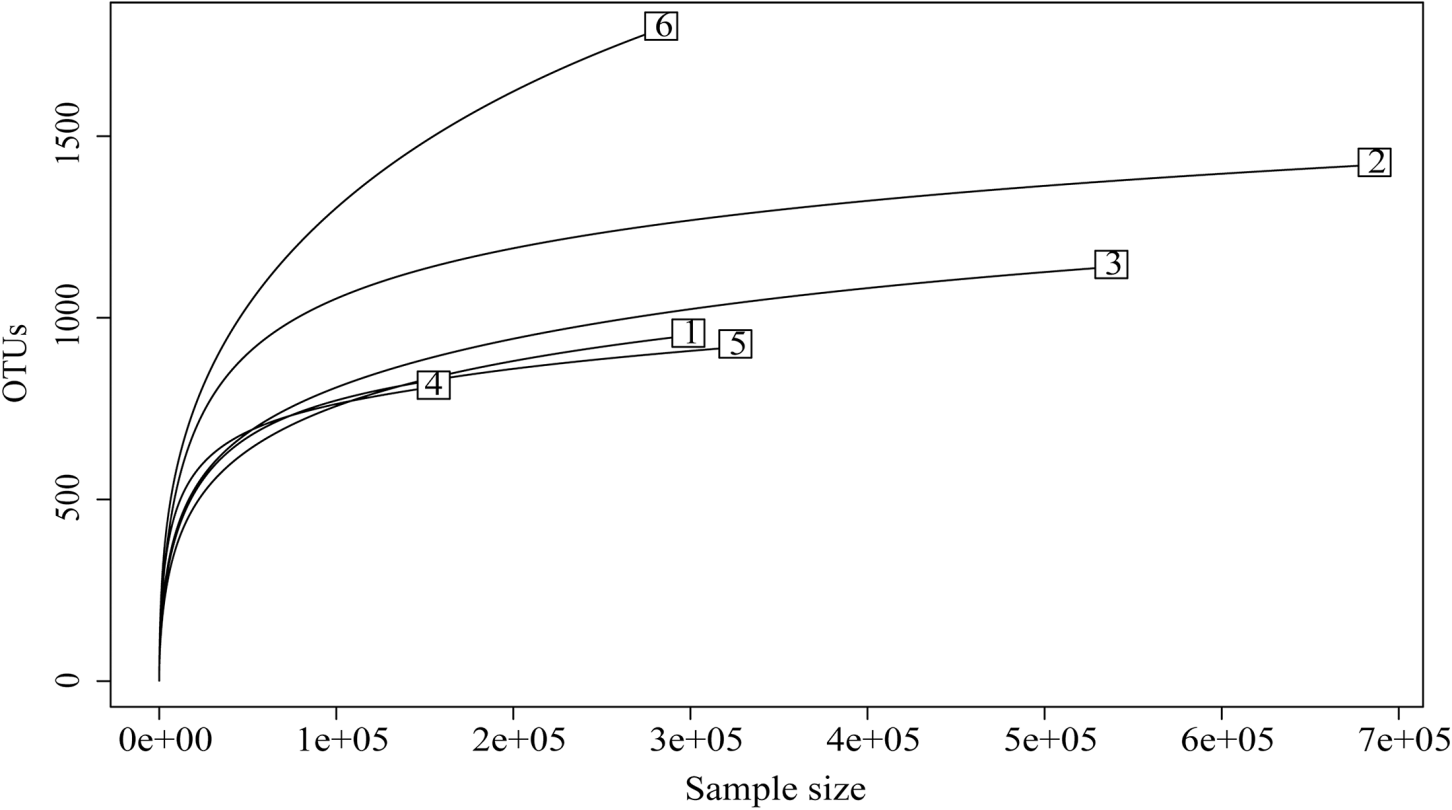
Rarefaction curves with pooled samples for the different sample types. OTU, Operational Taxonomic Unit (97% similarity threshold). S, starved; F, full gut; 1, *Acartia* S; 2, *Acartia* F; 3, *Centropages* S; 4, *Centropages* F, 5, *Calanus* F, 6, Seawater.

The projected total richness was greatest in the seawater samples, with the number of OTUs continuing to increase until 3x10^5^ sequences obtained from seawater (Fig 1). The second highest projected phylotype richness was present in *Acartia* F, with approximately 1300 OTUs at the full size of the sequence set. *Centropages* S had a slightly higher projected richness than *Centropages* F and *Calanus* F, and starved *Acartia* S, all of which had closely similar rarefaction curves. While they still had a slightly increasing OTU richness at the full size of each library, the curves from copepods had a more gradual increase than the sequences from seawater, suggesting lower overall richness.

### Community composition

**Fig 2:**
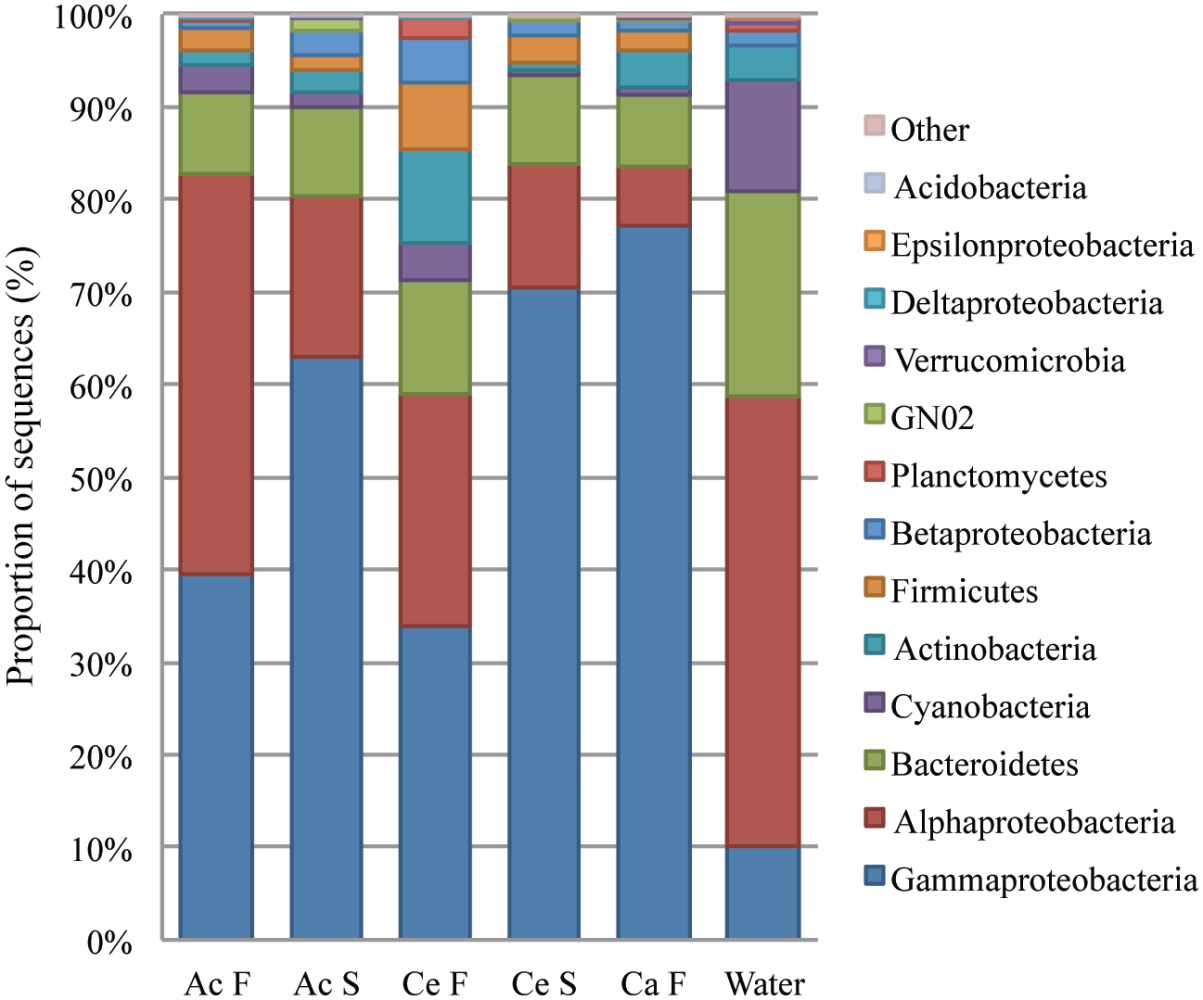
Average distribution of the community composition in samples discriminated at the phylum/sub-phylum level. F, full gut; S, starved; Ac, *Acartia*; Ce, *Centropages*; Ca, *Calanus*. *Acartia* F (n = 9), *Acartia* S (n = 6), *Calanus* F (n = 5), *Centropages* F (n = 4), *Centropages* S (n = 8), water (n = 5).

The proportion of Gammaproteobacteria was higher in copepods than in the water samples (57±23% vs. 10±3.2% of sequences respectively) (Fig 2). In *Acartia* F, the proportion of Gammaproteobacteria was close to equal to that of Alphaproteobacteria, but in all other copepod types, Gammaproteobacteria formed the largest part of the community. Proportion of Gammaproteobacteria was generally greater in starved *Acartia* and *Centropages* (63±9.7 and 70±8.4, respectively) than in their full gut counterparts (40±24% in *Acartia* F and 34±21% in *Centropages* F) (Fig 2). In *Calanus* F, the proportion of Gammaproteobacteria was similar to or higher (64–87% of sequences) than that in *Acartia* S and *Centropages* S.

**Fig 3:**
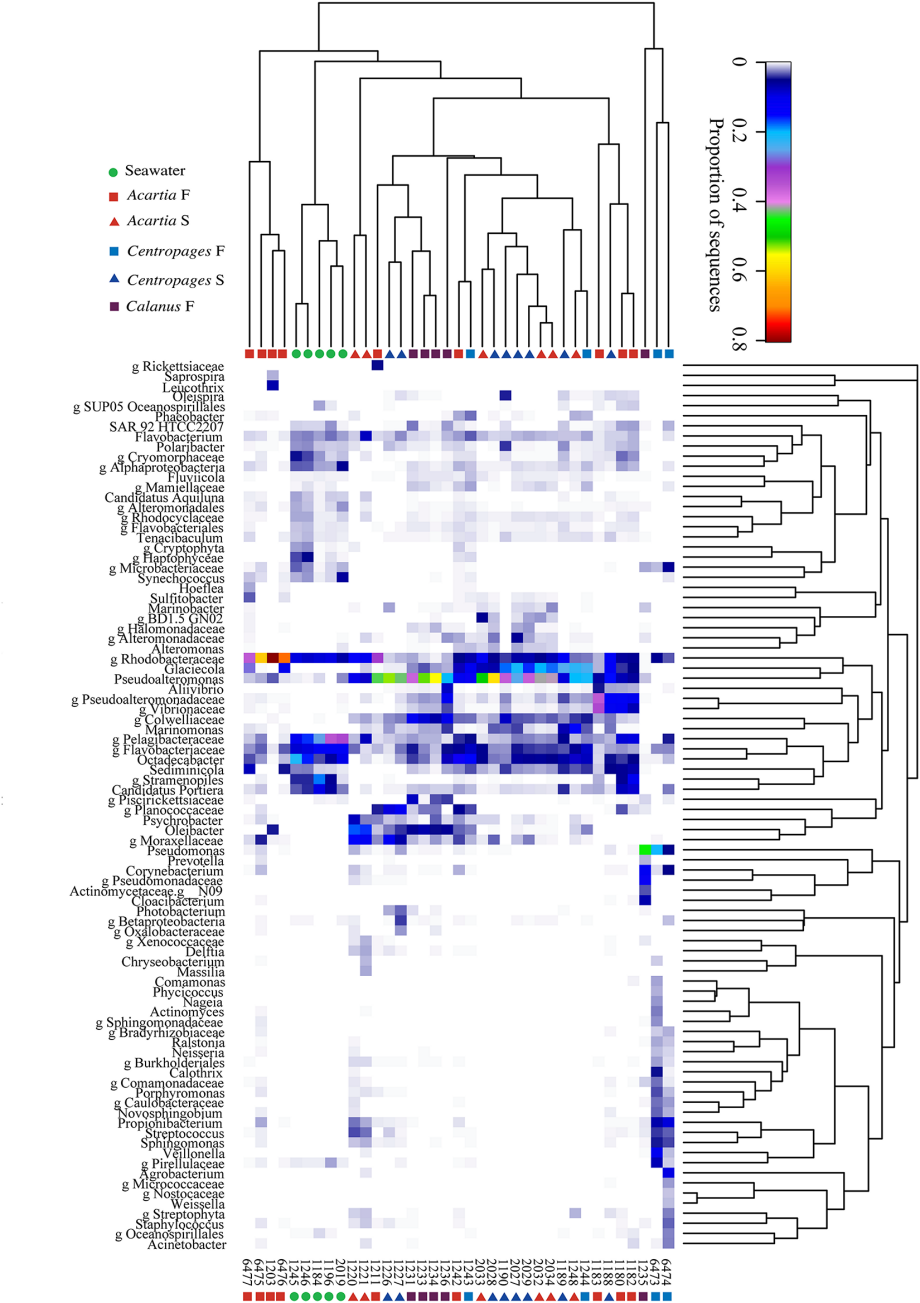
Heatmap with dendrograms (hierarchical clustering based on Bray-Curtis distance) for genus level groupings and samples. F, full gut; S, starved. The dendrogram at the top demonstrates the similarity of communities in several samples of starved *Acartia* and *Centropages*, although time-dependent variability is also present. Several co-varying genera with higher relative abundance in water samples are driving the separation from the other sample types. Higher relative abundance of *Pseudoalteromonas* sp. and several other bacterial genera is seen in copepods in comparison to water samples.

**Fig 4:**
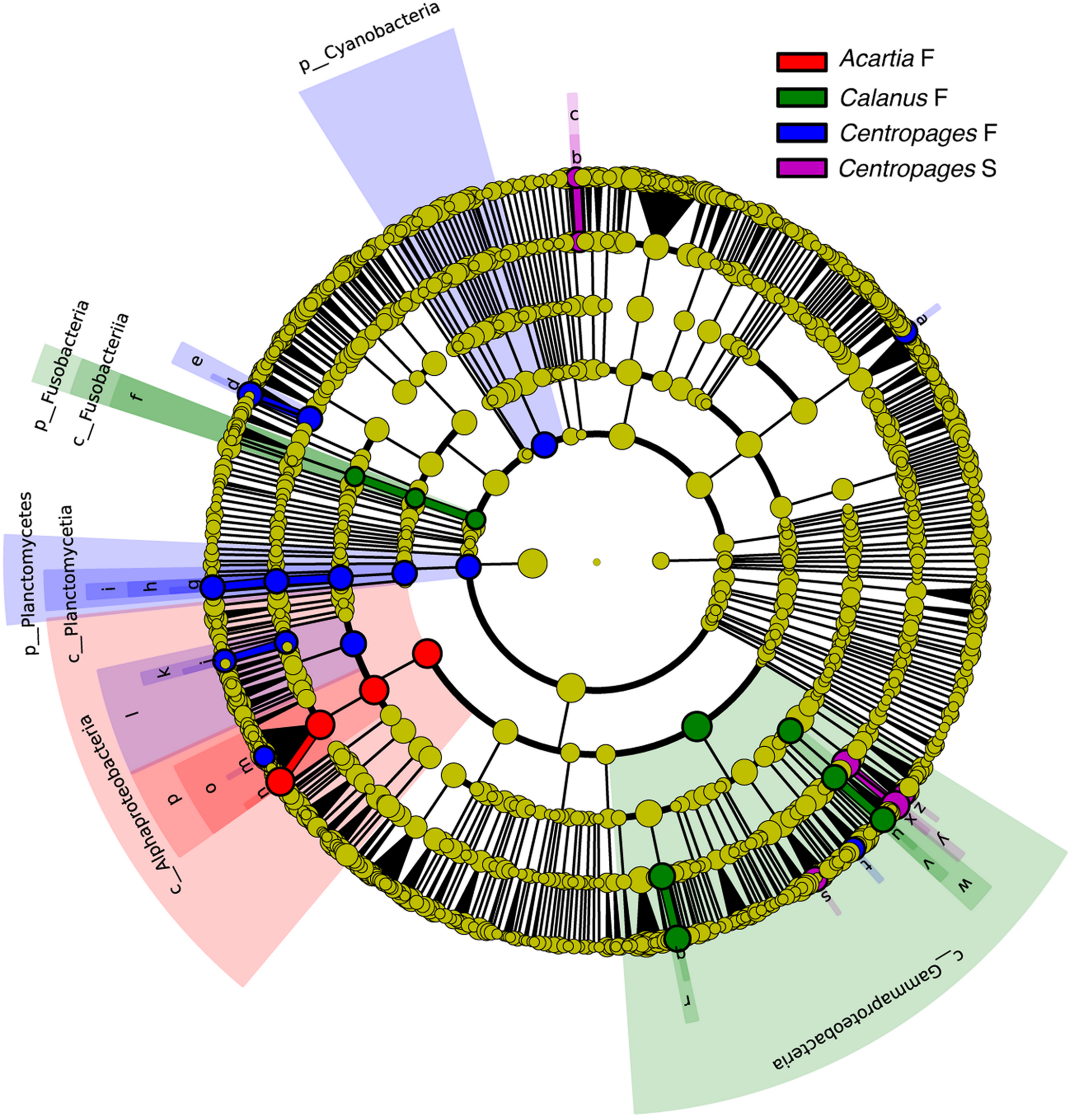
Groups from phylum to genus level determined to be significant representatives of their sample type from the LefSe analysis. In the graph, each ring represents a taxonomic level, with phylum, class, order, family, and genus from center to the periphery, respectively. Each circle is a taxonomic unit found in the dataset, with circles or nodes shown in color where the taxon was a significantly more abundant group. F, full gut; S, starved. ug, unidentified genus; a. ug_Microbacteriaceae b. ug_family NS11_12, c. family NS_12, d. *Veillonella* spp., e. Veillonellaceae, f. Fusobacteriales, g. ug_Pirellulaceae, h. Pirellulaceae, i. Pirellulales, j. ug_Bradyrhizobiaceae, k. Bradyrhizobiaeae, l. Rhizobiales, m. *Paracoccus* spp., n. ug_Rhodobacteraceae, o. Rhodobacteraceae, p. Rhodobacterales, q. ug_Colwelliaceae, r. Colwelliaceae, s. *Marinomonas* spp., t. *Acitenobacter* spp., u. ug_Piscirickettsiaceae, v. Piscirickettsiaceae, w. Thiotrichales, x. *Pseudoalteromonas* spp., y. Pseudoalteromonadaceae, z. *Photobacterium* spp.

Several Gammaproteobacteria were found at higher proportions in copepods than in seawater. The genus *Pseudoalteromonas* formed 7.4±9.5% of Gammaproteobacteria in water and 32±25% in copepods, being the most abundant and consistently dominant Gammaproteobacterium overall in copepods (Fig 3). *Glaciecola* sp. consistently formed a large proportion in copepods (17±21% of Gammaproteobacteria), and <0.5% of Gammaproteobacteria in all water samples. A genus in Colwelliaceae within Alteromonadales had high abundances in both full gut and starved copepods, and appeared to co-vary with abundances of *Pseudoalteromonas* spp. (Fig 3, S3 Table). The most abundant genus within Vibrionaceae formed 3.7±8.3% of all gammaproteobacterial sequences in copepods. *Photobacterium*, however, was a significant group in *Centropages* S (Figs 3 and 4). There were other groups within Gammaproteobacteria that were absent or formed a very small proportion of sequences in seawater, but were present in copepods at times at high proportions. These groups included Halomonadaceae, Moraxellaceae, and *Marinobacter* spp. (Fig 3, S3 Table) that were all found across many replicates in *Centropages* S, but were also found in other copepod types. Within Gammaproteobacteria, *Pseudoalteromonas* sp. and *Marinomonas* sp. were significantly more abundant groups in *Centropages* S, unidentified genera of Colwelliaceae and Piscirickettsiaceae were significantly elevated in *Calanus* F, and an *Acitenobacter* sp. was significantly elevated in *Centropages* F (LefSe, p<0.05, Fig 4).

A few ‘seawater-associated’ groups were also identified within Gammaproteobacteria, having consistently high abundances in seawater samples, and generally a low proportion in copepods, with the exception of a few, mostly full gut copepods (Fig 3). A phylotype identified as *Candidatus* Portiera formed a large component of the Gammaproteobacteria in seawater (27–62%), and in general formed a low proportion (<2%) of the community in copepods, with the exception of a few full gut copepods of all types and one starved *Centropages* where they formed up to 18% of Gammaproteobacteria (S3 Table). The SAR92 clade Gammaproteobacterium, represented by the strain HTCC2207 isolated from the Oregon coast [29], formed 3.3–20.3% of Gammaproteobacteria in water samples and a smaller proportion (0–10%) in copepods, being most abundant in a few full gut copepods of all copepod types.

**Fig 5:**
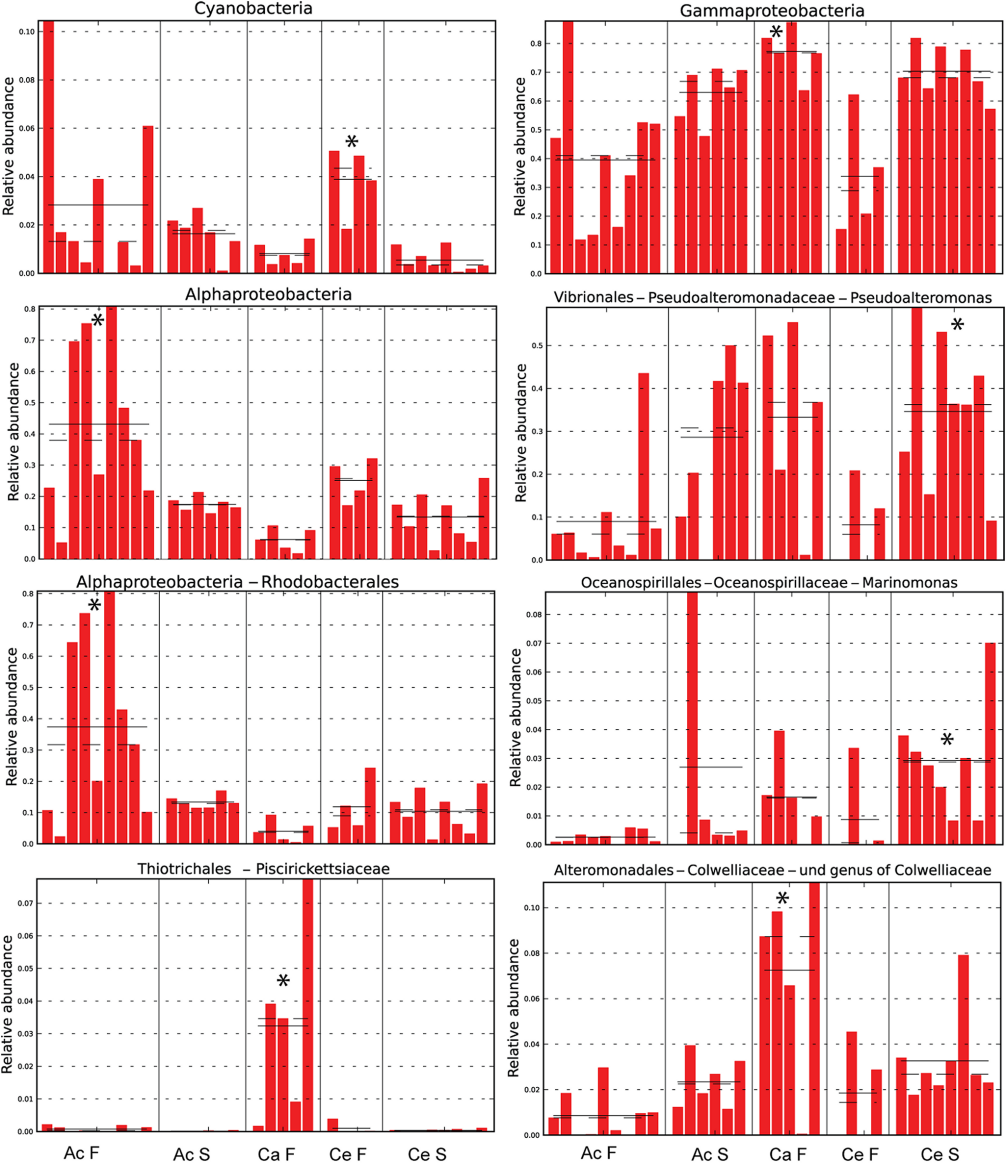
Selected taxa that were significantly more abundant in specific copepod types (LefSe, p<0.05). *, sample type in which the relative abundance of the taxon was significantly elevated. F, full gut; S, starved; Ac, *Acartia*; Ce, *Centropages*; Ca, *Calanus*. In *Acartia* S, no significantly more abundant groups were detected.

Alphaproteobacteria dominated the community in the water samples (48±6.4% of all sequences), and formed a smaller proportion, at 17±2.4% and 13±8.0% of sequences in *Acartia* S and *Centropages* S, respectively. Alphaproteobacteria formed a larger part of the community in *Acartia* F compared to other copepod types (Fig 2, S3 Table). An unidentified genus within Rhodobacteraceae dominated in copepods (39±27% of Alphaproteobacteria in copepods vs. 18±4.0% in seawater) while Pelagibacteraceae dominated in seawater (49±18% of Alphaproteobacteria in seawater vs. 16±13% in copepods) (Figs 3 and 5). The unidentified genus within Rhodobacteraceae was a significantly more abundant group in *Acartia* F than in other copepod types (Figs 4 and 5). A *Paracoccus* sp. and an unidentified genus in Bradyrhizobiaceae were significant alphaproteobacterial groups in *Centropages* F (Fig 4). An *Octadecabacter* sp. was common in all sample types, with 24±18% and 24±16% of Alphaproteobacteria in copepods and seawater, respectively.

Bacteroidetes was the second most abundant phylum in seawater (22±2.0% of all sequences) and the third most abundant phylum in copepods (9.4±4.2%). A dominant sequence type of Flavobacteriaceae persisted across all sample types, and formed on average 33±15% and 50±12% of Bacteroidetes sequences in copepods and water samples, respectively (Fig 3, S3 Table). The other major Bacteroidetes groups also had relatively similar relative abundances in seawater and copepods. The second most common Bacteroidetes in copepods was *Sedinimicola* sp. within Flavobacteria that formed on average 18±17% of the Bacteroidetes sequences in copepods and 6.4±4.0% in water. A *Flavobacterium* sp. formed 14±12% of Bacteroidetes in copepods, and 10±2.0% in water. A *Polaribacter* sp. was the fourth most abundant Bacteroidetes in both copepods and water. *Porphyromonas* sp. − anaerobic bacteria that were the fifth most abundant Bacteroidetes in copepods (3.5±9% of Bacteroidetes) − were not detected in water samples.

Actinobacteria formed 3.6±1.5% of sequences in the water and a more variable proportion among copepod types, with up to 26% in *Centropages* F. A taxon within Acidimicrobiales (Family SC4-41) dominated in copepods; it formed 23±28% of Actinobacteria sequences in copepods, and 7.5±4.4% in water samples. Several additional Actinobacteria did not have clear trends among the sample types (S3 Table). An unidentified genus in Microbacteriaceae was a significantly represented sequence type in *Centropages* F.

Cyanobacteria contributed on average 12% of the sequences in the water samples, and less in copepods, with up to 10% in *Acartia* F and <2.7% in starved copepods. In copepods, most of the Cyanobacteria sequences (~67%) were from microalgal chloroplasts. *Synechococcus* spp. formed 25±33% of Cyanobacteria/Chloroplast sequences in seawater and 4.0±6.1% in copepods. Eukaryotic genera within Stramenopiles and Mamiellaceae were particularly highly represented in seawater and several copepod samples, but the two microalgae seemed to alternate in abundances among the copepod samples (S3 Table). A few other cyanobacterial genera, including *Calothrix* sp., were identified but were present more sporadically.

Sequences from the bacterial phylum Firmicutes made up <0.7% of sequences in the water samples, and up to 15% of the sequences in copepod samples, with full gut individuals generally having higher proportions. Although low in abundance, several Firmicutes were more often detected in copepods compared to seawater. *Planococcus* spp. (Bacillales), Gram-positive aerobes or anaerobes, were the most abundant Firmicutes in copepods, at 31±38% of Firmicutes on average (0% in seawater). A *Veillonella* sp., gram-negative anaerobe (Clostridiales) was a highly represented group in *Centropages* F.

The average proportion of Betaproteobacteria was <5% in all sample types (Fig 2). The most dominant Betaproteobacterial group was a single genus in the Rhodocyclaceae family, at over 50% of all Betaproteobacteria sequences in the seawater and at a high abundance in many copepod samples. An unidentified genus within Methylophilaceae also had a high abundance in all water samples and many copepod samples. Planctomycetes had a very small proportion of the community in starved copepods compared to other sample types, but a genus within Pirellulaceae was significantly elevated in *Centropages* F. Within Deltaproteobacteria, *Bacteriovorax* spp. and another genus within Bdellovibrionales were present at low proportions in only some seawater samples but had elevated proportions in several copepods. Verrucomicrobia were commonly found in copepods and water samples, although their abundance was consistently low within the community (0.2±0.2% and 0.5±0.2% of copepod and seawater sequences, respectively). *Verrucomicrobium* spp. made 10–44% of the Verrucomicrobia sequences in 15 of the copepod samples. *Fusobacterium* spp., and *Leptotrichia* spp., obligate anaerobes, were the most common Fusobacteria, although at very low abundances compared to major sequences in the samples (S3 Table). Fusobacteriales was a significantly represented group in *Calanus* F (Fig 4).

### Comparisons among sample types and time points

**Fig 6:**
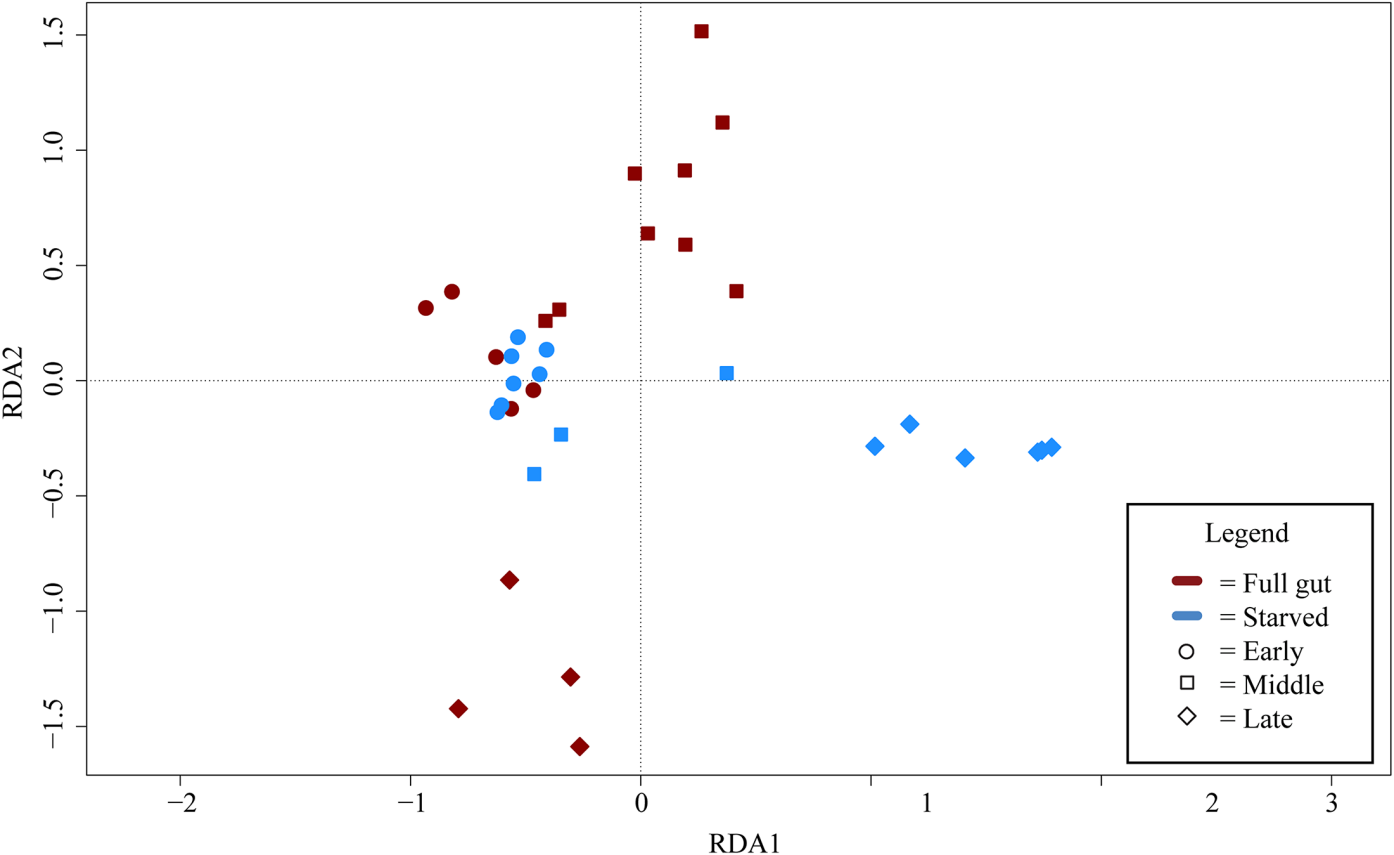
Redundancy analysis for the operational taxonomic unit level microbial community data.

Redundancy analysis showed a separation of full gut and starved communities when all copepod species were pooled (Fig 6). Temporal development in the community was also investigated, by grouping the samples to Early (June 5–11), Mid (June 16–19), and Late (June 23–24) month samples. ANOVA permutation test conducted for the RDA showed that the full gut and starved communities differed (p = 0.031), and sampling date had a significant influence on communities (p = 0.001). Additionally, there was a combination effect of date and treatment (full vs. starved) (p = 0.009; Fig 6).

Multidimensional Scaling (MDS) analysis was conducted to compare communities between the sample types. Communities in seawater were significantly or borderline significantly different from all copepod types (p<0.01 for *Acartia* S, *Centropages* S and F, and *Calanus* F, and p = 0.058 for *Acartia* F; ANOSIM) (S1 Fig). Communities in *Centropages* S were significantly different from *Acartia* F and *Centropages* F (p = 0.044 and p = 0.016, respectively), while communities in *Acartia* S were not significantly different from the communities in any other copepod type. Communities in *Acartia* F and *Centropages* F were not significantly different, and the difference between *Calanus* F and *Acartia* F was borderline significant (p = 0.061, ANOSIM).

## Discussion

### Copepod specific associations

Presence of *A*. *longiremis* and *C*. *hamatus* in the study area in early summer is supported by previous observations in the GoM. Both *A*. *longiremis* and *C*. *hamatus* tend to have high abundances in the spring (May-June) in the Northwestern Atlantic coastal waters, while *C*. *hamatus* abundances decrease in the summer [30, 31]. In a previous study, *A*. *longiremis* dominated in the surface waters in May, and the species dominance switched to *A*. *hudsonica* in late June [32]. Although on the annual basis, *C*. *typicus* dominated over *C*. *hamatus* [33], the latter dominated during spring and early summer in our study area [32]. The low abundances of *C*. *finmarchicus* compared to *Acartia* and *Centropages* in our samples were expected based on past reports in surface waters in the area where *C*. *finmarchicus* has a tendency to remain at deeper depths during summer [32].

The Gammaproteobacterium *Pseudoalteromonas* spp. (Pseudoalteromonadaceae, Vibrionales) was generally the most abundant and consistent group in the starved copepods, suggesting a stable association, and was present in all copepod taxa. Copepod environment may be a key niche for this group in GoM. Sequences within Vibrionaceae (Vibrionales), including *Vibrio* spp., were detected but were unexpectedly low in comparison [34, 35]. Although detected even at low temperatures, abundances of some *Vibrio* spp. have a significant positive correlation with water temperature [36, 37]. Our results suggest that the majority of the bacteria forming stable associations with GoM copepods are *Pseudoaltermonas* spp., however it is possible that *Vibrio* spp. become more abundant later in the summer as water temperatures increase. While *Vibrio* spp. are often thought to form the core copepod microbiome, as recently seen in the subtropical Sargasso Sea waters [14], our results suggest they are replaced by other Gammaproteobacteria during the cold season in temperate waters. Some *Pseudoalteromonas* spp. have chitinases [38], which could provide a carbon source from the copepod exoskeleton, as is the case for many *Vibrio* spp., however our methods cannot distinguish whether the detected bacteria were present as endo- or ectobionts on the copepods. Alteromonadales are also considered R-strategist copiotrophs, that would benefit from a high nutrient environment surrounding the copepod relative to seawater. They also contribute to antifouling on various marine animals [39], and could play such roles also on copepods, which would make the *Pseudoalteromonas*-copepod association mutualistic. Such defensive roles of microbes in animal and plant associations is considered one important form of symbiosis [40].

A few microbial groups had high specificity to one copepod type, indicating taxon-specific niches. *Marinomonas* sp. was a prominent gammaproteobacterial group that was found at a highest relative abundance in *Centropages* S, potentially forming a host-specific association. In addition, many species within *Photobacterium* spp. (Vibrionaceae), observed at high relative abundance in *Centropages* S, have bioluminescent properties, and may play multiple symbiotic, commensal, or pathogenic roles on marine organisms [41]. Genera within Colwelliaceae (Alteromonadales) and Piscirickettsiaceae each were observed that were significantly elevated in *Calanus* F when compared to other copepod sample types. Like many marine bacteria, some *Colwellia* spp. have chitinases [38], that would be beneficial in providing access to carbon on the copepod exoskeleton. Piscirickettsiae family includes several genera with a range of metabolic and ecological characteristics, some being fish pathogens and others that play specific roles in biogeochemical cycling of sulphur and carbon. Further work should investigate the roles of these groups in *C*. *finmarchicus* in more detail.

### Influence of seasonality and food on copepod microbiome

The sampling time coincided with the initiation of summer stratification of the GoM water mass [32]. As temperature increases, the life stage duration in the GoM copepods decreases, with more frequent molting [42]. It is generally assumed that the exterior of the copepod is colonized by bacteria each time after molting, thus some of the temporal changes in the communities detected could be related with the length of time the microbial community had time to colonize since last molting. The dominant groups found on copepods were detected also in seawater, suggesting rapid re-colonization is occurring. An example of such groups was Flavobacteria (Bacteroidetes), frequently recovered from copepods in previous studies [43], but it appeared to have represented a transient community member in copepods in this study, given its high relative abundance in seawater. Several other groups were found at high relative abundances in both seawater and copepods, suggesting the association of several bacterial groups with copepods may be food associated, not host promoted.

Temperature is a major driver of bacterioplankton growth in temperate marine waters, thus the increase in local temperature is likely to have influenced the ambient microbial abundance and growth rates [44]. The community composition in seawater did not show prominent temporal shifts over the study period, however, suggesting any temporal changes on the copepods were not based on major changes in the surrounding bacterial community.

Persistent and significant differences were observed between the communities in full-gut and starved copepods, demonstrating that temporary associations related to food play an important part in the variability of bacterial community found in association of copepods. Observed differences in full gut copepods among copepod species also strongly suggest food preferences influence their microbiomes. The adults, nauplii and copepodite life stages of *C*. *finmarchicus* are important grazers of primary producers and microzooplankton in the North Atlantic waters [45], while *A*. *longiremis* is primarily herbivorous and *C*. *hamatus* utilizes omnivory [46, 47]. Copepod feeding on picoplankton would be an unexpected path in the marine microbial food web but could occur as part of dead or live particles or via aggregate feeding [48, 49]. Direct bacterivory by copepods is generally thought to form only a minor portion of carbon flow in aquatic food webs [50, 51], but the data from this study suggest bacteria associated with food particles form a substantial portion of the copepod microbiome and possibly part of their nutrition. This conclusion is supported by our recent study from coastal North Atlantic waters in which the communities of N_2_-fixing bacteria in full gut copepods (primarily *Acartia*) had high similarity with communities in seawater while the diazotrophic communities in starved *Acartia* spp. were distinct [52]. In this study the particle-associated bacteria could have been associated with phytoplankton or microzooplankton targeted as food, or less likely, recently digested by such microzooplankton. The bacteria could also be in dead particles such as fecal pellets that the copepods consume. Based on this study, in herbivorous and omnivorous copepods, the pathway of carbon from such particle-associated bacteria directly to copepods could be significant.

Besides the direct influence of food associated bacteria, conditions internally in the copepod that are distinct from the surrounding seawater environment in terms of availability of oxygen [53], acidity, and nutrients, could promote a specific microbial community responding to variable food *in situ* [54]. The type and abundance of food resulted in differences in the cultivable bacterial load from *Acartia* [16], suggesting that depending on type and quantity of food, the core microbiome may differentially utilize carbon and nutrients. Changes in food quality during the study may thus have also indirectly contributed to the observed variability. In addition, *C*. *hamatus* can feed on both moving and suspended prey, but male and female clearance and ingestion rates and food selectivities differ [46], which may have induced variability in the data in this study, since females and males were not separated. Further, *C*. *hamatus*, like many other copepods, feed on fecal pellets [55], which could contribute to the exchange of microbial community members across copepod taxa, and could have influenced the fact that some groups known to be free-living (e.g. *Synechococcus* and SAR11) were at times still detected in copepods 24 h after starvation.

Overall our results suggest that these copepods have stable, potentially mutualistic associations with specific groups of Gammaproteobacteria, especially *Pseudoalteromonas* spp., during the early summer in the Gulf of Maine. In addition, there are transient interactions with high abundances of food-associated bacteria, including Rhodobacteraceae in *Acartia* that may contribute to copepod nutrition. Further work should investigate the quantitative importance of such pathway of bacterial carbon to these higher consumers not traditionally viewed as bacterivores. Additionally, there are many less abundant bacterial groups that play potential taxon-specific roles. Overall the results demonstrate the strong connections and multitude of interactions between bacterioplankton and mesozooplankton in temperate marine ecosystems.

## Supporting Information

S1 Fig Multidimensional Scaling Analysis clustering of the samples based on the sequence data. (TIF)

S1 Table Samples included in the study. (PDF)

S2 Table Primers used in this study. (PDF)

S3 Table Genus-level sequence data shown as proportions of all sequences. (PDF)

S1 Text Supplementary methods. (PDF)
