# Supplementary figures and images for: Stable Associations Masked by Temporal Variability in the Marine Copepod Microbiome
Source: PLoS One. 2015 Sep 22;10(9):e0138967. doi: 10.1371/journal.pone.0138967 (PMC4579122; doi:10.1371/journal.pone.0138967)

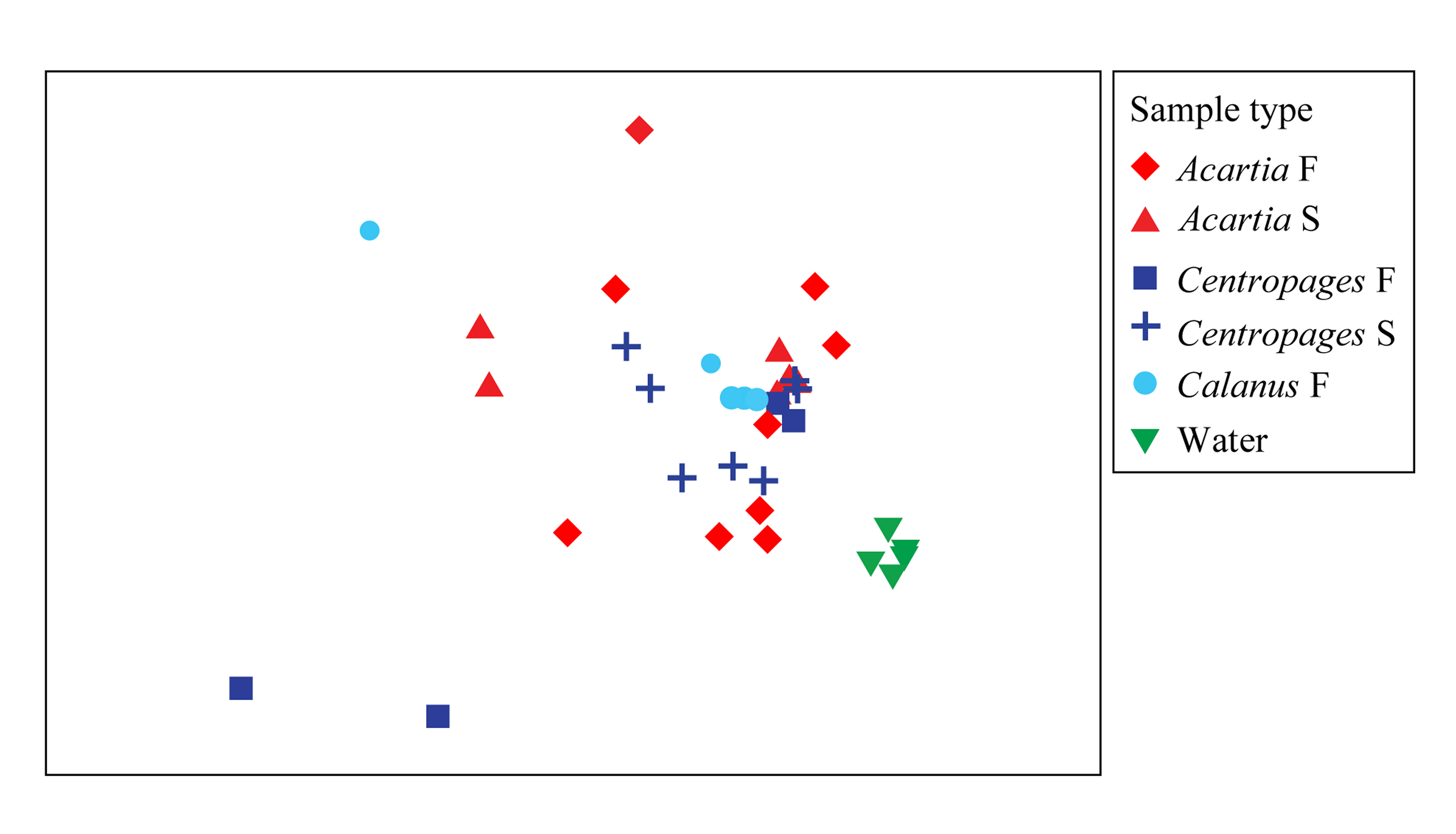

Supplement: S1 Fig — (TIF) [file pone.0138967.s001.tif]
