# Supplementary material for: Stable Associations Masked by Temporal Variability in the Marine Copepod Microbiome
Source: PLoS One. 2015 Sep 22;10(9):e0138967. doi: 10.1371/journal.pone.0138967 (PMC4579122; doi:10.1371/journal.pone.0138967)
Supplement: S1 Table — (PDF) [file pone.0138967.s002.pdf]

S1 Table. Samples included in the study.

Sample #	Date	Corres-ponding water sample ID	Sample type	Treatment	Number of individuals per tube	Seq. run	MiSeq Nextera indices		Number of sequences before removal of negatives	Number of sequences after removal of negatives
							N Index	S Index		
1180	6/5/11	5	*Acartia*	Full gut	5	1	AAGAGGCA	CTCTCTAT	70102	69315
1182	6/5/11	5	*Acartia*	Full gut	5	1	GTAGAGGA	CTCTCTAT	68958	67140
1183	6/5/11	5	*Acartia*	Full gut	5	1	GCTCATGA	CTCTCTAT	50973	49911
1203	6/11/11	9	*Acartia*	Full gut	10	1	ATCTCAGG	CTCTCTAT	50820	50442
1211	6/11/11	9	*Acartia*	Full gut	10	1	TAAGGCGA	TATCCTCT	42070	39499
1242	6/19/11	12	*Acartia*	Full gut	5	2	TAGGCATG	CGTCTAAT	125464	121648
6475	6/19/11	12	*Acartia*	Full gut	4	2	GCTCATGA	CTAAGCCT	57894	43081
6476	6/23/11	15	*Acartia*	Full gut	3	2	ATCTCAGG	CTAAGCCT	120841	118956
6477	6/23/11	15	*Acartia*	Full gut	4	2	TAAGGCGA	CGTCTAAT	132576	126312
1220	6/11/11	9	*Acartia*	Starved	10	1	CTCTCTAC	CTCTCTAT	15521	10752
1221	6/11/11	9	*Acartia*	Starved	10	1	CGAGGCTG	CTCTCTAT	7850	4752
1248	6/19/11	12	*Acartia*	Starved	10	2	CGAGGCTG	CGTCTAAT	107073	106441
2032	6/24/11	15	*Acartia*	Starved	10	1	TAGGCATG	CTCTCTAT	57869	57796

2033	6/24/11	15	*Acartia*	Starved	10	1	TCCTGAGC	CTCTCTAT	61287	60990
2034	6/24/11	15	*Acartia*	Starved	10	1	GGACTCCT	CTCTCTAT	58223	58129
1231	6/17/11	11	*Calanus*	Full gut	1	1	AAGAGGCA	TATCCTCT	75614	74977
1233	6/17/11	11	*Calanus*	Full gut	1	1	GTAGAGGA	TATCCTCT	76232	74622
1234	6/17/11	11	*Calanus*	Full gut	1	1	GCTCATGA	TATCCTCT	80760	79437
1235	6/17/11	11	*Calanus*	Full gut	6	1	ATCTCAGG	TATCCTCT	20523	18415
1236	6/17/11	11	*Calanus*	Full gut	6	1	TAAGGCGA	GTAAGGAG	78474	77906
1243	6/19/11	12	*Centropages*	Full gut	5	1	CTCTCTAC	TATCCTCT	58070	56804
1244	6/19/11	12	*Centropages*	Full gut	5	1	CGAGGCTG	TATCCTCT	81197	80786
6473	6/5/11	6	*Centropages*	Full gut	3	2	AAGAGGCA	CTAAGCCT	9329	1937
6474	6/5/11	6	*Centropages*	Full gut	4	2	GTAGAGGA	CTAAGCCT	31725	14044
1188	6/8/11	6	*Centropages*	Starved	4	2	CGTACTAG	CGTCTAAT	57354	50420
1189	6/8/11	6	*Centropages*	Starved	4	1	TAAGGCGA	CTCTCTAT	54905	54186
1190	6/8/11	6	*Centropages*	Starved	4	2	TCCTGAGC	CGTCTAAT	131992	129572
1226	6/11/11	9	*Centropages*	Starved	10	1	GGACTCCT	TATCCTCT	55467	51929
1227	6/11/11	9	*Centropages*	Starved	10	1	TAGGCATG	TATCCTCT	51429	48583
2027	6/24/11	15	*Centropages*	Starved	10	1	CGTACTAG	TATCCTCT	61162	61055

2028	6/24/11	15	*Centropages*	Starved	10	1	AGGCAGAA	TATCCTCT	71787	71738
2029	6/24/11	15	*Centropages*	Starved	10	1	TCCTGAGC	TATCCTCT	70355	70212
1184	6/5/11	5	Water	N/A	N/A	2	AGGCAGAA	CGTCTAAT	73893	73892
1196	6/10/11	9	Water	N/A	N/A	2	GGACTCCT	CGTCTAAT	67898	67898
1245	6/19/11	12	Water	N/A	N/A	1	CGTACTAG	CTCTCTAT	33456	33456
1246	6/17/11	11	Water	N/A	N/A	2	CTCTCTAC	CGTCTAAT	75569	75567
2019	6/23/11	15	Water	N/A	N/A	1	AGGCAGAA	CTCTCTAT	32689	32688
Neg1	11/14	N/A	Negative	N/A	N/A	1	CGTACTAG	GTAAGGAG	941	N/A
Neg2	12/14	N/A	Negative	N/A	N/A	2	GCTCATGA	CGTCTAAT	743	N/A
