# Supplementary material for: Stable Associations Masked by Temporal Variability in the Marine Copepod Microbiome
Source: PLoS One. 2015 Sep 22;10(9):e0138967. doi: 10.1371/journal.pone.0138967 (PMC4579122; doi:10.1371/journal.pone.0138967)
Supplement: S2 Table — (PDF) [file pone.0138967.s003.pdf]

S2 Table. Primers used in this study.

Target	Primer name	Primer sequence	Length of amplicon	Reference
Mitochondrial cytochrome oxidase c subunit I	LCO1490F	5'-GGTCAACAAATCATAAAGATATTGG-3'	710 bp	Folmer 1994
	HCO2198R	5'-TAAACTTCAGGGTGACCAAAAAATCA-3'		
16S rRNA gene	Bact 341F	5’-CCT ACG GGN GGC WGC AG-3’	464 bp	Klindworth et al. 2013
	Bact 785R	5’-GAC TAC HVG GGT ATC TAA TCC-3’		
16S rRNA gene	Bact 341F_Overhang	5’-TCG TCG GCA GCG TCA GAT GTG TAT AAG AGA CAG CCT ACG GGN GGC WGC AG-3’	464 bp	Illumina, Inc., 2014
	Bact 785R_Overhang	5’-GTC TCG TGG GCT CGG AGA TGT GTA TAA GAG ACA GGA CTA CHV GGG TAT CTA ATC C-3’		
