# Supplementary material for: Stable Associations Masked by Temporal Variability in the Marine Copepod Microbiome
Source: PLoS One. 2015 Sep 22;10(9):e0138967. doi: 10.1371/journal.pone.0138967 (PMC4579122; doi:10.1371/journal.pone.0138967)
Supplement: S3 Table — (PDF) [file pone.0138967.s004.pdf]

S3 Table. Genus-level sequence data shown as proportions of all sequences.

| Taxon | 1180 | 1182 | 1183 | 1203 | 1211 | 1242 | 6475 | 6476 | 6477 | 1220 | 1221 | 1248 | 2032 | 2033 | 2034 |
|---|---|---|---|---|---|---|---|---|---|---|---|---|---|---|---|
| Copepod genus | Acartia | Acartia | Acartia | Acartia | Acartia | Acartia | Acartia | Acartia | Acartia | Acartia | Acartia | Acartia | Acartia | Acartia | Acartia |
| Type of sample | FullGut | FullGut | FullGut | FullGut | FullGut | FullGut | FullGut | FullGut | FullGut | Starved | Starved | Starved | Starved | Starved | Starved |
| k__Archaea;p__Crenarchaeota;c__Thaumarchaeota;o__Cenarchaeales;f__Cenarchaeaceae;g__Nitrosopumilus | 0 | 0.00013405 | 0 | 0 | 0 | 0 | 0 | 0 | 0 | 0 | 0 | 0 | 0 | 0 | 0 |
| k__Archaea;p__Euryarchaeota;c__Thermoplasmata;o__E2;f__Marine group II;g__ | 0.00020198 | 0.00044683 | 0.00010018 | 1.98E-05 | 0 | 0 | 0 | 0 | 3.96E-05 | 0 | 0 | 3.76E-05 | 3.46E-05 | 4.92E-05 | 1.72E-05 |
| k__Bacteria;p__AC1;c__SHA-114;o__;f__;g__ | 0 | 0 | 0 | 0 | 0 | 0 | 0 | 0 | 0 | 0 | 0 | 0 | 0 | 0 | 0 |
| k__Bacteria;p__Acidobacteria;c__;o__;f__;g__ | 0 | 0 | 0 | 0 | 0 | 0 | 0 | 0 | 0 | 0 | 0 | 0 | 0 | 0 | 0 |
| k__Bacteria;p__Acidobacteria;c__Acidobacteria-6;o__iii1-15;f__;g__ | 0 | 0 | 0 | 0 | 0 | 0 | 0 | 0 | 6.33E-05 | 0.00762649 | 0 | 0 | 0 | 0 | 0 |
| k__Bacteria;p__Acidobacteria;c__Acidobacteriia;o__Acidobacteriales;f__Acidobacteriaceae;g__ | 0.00014427 | 0 | 0 | 0 | 0 | 0 | 0 | 0 | 0 | 0 | 0 | 0 | 0 | 0 | 0 |
| k__Bacteria;p__Acidobacteria;c__Acidobacteriia;o__Acidobacteriales;f__Koribacteraceae;g__ | 0 | 0 | 0 | 0 | 0 | 0 | 0 | 0 | 0 | 0 | 0 | 0 | 0 | 0 | 0 |
| k__Bacteria;p__Acidobacteria;c__Acidobacteriia;o__Acidobacteriales;f__Koribacteraceae;g__Candidatus Koribacter | 0.00018755 | 0 | 0 | 0 | 0 | 0 | 0 | 0 | 0 | 0 | 0 | 0 | 0 | 0 | 0 |
| k__Bacteria;p__Acidobacteria;c__OS-K;o__;f__;g__ | 0 | 0 | 0 | 0 | 0 | 0 | 0 | 0 | 0 | 0 | 0 | 0 | 0 | 0 | 0 |
| k__Bacteria;p__Acidobacteria;c__Solibacteres;o__Solibacterales;f__;g__ | 1.44E-05 | 0 | 0 | 0 | 0 | 0 | 0 | 0 | 0 | 0 | 0 | 0 | 0 | 0 | 0 |
| k__Bacteria;p__Acidobacteria;c__Solibacteres;o__Solibacterales;f__PAUC26f;g__ | 0 | 0 | 0 | 0 | 0 | 0 | 0 | 0 | 0 | 0 | 0 | 0 | 0 | 0 | 0 |
| k__Bacteria;p__Acidobacteria;c__Sva0725;o__Sva0725;f__;g__ | 0 | 0 | 0 | 0 | 0 | 0 | 0 | 0 | 0 | 0 | 0 | 0 | 0 | 0 | 0 |
| k__Bacteria;p__Acidobacteria;c__[Chloracidobacteria];o__PK29;f__;g__ | 0 | 0 | 0 | 0 | 0 | 0 | 0 | 0 | 0 | 0.00083705 | 0 | 0 | 0 | 0 | 0 |
| k__Bacteria;p__Acidobacteria;c__[Chloracidobacteria];o__RB41;f__;g__ | 0 | 0 | 0 | 0 | 0 | 0 | 0 | 0 | 0 | 0 | 0 | 0 | 0 | 0 | 0 |
| k__Bacteria;p__Actinobacteria;c__Acidimicrobiia;o__Acidimicrobiales;f__;g__ | 0 | 0 | 0 | 0 | 0 | 0.00046034 | 0 | 0 | 0 | 0 | 0 | 0 | 0 | 0 | 0 |
| k__Bacteria;p__Actinobacteria;c__Acidimicrobiia;o__Acidimicrobiales;f__C111;g__ | 0.00043281 | 0 | 0 | 1.98E-05 | 0 | 0 | 0 | 0.00010088 | 0.00045126 | 0.00855655 | 0 | 0.00014092 | 0.00017302 | 0.00026234 | 0.00013762 |
| k__Bacteria;p__Actinobacteria;c__Acidimicrobiia;o__Acidimicrobiales;f__JdFBGBact;g__ | 0 | 0 | 0 | 0 | 0 | 0.00014797 | 0 | 0 | 0 | 0 | 0 | 6.58E-05 | 0 | 0 | 0 |
| k__Bacteria;p__Actinobacteria;c__Acidimicrobiia;o__Acidimicrobiales;f__Microthrixaceae;g__ | 0 | 0 | 0 | 0 | 0 | 0 | 0 | 0 | 0 | 0 | 0 | 0 | 0 | 0 | 0 |
| k__Bacteria;p__Actinobacteria;c__Acidimicrobiia;o__Acidimicrobiales;f__OCS155;g__ | 0.00050494 | 0.00046172 | 2.00E-05 | 0 | 0 | 0.00105222 | 0 | 0 | 0 | 0 | 0 | 0.00046974 | 0.00057097 | 0.00016396 | 0.00036127 |
| k__Bacteria;p__Actinobacteria;c__Acidimicrobiia;o__Acidimicrobiales;f__SC3-41;g__ | 0.0001587 | 0 | 0.00032057 | 0 | 0 | 0.00409378 | 0 | 0.00019335 | 0.00123504 | 0 | 0 | 0.00271512 | 0.00396221 | 0.00459092 | 0.0038707 |
| k__Bacteria;p__Actinobacteria;c__Acidimicrobiia;o__Acidimicrobiales;f__TK06;g__ | 0 | 0 | 0 | 0 | 0 | 0 | 0 | 0 | 0 | 0 | 0 | 0 | 0 | 0 | 0 |
| k__Bacteria;p__Actinobacteria;c__Acidimicrobiia;o__Acidimicrobiales;f__ZA3409c;g__ | 0 | 0 | 0 | 0 | 0 | 0 | 0 | 0 | 0 | 0 | 0 | 0 | 0 | 0 | 0 |
| k__Bacteria;p__Actinobacteria;c__Acidimicrobiia;o__Acidimicrobiales;f__koll13;g__ | 0 | 0 | 0 | 0 | 0 | 0 | 0 | 0 | 0 | 0 | 0 | 0 | 0 | 0 | 0 |
| k__Bacteria;p__Actinobacteria;c__Acidimicrobiia;o__Acidimicrobiales;f__ntu14;g__ | 0 | 0 | 0 | 0 | 0 | 0 | 0 | 0 | 0 | 0 | 0 | 0 | 0 | 0 | 0 |
| k__Bacteria;p__Actinobacteria;c__Acidimicrobiia;o__Acidimicrobiales;f__wb1_P06;g__ | 0 | 0 | 0 | 0 | 0 | 0.00014797 | 0 | 0 | 0 | 0 | 0 | 9.39E-05 | 8.65E-05 | 6.56E-05 | 0.00010322 |
| k__Bacteria;p__Actinobacteria;c__Actinobacteria;o__Actinomycetales;f__;g__ | 0.00090889 | 0.00108728 | 0.00030053 | 0 | 0 | 0.00665034 | 0.00208909 | 0.00037829 | 4.75E-05 | 0.00269717 | 0 | 0.00033822 | 1.73E-05 | 6.56E-05 | 8.60E-05 |
| k__Bacteria;p__Actinobacteria;c__Actinobacteria;o__Actinomycetales;f__ACK-M1;g__ | 0 | 0 | 0 | 0 | 0 | 0 | 0 | 3.36E-05 | 0 | 0 | 0 | 0 | 0 | 0 | 0 |
| k__Bacteria;p__Actinobacteria;c__Actinobacteria;o__Actinomycetales;f__Actinomycetaceae;g__ | 0 | 0 | 0 | 0 | 0 | 0 | 0 | 0 | 0 | 0 | 0 | 0 | 0 | 0 | 0 |
| k__Bacteria;p__Actinobacteria;c__Actinobacteria;o__Actinomycetales;f__Actinomycetaceae;g__Actinomyces | 7.21E-05 | 0 | 0 | 0 | 0 | 0.00012331 | 0.00208909 | 0 | 0 | 0 | 0.00147306 | 0 | 0 | 0 | 6.88E-05 |
| k__Bacteria;p__Actinobacteria;c__Actinobacteria;o__Actinomycetales;f__Actinomycetaceae;g__Mobiluncus | 0 | 0 | 0 | 0 | 0 | 0 | 0 | 0 | 0 | 0 | 0 | 0 | 0 | 0 | 0 |
| k__Bacteria;p__Actinobacteria;c__Actinobacteria;o__Actinomycetales;f__Actinomycetaceae;g__N09 | 0.00011542 | 0 | 0 | 0 | 0.00050634 | 0 | 0 | 0 | 0 | 0 | 0 | 0 | 0 | 0 | 0 |
| k__Bacteria;p__Actinobacteria;c__Actinobacteria;o__Actinomycetales;f__Actinomycetaceae;g__Varibaculum | 0 | 0 | 0 | 0 | 0 | 0 | 0 | 0 | 0 | 0 | 0 | 0 | 0 | 0 | 0 |
| k__Bacteria;p__Actinobacteria;c__Actinobacteria;o__Actinomycetales;f__Brevibacteriaceae;g__Brevibacterium | 0 | 0 | 0.00014025 | 0 | 0 | 0 | 0 | 0 | 0 | 0 | 0 | 0 | 0 | 0 | 5.16E-05 |
| k__Bacteria;p__Actinobacteria;c__Actinobacteria;o__Actinomycetales;f__Cellulomonadaceae;g__Actinotalea | 0 | 0 | 0 | 0 | 0 | 0 | 0 | 0 | 0 | 0 | 0 | 0 | 0 | 0 | 0 |
| k__Bacteria;p__Actinobacteria;c__Actinobacteria;o__Actinomycetales;f__Cellulomonadaceae;g__Cellulomonas | 0 | 0 | 0 | 0 | 0 | 0 | 0 | 0 | 0 | 0 | 0 | 0 | 0 | 0 | 0 |
| k__Bacteria;p__Actinobacteria;c__Actinobacteria;o__Actinomycetales;f__Cellulomonadaceae;g__Demequina | 0 | 4.47E-05 | 0 | 0 | 0 | 0 | 0 | 0 | 0 | 0 | 0 | 0 | 0 | 0 | 0 |
| k__Bacteria;p__Actinobacteria;c__Actinobacteria;o__Actinomycetales;f__Corynebacteriaceae;g__Corynebacterium | 0.00174565 | 0.00067024 | 0.00032057 | 0.0001586 | 0.00030381 | 0.01561061 | 0.01313804 | 0.00076499 | 0.0012667 | 0.01134673 | 0.00462963 | 6.58E-05 | 3.46E-05 | 0.00067224 | 0.00020644 |
| k__Bacteria;p__Actinobacteria;c__Actinobacteria;o__Actinomycetales;f__Dermabacteraceae;g__Brachybacterium | 0 | 0.00274054 | 0 | 0 | 0 | 0 | 0 | 0 | 0 | 0 | 0 | 0 | 0 | 0 | 0 |
| k__Bacteria;p__Actinobacteria;c__Actinobacteria;o__Actinomycetales;f__Dermacoccaceae;g__Dermacoccus | 0 | 0 | 0 | 0 | 0.00010127 | 0 | 0 | 0.00015972 | 0 | 0 | 0 | 0 | 0 | 0 | 0 |
| k__Bacteria;p__Actinobacteria;c__Actinobacteria;o__Actinomycetales;f__Dietziaceae;g__Dietzia | 0 | 0.00131069 | 0 | 0 | 0.00037976 | 0 | 0 | 0 | 0 | 0 | 0 | 0 | 0 | 0 | 0 |
| k__Bacteria;p__Actinobacteria;c__Actinobacteria;o__Actinomycetales;f__Frankiaceae;g__ | 0 | 0 | 0 | 0 | 0 | 0 | 0 | 0 | 0 | 0 | 0 | 0 | 0 | 0 | 0 |
| k__Bacteria;p__Actinobacteria;c__Actinobacteria;o__Actinomycetales;f__Geodermatophilaceae;g__ | 0 | 0 | 0 | 0 | 0 | 0.00011509 | 0 | 0 | 0 | 0 | 0 | 0 | 0 | 0 | 0 |
| k__Bacteria;p__Actinobacteria;c__Actinobacteria;o__Actinomycetales;f__Geodermatophilaceae;g__Blastococcus | 0 | 0 | 0 | 0 | 0 | 0 | 0 | 0 | 0 | 0 | 0 | 0 | 0 | 0 | 1.72E-05 |
| k__Bacteria;p__Actinobacteria;c__Actinobacteria;o__Actinomycetales;f__Gordoniaceae;g__Gordonia | 4.33E-05 | 0 | 0 | 0 | 0 | 0 | 0 | 0 | 0 | 0 | 0 | 0 | 0 | 0 | 0 |
| k__Bacteria;p__Actinobacteria;c__Actinobacteria;o__Actinomycetales;f__Intrasporangiaceae;g__ | 4.33E-05 | 0 | 0 | 0 | 0 | 0 | 0 | 6.73E-05 | 0 | 0 | 0 | 0 | 0 | 0 | 0 |
| k__Bacteria;p__Actinobacteria;c__Actinobacteria;o__Actinomycetales;f__Intrasporangiaceae;g__Knoellia | 0 | 0 | 0 | 0 | 0 | 0 | 0 | 0 | 0 | 0 | 0 | 0 | 0 | 0 | 0 |
| k__Bacteria;p__Actinobacteria;c__Actinobacteria;o__Actinomycetales;f__Intrasporangiaceae;g__Oryzihumus | 0 | 0 | 0 | 0 | 0 | 0.00035348 | 0 | 0 | 0 | 0 | 0 | 0 | 0 | 0 | 0 |
| k__Bacteria;p__Actinobacteria;c__Actinobacteria;o__Actinomycetales;f__Intrasporangiaceae;g__Phycicoccus | 0 | 0 | 0 | 0 | 0 | 0 | 0 | 2.52E-05 | 0 | 0 | 0 | 0 | 0 | 0 | 0 |
| k__Bacteria;p__Actinobacteria;c__Actinobacteria;o__Actinomycetales;f__Intrasporangiaceae;g__Tetrasphaera | 0 | 0 | 0 | 0 | 0 | 0 | 0 | 0.00029423 | 0 | 0 | 0 | 0 | 0 | 0 | 0 |
| k__Bacteria;p__Actinobacteria;c__Actinobacteria;o__Actinomycetales;f__Kineosporiaceae;g__ | 0 | 0 | 0 | 0 | 0 | 0 | 0 | 0 | 0 | 0 | 0 | 0 | 0 | 0 | 0 |
| k__Bacteria;p__Actinobacteria;c__Actinobacteria;o__Actinomycetales;f__Microbacteriaceae;g__ | 0.00047609 | 0.00022341 | 0.00032057 | 0 | 0.00075951 | 0.00178384 | 0.00875096 | 0.00169811 | 0.00235132 | 0 | 0.00021044 | 0.00044156 | 8.65E-05 | 0.00026234 | 5.16E-05 |
| k__Bacteria;p__Actinobacteria;c__Actinobacteria;o__Actinomycetales;f__Microbacteriaceae;g__Agrococcus | 0 | 5.96E-05 | 0 | 0 | 0 | 0 | 0 | 0 | 0 | 0 | 0 | 0 | 0 | 0 | 0 |
| k__Bacteria;p__Actinobacteria;c__Actinobacteria;o__Actinomycetales;f__Microbacteriaceae;g__Candidatus Aquiluna | 0.00126957 | 0.00089366 | 0.00080143 | 0 | 0 | 0.00274563 | 0.00283187 | 0.00024379 | 0.0037922 | 0 | 0.01031145 | 0.00076098 | 0.00036335 | 0.00047549 | 0.00043008 |
| k__Bacteria;p__Actinobacteria;c__Actinobacteria;o__Actinomycetales;f__Microbacteriaceae;g__Candidatus Rhodoluna | 0 | 0 | 0 | 0 | 0 | 0 | 0 | 0 | 0 | 0 | 0 | 0 | 0 | 0 | 0 |
| k__Bacteria;p__Actinobacteria;c__Actinobacteria;o__Actinomycetales;f__Microbacteriaceae;g__Cryocola | 0 | 0 | 0 | 0 | 0 | 0 | 0 | 0 | 0 | 0.00074405 | 0 | 0 | 0 | 3.28E-05 | 0 |
| k__Bacteria;p__Actinobacteria;c__Actinobacteria;o__Actinomycetales;f__Microbacteriaceae;g__Curtobacterium | 0 | 0 | 0 | 0 | 0 | 0 | 0 | 0 | 0 | 0 | 0 | 0 | 0 | 0 | 0 |
| k__Bacteria;p__Actinobacteria;c__Actinobacteria;o__Actinomycetales;f__Microbacteriaceae;g__Frigoribacterium | 7.21E-05 | 0 | 0.00012021 | 0 | 0 | 0 | 0 | 0 | 0 | 0 | 0 | 0 | 0 | 0 | 0 |
| k__Bacteria;p__Actinobacteria;c__Actinobacteria;o__Actinomycetales;f__Microbacteriaceae;g__Herbiconiux | 0 | 0 | 0 | 0 | 0 | 0 | 0 | 0 | 0 | 0 | 0 | 0 | 0 | 0 | 0 |
| k__Bacteria;p__Actinobacteria;c__Actinobacteria;o__Actinomycetales;f__Microbacteriaceae;g__Microbacterium | 1.44E-05 | 0.00040214 | 0 | 1.98E-05 | 0 | 8.22E-05 | 0 | 0 | 0 | 0 | 0.0016835 | 0 | 0 | 0 | 0 |
| k__Bacteria;p__Actinobacteria;c__Actinobacteria;o__Actinomycetales;f__Microbacteriaceae;g__Mycetocola | 0 | 0 | 0 | 0 | 0 | 0 | 0 | 0 | 0 | 0 | 0 | 0 | 0 | 0 | 0 |
| k__Bacteria;p__Actinobacteria;c__Actinobacteria;o__Actinomycetales;f__Microbacteriaceae;g__Pseudoclavibacter | 0 | 0 | 0.00024043 | 0 | 0 | 0 | 0 | 0 | 0 | 0 | 0 | 0 | 0 | 0 | 0 |
| k__Bacteria;p__Actinobacteria;c__Actinobacteria;o__Actinomycetales;f__Microbacteriaceae;g__Salinibacterium | 0 | 0 | 0 | 0 | 0 | 9.04E-05 | 0 | 0.00023538 | 0 | 0.00213914 | 0 | 0 | 0 | 0 | 0 |
| k__Bacteria;p__Actinobacteria;c__Actinobacteria;o__Actinomycetales;f__Microbacteriaceae;g__Yonghaparkia | 0 | 0 | 0 | 0 | 0 | 0 | 0 | 0 | 0 | 0 | 0 | 0 | 0 | 0 | 0 |
| k__Bacteria;p__Actinobacteria;c__Actinobacteria;o__Actinomycetales;f__Micrococcaceae;g__ | 0.00040395 | 0 | 0 | 7.93E-05 | 0.00075951 | 0.00033704 | 0 | 0.00053801 | 0 | 0 | 0 | 0 | 0 | 0 | 5.16E-05 |
| k__Bacteria;p__Actinobacteria;c__Actinobacteria;o__Actinomycetales;f__Micrococcaceae;g__Arthrobacter | 0 | 0.0002681 | 0 | 3.96E-05 | 0 | 0.00042746 | 0 | 0 | 0 | 0 | 0 | 0 | 0 | 0 | 0 |
| k__Bacteria;p__Actinobacteria;c__Actinobacteria;o__Actinomycetales;f__Micrococcaceae;g__Kocuria | 0 | 0 | 0 | 0 | 0 | 0 | 0 | 0.00010928 | 0 | 0 | 0 | 0 | 0 | 0 | 0 |
| k__Bacteria;p__Actinobacteria;c__Actinobacteria;o__Actinomycetales;f__Micrococcaceae;g__Microbispora | 0 | 0 | 0 | 0 | 0 | 0 | 0 | 0 | 0 | 0 | 0 | 0 | 0 | 0 | 0 |
| k__Bacteria;p__Actinobacteria;c__Actinobacteria;o__Actinomycetales;f__Micrococcaceae;g__Micrococcus | 0.00010099 | 0 | 0 | 0 | 0 | 0 | 0 | 0.00021857 | 0.00057002 | 0 | 0 | 0 | 0 | 0 | 0 |
| k__Bacteria;p__Actinobacteria;c__Actinobacteria;o__Actinomycetales;f__Micrococcaceae;g__Renibacterium | 0 | 0 | 0 | 0 | 0 | 0 | 0 | 0 | 0 | 0 | 0 | 0 | 0 | 0 | 0 |
| k__Bacteria;p__Actinobacteria;c__Actinobacteria;o__Actinomycetales;f__Micrococcaceae;g__Rothia | 4.33E-05 | 0 | 0.00032057 | 0 | 0 | 0 | 0 | 0.00016813 | 0 | 0.00269717 | 0.00210438 | 0 | 0 | 0 | 1.72E-05 |
| k__Bacteria;p__Actinobacteria;c__Actinobacteria;o__Actinomycetales;f__Micromonosporaceae;g__ | 0 | 0 | 0 | 0 | 0 | 0 | 0 | 0 | 0 | 0.0063244 | 0 | 0 | 0 | 0 | 0 |
| k__Bacteria;p__Actinobacteria;c__Actinobacteria;o__Actinomycetales;f__Micromonosporaceae;g__Phytohabitans | 0 | 0 | 0 | 0 | 0 | 0 | 0 | 0 | 0 | 0 | 0 | 0 | 0 | 0 | 0 |
| k__Bacteria;p__Actinobacteria;c__Actinobacteria;o__Actinomycetales;f__Micromonosporaceae;g__Verrucosispora | 0 | 0 | 0 | 0 | 0 | 0 | 0 | 0 | 0 | 9.30E-05 | 0 | 0 | 0 | 0 | 0 |

| Taxon | | 1180 | 1182 | 1183 | 1203 | 1211 | 1242 | 6475 | 6476 | 6477 | 1220 | 1221 | 1248 | 2032 | 2033 | 2034 |
|---|---|---|---|---|---|---|---|---|---|---|---|---|---|---|---|---|
| | Copepod genus | Acartia | Acartia | Acartia | Acartia | Acartia | Acartia | Acartia | Acartia | Acartia | Acartia | Acartia | Acartia | Acartia | Acartia | Acartia |
| | Type of sample | FullGut | FullGut | FullGut | FullGut | FullGut | FullGut | FullGut | FullGut | FullGut | Starved | Starved | Starved | Starved | Starved | Starved |
| k__Bacteria;p__Actinobacteria;c__Actinobacteria;o__Actinomycetales;f__Mycobacteriaceae;g__Mycobacterium | | 0 | 0 | 0 | 0 | 0 | 0.00037814 | 0 | 0 | 0 | 0.00018601 | 0.0016835 | 0 | 0 | 0 | 0 |
| k__Bacteria;p__Actinobacteria;c__Actinobacteria;o__Actinomycetales;f__Nakamurellaceae;g__ | | 0 | 0 | 0 | 0 | 0 | 0 | 0 | 0 | 0 | 0 | 0 | 0 | 0 | 0 | 0 |
| k__Bacteria;p__Actinobacteria;c__Actinobacteria;o__Actinomycetales;f__Nocardiaceae;g__ | | 0 | 0 | 0 | 0 | 0 | 0 | 0 | 0 | 0 | 0 | 0 | 0 | 0 | 0 | 0 |
| k__Bacteria;p__Actinobacteria;c__Actinobacteria;o__Actinomycetales;f__Nocardiaceae;g__Rhodococcus | | 0 | 0 | 0.00036064 | 0 | 0 | 0.0004439 | 0 | 0.00010088 | 0 | 0 | 0 | 0 | 0 | 0 | 0 |
| k__Bacteria;p__Actinobacteria;c__Actinobacteria;o__Actinomycetales;f__Nocardioidaceae;g__ | | 0.00012984 | 4.47E-05 | 0 | 0 | 0 | 0.00024661 | 0 | 0 | 0 | 0.00716146 | 0 | 0 | 0 | 0 | 0 |
| k__Bacteria;p__Actinobacteria;c__Actinobacteria;o__Actinomycetales;f__Nocardioidaceae;g__Aeromicrobium | | 0 | 0 | 0 | 0 | 0 | 0 | 0 | 0 | 0 | 0 | 0 | 0 | 0 | 0 | 0 |
| k__Bacteria;p__Actinobacteria;c__Actinobacteria;o__Actinomycetales;f__Nocardioidaceae;g__Friedmanniella | | 0 | 0 | 0 | 0 | 0 | 0 | 0 | 0 | 0 | 0 | 0 | 0 | 0 | 0 | 0 |
| k__Bacteria;p__Actinobacteria;c__Actinobacteria;o__Actinomycetales;f__Nocardioidaceae;g__Nocardioides | | 0 | 0 | 0 | 0 | 0 | 0 | 0 | 0 | 0 | 0 | 0 | 0 | 0 | 0 | 0 |
| k__Bacteria;p__Actinobacteria;c__Actinobacteria;o__Actinomycetales;f__Nocardioidaceae;g__Propionicimonas | | 0 | 0 | 0 | 0 | 0 | 0 | 0 | 0.00015132 | 0 | 0 | 0 | 0 | 0 | 0 | 0 |
| k__Bacteria;p__Actinobacteria;c__Actinobacteria;o__Actinomycetales;f__Promicromonosporaceae;g__Promicromonospora | | 0 | 0 | 0 | 0 | 0 | 0 | 0 | 0 | 0 | 0 | 0 | 0 | 0 | 0 | 0 |
| k__Bacteria;p__Actinobacteria;c__Actinobacteria;o__Actinomycetales;f__Propionibacteriaceae;g__ | | 0 | 0 | 0 | 0 | 0 | 0 | 0 | 0 | 0 | 0 | 0 | 0 | 0 | 0 | 0 |
| k__Bacteria;p__Actinobacteria;c__Actinobacteria;o__Actinomycetales;f__Propionibacteriaceae;g__Propionibacterium | | 0.00095217 | 0.00142985 | 0.00034061 | 0.00035685 | 0.00443049 | 0.00580363 | 0.01694482 | 0.0009079 | 0.0006096 | 0.02799479 | 0.0162037 | 0.00020669 | 6.92E-05 | 9.84E-05 | 0.00027525 |
| k__Bacteria;p__Actinobacteria;c__Actinobacteria;o__Actinomycetales;f__Pseudonocardiaceae;g__Pseudonocardia | | 0 | 0 | 0 | 0 | 0 | 0 | 0 | 0 | 0 | 0 | 0 | 0 | 0 | 0 | 0 |
| k__Bacteria;p__Actinobacteria;c__Actinobacteria;o__Actinomycetales;f__Pseudonocardiaceae;g__Saccharopolyspora | | 0 | 0 | 0 | 0 | 0 | 0 | 0 | 0 | 0 | 0 | 0 | 0 | 0 | 0 | 0 |
| k__Bacteria;p__Actinobacteria;c__Actinobacteria;o__Actinomycetales;f__Sanguibacteraceae;g__Sanguibacter | | 0 | 0 | 0 | 0 | 0 | 0 | 0 | 0 | 0 | 0 | 0 | 0 | 1.73E-05 | 0 | 0 |
| k__Bacteria;p__Actinobacteria;c__Actinobacteria;o__Actinomycetales;f__Sporichthyaceae;g__ | | 0 | 0.0002681 | 0 | 0 | 0 | 0 | 0 | 0 | 0 | 0 | 0 | 0 | 0 | 0 | 0 |
| k__Bacteria;p__Actinobacteria;c__Actinobacteria;o__Actinomycetales;f__Streptomycetaceae;g__ | | 0 | 0 | 0 | 0 | 0 | 0 | 0 | 0 | 0 | 0 | 0 | 0 | 0 | 0 | 0 |
| k__Bacteria;p__Actinobacteria;c__Actinobacteria;o__Actinomycetales;f__Williamsiaceae;g__Williamsia | | 0 | 0 | 0 | 0 | 0 | 0 | 0 | 0 | 0 | 0 | 0 | 0 | 0 | 0 | 0 |
| k__Bacteria;p__Actinobacteria;c__Actinobacteria;o__Actinomycetales;f__Yaniellaceae;g__Auritibacter | | 0.00017312 | 0 | 0 | 0 | 0 | 0 | 0 | 0 | 0 | 0 | 0 | 0 | 0 | 0 | 0 |
| k__Bacteria;p__Actinobacteria;c__Actinobacteria;o__Bifidobacteriales;f__Bifidobacteriaceae;g__ | | 0 | 0 | 0 | 0 | 0 | 0 | 0 | 0 | 0 | 0 | 0 | 0 | 0 | 0 | 0 |
| k__Bacteria;p__Actinobacteria;c__Actinobacteria;o__Bifidobacteriales;f__Bifidobacteriaceae;g__Alloscardovia | | 0 | 0 | 0 | 0 | 0 | 0 | 0 | 0 | 0 | 0 | 0 | 0 | 0 | 0 | 0 |
| k__Bacteria;p__Actinobacteria;c__Actinobacteria;o__Bifidobacteriales;f__Bifidobacteriaceae;g__Bifidobacterium | | 0 | 0 | 0 | 0 | 0 | 0 | 0 | 0 | 0 | 0 | 0 | 0 | 0 | 1.64E-05 | 0 |
| k__Bacteria;p__Actinobacteria;c__Actinobacteria;o__Bifidobacteriales;f__Bifidobacteriaceae;g__Gardnerella | | 0 | 0 | 0 | 0 | 0 | 0 | 0 | 0 | 0 | 0 | 0 | 0 | 0 | 0 | 0 |
| k__Bacteria;p__Actinobacteria;c__Actinobacteria;o__Bifidobacteriales;f__Bifidobacteriaceae;g__Scardovia | | 0 | 0 | 0 | 0 | 0 | 0 | 0 | 0 | 0 | 0 | 0 | 0 | 0 | 0 | 0 |
| k__Bacteria;p__Actinobacteria;c__Actinobacteria;o__WCHB1-81;f__At425_EubF1;g__ | | 0 | 0 | 0 | 0 | 0 | 0 | 0 | 0 | 0 | 0 | 0 | 0 | 0 | 0 | 0 |
| k__Bacteria;p__Actinobacteria;c__Coriobacteriia;o__Coriobacteriales;f__Coriobacteriaceae;g__Atopobium | | 0 | 0 | 0 | 0 | 0 | 0 | 0 | 0 | 0 | 0 | 0 | 0 | 0 | 0 | 0 |
| k__Bacteria;p__Actinobacteria;c__Rubrobacteria;o__Rubrobacterales;f__Rubrobacteraceae;g__Rubrobacter | | 0 | 0.00098302 | 0 | 0 | 0 | 0 | 0 | 0 | 0 | 0 | 0 | 0 | 0 | 0 | 0 |
| k__Bacteria;p__Actinobacteria;c__Thermoleophilia;o__Gaiellales;f__Gaiellaceae;g__ | | 0 | 0 | 0.00022039 | 0 | 0 | 0 | 0 | 0 | 0 | 0.00186012 | 0 | 0 | 0 | 0 | 0 |
| k__Bacteria;p__Actinobacteria;c__Thermoleophilia;o__Solirubrobacterales;f__Conexibacteraceae;g__ | | 0 | 0 | 0 | 0 | 0 | 0.0003206 | 0 | 0 | 0 | 0 | 0 | 0 | 0 | 0 | 0 |
| k__Bacteria;p__Actinobacteria;c__Thermoleophilia;o__Solirubrobacterales;f__Patulibacteraceae;g__ | | 0 | 0 | 0 | 0 | 0 | 0 | 0 | 0 | 0 | 0 | 0 | 0 | 0 | 0 | 0 |
| k__Bacteria;p__Actinobacteria;c__Thermoleophilia;o__Solirubrobacterales;f__Solirubrobacteraceae;g__ | | 0 | 0 | 0 | 0 | 0 | 0.00014797 | 0 | 0 | 0 | 0 | 0 | 0 | 0 | 0 | 0 |
| k__Bacteria;p__Actinobacteria;c__Thermoleophilia;o__Solirubrobacterales;f__Solirubrobacteraceae;g__Solirubrobacter | | 0 | 0 | 0 | 0 | 0 | 0 | 0 | 0 | 0 | 0 | 0 | 0 | 0 | 0 | 0 |
| k__Bacteria;p__Bacteroidetes;c__Bacteroidia;o__Bacteroidales;f__;g__ | | 0 | 0 | 0 | 0 | 0 | 0 | 0 | 0 | 0 | 0 | 0 | 0 | 0 | 0 | 0 |
| k__Bacteria;p__Bacteroidetes;c__Bacteroidia;o__Bacteroidales;f__Bacteroidaceae;g__Bacteroides | | 0 | 0.00016384 | 0 | 0 | 0 | 0 | 0 | 1.68E-05 | 0 | 0 | 0 | 0 | 0 | 0 | 0 |
| k__Bacteria;p__Bacteroidetes;c__Bacteroidia;o__Bacteroidales;f__Porphyromonadaceae;g__ | | 0 | 0 | 0 | 0 | 0 | 0 | 0 | 0 | 0.00041168 | 0 | 0 | 0 | 0 | 0 | 0 |
| k__Bacteria;p__Bacteroidetes;c__Bacteroidia;o__Bacteroidales;f__Porphyromonadaceae;g__Paludibacter | | 0 | 0 | 0 | 0 | 0 | 0 | 0 | 0 | 0 | 0 | 0 | 0 | 0 | 1.64E-05 | 0 |
| k__Bacteria;p__Bacteroidetes;c__Bacteroidia;o__Bacteroidales;f__Porphyromonadaceae;g__Parabacteroides | | 0 | 0 | 0 | 0 | 0 | 0 | 0 | 0 | 0 | 0 | 0 | 0 | 0 | 0 | 0 |
| k__Bacteria;p__Bacteroidetes;c__Bacteroidia;o__Bacteroidales;f__Porphyromonadaceae;g__Porphyromonas | | 0.00020198 | 0 | 0.00020036 | 0.00027755 | 0.00397478 | 0 | 0.00545484 | 0.00015972 | 0.0001425 | 0.00325521 | 0.00610269 | 0 | 1.73E-05 | 0 | 0.00012042 |
| k__Bacteria;p__Bacteroidetes;c__Bacteroidia;o__Bacteroidales;f__Prevotellaceae;g__Prevotella | | 0.00014427 | 0.00047662 | 0 | 5.95E-05 | 0 | 0 | 0.00582623 | 0 | 0.00028501 | 0 | 0 | 0 | 0 | 0 | 0.00025805 |
| k__Bacteria;p__Bacteroidetes;c__Bacteroidia;o__Bacteroidales;f__Rikenellaceae;g__ | | 0 | 0 | 0 | 0 | 0 | 0 | 0 | 0 | 0 | 0 | 0 | 0 | 0 | 0 | 0 |
| k__Bacteria;p__Bacteroidetes;c__Bacteroidia;o__Bacteroidales;f__SB-1;g__ | | 0 | 0 | 0 | 0 | 0 | 0 | 0 | 0 | 0 | 0 | 0 | 0 | 0 | 0 | 0 |
| k__Bacteria;p__Bacteroidetes;c__Bacteroidia;o__Bacteroidales;f__[Odoribacteraceae];g__Butyricimonas | | 0 | 0 | 0 | 0 | 0 | 0 | 0 | 0 | 0 | 0 | 0 | 0 | 0 | 0 | 0 |
| k__Bacteria;p__Bacteroidetes;c__Bacteroidia;o__Bacteroidales;f__[Odoribacteraceae];g__Odoribacter | | 0 | 0 | 0 | 0 | 0 | 0 | 0 | 0 | 0 | 0 | 0 | 0 | 0 | 0 | 0 |
| k__Bacteria;p__Bacteroidetes;c__Bacteroidia;o__Bacteroidales;f__[Paraprevotellaceae];g__[Prevotella] | | 0 | 0 | 6.01E-05 | 0 | 0 | 0.00018907 | 0 | 0 | 0.00030084 | 0 | 0 | 0 | 0 | 0 | 0 |
| k__Bacteria;p__Bacteroidetes;c__Cytophagia;o__Cytophagales;f__Cyclobacteriaceae;g__ | | 0 | 0 | 0 | 0 | 0 | 0 | 0 | 0 | 0 | 0 | 0 | 0 | 0 | 0 | 0 |
| k__Bacteria;p__Bacteroidetes;c__Cytophagia;o__Cytophagales;f__Cytophagaceae;g__ | | 0 | 0 | 0.00016029 | 0 | 0 | 0 | 0 | 0 | 0 | 0 | 0 | 0 | 0 | 0 | 0 |
| k__Bacteria;p__Bacteroidetes;c__Cytophagia;o__Cytophagales;f__Cytophagaceae;g__Adhaeribacter | | 0 | 8.94E-05 | 0 | 0 | 0 | 0 | 0 | 0 | 0 | 0 | 0 | 0 | 0 | 0 | 0 |
| k__Bacteria;p__Bacteroidetes;c__Cytophagia;o__Cytophagales;f__Cytophagaceae;g__Cytophaga | | 0 | 0 | 0 | 0 | 0 | 0 | 0 | 0 | 0 | 0 | 0 | 4.70E-05 | 0 | 0 | 0 |
| k__Bacteria;p__Bacteroidetes;c__Cytophagia;o__Cytophagales;f__Cytophagaceae;g__Hymenobacter | | 0 | 0 | 0 | 0 | 0 | 0.00057543 | 0 | 0 | 0 | 0 | 0 | 0 | 0 | 0 | 1.72E-05 |
| k__Bacteria;p__Bacteroidetes;c__Cytophagia;o__Cytophagales;f__Cytophagaceae;g__Leadbetterella | | 0 | 0.0005064 | 0 | 0 | 0 | 0 | 0 | 0 | 0 | 0 | 0 | 0 | 0 | 0 | 0 |
| k__Bacteria;p__Bacteroidetes;c__Cytophagia;o__Cytophagales;f__Cytophagaceae;g__Spirosoma | | 0 | 0 | 0 | 0 | 0 | 0 | 0 | 0 | 0.00031668 | 0 | 0 | 0 | 0 | 0 | 0 |
| k__Bacteria;p__Bacteroidetes;c__Cytophagia;o__Cytophagales;f__Flammeovirgaceae;g__ | | 0.0007502 | 0.00020852 | 0.00026046 | 0 | 0.00040507 | 0.00194002 | 0 | 0.00021016 | 0 | 0 | 0 | 0.00049793 | 0.00032874 | 0.00018036 | 0.00015483 |
| k__Bacteria;p__Bacteroidetes;c__Cytophagia;o__Cytophagales;f__Flammeovirgaceae;g__JTB248 | | 0 | 0.00013405 | 0 | 0 | 0 | 0 | 0 | 0 | 0 | 0 | 0 | 0 | 0 | 0 | 0 |
| k__Bacteria;p__Bacteroidetes;c__Cytophagia;o__Cytophagales;f__Flammeovirgaceae;g__Roseivirga | | 0.00030296 | 0 | 0.00022039 | 0 | 0 | 0 | 0 | 0 | 0 | 0 | 0 | 0 | 0 | 1.64E-05 | 0 |
| k__Bacteria;p__Bacteroidetes;c__Cytophagia;o__Cytophagales;f__[Amoebophilaceae];g__SC3-56 | | 0 | 0 | 0 | 0 | 0 | 0 | 0 | 0 | 0 | 0 | 0 | 0 | 0 | 0 | 0 |
| k__Bacteria;p__Bacteroidetes;c__Flavobacteriia;o__Flavobacteriales;f__;g__ | | 0.00461661 | 0.00460232 | 0.00078139 | 0 | 0.00159498 | 0.00735729 | 0 | 0.00033626 | 0.00296884 | 9.30E-05 | 0 | 0.00234872 | 0.00371998 | 0.0025414 | 0.00202997 |
| k__Bacteria;p__Bacteroidetes;c__Flavobacteriia;o__Flavobacteriales;f__Cryomorphaceae;g__ | | 0.02911347 | 0.02158177 | 0.00534952 | 0 | 0 | 0.00596804 | 0.0048049 | 0 | 0.00022959 | 0 | 0.00505051 | 0.00185079 | 0.00119385 | 0.00075422 | 0.00116981 |
| k__Bacteria;p__Bacteroidetes;c__Flavobacteriia;o__Flavobacteriales;f__Cryomorphaceae;g__Crocinitomix | | 0 | 0 | 0 | 0 | 0 | 0 | 0 | 0 | 0.00043543 | 0 | 0 | 0.0005543 | 0.00019032 | 0.00018036 | 0.00017203 |
| k__Bacteria;p__Bacteroidetes;c__Flavobacteriia;o__Flavobacteriales;f__Cryomorphaceae;g__Fluviicola | | 0.00137055 | 0.00150432 | 0.00096171 | 0 | 0.00081015 | 0.0037403 | 0 | 0.00016813 | 0.00145671 | 0 | 0 | 0.00356066 | 0.00325282 | 0.00500082 | 0.00345783 |
| k__Bacteria;p__Bacteroidetes;c__Flavobacteriia;o__Flavobacteriales;f__Flavobacteriaceae;g__ | | 0.02107769 | 0.01732201 | 0.00875558 | 0.00551128 | 0.01405099 | 0.05602229 | 0.0316845 | 0.03215475 | 0.02127272 | 0.02929688 | 0.00715488 | 0.05706448 | 0.03730362 | 0.05300869 | 0.03881023 |
| k__Bacteria;p__Bacteroidetes;c__Flavobacteriia;o__Flavobacteriales;f__Flavobacteriaceae;g__Algibacter | | 0 | 0 | 0 | 0 | 0 | 0 | 0 | 0 | 0 | 0 | 0 | 0 | 0 | 0 | 0 |
| k__Bacteria;p__Bacteroidetes;c__Flavobacteriia;o__Flavobacteriales;f__Flavobacteriaceae;g__Aquimarina | | 0 | 0 | 0 | 0 | 0 | 0 | 0 | 0 | 0 | 0 | 0 | 0 | 0 | 0 | 0 |
| k__Bacteria;p__Bacteroidetes;c__Flavobacteriia;o__Flavobacteriales;f__Flavobacteriaceae;g__Bizionia | | 0 | 0 | 0 | 0 | 0 | 0 | 0 | 0 | 0 | 0 | 0 | 0 | 0 | 1.64E-05 | 0 |
| k__Bacteria;p__Bacteroidetes;c__Flavobacteriia;o__Flavobacteriales;f__Flavobacteriaceae;g__Capnocytophaga | | 0 | 0 | 0 | 0 | 0 | 0 | 0.00162485 | 0 | 0 | 0 | 0 | 0 | 0 | 0 | 8.60E-05 |
| k__Bacteria;p__Bacteroidetes;c__Flavobacteriia;o__Flavobacteriales;f__Flavobacteriaceae;g__Cellulophaga | | 0 | 0 | 0 | 0 | 0 | 0 | 0 | 0 | 0 | 0 | 0 | 0 | 0 | 0 | 0 |
| k__Bacteria;p__Bacteroidetes;c__Flavobacteriia;o__Flavobacteriales;f__Flavobacteriaceae;g__Coccinimonas | | 0 | 0 | 0 | 0 | 0 | 0 | 0 | 0 | 0 | 0 | 0 | 0 | 0 | 1.64E-05 | 0 |
| k__Bacteria;p__Bacteroidetes;c__Flavobacteriia;o__Flavobacteriales;f__Flavobacteriaceae;g__Flavobacterium | | 0.00952175 | 0.01465594 | 0.00446795 | 0.00138773 | 0.00764576 | 0.01992634 | 0.00680114 | 0.00474966 | 0.00843942 | 0.01032366 | 0.07744108 | 0.01153691 | 0.01382449 | 0.01421545 | 0.01278192 |
| k__Bacteria;p__Bacteroidetes;c__Flavobacteriia;o__Flavobacteriales;f__Flavobacteriaceae;g__Formosa | | 0 | 0 | 0 | 0 | 0 | 0 | 0 | 0 | 0 | 0 | 0 | 1.88E-05 | 0 | 0 | 0 |
| k__Bacteria;p__Bacteroidetes;c__Flavobacteriia;o__Flavobacteriales;f__Flavobacteriaceae;g__Gaetbulibacter | | 0 | 0 | 0 | 0 | 0 | 0 | 0 | 0 | 0 | 0 | 0 | 0 | 0 | 0 | 1.72E-05 |
| k__Bacteria;p__Bacteroidetes;c__Flavobacteriia;o__Flavobacteriales;f__Flavobacteriaceae;g__Gillisia | | 0 | 0 | 0 | 0 | 0 | 0.00097823 | 0.00280866 | 0.00083224 | 0.00023751 | 0 | 0 | 0 | 0.00015572 | 0.00021315 | 3.44E-05 |
| k__Bacteria;p__Bacteroidetes;c__Flavobacteriia;o__Flavobacteriales;f__Flavobacteriaceae;g__Gilvibacter | | 0 | 0 | 0 | 0 | 0 | 0 | 0 | 0 | 0 | 0 | 0 | 0 | 0 | 0 | 0 |
| k__Bacteria;p__Bacteroidetes;c__Flavobacteriia;o__Flavobacteriales;f__Flavobacteriaceae;g__Gramella | | 0 | 0 | 0 | 0 | 0 | 0 | 0 | 0 | 0 | 0 | 0 | 0 | 3.46E-05 | 0 | 0 |
| k__Bacteria;p__Bacteroidetes;c__Flavobacteriia;o__Flavobacteriales;f__Flavobacteriaceae;g__Kordia | | 0 | 0 | 0 | 0 | 0 | 0.0003206 | 0 | 0 | 0 | 0 | 0 | 0 | 0 | 0 | 0 |
| k__Bacteria;p__Bacteroidetes;c__Flavobacteriia;o__Flavobacteriales;f__Flavobacteriaceae;g__Krokinobacter | | 0 | 0 | 0 | 0.00051544 | 0 | 0.00037814 | 0 | 0 | 0.00054627 | 0 | 0 | 0.00012213 | 0.00025953 | 0.00013117 | 0 |

| Taxon | | 1180 | 1182 | 1183 | 1203 | 1211 | 1242 | 6475 | 6476 | 6477 | 1220 | 1221 | 1248 | 2032 | 2033 | 2034 |
|---|---|---|---|---|---|---|---|---|---|---|---|---|---|---|---|---|
| | Copepod genus | Acartia | Acartia | Acartia | Acartia | Acartia | Acartia | Acartia | Acartia | Acartia | Acartia | Acartia | Acartia | Acartia | Acartia | Acartia |
| | Type of sample | FullGut | FullGut | FullGut | FullGut | FullGut | FullGut | FullGut | FullGut | FullGut | Starved | Starved | Starved | Starved | Starved | Starved |
| k__Bacteria;p__Bacteroidetes;c__Flavobacteriia;o__Flavobacteriales;f__Flavobacteriaceae;g__Lacinutrix | | 2.89E-05 | 2.98E-05 | 0 | 0 | 0 | 2.47E-05 | 0 | 0 | 3.96E-05 | 0 | 0 | 7.52E-05 | 3.46E-05 | 8.20E-05 | 5.16E-05 |
| k__Bacteria;p__Bacteroidetes;c__Flavobacteriia;o__Flavobacteriales;f__Flavobacteriaceae;g__Leeuwenhoekiella | | 0 | 0 | 0 | 0 | 0 | 0.00262232 | 0 | 0.00014291 | 0 | 0 | 0 | 0 | 0.00010381 | 0 | 8.60E-05 |
| k__Bacteria;p__Bacteroidetes;c__Flavobacteriia;o__Flavobacteriales;f__Flavobacteriaceae;g__Lutimonas | | 0 | 0 | 0 | 0 | 0 | 0 | 0 | 0 | 0 | 0 | 0 | 0 | 0 | 0 | 0 |
| k__Bacteria;p__Bacteroidetes;c__Flavobacteriia;o__Flavobacteriales;f__Flavobacteriaceae;g__Maribacter | | 0 | 0 | 0 | 0 | 0 | 0.00010687 | 0 | 0 | 0 | 0 | 0 | 0 | 0 | 0 | 0 |
| k__Bacteria;p__Bacteroidetes;c__Flavobacteriia;o__Flavobacteriales;f__Flavobacteriaceae;g__Mesonia | | 0 | 0 | 0 | 0 | 0 | 0 | 0 | 0 | 0.00013459 | 0 | 0 | 0 | 3.46E-05 | 0.00014757 | 0.00012042 |
| k__Bacteria;p__Bacteroidetes;c__Flavobacteriia;o__Flavobacteriales;f__Flavobacteriaceae;g__Olleya | | 0 | 1.49E-05 | 0 | 0 | 0 | 0 | 0 | 0.00096674 | 0.00084711 | 0 | 0 | 0.00024427 | 0.0007613 | 0.00177078 | 0.00103219 |
| k__Bacteria;p__Bacteroidetes;c__Flavobacteriia;o__Flavobacteriales;f__Flavobacteriaceae;g__Persicivirga | | 0 | 0 | 0 | 0 | 0 | 0 | 0 | 0 | 0.00018209 | 0 | 0 | 0 | 0.00027684 | 0.00057386 | 0.00030966 |
| k__Bacteria;p__Bacteroidetes;c__Flavobacteriia;o__Flavobacteriales;f__Flavobacteriaceae;g__Polaribacter | | 0.01269566 | 0.01508788 | 0.00462824 | 0.00156616 | 0.00045571 | 0.00895206 | 0.00099812 | 0.00042032 | 0.00186047 | 0.0063244 | 0.00084175 | 0.00289362 | 0.00115925 | 0.00059026 | 0.00113541 |
| k__Bacteria;p__Bacteroidetes;c__Flavobacteriia;o__Flavobacteriales;f__Flavobacteriaceae;g__Pontirhabdus | | 0 | 0 | 0 | 0 | 0 | 0 | 0 | 0 | 0 | 0 | 0 | 0 | 0 | 0 | 0 |
| k__Bacteria;p__Bacteroidetes;c__Flavobacteriia;o__Flavobacteriales;f__Flavobacteriaceae;g__Psychroserpens | | 4.33E-05 | 0 | 0 | 1.98E-05 | 0 | 0.00019729 | 2.32E-05 | 0.00015132 | 0 | 0 | 0 | 9.39E-06 | 0.00019032 | 0.00021315 | 0.00030966 |
| k__Bacteria;p__Bacteroidetes;c__Flavobacteriia;o__Flavobacteriales;f__Flavobacteriaceae;g__Robiginitalea | | 0 | 0 | 0 | 0 | 0 | 0 | 0 | 0 | 0 | 0 | 0 | 0 | 0 | 0 | 0 |
| k__Bacteria;p__Bacteroidetes;c__Flavobacteriia;o__Flavobacteriales;f__Flavobacteriaceae;g__Sediminicola | | 0.05600519 | 0.06717307 | 0.00991765 | 0 | 0 | 0.02226095 | 0 | 0.04695013 | 0.07503642 | 0 | 0 | 0.0329948 | 0.01652363 | 0.01552714 | 0.01945673 |
| k__Bacteria;p__Bacteroidetes;c__Flavobacteriia;o__Flavobacteriales;f__Flavobacteriaceae;g__Tenacibaculum | | 0.00214961 | 0.00248734 | 0.00222396 | 0 | 0 | 0.00591872 | 0 | 0.00352231 | 0.00774273 | 9.30E-05 | 0 | 0.00639791 | 0.00342584 | 0.00259059 | 0.00261487 |
| k__Bacteria;p__Bacteroidetes;c__Flavobacteriia;o__Flavobacteriales;f__Flavobacteriaceae;g__Ulvibacter | | 0 | 0 | 0.00016029 | 0 | 0 | 0.00055899 | 0 | 8.41E-06 | 0 | 0 | 0 | 0.00015032 | 6.92E-05 | 8.20E-05 | 0.00013762 |
| k__Bacteria;p__Bacteroidetes;c__Flavobacteriia;o__Flavobacteriales;f__Flavobacteriaceae;g__Winogradskyella | | 0 | 0 | 0 | 0 | 0 | 0 | 0 | 0 | 0 | 0 | 0 | 0.00011274 | 0.00010381 | 0.00011477 | 3.44E-05 |
| k__Bacteria;p__Bacteroidetes;c__Flavobacteriia;o__Flavobacteriales;f__NS9;g__ | | 0.00090889 | 0.00189157 | 0.00034061 | 0 | 0 | 0.00114264 | 0 | 7.57E-05 | 0 | 0 | 0 | 3.76E-05 | 0.00091702 | 0.00027873 | 0.00075694 |
| k__Bacteria;p__Bacteroidetes;c__Flavobacteriia;o__Flavobacteriales;f__[Weeksellaceae];g__ | | 0 | 0 | 6.01E-05 | 0 | 0.0001519 | 8.22E-06 | 0 | 0.00010928 | 0 | 0.00409226 | 0 | 0 | 0 | 0 | 0 |
| k__Bacteria;p__Bacteroidetes;c__Flavobacteriia;o__Flavobacteriales;f__[Weeksellaceae];g__Chryseobacterium | | 7.21E-05 | 0.00011915 | 0 | 0 | 0.00065824 | 0 | 0.00083564 | 0 | 0 | 0 | 0.01409933 | 0 | 0 | 0 | 0 |
| k__Bacteria;p__Bacteroidetes;c__Flavobacteriia;o__Flavobacteriales;f__[Weeksellaceae];g__Cloacibacterium | | 0 | 0.00198094 | 0 | 0 | 0 | 0 | 0.00197303 | 5.04E-05 | 0 | 0 | 0 | 2.82E-05 | 0 | 0 | 0 |
| k__Bacteria;p__Bacteroidetes;c__Flavobacteriia;o__Flavobacteriales;f__[Weeksellaceae];g__Weeksella | | 0 | 0 | 0 | 0 | 0 | 0 | 0 | 0 | 0 | 0 | 0 | 0 | 0 | 0 | 0 |
| k__Bacteria;p__Bacteroidetes;c__Sphingobacteriia;o__Sphingobacteriales;f__;g__ | | 0 | 0 | 0 | 0 | 0 | 0.00255656 | 0.00062673 | 0.00044554 | 0.00059377 | 0 | 0 | 0.00333518 | 0.00531179 | 0.00403345 | 0.00359545 |
| k__Bacteria;p__Bacteroidetes;c__Sphingobacteriia;o__Sphingobacteriales;f__NS11-12;g__ | | 0.0003751 | 0.00074471 | 0 | 9.91E-05 | 0 | 0.00030416 | 0 | 0 | 0 | 0 | 0 | 1.88E-05 | 0 | 0 | 0 |
| k__Bacteria;p__Bacteroidetes;c__Sphingobacteriia;o__Sphingobacteriales;f__Sphingobacteriaceae;g__Pedobacter | | 0.0001587 | 0.0007894 | 0 | 0 | 0 | 0 | 0 | 0 | 0.00057002 | 0 | 0 | 1.88E-05 | 0 | 0 | 0 |
| k__Bacteria;p__Bacteroidetes;c__Sphingobacteriia;o__Sphingobacteriales;f__Sphingobacteriaceae;g__Sphingobacterium | | 0 | 0 | 0 | 0 | 0 | 0 | 0 | 0 | 0 | 0 | 0 | 0 | 0 | 0 | 0 |
| k__Bacteria;p__Bacteroidetes;c__VC2_1_Bac22;o__;f__;g__ | | 0 | 0 | 0 | 0 | 0 | 0 | 0 | 0 | 0 | 0 | 0 | 0 | 0 | 0 | 0 |
| k__Bacteria;p__Bacteroidetes;c__[Rhodothermi];o__[Rhodothermales];f__[Balneolaceae];g__Balneola | | 0 | 0 | 0 | 0 | 0 | 0 | 0 | 0 | 0 | 0 | 0 | 2.82E-05 | 1.73E-05 | 0 | 3.44E-05 |
| k__Bacteria;p__Bacteroidetes;c__[Saprospirae];o__[Saprospirales];f__;g__ | | 0 | 0 | 0 | 0 | 0 | 0 | 0 | 0 | 0 | 0 | 0 | 0 | 0 | 0.00013117 | 0 |
| k__Bacteria;p__Bacteroidetes;c__[Saprospirae];o__[Saprospirales];f__Chitinophagaceae;g__ | | 0 | 0 | 0 | 9.91E-05 | 0 | 0 | 0 | 0.00018494 | 0 | 0.00139509 | 0 | 4.70E-05 | 0 | 1.64E-05 | 0 |
| k__Bacteria;p__Bacteroidetes;c__[Saprospirae];o__[Saprospirales];f__Chitinophagaceae;g__Chitinophaga | | 0 | 0 | 0 | 0 | 0 | 0 | 0 | 0 | 0 | 0 | 0.003367 | 0 | 0 | 0 | 0 |
| k__Bacteria;p__Bacteroidetes;c__[Saprospirae];o__[Saprospirales];f__Chitinophagaceae;g__Niabella | | 0 | 0 | 0 | 0 | 0 | 0 | 0 | 0.0001345 | 0 | 0 | 0 | 0 | 0 | 0 | 0 |
| k__Bacteria;p__Bacteroidetes;c__[Saprospirae];o__[Saprospirales];f__Chitinophagaceae;g__Sediminibacterium | | 0 | 0 | 0 | 0 | 0 | 0 | 0 | 0 | 0.00018209 | 0 | 0 | 9.39E-06 | 0 | 0 | 0 |
| k__Bacteria;p__Bacteroidetes;c__[Saprospirae];o__[Saprospirales];f__Saprospiraceae;g__ | | 7.21E-05 | 0 | 0 | 0.00105071 | 0 | 0 | 0 | 0 | 0 | 0 | 0 | 0 | 3.46E-05 | 0 | 0 |
| k__Bacteria;p__Bacteroidetes;c__[Saprospirae];o__[Saprospirales];f__Saprospiraceae;g__Lewinella | | 0 | 0 | 0 | 0 | 0 | 0 | 0 | 0 | 0 | 0 | 0 | 0 | 0 | 0 | 6.88E-05 |
| k__Bacteria;p__Bacteroidetes;c__[Saprospirae];o__[Saprospirales];f__Saprospiraceae;g__Saprospira | | 0 | 0 | 0.00074132 | 0.01542365 | 0 | 0 | 0 | 0 | 0 | 0 | 0 | 0 | 0 | 0 | 0.00015483 |
| k__Bacteria;p__Chlamydiae;c__Chlamydiia;o__Chlamydiales;f__;g__ | | 0 | 0 | 0 | 0 | 0 | 0 | 0 | 0 | 0 | 0 | 0 | 0 | 0 | 0 | 0 |
| k__Bacteria;p__Chloroflexi;c__Anaerolineae;o__Caldilineales;f__Caldilineaceae;g__ | | 0 | 0 | 0 | 0 | 0 | 0 | 0 | 0 | 0 | 0 | 0 | 0 | 0 | 0 | 0 |
| k__Bacteria;p__Chloroflexi;c__Anaerolineae;o__GCA004;f__;g__ | | 0 | 0 | 0 | 0 | 0 | 0 | 0 | 0 | 0 | 0 | 0 | 0 | 0 | 0 | 0 |
| k__Bacteria;p__Chloroflexi;c__Chloroflexi;o__[Roseiflexales];f__;g__ | | 0 | 0 | 0 | 0 | 0 | 0.00020551 | 0 | 0 | 0 | 0 | 0 | 0 | 0 | 0 | 0 |
| k__Bacteria;p__Chloroflexi;c__Ellin6529;o__;f__;g__ | | 0 | 0 | 0 | 0 | 0 | 0 | 0 | 0 | 0 | 0 | 0 | 0 | 0 | 0 | 0 |
| k__Bacteria;p__Chloroflexi;c__Gitt-GS-136;o__;f__;g__ | | 0 | 0 | 0 | 0 | 0 | 0 | 0 | 0 | 0 | 0 | 0 | 0 | 0 | 0 | 0 |
| k__Bacteria;p__Chloroflexi;c__S085;o__;f__;g__ | | 0 | 0 | 0 | 0 | 0 | 0 | 0 | 0 | 0 | 0 | 0 | 0 | 0 | 0 | 0 |
| k__Bacteria;p__Chloroflexi;c__SAR202;o__;f__;g__ | | 0 | 0 | 0 | 0 | 0 | 0 | 0 | 0 | 0 | 0 | 0 | 0 | 0 | 0 | 0 |
| k__Bacteria;p__Chloroflexi;c__TK10;o__AKYG885;f__Dolo_23;g__ | | 0 | 0 | 0 | 0 | 0 | 0 | 0.0011606 | 0 | 0 | 0 | 0 | 0 | 0 | 0 | 0 |
| k__Bacteria;p__Cyanobacteria;c__4C0d-2;o__MLE1-12;f__;g__ | | 0 | 0 | 0 | 0 | 0.00075951 | 0 | 0.00176412 | 0 | 0 | 0 | 0 | 0 | 0 | 0 | 0 |
| k__Bacteria;p__Cyanobacteria;c__4C0d-2;o__YS2;f__;g__ | | 0 | 0 | 0 | 0 | 0 | 0 | 0 | 0 | 0 | 0 | 0 | 0 | 0 | 0 | 0 |
| k__Bacteria;p__Cyanobacteria;c__Chloroplast;o__;f__;g__ | | 0 | 0 | 0 | 0 | 0 | 0 | 0 | 0 | 0 | 0 | 0 | 0 | 5.19E-05 | 0 | 3.44E-05 |
| k__Bacteria;p__Cyanobacteria;c__Chloroplast;o__Cercozoa;f__;g__ | | 0 | 0 | 0 | 0 | 0 | 0 | 0 | 0 | 0 | 0 | 0 | 0 | 0 | 0 | 0 |
| k__Bacteria;p__Cyanobacteria;c__Chloroplast;o__Chlorophyta;f__;g__ | | 0.00018755 | 0.00022341 | 0 | 0 | 0 | 0.00266342 | 0.00106776 | 0 | 7.92E-06 | 0 | 0 | 0.00061067 | 0.00690359 | 6.56E-05 | 0.00505772 |
| k__Bacteria;p__Cyanobacteria;c__Chloroplast;o__Chlorophyta;f__Mamiellaceae;g__ | | 0 | 0 | 0 | 0 | 0 | 0.01109759 | 0 | 0 | 0.00029293 | 0 | 0 | 0.00279967 | 0.00920479 | 0.00060666 | 0.00626193 |
| k__Bacteria;p__Cyanobacteria;c__Chloroplast;o__Chlorophyta;f__Mamiellaceae;g__Micromonas | | 0 | 0 | 0 | 0 | 0 | 0 | 0 | 0 | 0 | 0 | 0 | 0 | 0 | 0 | 6.88E-05 |
| k__Bacteria;p__Cyanobacteria;c__Chloroplast;o__Chlorophyta;f__Trebouxiophyceae;g__ | | 0 | 0 | 0 | 0 | 0 | 0 | 0 | 0 | 0 | 0 | 0 | 0 | 0 | 0 | 0 |
| k__Bacteria;p__Cyanobacteria;c__Chloroplast;o__Cryptophyta;f__;g__ | | 0 | 0 | 0.00026046 | 0 | 0 | 0.00983987 | 0 | 0 | 0 | 0.00362723 | 0 | 0 | 1.73E-05 | 3.28E-05 | 6.88E-05 |
| k__Bacteria;p__Cyanobacteria;c__Chloroplast;o__Haptophyceae;f__;g__ | | 0.00030296 | 0 | 0.00046082 | 0 | 0 | 0.00571321 | 0 | 0 | 0 | 0 | 0 | 9.39E-06 | 0 | 0 | 0.00017203 |
| k__Bacteria;p__Cyanobacteria;c__Chloroplast;o__Rhodophyta;f__;g__ | | 0 | 0 | 0 | 0 | 0 | 0 | 0 | 0 | 0 | 0 | 0 | 0 | 0 | 0 | 0 |
| k__Bacteria;p__Cyanobacteria;c__Chloroplast;o__Stramenopiles;f__;g__ | | 0.05705836 | 0.10403634 | 0.01582817 | 1.98E-05 | 0 | 0.00397869 | 0.00359787 | 0.00031104 | 0.00035626 | 0 | 0 | 0.00022548 | 0.00043256 | 0.00014757 | 0.00092897 |
| k__Bacteria;p__Cyanobacteria;c__Chloroplast;o__Streptophyta;f__;g__ | | 0.00264012 | 5.96E-05 | 0.00012021 | 3.96E-05 | 0 | 0.00318953 | 0 | 0 | 0 | 0.0093936 | 0.01136364 | 0.01489088 | 0 | 0 | 0.00029245 |
| k__Bacteria;p__Cyanobacteria;c__Nostocophycideae;o__Nostocales;f__Nostocaceae;g__ | | 0 | 0 | 0 | 0 | 0 | 0 | 0 | 0 | 0 | 0 | 0 | 0 | 0 | 0 | 0 |
| k__Bacteria;p__Cyanobacteria;c__Nostocophycideae;o__Stigonematales;f__Rivulariaceae;g__Calothrix | | 0 | 0 | 0 | 5.95E-05 | 0 | 4.11E-05 | 0 | 0 | 0 | 0.00288318 | 0 | 2.82E-05 | 3.46E-05 | 0 | 1.72E-05 |
| k__Bacteria;p__Cyanobacteria;c__Oscillatoriophycideae;o__Chroococcales;f__Cyanobacteriaceae;g__ | | 0 | 0 | 0 | 0 | 0 | 0 | 0 | 0 | 0 | 0 | 0 | 0 | 0 | 0 | 0 |
| k__Bacteria;p__Cyanobacteria;c__Oscillatoriophycideae;o__Chroococcales;f__Microcystaceae;g__Microcystis | | 0 | 0 | 0 | 0 | 0 | 0 | 0 | 0.00010088 | 0 | 0 | 0 | 0 | 3.46E-05 | 0 | 5.16E-05 |
| k__Bacteria;p__Cyanobacteria;c__Oscillatoriophycideae;o__Chroococcales;f__Xenococcaceae;g__ | | 0.00047609 | 0.00020852 | 0.00024043 | 5.95E-05 | 0.00220259 | 0 | 2.32E-05 | 0 | 0 | 0.00530134 | 0.01515152 | 0 | 0 | 8.20E-05 | 1.72E-05 |
| k__Bacteria;p__Cyanobacteria;c__Synechococcophycideae;o__Pseudanabaenales;f__Pseudanabaenaceae;g__ | | 0 | 0 | 0 | 0 | 0 | 0 | 0 | 0 | 0 | 0 | 0 | 0 | 0 | 0 | 0 |
| k__Bacteria;p__Cyanobacteria;c__Synechococcophycideae;o__Pseudanabaenales;f__Pseudanabaenaceae;g__Pseudanabaena | | 0 | 0 | 0 | 0 | 0 | 0 | 0 | 0 | 0 | 0 | 0 | 0 | 0 | 0 | 0 |
| k__Bacteria;p__Cyanobacteria;c__Synechococcophycideae;o__Synechococcales;f__Synechococcaceae;g__Prochlorococcus | | 0 | 0 | 0 | 0 | 0 | 0 | 0 | 0 | 0 | 0.00046503 | 0 | 0 | 0 | 0 | 0 |
| k__Bacteria;p__Cyanobacteria;c__Synechococcophycideae;o__Synechococcales;f__Synechococcaceae;g__Synechococcus | | 0.00024526 | 1.49E-05 | 0 | 0 | 0.0001519 | 0.00239215 | 0.00673151 | 0.00398467 | 0.01205745 | 0 | 0.00042088 | 0.00012213 | 0.00012112 | 6.56E-05 | 0.00024084 |
| k__Bacteria;p__FBP;c__;o__;f__;g__ | | 0 | 0 | 0 | 0 | 0 | 0 | 0 | 0 | 0 | 0 | 0 | 0 | 0 | 0 | 0 |
| k__Bacteria;p__Fibrobacteres;c__Fibrobacteria;o__Fibrobacterales;f__;g__ | | 0 | 0 | 0 | 0 | 0 | 4.11E-05 | 0 | 0 | 0 | 0 | 0 | 0 | 0 | 0 | 0 |
| k__Bacteria;p__Firmicutes;c__Bacilli;o__Bacillales;f__;g__ | | 0 | 0.00053619 | 0 | 0 | 0 | 0 | 0 | 0 | 0.00029293 | 0 | 0 | 0 | 0 | 0 | 0 |
| k__Bacteria;p__Firmicutes;c__Bacilli;o__Bacillales;f__Bacillaceae;g__Anoxybacillus | | 0 | 0 | 0 | 0 | 0 | 0.00046034 | 0 | 0 | 0.00361803 | 0 | 0 | 0 | 0 | 0 | 0 |
| k__Bacteria;p__Firmicutes;c__Bacilli;o__Bacillales;f__Bacillaceae;g__Bacillus | | 0 | 0.003098 | 0 | 0 | 0 | 0 | 0 | 0 | 0.00277092 | 0 | 0 | 5.64E-05 | 0 | 0 | 0 |
| k__Bacteria;p__Firmicutes;c__Bacilli;o__Bacillales;f__Bacillaceae;g__Geobacillus | | 0 | 0.00011915 | 0 | 0 | 0 | 0 | 0 | 0 | 0.00138546 | 0 | 0 | 0 | 0 | 0 | 0 |
| k__Bacteria;p__Firmicutes;c__Bacilli;o__Bacillales;f__Bacillaceae;g__Marinococcus | | 0 | 0 | 0 | 0 | 0 | 0 | 0 | 0 | 0 | 0 | 0 | 0 | 0 | 0 | 0 |
| k__Bacteria;p__Firmicutes;c__Bacilli;o__Bacillales;f__Listeriaceae;g__ | | 0 | 0 | 0 | 0 | 0 | 0 | 0 | 0 | 0 | 0 | 0 | 0 | 0 | 0.00013117 | 0 |
| k__Bacteria;p__Firmicutes;c__Bacilli;o__Bacillales;f__Listeriaceae;g__Brochothrix | | 0 | 0 | 0 | 0 | 0 | 0 | 0 | 0 | 0 | 0 | 0 | 0 | 0 | 0 | 0 |

| Taxon | | 1180 | 1182 | 1183 | 1203 | 1211 | 1242 | 6475 | 6476 | 6477 | 1220 | 1221 | 1248 | 2032 | 2033 | 2034 |
|---|---|---|---|---|---|---|---|---|---|---|---|---|---|---|---|---|
| | Copepod genus | Acartia | Acartia | Acartia | Acartia | Acartia | Acartia | Acartia | Acartia | Acartia | Acartia | Acartia | Acartia | Acartia | Acartia | Acartia |
| | Type of sample | FullGut | FullGut | FullGut | FullGut | FullGut | FullGut | FullGut | FullGut | FullGut | Starved | Starved | Starved | Starved | Starved | Starved |
| k__Bacteria;p__Firmicutes;c__Bacilli;o__Bacillales;f__Paenibacillaceae;g__Paenibacillus | | 0 | 0 | 0 | 0 | 0 | 0.00027949 | 0 | 0 | 0.00045126 | 0 | 0 | 0 | 0 | 0 | 0 |
| k__Bacteria;p__Firmicutes;c__Bacilli;o__Bacillales;f__Planococcaceae;g__ | | 0.03404746 | 0.0075067 | 0.00012021 | 0.00039649 | 0.04481126 | 0.0546988 | 0.00647617 | 0 | 0 | 0 | 0 | 0 | 0 | 0 | 5.16E-05 |
| k__Bacteria;p__Firmicutes;c__Bacilli;o__Bacillales;f__Planococcaceae;g__Bacillus | | 0 | 0 | 0 | 0 | 0 | 0 | 0 | 0 | 0 | 0 | 0 | 0 | 0 | 0 | 0 |
| k__Bacteria;p__Firmicutes;c__Bacilli;o__Bacillales;f__Planococcaceae;g__Planococcus | | 0 | 0 | 0 | 0 | 0 | 0 | 0 | 0 | 0 | 0 | 0.00399832 | 0 | 0 | 0 | 0 |
| k__Bacteria;p__Firmicutes;c__Bacilli;o__Bacillales;f__Planococcaceae;g__Planomicrobium | | 0 | 0 | 0 | 0 | 0 | 0.00035348 | 0 | 0 | 0 | 0 | 0 | 0 | 0 | 0 | 0 |
| k__Bacteria;p__Firmicutes;c__Bacilli;o__Bacillales;f__Staphylococcaceae;g__Jeotgalicoccus | | 0 | 0 | 0 | 0 | 0 | 0 | 0 | 0 | 0 | 0 | 0 | 0 | 0 | 0 | 0 |
| k__Bacteria;p__Firmicutes;c__Bacilli;o__Bacillales;f__Staphylococcaceae;g__Staphylococcus | | 0.00190435 | 0.00043193 | 0.00036064 | 0.00047579 | 0.00162029 | 0.00170985 | 0.0051763 | 0.00136185 | 0 | 0.00548735 | 0.0111532 | 0.00015032 | 5.19E-05 | 6.56E-05 | 0.00013762 |
| k__Bacteria;p__Firmicutes;c__Bacilli;o__Bacillales;f__Thermoactinomycetaceae;g__ | | 0 | 0 | 0 | 0 | 0 | 0 | 0 | 0 | 0.00033251 | 0 | 0 | 0 | 0 | 0 | 0 |
| k__Bacteria;p__Firmicutes;c__Bacilli;o__Bacillales;f__Thermoactinomycetaceae;g__Planifilum | | 0 | 0 | 0 | 0 | 0 | 0 | 0 | 0 | 3.96E-05 | 0 | 0 | 0 | 0 | 0 | 0 |
| k__Bacteria;p__Firmicutes;c__Bacilli;o__Bacillales;f__[Exiguobacteraceae];g__Exiguobacterium | | 0 | 0 | 0 | 9.91E-05 | 0 | 0 | 0 | 0 | 0 | 0 | 0 | 0 | 0 | 0 | 0 |
| k__Bacteria;p__Firmicutes;c__Bacilli;o__Bacillales;f__[Thermicanaceae];g__Thermicanus | | 0 | 0 | 0 | 0 | 0 | 0 | 0 | 0 | 0.00079961 | 0 | 0 | 0 | 0 | 0 | 0 |
| k__Bacteria;p__Firmicutes;c__Bacilli;o__Gemellales;f__Gemellaceae;g__ | | 7.21E-05 | 0.00014894 | 0 | 0 | 0 | 0 | 0 | 0 | 0 | 0 | 0 | 0 | 0 | 0 | 8.60E-05 |
| k__Bacteria;p__Firmicutes;c__Bacilli;o__Gemellales;f__Gemellaceae;g__Gemella | | 0 | 0 | 0 | 0 | 0 | 0 | 0 | 0 | 0 | 0 | 0 | 0 | 0 | 0 | 1.72E-05 |
| k__Bacteria;p__Firmicutes;c__Bacilli;o__Lactobacillales;f__Aerococcaceae;g__ | | 0.00012984 | 0.00168305 | 0.00022039 | 5.95E-05 | 0 | 0 | 0 | 0 | 0 | 0 | 0 | 9.39E-06 | 1.73E-05 | 0 | 0 |
| k__Bacteria;p__Firmicutes;c__Bacilli;o__Lactobacillales;f__Aerococcaceae;g__Aerococcus | | 0 | 0 | 0 | 0.00017842 | 0 | 0 | 0 | 0 | 0 | 0 | 0 | 0 | 0 | 0 | 0 |
| k__Bacteria;p__Firmicutes;c__Bacilli;o__Lactobacillales;f__Aerococcaceae;g__Alloiococcus | | 0.00024526 | 0 | 0 | 0.0001586 | 0 | 0.00694627 | 0 | 0 | 0 | 0.00213914 | 0 | 0 | 0 | 0 | 5.16E-05 |
| k__Bacteria;p__Firmicutes;c__Bacilli;o__Lactobacillales;f__Aerococcaceae;g__Facklamia | | 0 | 0 | 0 | 0 | 0 | 0 | 0 | 0 | 0 | 0 | 0 | 0 | 0 | 0 | 0 |
| k__Bacteria;p__Firmicutes;c__Bacilli;o__Lactobacillales;f__Aerococcaceae;g__Marinilactibacillus | | 0 | 0 | 0 | 0 | 0 | 0 | 0 | 0 | 0 | 0 | 0 | 0 | 0 | 0 | 0 |
| k__Bacteria;p__Firmicutes;c__Bacilli;o__Lactobacillales;f__Carnobacteriaceae;g__ | | 0 | 0 | 0 | 0 | 0 | 0 | 0 | 7.57E-05 | 0 | 0 | 0 | 0 | 0 | 0 | 0 |
| k__Bacteria;p__Firmicutes;c__Bacilli;o__Lactobacillales;f__Carnobacteriaceae;g__Granulicatella | | 0 | 0.00023831 | 2.00E-05 | 7.93E-05 | 0.00022785 | 0.00012331 | 0 | 0 | 0 | 0 | 0 | 0 | 0 | 0 | 5.16E-05 |
| k__Bacteria;p__Firmicutes;c__Bacilli;o__Lactobacillales;f__Carnobacteriaceae;g__Trichococcus | | 0 | 0 | 0 | 7.93E-05 | 0 | 0 | 0 | 0.00124416 | 0 | 0 | 0 | 0 | 0 | 0 | 0 |
| k__Bacteria;p__Firmicutes;c__Bacilli;o__Lactobacillales;f__Enterococcaceae;g__Enterococcus | | 0.00014427 | 0 | 0 | 0 | 0 | 0 | 0 | 0 | 0 | 0 | 0 | 0 | 0 | 8.20E-05 | 0 |
| k__Bacteria;p__Firmicutes;c__Bacilli;o__Lactobacillales;f__Lactobacillaceae;g__Lactobacillus | | 0.00064921 | 0 | 0 | 5.95E-05 | 0 | 0 | 0 | 0 | 7.13E-05 | 0.00697545 | 0 | 1.88E-05 | 0 | 0 | 0.00034406 |
| k__Bacteria;p__Firmicutes;c__Bacilli;o__Lactobacillales;f__Leuconostocaceae;g__ | | 0 | 0 | 0 | 0 | 0 | 0 | 0 | 0 | 0 | 0 | 0 | 0 | 0 | 0 | 0 |
| k__Bacteria;p__Firmicutes;c__Bacilli;o__Lactobacillales;f__Leuconostocaceae;g__Leuconostoc | | 0 | 0 | 0 | 0 | 0 | 0 | 0 | 0 | 0 | 0 | 0 | 0 | 0 | 0 | 0 |
| k__Bacteria;p__Firmicutes;c__Bacilli;o__Lactobacillales;f__Leuconostocaceae;g__Weissella | | 0 | 0 | 0 | 0 | 0 | 0 | 0 | 0 | 0.0001425 | 0 | 0 | 0 | 0 | 0 | 0 |
| k__Bacteria;p__Firmicutes;c__Bacilli;o__Lactobacillales;f__Streptococcaceae;g__Lactococcus | | 0.00031739 | 0 | 0 | 0 | 0 | 4.11E-05 | 0 | 0 | 0 | 0 | 0 | 0 | 0 | 0 | 0 |
| k__Bacteria;p__Firmicutes;c__Bacilli;o__Lactobacillales;f__Streptococcaceae;g__Streptococcus | | 0.00285652 | 0.00123622 | 0.000561 | 0.00019825 | 0.00040507 | 0.00142214 | 0.00343539 | 0.00033626 | 0.00083919 | 0.03580729 | 0.02819865 | 0 | 0 | 1.64E-05 | 0.00049889 |
| k__Bacteria;p__Firmicutes;c__Clostridia;o__Clostridiales;f__;g__ | | 1.44E-05 | 0 | 0 | 1.98E-05 | 0 | 0 | 0 | 0 | 0 | 0 | 0 | 0 | 0 | 0 | 0 |
| k__Bacteria;p__Firmicutes;c__Clostridia;o__Clostridiales;f__Clostridiaceae;g__ | | 7.21E-05 | 0 | 0 | 0 | 0 | 0 | 0 | 0 | 0 | 0 | 0 | 0 | 0 | 0 | 0 |
| k__Bacteria;p__Firmicutes;c__Clostridia;o__Clostridiales;f__Clostridiaceae;g__Clostridium | | 0 | 0 | 0 | 0 | 0 | 0.0007234 | 0 | 0 | 0 | 0 | 0 | 1.88E-05 | 0 | 0 | 0 |
| k__Bacteria;p__Firmicutes;c__Clostridia;o__Clostridiales;f__Lachnospiraceae;g__ | | 0 | 0 | 0 | 0 | 0 | 0 | 0 | 0 | 0.00019001 | 0 | 0 | 0 | 0 | 0 | 3.44E-05 |
| k__Bacteria;p__Firmicutes;c__Clostridia;o__Clostridiales;f__Lachnospiraceae;g__Blautia | | 0 | 0 | 0 | 0 | 0 | 0 | 0 | 0 | 0 | 0 | 0 | 0 | 0 | 0 | 0 |
| k__Bacteria;p__Firmicutes;c__Clostridia;o__Clostridiales;f__Lachnospiraceae;g__Dorea | | 0 | 0 | 0 | 0 | 0 | 0 | 0 | 0 | 0 | 0 | 0.00799663 | 0 | 0 | 0 | 0 |
| k__Bacteria;p__Firmicutes;c__Clostridia;o__Clostridiales;f__Lachnospiraceae;g__Moryella | | 0.00014427 | 0 | 0 | 0 | 0 | 0 | 0 | 0 | 0 | 0 | 0 | 1.88E-05 | 0 | 0 | 5.16E-05 |
| k__Bacteria;p__Firmicutes;c__Clostridia;o__Clostridiales;f__Lachnospiraceae;g__Oribacterium | | 0 | 0 | 0 | 0 | 0 | 0 | 0 | 0 | 0 | 0 | 0 | 0 | 0 | 0 | 0 |
| k__Bacteria;p__Firmicutes;c__Clostridia;o__Clostridiales;f__Lachnospiraceae;g__[Ruminococcus] | | 0 | 0 | 0 | 0 | 0 | 0 | 0 | 0 | 0 | 0 | 0 | 0 | 0 | 0 | 0 |
| k__Bacteria;p__Firmicutes;c__Clostridia;o__Clostridiales;f__Peptostreptococcaceae;g__ | | 0 | 0 | 0 | 0 | 0 | 0 | 0 | 0.00019335 | 0 | 0 | 0 | 0 | 0 | 0 | 0 |
| k__Bacteria;p__Firmicutes;c__Clostridia;o__Clostridiales;f__Ruminococcaceae;g__ | | 0 | 0.00014894 | 0 | 0 | 0 | 0 | 0 | 0 | 0.0004196 | 0 | 0 | 0 | 0 | 0 | 0 |
| k__Bacteria;p__Firmicutes;c__Clostridia;o__Clostridiales;f__Ruminococcaceae;g__Clostridium | | 0 | 0 | 0 | 0 | 0 | 0 | 0 | 0 | 1.58E-05 | 0 | 0 | 0 | 0 | 0 | 0 |
| k__Bacteria;p__Firmicutes;c__Clostridia;o__Clostridiales;f__Ruminococcaceae;g__Faecalibacterium | | 0 | 0 | 0 | 0 | 0 | 0 | 0 | 0 | 0 | 0 | 0 | 0 | 0 | 0 | 0 |
| k__Bacteria;p__Firmicutes;c__Clostridia;o__Clostridiales;f__Ruminococcaceae;g__Oscillospira | | 0 | 0 | 0 | 0 | 0 | 0 | 0 | 0 | 0 | 0 | 0 | 0 | 0 | 0 | 0 |
| k__Bacteria;p__Firmicutes;c__Clostridia;o__Clostridiales;f__Veillonellaceae;g__ | | 0 | 0 | 0 | 0 | 0 | 0 | 0 | 0 | 0 | 0 | 0 | 0 | 0 | 0 | 0 |
| k__Bacteria;p__Firmicutes;c__Clostridia;o__Clostridiales;f__Veillonellaceae;g__Anaeromusa | | 0 | 0 | 0 | 0 | 0 | 0 | 0 | 0 | 0 | 0 | 0 | 0 | 0 | 0 | 0 |
| k__Bacteria;p__Firmicutes;c__Clostridia;o__Clostridiales;f__Veillonellaceae;g__Dialister | | 0 | 0 | 0 | 0 | 0 | 0 | 0 | 0.00010088 | 0 | 0 | 0 | 0 | 0 | 0 | 1.72E-05 |
| k__Bacteria;p__Firmicutes;c__Clostridia;o__Clostridiales;f__Veillonellaceae;g__Pelosinus | | 0 | 0 | 0 | 0 | 0 | 0 | 0 | 0 | 0 | 0 | 0 | 0 | 0 | 0 | 0 |
| k__Bacteria;p__Firmicutes;c__Clostridia;o__Clostridiales;f__Veillonellaceae;g__Phascolarctobacterium | | 0 | 0 | 0 | 0 | 0 | 0 | 0 | 0 | 0 | 0 | 0 | 0 | 0 | 0 | 0 |
| k__Bacteria;p__Firmicutes;c__Clostridia;o__Clostridiales;f__Veillonellaceae;g__Selenomonas | | 0 | 0 | 0 | 0 | 0 | 0 | 0 | 0 | 0.00018209 | 0 | 0 | 0 | 0 | 0 | 0 |
| k__Bacteria;p__Firmicutes;c__Clostridia;o__Clostridiales;f__Veillonellaceae;g__Veillonella | | 0 | 0 | 0.00016029 | 0 | 0 | 0.00032882 | 0 | 0 | 0 | 0 | 0.003367 | 2.82E-05 | 0 | 0 | 0.00018923 |
| k__Bacteria;p__Firmicutes;c__Clostridia;o__Clostridiales;f__[Acidaminobacteraceae];g__Fusibacter | | 0 | 0 | 0 | 0 | 0 | 0 | 0 | 0 | 0 | 0 | 0 | 0 | 0 | 0 | 0 |
| k__Bacteria;p__Firmicutes;c__Clostridia;o__Clostridiales;f__[Acidaminobacteraceae];g__WH1-8 | | 0 | 0 | 0 | 0 | 0 | 0 | 0 | 0 | 0 | 0 | 0 | 0 | 0 | 0 | 0 |
| k__Bacteria;p__Firmicutes;c__Clostridia;o__Clostridiales;f__[Mogibacteriaceae];g__ | | 0.00012984 | 0 | 0 | 0 | 0 | 0 | 0 | 0 | 0 | 0 | 0 | 0 | 0 | 0 | 0 |
| k__Bacteria;p__Firmicutes;c__Clostridia;o__Clostridiales;f__[Mogibacteriaceae];g__Mogibacterium | | 0 | 0 | 0 | 0 | 0 | 0 | 0 | 0 | 0 | 0 | 0 | 0 | 0 | 0 | 0 |
| k__Bacteria;p__Firmicutes;c__Clostridia;o__Clostridiales;f__[Tissierellaceae];g__Anaerococcus | | 0.00034625 | 0 | 6.01E-05 | 9.91E-05 | 0.0007342 | 0.0004439 | 0.0078689 | 0 | 0 | 0 | 0 | 0 | 0 | 3.28E-05 | 0.00012042 |
| k__Bacteria;p__Firmicutes;c__Clostridia;o__Clostridiales;f__[Tissierellaceae];g__Finegoldia | | 0 | 0.0002681 | 0 | 0 | 0 | 0 | 0 | 0 | 0 | 0 | 0 | 0 | 0 | 0 | 5.16E-05 |
| k__Bacteria;p__Firmicutes;c__Clostridia;o__Clostridiales;f__[Tissierellaceae];g__Gallicola | | 0 | 0 | 0 | 0 | 0 | 0 | 0 | 0 | 0 | 0.00260417 | 0 | 0 | 0 | 0 | 1.72E-05 |
| k__Bacteria;p__Firmicutes;c__Clostridia;o__Clostridiales;f__[Tissierellaceae];g__Parvimonas | | 0 | 0 | 0 | 0 | 0 | 0 | 0 | 0 | 0.00017417 | 0 | 0 | 0 | 0 | 0 | 0 |
| k__Bacteria;p__Firmicutes;c__Clostridia;o__Clostridiales;f__[Tissierellaceae];g__Peptoniphilus | | 0 | 0.00034257 | 0 | 0 | 0 | 0 | 0 | 0 | 0 | 0 | 0 | 0 | 0 | 0 | 6.88E-05 |
| k__Bacteria;p__Firmicutes;c__Clostridia;o__Clostridiales;f__[Tissierellaceae];g__WAL_1855D | | 0 | 0 | 0 | 0 | 0 | 0 | 0 | 0 | 0 | 0 | 0 | 0 | 0 | 0 | 5.16E-05 |
| k__Bacteria;p__Firmicutes;c__Clostridia;o__Thermoanaerobacterales;f__Caldicellulosiruptoraceae;g__Caldicellulosiruptor | | 0 | 0 | 0 | 0 | 0 | 0 | 0 | 0 | 0.00066502 | 0 | 0 | 0 | 0 | 0 | 0 |
| k__Bacteria;p__Firmicutes;c__Erysipelotrichi;o__Erysipelotrichales;f__Erysipelotrichaceae;g__ | | 0 | 0.00044683 | 0 | 0 | 0 | 0 | 0 | 0 | 0 | 0 | 0 | 0 | 0 | 0 | 0 |
| k__Bacteria;p__Firmicutes;c__Erysipelotrichi;o__Erysipelotrichales;f__Erysipelotrichaceae;g__Coprobacillus | | 0 | 0 | 0 | 0 | 0 | 0 | 0 | 0 | 0 | 0 | 0 | 0 | 0 | 0 | 0 |
| k__Bacteria;p__Firmicutes;c__Erysipelotrichi;o__Erysipelotrichales;f__Erysipelotrichaceae;g__RFN20 | | 0 | 0 | 0 | 0 | 0 | 0 | 0 | 8.41E-05 | 0 | 0 | 0 | 0 | 0 | 0 | 0 |
| k__Bacteria;p__Firmicutes;c__Erysipelotrichi;o__Erysipelotrichales;f__Erysipelotrichaceae;g__[Eubacterium] | | 0 | 0.00014894 | 0 | 0 | 0 | 0 | 0 | 0 | 0 | 0 | 0 | 0 | 0 | 0 | 0 |
| k__Bacteria;p__Fusobacteria;c__Fusobacteriia;o__Fusobacteriales;f__;g__ | | 0 | 0 | 0 | 0 | 0 | 0 | 0 | 0 | 0 | 0 | 0 | 0 | 0 | 0 | 0 |
| k__Bacteria;p__Fusobacteria;c__Fusobacteriia;o__Fusobacteriales;f__Fusobacteriaceae;g__Cetobacterium | | 0 | 0 | 0 | 0 | 0 | 0 | 0 | 0 | 0 | 0 | 0 | 0 | 0 | 0 | 0 |
| k__Bacteria;p__Fusobacteria;c__Fusobacteriia;o__Fusobacteriales;f__Fusobacteriaceae;g__Fusobacterium | | 0 | 0.00019363 | 0.00020036 | 0 | 0.00032912 | 0.00023017 | 0.00146236 | 0 | 0.00071252 | 0 | 0 | 0 | 0 | 0 | 0 |
| k__Bacteria;p__Fusobacteria;c__Fusobacteriia;o__Fusobacteriales;f__Fusobacteriaceae;g__Propionigenium | | 0 | 0 | 0 | 0 | 0 | 0 | 0 | 0 | 0 | 0 | 0 | 0 | 0 | 0 | 0 |
| k__Bacteria;p__Fusobacteria;c__Fusobacteriia;o__Fusobacteriales;f__Fusobacteriaceae;g__Psychrilyobacter | | 0 | 0 | 0 | 0 | 0 | 0 | 0 | 0 | 0 | 0 | 0 | 0 | 0 | 0 | 0 |
| k__Bacteria;p__Fusobacteria;c__Fusobacteriia;o__Fusobacteriales;f__Fusobacteriaceae;g__u114 | | 0 | 0 | 0 | 0 | 0 | 0 | 0 | 0 | 0 | 0 | 0 | 0 | 0 | 0 | 0 |
| k__Bacteria;p__Fusobacteria;c__Fusobacteriia;o__Fusobacteriales;f__Leptotrichiaceae;g__Leptotrichia | | 0 | 0 | 0 | 7.93E-05 | 0 | 0 | 0.00123024 | 0 | 0 | 0 | 0 | 0 | 0 | 6.56E-05 | 0 |
| k__Bacteria;p__GN02;c__;o__;f__;g__ | | 0 | 0 | 0 | 0 | 0 | 0 | 0 | 0 | 0 | 0 | 0 | 0 | 0 | 0 | 0 |
| k__Bacteria;p__GN02;c__BD1-5;o__;f__;g__ | | 0 | 1.49E-05 | 0 | 0 | 0 | 0.00049323 | 0 | 0 | 0.00297676 | 0 | 0 | 4.70E-05 | 0.02064157 | 0.05504181 | 0.00887681 |
| k__Bacteria;p__GN02;c__IIB17;o__;f__;g__ | | 0 | 0 | 0 | 0 | 0 | 0 | 0 | 0 | 0 | 0 | 0 | 0 | 0 | 0 | 0 |

| Taxon | | 1180 | 1182 | 1183 | 1203 | 1211 | 1242 | 6475 | 6476 | 6477 | 1220 | 1221 | 1248 | 2032 | 2033 | 2034 |
|---|---|---|---|---|---|---|---|---|---|---|---|---|---|---|---|---|
| | Copepod genus | Acartia | Acartia | Acartia | Acartia | Acartia | Acartia | Acartia | Acartia | Acartia | Acartia | Acartia | Acartia | Acartia | Acartia | Acartia |
| | Type of sample | FullGut | FullGut | FullGut | FullGut | FullGut | FullGut | FullGut | FullGut | FullGut | Starved | Starved | Starved | Starved | Starved | Starved |
| k__Bacteria;p__Gemmatimonadetes;c__Gemmatimonadetes;o__;f__;g__ | | 0 | 0 | 0 | 0 | 0 | 0.00064941 | 0 | 0 | 0 | 0 | 0 | 0 | 0 | 0 | 0 |
| k__Bacteria;p__Gemmatimonadetes;c__Gemmatimonadetes;o__Gemmatimonadales;f__;g__ | | 0 | 0 | 0 | 0 | 0 | 0 | 0.00167127 | 0 | 0 | 0 | 0 | 0 | 0 | 0 | 0 |
| k__Bacteria;p__Lentisphaerae;c__[Lentisphaeria];o__Lentisphaerales;f__;g__ | | 0 | 0 | 0 | 0 | 0 | 0 | 0 | 0 | 0 | 0 | 0 | 0 | 0 | 0 | 0 |
| k__Bacteria;p__Lentisphaerae;c__[Lentisphaeria];o__Lentisphaerales;f__Lentisphaeraceae;g__ | | 0 | 0 | 0 | 0 | 0 | 0 | 0 | 0 | 0 | 0 | 0 | 0 | 0 | 0 | 0 |
| k__Bacteria;p__Lentisphaerae;c__[Lentisphaeria];o__Lentisphaerales;f__Lentisphaeraceae;g__Lentisphaera | | 0 | 0 | 0 | 0 | 0 | 0.00023017 | 0 | 0 | 0 | 0 | 0 | 0 | 0 | 0 | 0 |
| k__Bacteria;p__Lentisphaerae;c__[Lentisphaeria];o__Victivallales;f__Victivallaceae;g__ | | 0 | 0 | 0 | 0 | 0 | 0 | 0 | 0 | 0 | 0 | 0 | 0 | 0 | 0 | 0 |
| k__Bacteria;p__NKB19;c__SHAB590;o__;f__;g__ | | 0 | 0 | 0 | 0 | 0 | 0 | 0 | 0 | 0 | 0 | 0 | 0 | 0 | 0 | 0 |
| k__Bacteria;p__Nitrospirae;c__Nitrospira;o__Nitrospirales;f__Nitrospiraceae;g__ | | 0 | 0 | 0 | 0 | 0 | 0 | 0 | 0 | 0 | 0 | 0 | 0 | 0 | 0 | 0 |
| k__Bacteria;p__OD1;c__;o__;f__;g__ | | 0 | 0 | 0 | 0 | 0 | 0 | 0 | 0 | 0 | 0 | 0 | 0 | 0 | 0 | 0 |
| k__Bacteria;p__OD1;c__ABY1;o__;f__;g__ | | 0 | 0 | 0 | 0 | 0 | 0 | 0.00125345 | 0 | 0 | 0 | 0 | 0 | 0 | 0 | 0 |
| k__Bacteria;p__OD1;c__SM2F11;o__;f__;g__ | | 5.77E-05 | 0.00014894 | 0 | 0 | 5.06E-05 | 0 | 0 | 0 | 0 | 0 | 0 | 0 | 0 | 0 | 0 |
| k__Bacteria;p__OD1;c__ZB2;o__;f__;g__ | | 0 | 0 | 0 | 0 | 0 | 0 | 0 | 0 | 0 | 0 | 0 | 0 | 0 | 0 | 0 |
| k__Bacteria;p__OP8;c__OP8_1;o__HMMVPog-54;f__;g__ | | 0 | 0 | 0 | 0 | 0 | 0 | 0 | 0 | 0 | 0 | 0 | 0 | 0 | 0 | 0 |
| k__Bacteria;p__Planctomycetes;c__OM190;o__CL500-15;f__;g__ | | 0 | 0 | 0 | 0 | 0 | 0 | 0 | 0 | 0 | 0 | 0 | 0 | 0 | 0 | 0 |
| k__Bacteria;p__Planctomycetes;c__OM190;o__agg27;f__;g__ | | 0 | 0 | 0 | 0 | 0 | 0 | 0 | 0 | 0.00016625 | 0 | 0 | 0 | 0 | 0 | 3.44E-05 |
| k__Bacteria;p__Planctomycetes;c__Phycisphaerae;o__Phycisphaerales;f__;g__ | | 0 | 0 | 0 | 0 | 0 | 0.00018907 | 0 | 0 | 0.00047501 | 0 | 0 | 0 | 5.19E-05 | 0 | 0 |
| k__Bacteria;p__Planctomycetes;c__Phycisphaerae;o__Phycisphaerales;f__Phycisphaeraceae;g__ | | 0 | 0 | 0 | 0 | 0 | 0 | 0 | 0 | 0 | 0 | 0 | 0 | 1.73E-05 | 0 | 0 |
| k__Bacteria;p__Planctomycetes;c__Planctomycetia;o__Gemmatales;f__Gemmataceae;g__ | | 0 | 0 | 0 | 0 | 0 | 0 | 0 | 0 | 0 | 0 | 0 | 0 | 0 | 0 | 0 |
| k__Bacteria;p__Planctomycetes;c__Planctomycetia;o__Gemmatales;f__Isosphaeraceae;g__ | | 0 | 0 | 0 | 0 | 0 | 0 | 0 | 0 | 0 | 0 | 0 | 0 | 0 | 0 | 0 |
| k__Bacteria;p__Planctomycetes;c__Planctomycetia;o__Pirellulales;f__Pirellulaceae;g__ | | 0.00038953 | 0.00043193 | 0.00070125 | 9.91E-05 | 0 | 0.00154544 | 0.00385321 | 0.00162245 | 0.00140921 | 0 | 0 | 0 | 5.19E-05 | 4.92E-05 | 0.00010322 |
| k__Bacteria;p__Planctomycetes;c__Planctomycetia;o__Pirellulales;f__Pirellulaceae;g__Planctomycete | | 0 | 0 | 0 | 0 | 0 | 0 | 0 | 0 | 0 | 0 | 0 | 0 | 0 | 0 | 0 |
| k__Bacteria;p__Planctomycetes;c__Planctomycetia;o__Pirellulales;f__Pirellulaceae;g__Rhodopirellula | | 0 | 0 | 0 | 0 | 0 | 0.00020551 | 0 | 0 | 0 | 0 | 0 | 0 | 0 | 0 | 0 |
| k__Bacteria;p__Planctomycetes;c__Planctomycetia;o__Planctomycetales;f__Planctomycetaceae;g__Planctomyces | | 0 | 0 | 0 | 0 | 0 | 0 | 0 | 0 | 0 | 0 | 0 | 0 | 0 | 0 | 0 |
| k__Bacteria;p__Planctomycetes;c__vadinHA49;o__;f__;g__ | | 0 | 0 | 0 | 0 | 0 | 0.00027127 | 0 | 0 | 0 | 0 | 0 | 0 | 0 | 0 | 0 |
| k__Bacteria;p__Proteobacteria;c__Alphaproteobacteria;o__;f__;g__ | | 0.01858184 | 0.01407507 | 0.00530945 | 5.95E-05 | 0 | 0.01487077 | 0.00559411 | 0.00277413 | 0.00813858 | 0 | 0 | 0.00561814 | 0.00494844 | 0.00286932 | 0.00596948 |
| k__Bacteria;p__Proteobacteria;c__Alphaproteobacteria;o__BD7-3;f__;g__ | | 0 | 0 | 0 | 0 | 0 | 0.0019318 | 0 | 0.00243788 | 0.00476598 | 0 | 0 | 0.00301576 | 0.00012112 | 0.00011477 | 8.60E-05 |
| k__Bacteria;p__Proteobacteria;c__Alphaproteobacteria;o__Caulobacterales;f__Caulobacteraceae;g__ | | 0 | 0.0002532 | 0 | 0.00025772 | 0 | 0.00093713 | 0 | 0 | 0.00032459 | 0.00595238 | 0 | 8.46E-05 | 0 | 0 | 1.72E-05 |
| k__Bacteria;p__Proteobacteria;c__Alphaproteobacteria;o__Caulobacterales;f__Caulobacteraceae;g__Brevundimonas | | 0 | 0.00064045 | 0 | 0 | 0 | 0.00022195 | 0 | 0 | 0 | 0.00186012 | 0 | 0 | 0 | 0 | 0 |
| k__Bacteria;p__Proteobacteria;c__Alphaproteobacteria;o__Caulobacterales;f__Caulobacteraceae;g__Mycoplana | | 0 | 0 | 0 | 0 | 0 | 0 | 0 | 5.04E-05 | 0 | 0 | 0 | 0 | 0 | 0 | 0 |
| k__Bacteria;p__Proteobacteria;c__Alphaproteobacteria;o__Caulobacterales;f__Caulobacteraceae;g__Phenylobacterium | | 0 | 0.00035746 | 0 | 0 | 0 | 0.00018085 | 0 | 0 | 0 | 0 | 0 | 0 | 0 | 0.00013117 | 1.72E-05 |
| k__Bacteria;p__Proteobacteria;c__Alphaproteobacteria;o__Ellin329;f__;g__ | | 0 | 0 | 0 | 0 | 0 | 0 | 0 | 0 | 0 | 0 | 0 | 0 | 0 | 0 | 0 |
| k__Bacteria;p__Proteobacteria;c__Alphaproteobacteria;o__Kiloniellales;f__;g__ | | 0 | 0 | 0 | 0 | 0 | 0 | 0 | 0.00010088 | 0 | 0 | 0 | 2.82E-05 | 0 | 4.92E-05 | 8.60E-05 |
| k__Bacteria;p__Proteobacteria;c__Alphaproteobacteria;o__Kiloniellales;f__Kiloniellaceae;g__ | | 0.0018755 | 0.00131069 | 0.00034061 | 0 | 0 | 0 | 0 | 0.0001261 | 0.0005146 | 0 | 0 | 0 | 0 | 0 | 1.72E-05 |
| k__Bacteria;p__Proteobacteria;c__Alphaproteobacteria;o__Kiloniellales;f__Kiloniellaceae;g__Thalassospira | | 0 | 0 | 0 | 0 | 0 | 0 | 0 | 0 | 0 | 0 | 0 | 0 | 0 | 0 | 1.72E-05 |
| k__Bacteria;p__Proteobacteria;c__Alphaproteobacteria;o__Kordiimonadales;f__Kordiimonadaceae;g__ | | 0 | 0 | 0 | 0 | 0 | 0 | 0 | 0 | 0 | 0 | 0 | 0 | 1.73E-05 | 0 | 0.00012042 |
| k__Bacteria;p__Proteobacteria;c__Alphaproteobacteria;o__Rhizobiales;f__;g__ | | 0.00057708 | 0 | 0.00014025 | 0 | 0 | 0 | 0 | 0 | 0.00064919 | 0 | 0 | 9.39E-06 | 3.46E-05 | 1.64E-05 | 0.00013762 |
| k__Bacteria;p__Proteobacteria;c__Alphaproteobacteria;o__Rhizobiales;f__Aurantimonadaceae;g__Martelella | | 0 | 0 | 0 | 0 | 0 | 0 | 0 | 0 | 0 | 0 | 0 | 0 | 0 | 0 | 0 |
| k__Bacteria;p__Proteobacteria;c__Alphaproteobacteria;o__Rhizobiales;f__Beijerinckiaceae;g__Chelatococcus | | 0 | 0 | 0 | 0 | 0 | 0 | 0 | 0 | 0 | 0 | 0 | 0 | 0 | 0 | 0 |
| k__Bacteria;p__Proteobacteria;c__Alphaproteobacteria;o__Rhizobiales;f__Bradyrhizobiaceae;g__ | | 0.00010099 | 0 | 0.00034061 | 0 | 0.00043039 | 0.00043568 | 0.00369072 | 0.00010088 | 0.00038001 | 0 | 0 | 0.00016911 | 3.46E-05 | 0 | 0 |
| k__Bacteria;p__Proteobacteria;c__Alphaproteobacteria;o__Rhizobiales;f__Bradyrhizobiaceae;g__Balneimonas | | 0 | 0 | 0 | 0 | 0 | 0 | 0 | 0 | 0.00019001 | 0 | 0 | 0 | 0 | 0 | 0 |
| k__Bacteria;p__Proteobacteria;c__Alphaproteobacteria;o__Rhizobiales;f__Bradyrhizobiaceae;g__Pseudomonas | | 0 | 0 | 0 | 0 | 0 | 0 | 0 | 0 | 1.58E-05 | 0 | 0 | 0 | 0 | 0 | 0 |
| k__Bacteria;p__Proteobacteria;c__Alphaproteobacteria;o__Rhizobiales;f__Cohaesibacteraceae;g__Cohaesibacter | | 0 | 0 | 0 | 0 | 0 | 8.22E-06 | 0 | 0 | 0 | 0 | 0 | 0 | 0 | 0 | 0 |
| k__Bacteria;p__Proteobacteria;c__Alphaproteobacteria;o__Rhizobiales;f__Hyphomicrobiaceae;g__ | | 0 | 0 | 0 | 0 | 0 | 0 | 0.00392284 | 0 | 0 | 0 | 0 | 0 | 0 | 0 | 0 |
| k__Bacteria;p__Proteobacteria;c__Alphaproteobacteria;o__Rhizobiales;f__Hyphomicrobiaceae;g__Devosia | | 0 | 0.00010426 | 0 | 0 | 0 | 0.00258944 | 0 | 0.00066411 | 0.00361011 | 0 | 0 | 0.0012777 | 3.46E-05 | 8.20E-05 | 0 |
| k__Bacteria;p__Proteobacteria;c__Alphaproteobacteria;o__Rhizobiales;f__Hyphomicrobiaceae;g__Rhodoplanes | | 0 | 0 | 0 | 0 | 0 | 0 | 0 | 0.00010088 | 0 | 0 | 0 | 0 | 0 | 0 | 0 |
| k__Bacteria;p__Proteobacteria;c__Alphaproteobacteria;o__Rhizobiales;f__Methylobacteriaceae;g__ | | 0.00011542 | 0 | 0 | 0 | 0 | 0 | 0.00148557 | 0.0001345 | 7.92E-05 | 0 | 0 | 0 | 0 | 3.28E-05 | 0 |
| k__Bacteria;p__Proteobacteria;c__Alphaproteobacteria;o__Rhizobiales;f__Methylobacteriaceae;g__Methylobacterium | | 0 | 0.00017873 | 0 | 0 | 0.00035444 | 0.00022195 | 0.00016248 | 5.04E-05 | 0.00030084 | 0 | 0 | 0 | 0 | 0 | 0 |
| k__Bacteria;p__Proteobacteria;c__Alphaproteobacteria;o__Rhizobiales;f__Methylocystaceae;g__ | | 0 | 0.00010426 | 0 | 0 | 0 | 6.58E-05 | 0 | 0 | 0.00010292 | 0 | 0 | 0 | 0 | 0 | 0 |
| k__Bacteria;p__Proteobacteria;c__Alphaproteobacteria;o__Rhizobiales;f__Methylocystaceae;g__Methylosinus | | 0 | 0 | 0 | 0 | 0 | 0 | 0 | 0 | 0 | 0 | 0 | 0 | 0 | 0 | 0 |
| k__Bacteria;p__Proteobacteria;c__Alphaproteobacteria;o__Rhizobiales;f__Methylocystaceae;g__Pleomorphomonas | | 0 | 0 | 0 | 0 | 0 | 0 | 0 | 0 | 0 | 0 | 0 | 0 | 0 | 0 | 0 |
| k__Bacteria;p__Proteobacteria;c__Alphaproteobacteria;o__Rhizobiales;f__Phyllobacteriaceae;g__ | | 0 | 0 | 0 | 0 | 0.00040507 | 0.00036992 | 0 | 0.00125256 | 0.00394262 | 0 | 0.00021044 | 0.00031943 | 0 | 0 | 3.44E-05 |
| k__Bacteria;p__Proteobacteria;c__Alphaproteobacteria;o__Rhizobiales;f__Phyllobacteriaceae;g__Chelativorans | | 0 | 0 | 0 | 0 | 0 | 0 | 0 | 5.04E-05 | 0.0001425 | 0 | 0 | 0 | 0 | 0 | 0 |
| k__Bacteria;p__Proteobacteria;c__Alphaproteobacteria;o__Rhizobiales;f__Phyllobacteriaceae;g__Hoeflea | | 0 | 0 | 0 | 0 | 0 | 0.00167697 | 0 | 0.00265644 | 0.01702134 | 0 | 0 | 6.58E-05 | 5.19E-05 | 0 | 0 |
| k__Bacteria;p__Proteobacteria;c__Alphaproteobacteria;o__Rhizobiales;f__Phyllobacteriaceae;g__Mesorhizobium | | 0 | 0 | 0 | 0 | 0 | 0 | 0 | 8.41E-06 | 0 | 0 | 0 | 0 | 0 | 0 | 0 |
| k__Bacteria;p__Proteobacteria;c__Alphaproteobacteria;o__Rhizobiales;f__Phyllobacteriaceae;g__Phyllobacterium | | 0 | 0 | 0 | 0 | 0 | 0 | 0 | 0 | 0 | 9.30E-05 | 0.00021044 | 0 | 0 | 0 | 0 |
| k__Bacteria;p__Proteobacteria;c__Alphaproteobacteria;o__Rhizobiales;f__Rhizobiaceae;g__ | | 0 | 0 | 0 | 0 | 0 | 0 | 0 | 0 | 0.00045126 | 0 | 0 | 0 | 0 | 0 | 0 |
| k__Bacteria;p__Proteobacteria;c__Alphaproteobacteria;o__Rhizobiales;f__Rhizobiaceae;g__Agrobacterium | | 0 | 0.0002681 | 0 | 0 | 0.00032912 | 0 | 0 | 0 | 0.00041168 | 0 | 0.00484007 | 0 | 0 | 0 | 0 |
| k__Bacteria;p__Proteobacteria;c__Alphaproteobacteria;o__Rhizobiales;f__Rhizobiaceae;g__Rhizobium | | 0.00014427 | 0 | 0 | 0.00011895 | 0.00055698 | 0 | 0 | 0 | 0 | 0 | 0 | 0 | 0 | 0 | 0 |
| k__Bacteria;p__Proteobacteria;c__Alphaproteobacteria;o__Rhizobiales;f__Rhizobiaceae;g__Shinella | | 0 | 0 | 0 | 0 | 0 | 0 | 0 | 0 | 0 | 0 | 0 | 0 | 0 | 0 | 0 |
| k__Bacteria;p__Proteobacteria;c__Alphaproteobacteria;o__Rhizobiales;f__Xanthobacteraceae;g__Xanthobacter | | 0 | 0 | 0 | 0 | 0.00139244 | 0 | 0 | 0 | 0 | 0 | 0 | 0 | 0 | 0 | 0 |
| k__Bacteria;p__Proteobacteria;c__Alphaproteobacteria;o__Rhodobacterales;f__Hyphomonadaceae;g__ | | 0.00108202 | 0.00023831 | 0.00080143 | 0.00035685 | 0 | 0.00127417 | 0 | 4.20E-05 | 0.00015042 | 0 | 0 | 2.82E-05 | 1.73E-05 | 0.00011477 | 5.16E-05 |
| k__Bacteria;p__Proteobacteria;c__Alphaproteobacteria;o__Rhodobacterales;f__Hyphomonadaceae;g__Maricaulis | | 0 | 0 | 0 | 0 | 0 | 0 | 0 | 0 | 0 | 0 | 0 | 0 | 0 | 0 | 0 |
| k__Bacteria;p__Proteobacteria;c__Alphaproteobacteria;o__Rhodobacterales;f__Hyphomonadaceae;g__Robiginitomaculum | | 0 | 0 | 0 | 0 | 0 | 0 | 0 | 0 | 0 | 0 | 0 | 0 | 0 | 0 | 0 |
| k__Bacteria;p__Proteobacteria;c__Alphaproteobacteria;o__Rhodobacterales;f__Rhodobacteraceae;g__ | | 0.06018899 | 0.05564492 | 0.01510689 | 0.80383411 | 0.3158561 | 0.04939662 | 0.61502751 | 0.71448267 | 0.35631611 | 0.13885789 | 0.10690236 | 0.02893622 | 0.05296214 | 0.10967372 | 0.07220148 |
| k__Bacteria;p__Proteobacteria;c__Alphaproteobacteria;o__Rhodobacterales;f__Rhodobacteraceae;g__Amaricoccus | | 0 | 0 | 0 | 0 | 0 | 0 | 0 | 0 | 0 | 0 | 0 | 0 | 0 | 0 | 0 |
| k__Bacteria;p__Proteobacteria;c__Alphaproteobacteria;o__Rhodobacterales;f__Rhodobacteraceae;g__Anaerospora | | 0 | 0 | 0 | 0 | 0 | 0.00052611 | 0 | 0.00072296 | 0.00340427 | 0 | 0 | 4.70E-05 | 0 | 0.00013117 | 3.44E-05 |
| k__Bacteria;p__Proteobacteria;c__Alphaproteobacteria;o__Rhodobacterales;f__Rhodobacteraceae;g__Celeribacter | | 0 | 0 | 0 | 0 | 0 | 0 | 0 | 0 | 0 | 0 | 0 | 0 | 0 | 0 | 0 |
| k__Bacteria;p__Proteobacteria;c__Alphaproteobacteria;o__Rhodobacterales;f__Rhodobacteraceae;g__Dinoroseobacter | | 0 | 0 | 0 | 0.0001586 | 2.53E-05 | 0 | 0 | 0.00021016 | 0 | 0 | 0 | 4.70E-05 | 0.00027684 | 0.00116413 | 0.00175472 |
| k__Bacteria;p__Proteobacteria;c__Alphaproteobacteria;o__Rhodobacterales;f__Rhodobacteraceae;g__Donghicola | | 0 | 0 | 0 | 0 | 0 | 0 | 0 | 0 | 7.92E-06 | 0 | 0 | 0 | 0 | 0 | 0 |
| k__Bacteria;p__Proteobacteria;c__Alphaproteobacteria;o__Rhodobacterales;f__Rhodobacteraceae;g__Jannaschia | | 0 | 0 | 0 | 0 | 0 | 0 | 0 | 0 | 4.75E-05 | 0 | 0 | 0 | 0 | 3.28E-05 | 0 |
| k__Bacteria;p__Proteobacteria;c__Alphaproteobacteria;o__Rhodobacterales;f__Rhodobacteraceae;g__Loktanella | | 0 | 0 | 0 | 0.00013877 | 0 | 1.64E-05 | 0 | 8.41E-06 | 7.92E-06 | 0 | 0 | 0.0001879 | 0.00024223 | 8.20E-05 | 5.16E-05 |
| k__Bacteria;p__Proteobacteria;c__Alphaproteobacteria;o__Rhodobacterales;f__Rhodobacteraceae;g__Maribius | | 0 | 0 | 0 | 0 | 0 | 0 | 0 | 0 | 0 | 0 | 0 | 0 | 0 | 0 | 0 |
| k__Bacteria;p__Proteobacteria;c__Alphaproteobacteria;o__Rhodobacterales;f__Rhodobacteraceae;g__Marivita | | 0 | 0 | 0 | 0 | 0 | 0.00026305 | 0 | 0 | 0 | 0 | 0 | 0 | 0 | 0 | 0 |
| k__Bacteria;p__Proteobacteria;c__Alphaproteobacteria;o__Rhodobacterales;f__Rhodobacteraceae;g__Nautella | | 0 | 0 | 0 | 0 | 0 | 0 | 0 | 5.04E-05 | 0 | 0 | 0 | 0 | 0 | 0 | 0 |

| Taxon | | 1180 | 1182 | 1183 | 1203 | 1211 | 1242 | 6475 | 6476 | 6477 | 1220 | 1221 | 1248 | 2032 | 2033 | 2034 |
|---|---|---|---|---|---|---|---|---|---|---|---|---|---|---|---|---|
| | Copepod genus | Acartia | Acartia | Acartia | Acartia | Acartia | Acartia | Acartia | Acartia | Acartia | Acartia | Acartia | Acartia | Acartia | Acartia | Acartia |
| | Type of sample | FullGut | FullGut | FullGut | FullGut | FullGut | FullGut | FullGut | FullGut | FullGut | Starved | Starved | Starved | Starved | Starved | Starved |
| k__Bacteria;p__Proteobacteria;c__Alphaproteobacteria;o__Rhodobacterales;f__Rhodobacteraceae;g__Oceanicola | | 0 | 0 | 0 | 0 | 0 | 0 | 0 | 0 | 0 | 0 | 0 | 0 | 0 | 0 | 0 |
| k__Bacteria;p__Proteobacteria;c__Alphaproteobacteria;o__Rhodobacterales;f__Rhodobacteraceae;g__Octadecabacter | | 0.03922672 | 0.05010426 | 0.0074733 | 0.00085246 | 0.00116459 | 0.12924997 | 0.02010167 | 0.01361007 | 0.0348423 | 0.00018601 | 0.0077862 | 0.08282523 | 0.05759914 | 0.05274635 | 0.05212545 |
| k__Bacteria;p__Proteobacteria;c__Alphaproteobacteria;o__Rhodobacterales;f__Rhodobacteraceae;g__Paracoccus | | 0 | 0 | 0 | 0 | 0.00017722 | 0 | 0.00255333 | 0.00047917 | 0 | 0 | 0 | 0 | 0 | 0 | 0 |
| k__Bacteria;p__Proteobacteria;c__Alphaproteobacteria;o__Rhodobacterales;f__Rhodobacteraceae;g__Phaeobacter | | 0 | 0 | 0 | 0.00233932 | 0 | 0.01736157 | 0.00299436 | 0.00031945 | 0.00081544 | 0.00446429 | 0 | 0.01533244 | 0.000744 | 0.00054107 | 0.00051609 |
| k__Bacteria;p__Proteobacteria;c__Alphaproteobacteria;o__Rhodobacterales;f__Rhodobacteraceae;g__Pseudoroseobacter | | 0 | 0 | 0 | 0 | 0 | 0 | 0 | 0.00032785 | 6.33E-05 | 0 | 0 | 1.88E-05 | 3.46E-05 | 0.00013117 | 0.00018923 |
| k__Bacteria;p__Proteobacteria;c__Alphaproteobacteria;o__Rhodobacterales;f__Rhodobacteraceae;g__Pseudoruegeria | | 0.00043281 | 0.00068514 | 0 | 0.00013877 | 0 | 0 | 0.003064 | 0.0001261 | 0.00052252 | 0 | 0 | 6.58E-05 | 5.19E-05 | 8.20E-05 | 0.00010322 |
| k__Bacteria;p__Proteobacteria;c__Alphaproteobacteria;o__Rhodobacterales;f__Rhodobacteraceae;g__Rhodobacter | | 0.00047609 | 0 | 0 | 0 | 0 | 0 | 0 | 0.00033626 | 0 | 0 | 0 | 0 | 0 | 0 | 0 |
| k__Bacteria;p__Proteobacteria;c__Alphaproteobacteria;o__Rhodobacterales;f__Rhodobacteraceae;g__Roseicyclus | | 0 | 0 | 0 | 0 | 0 | 0 | 0 | 0 | 0 | 0 | 0 | 0 | 0 | 4.92E-05 | 0 |
| k__Bacteria;p__Proteobacteria;c__Alphaproteobacteria;o__Rhodobacterales;f__Rhodobacteraceae;g__Roseobacter | | 0 | 0 | 0 | 0 | 0 | 0 | 0 | 0 | 0 | 0 | 0 | 0 | 0 | 0 | 0 |
| k__Bacteria;p__Proteobacteria;c__Alphaproteobacteria;o__Rhodobacterales;f__Rhodobacteraceae;g__Rubellimicrobium | | 0 | 0 | 0 | 0 | 0 | 0 | 0 | 6.73E-05 | 0.00020584 | 0.00102307 | 0 | 0 | 0 | 0 | 0 |
| k__Bacteria;p__Proteobacteria;c__Alphaproteobacteria;o__Rhodobacterales;f__Rhodobacteraceae;g__Shimia | | 0 | 0 | 0 | 0 | 0 | 0 | 2.32E-05 | 0 | 0 | 0 | 0 | 0 | 0 | 0 | 0 |
| k__Bacteria;p__Proteobacteria;c__Alphaproteobacteria;o__Rhodobacterales;f__Rhodobacteraceae;g__Sulfitobacter | | 0 | 0 | 0 | 0 | 0 | 0.00223596 | 0 | 0.00618716 | 0.03238014 | 0 | 0 | 0.00047914 | 0.0030971 | 0.00439416 | 0.00235683 |
| k__Bacteria;p__Proteobacteria;c__Alphaproteobacteria;o__Rhodobacterales;f__Rhodobacteraceae;g__Thalassobacter | | 0 | 0 | 0 | 0 | 0 | 0 | 0 | 0 | 7.92E-06 | 0 | 0 | 0 | 0 | 1.64E-05 | 0 |
| k__Bacteria;p__Proteobacteria;c__Alphaproteobacteria;o__Rhodobacterales;f__Rhodobacteraceae;g__Thalassobius | | 0 | 0 | 0 | 0 | 0 | 0 | 9.28E-05 | 4.20E-05 | 0 | 0 | 0 | 0.00025366 | 0.00039795 | 0.00029513 | 0.00029245 |
| k__Bacteria;p__Proteobacteria;c__Alphaproteobacteria;o__Rhodobacterales;f__Rhodobacteraceae;g__Tropicibacter | | 0 | 0 | 0 | 0 | 0 | 0 | 0 | 1.68E-05 | 1.58E-05 | 0 | 0 | 0 | 1.73E-05 | 1.64E-05 | 1.72E-05 |
| k__Bacteria;p__Proteobacteria;c__Alphaproteobacteria;o__Rhodospirillales;f__;g__ | | 0 | 0 | 0 | 0 | 0 | 0 | 0 | 0.00014291 | 0 | 0 | 0 | 0 | 0 | 0 | 0 |
| k__Bacteria;p__Proteobacteria;c__Alphaproteobacteria;o__Rhodospirillales;f__Acetobacteraceae;g__ | | 4.33E-05 | 2.98E-05 | 0 | 0 | 0.00032912 | 0.00026305 | 0 | 5.04E-05 | 1.58E-05 | 0 | 0 | 0 | 3.46E-05 | 0 | 0 |
| k__Bacteria;p__Proteobacteria;c__Alphaproteobacteria;o__Rhodospirillales;f__Acetobacteraceae;g__Roseococcus | | 0 | 0.00038725 | 0 | 0 | 0 | 0 | 0 | 0 | 0 | 0 | 0 | 0 | 0 | 0 | 0 |
| k__Bacteria;p__Proteobacteria;c__Alphaproteobacteria;o__Rhodospirillales;f__Rhodospirillaceae;g__ | | 0.00109644 | 0.00204051 | 0 | 0 | 0 | 0.00180028 | 0 | 0.00052961 | 0.00103711 | 0 | 0 | 0.00178503 | 0.00371998 | 0.0025578 | 0.00414595 |
| k__Bacteria;p__Proteobacteria;c__Alphaproteobacteria;o__Rhodospirillales;f__Rhodospirillaceae;g__Skermanella | | 0 | 0 | 0 | 0 | 0 | 0 | 0 | 0 | 0 | 0 | 0 | 0 | 0 | 0 | 0 |
| k__Bacteria;p__Proteobacteria;c__Alphaproteobacteria;o__Rickettsiales;f__;g__ | | 0.00308735 | 0.00500447 | 0.0025846 | 0 | 0 | 0.00014797 | 0 | 5.88E-05 | 0.00022959 | 0 | 0 | 0.00011274 | 0 | 4.92E-05 | 0.00027525 |
| k__Bacteria;p__Proteobacteria;c__Alphaproteobacteria;o__Rickettsiales;f__AEGEAN_112;g__ | | 0.00014427 | 0 | 0.00032057 | 0 | 0 | 0 | 0 | 0 | 0 | 0.0031622 | 0 | 0 | 0 | 0 | 0 |
| k__Bacteria;p__Proteobacteria;c__Alphaproteobacteria;o__Rickettsiales;f__Pelagibacteraceae;g__ | | 0.08995167 | 0.09414656 | 0.01885356 | 0.00017842 | 0.00650649 | 0.04126661 | 0.02439591 | 0.00404351 | 0.0104899 | 0.01320685 | 0.0770202 | 0.01360378 | 0.02083189 | 0.00605017 | 0.02329302 |
| k__Bacteria;p__Proteobacteria;c__Alphaproteobacteria;o__Rickettsiales;f__Rickettsiaceae;g__ | | 8.66E-05 | 0 | 0 | 0 | 0.04883668 | 0 | 0 | 0 | 0 | 0 | 0 | 0 | 0 | 0 | 0 |
| k__Bacteria;p__Proteobacteria;c__Alphaproteobacteria;o__Rickettsiales;f__mitochondria;g__ | | 2.89E-05 | 0 | 0 | 0 | 0 | 0 | 0 | 0 | 0 | 0 | 0 | 0.00169108 | 0 | 0 | 0 |
| k__Bacteria;p__Proteobacteria;c__Alphaproteobacteria;o__Rickettsiales;f__mitochondria;g__Carludovica | | 0 | 0 | 0 | 0 | 0 | 8.22E-06 | 0 | 0 | 0 | 0 | 0 | 0 | 0 | 0 | 0 |
| k__Bacteria;p__Proteobacteria;c__Alphaproteobacteria;o__Rickettsiales;f__mitochondria;g__Citrullus | | 0 | 0 | 0 | 0 | 0 | 3.29E-05 | 0 | 0 | 0 | 0 | 0 | 0 | 0 | 0 | 0 |
| k__Bacteria;p__Proteobacteria;c__Alphaproteobacteria;o__Rickettsiales;f__mitochondria;g__Lupinus | | 0 | 0 | 0 | 0 | 0 | 8.22E-06 | 0 | 0 | 0 | 0 | 0 | 0 | 0 | 0 | 0 |
| k__Bacteria;p__Proteobacteria;c__Alphaproteobacteria;o__Rickettsiales;f__mitochondria;g__Nageia | | 0 | 0 | 0 | 0 | 0 | 0 | 0 | 0 | 0 | 0 | 0 | 0 | 0 | 0 | 0 |
| k__Bacteria;p__Proteobacteria;c__Alphaproteobacteria;o__Rickettsiales;f__mitochondria;g__Nelumbo | | 0 | 0 | 0 | 0 | 0 | 0 | 0 | 0 | 0 | 0 | 0 | 0 | 0 | 0 | 0 |
| k__Bacteria;p__Proteobacteria;c__Alphaproteobacteria;o__Rickettsiales;f__mitochondria;g__Oenothera | | 0 | 0 | 0 | 0 | 0 | 0.001044 | 0 | 0 | 0 | 0 | 0 | 0 | 0 | 0 | 1.72E-05 |
| k__Bacteria;p__Proteobacteria;c__Alphaproteobacteria;o__Rickettsiales;f__mitochondria;g__Phyllocladus | | 0 | 0 | 0 | 0 | 0 | 0 | 0 | 0 | 0 | 0 | 0 | 0 | 0 | 0 | 0 |
| k__Bacteria;p__Proteobacteria;c__Alphaproteobacteria;o__Rickettsiales;f__mitochondria;g__Zea | | 0 | 0 | 0 | 0 | 0 | 0 | 0 | 0 | 0 | 0 | 0 | 0 | 0 | 0 | 0 |
| k__Bacteria;p__Proteobacteria;c__Alphaproteobacteria;o__Sphingomonadales;f__;g__ | | 0 | 0 | 0 | 0 | 0 | 0 | 2.32E-05 | 0.00051279 | 0.00020584 | 0 | 0 | 2.82E-05 | 0 | 0 | 3.44E-05 |
| k__Bacteria;p__Proteobacteria;c__Alphaproteobacteria;o__Sphingomonadales;f__Erythrobacteraceae;g__ | | 0 | 0 | 0 | 0 | 0 | 0 | 0 | 0 | 0 | 0 | 0 | 0 | 0 | 0 | 0 |
| k__Bacteria;p__Proteobacteria;c__Alphaproteobacteria;o__Sphingomonadales;f__Erythrobacteraceae;g__Altererythrobacter | | 0 | 0 | 0 | 0 | 0 | 0 | 0 | 0 | 0 | 0 | 0 | 0 | 0 | 0 | 0 |
| k__Bacteria;p__Proteobacteria;c__Alphaproteobacteria;o__Sphingomonadales;f__Erythrobacteraceae;g__Erythrobacter | | 0 | 0 | 0 | 0 | 0 | 0 | 0 | 0 | 0 | 0 | 0 | 0 | 0 | 0 | 0 |
| k__Bacteria;p__Proteobacteria;c__Alphaproteobacteria;o__Sphingomonadales;f__Erythrobacteraceae;g__Lutibacterium | | 0 | 0 | 0 | 0 | 0 | 0 | 0 | 0 | 0 | 0 | 0 | 0 | 0 | 0 | 0 |
| k__Bacteria;p__Proteobacteria;c__Alphaproteobacteria;o__Sphingomonadales;f__Sphingomonadaceae;g__ | | 1.44E-05 | 0.0002681 | 0.00022039 | 7.93E-05 | 7.60E-05 | 0.00031238 | 0.00471205 | 0.00010088 | 0.00016625 | 0.00055804 | 0 | 0 | 1.73E-05 | 0 | 0 |
| k__Bacteria;p__Proteobacteria;c__Alphaproteobacteria;o__Sphingomonadales;f__Sphingomonadaceae;g__Kaistobacter | | 0 | 0 | 0 | 0 | 0 | 0 | 0 | 0 | 0 | 0 | 0 | 0 | 0 | 0 | 3.44E-05 |
| k__Bacteria;p__Proteobacteria;c__Alphaproteobacteria;o__Sphingomonadales;f__Sphingomonadaceae;g__Novosphingobium | | 8.66E-05 | 0.00017873 | 0 | 0 | 0 | 0 | 0.00399248 | 0 | 0 | 0.00539435 | 0 | 0.00013153 | 0 | 0 | 0 |
| k__Bacteria;p__Proteobacteria;c__Alphaproteobacteria;o__Sphingomonadales;f__Sphingomonadaceae;g__Sphingobium | | 0 | 0 | 0 | 0 | 0 | 0 | 0 | 0 | 0 | 0 | 0 | 2.82E-05 | 0 | 0 | 0 |
| k__Bacteria;p__Proteobacteria;c__Alphaproteobacteria;o__Sphingomonadales;f__Sphingomonadaceae;g__Sphingomonas | | 0.00046166 | 0.00072982 | 0.00034061 | 0.00011895 | 0.00316464 | 0.00046034 | 0.00352824 | 0.00026901 | 0.00031668 | 0.01190476 | 0.01599327 | 7.52E-05 | 1.73E-05 | 0.00013117 | 0.00013762 |
| k__Bacteria;p__Proteobacteria;c__Alphaproteobacteria;o__Sphingomonadales;f__Sphingomonadaceae;g__Sphingopyxis | | 0 | 0 | 0 | 0 | 0 | 0 | 0 | 0 | 9.50E-05 | 0 | 0 | 4.70E-05 | 0 | 0 | 0 |
| k__Bacteria;p__Proteobacteria;c__Alphaproteobacteria;o__Sphingomonadales;f__Sphingomonadaceae;g__Zymomonas | | 0 | 0 | 0 | 0 | 0 | 0 | 0 | 0 | 0 | 0 | 0 | 0 | 0 | 0 | 0 |
| k__Bacteria;p__Proteobacteria;c__Betaproteobacteria;o__;f__;g__ | | 0 | 0 | 0.00022039 | 0 | 0 | 0.00195646 | 0.00236763 | 0.00031945 | 0.00057002 | 0.00344122 | 0 | 0.00030064 | 0.00193785 | 0.00021315 | 0.00189234 |
| k__Bacteria;p__Proteobacteria;c__Betaproteobacteria;o__Burkholderiales;Comamonadaceae;g__ | | 0.00030296 | 0.00089366 | 0.00024043 | 1.98E-05 | 0.00027849 | 0.00069052 | 0.00285509 | 0.00063049 | 0.00102128 | 0.00530134 | 0.00189394 | 5.64E-05 | 0 | 6.56E-05 | 3.44E-05 |
| k__Bacteria;p__Proteobacteria;c__Betaproteobacteria;o__Burkholderiales;f__;g__ | | 0.00043281 | 0.00081918 | 0.00108193 | 0.0001586 | 0.00093673 | 0.00110154 | 0 | 2.52E-05 | 0.00095795 | 0.00762649 | 0.00757576 | 2.82E-05 | 1.73E-05 | 0 | 0 |
| k__Bacteria;p__Proteobacteria;c__Betaproteobacteria;o__Burkholderiales;f__Alcaligenaceae;g__ | | 0 | 0 | 0 | 0 | 0 | 0.00022195 | 0 | 0 | 0 | 0 | 0 | 0 | 0 | 3.28E-05 | 0 |
| k__Bacteria;p__Proteobacteria;c__Betaproteobacteria;o__Burkholderiales;f__Alcaligenaceae;g__Achromobacter | | 0.00010099 | 0 | 0.00024043 | 3.96E-05 | 0.00113927 | 0.00018085 | 0 | 0 | 0 | 0 | 0.00547138 | 0 | 0 | 0 | 0 |
| k__Bacteria;p__Proteobacteria;c__Betaproteobacteria;o__Burkholderiales;f__Alcaligenaceae;g__Sutterella | | 0 | 8.94E-05 | 0 | 0 | 0 | 0 | 0 | 0 | 0 | 0 | 0 | 0 | 0 | 0 | 0 |
| k__Bacteria;p__Proteobacteria;c__Betaproteobacteria;o__Burkholderiales;f__Burkholderiaceae;g__ | | 5.77E-05 | 0.00013405 | 0.00034061 | 0 | 0.00050634 | 0.00114264 | 0 | 0 | 0 | 0.00325521 | 0.00673401 | 0 | 0 | 0 | 0 |
| k__Bacteria;p__Proteobacteria;c__Betaproteobacteria;o__Burkholderiales;f__Burkholderiaceae;g__Burkholderia | | 0 | 2.98E-05 | 0 | 0 | 7.60E-05 | 0 | 0 | 0 | 0.00093419 | 9.30E-05 | 0 | 0 | 0 | 0 | 0 |
| k__Bacteria;p__Proteobacteria;c__Betaproteobacteria;o__Burkholderiales;f__Burkholderiaceae;g__Lautropia | | 0 | 0 | 0 | 0 | 0 | 0 | 0 | 0 | 0 | 0 | 0 | 0 | 0 | 0 | 1.72E-05 |
| k__Bacteria;p__Proteobacteria;c__Betaproteobacteria;o__Burkholderiales;f__Comamonadaceae;g__Acidovorax | | 0 | 0 | 0 | 0 | 0 | 0 | 0 | 5.04E-05 | 0 | 0 | 0 | 0 | 0 | 0 | 0 |
| k__Bacteria;p__Proteobacteria;c__Betaproteobacteria;o__Burkholderiales;f__Comamonadaceae;g__Comamonas | | 0 | 0.00281501 | 0 | 0 | 0 | 0 | 0 | 0 | 0.00010292 | 0 | 0 | 0 | 0 | 0 | 0 |
| k__Bacteria;p__Proteobacteria;c__Betaproteobacteria;o__Burkholderiales;f__Comamonadaceae;g__Curvibacter | | 1.44E-05 | 0 | 4.01E-05 | 0 | 0 | 0.00013975 | 0 | 0.00010088 | 0.00015834 | 0 | 0 | 9.39E-05 | 0 | 0 | 0 |
| k__Bacteria;p__Proteobacteria;c__Betaproteobacteria;o__Burkholderiales;f__Comamonadaceae;g__Delftia | | 0.00038953 | 0.0005064 | 0.00072128 | 0.00027755 | 0.00182283 | 0.00132349 | 0 | 0 | 0 | 0.01106771 | 0.01767677 | 0 | 0 | 0 | 0 |
| k__Bacteria;p__Proteobacteria;c__Betaproteobacteria;o__Burkholderiales;f__Comamonadaceae;g__Hydrogenophaga | | 0 | 0.00105749 | 0 | 0 | 0 | 0 | 0 | 0 | 0 | 0 | 0 | 0 | 0 | 0 | 0 |
| k__Bacteria;p__Proteobacteria;c__Betaproteobacteria;o__Burkholderiales;f__Comamonadaceae;g__Leptothrix | | 0 | 0 | 0 | 0 | 0 | 0 | 0 | 0 | 0 | 9.30E-05 | 0.00084175 | 0 | 0 | 0 | 0 |
| k__Bacteria;p__Proteobacteria;c__Betaproteobacteria;o__Burkholderiales;f__Comamonadaceae;g__Limnohabitans | | 0 | 0 | 0 | 0 | 0 | 0 | 0 | 0 | 0 | 0.00018601 | 0 | 0 | 0 | 0 | 0 |
| k__Bacteria;p__Proteobacteria;c__Betaproteobacteria;o__Burkholderiales;f__Comamonadaceae;g__Methylibium | | 0 | 0 | 0 | 0 | 0 | 0 | 0 | 0 | 0 | 0 | 0 | 0 | 0 | 0 | 0 |
| k__Bacteria;p__Proteobacteria;c__Betaproteobacteria;o__Burkholderiales;f__Comamonadaceae;g__Polaromonas | | 0 | 0 | 0 | 0 | 0 | 0 | 0 | 0.00015132 | 0 | 0 | 0 | 0 | 0 | 0 | 0 |
| k__Bacteria;p__Proteobacteria;c__Betaproteobacteria;o__Burkholderiales;f__Comamonadaceae;g__RS62 | | 0.00030296 | 0 | 0 | 0 | 0 | 0.00052611 | 0 | 0 | 0.00022167 | 0 | 0 | 0 | 6.92E-05 | 0 | 0.00010322 |
| k__Bacteria;p__Proteobacteria;c__Betaproteobacteria;o__Burkholderiales;f__Comamonadaceae;g__Rhodoferax | | 0 | 0 | 0 | 0 | 0 | 0 | 0 | 0 | 0 | 0 | 0 | 0 | 0 | 0 | 0 |
| k__Bacteria;p__Proteobacteria;c__Betaproteobacteria;o__Burkholderiales;f__Comamonadaceae;g__Roseateles | | 0 | 0 | 0 | 0 | 0 | 0 | 0 | 0 | 0 | 0 | 0.00021044 | 0 | 0 | 0 | 0 |
| k__Bacteria;p__Proteobacteria;c__Betaproteobacteria;o__Burkholderiales;f__Comamonadaceae;g__Schlegelella | | 0 | 0 | 0 | 7.93E-05 | 0 | 0 | 0 | 0 | 0 | 0 | 0 | 3.76E-05 | 0 | 0 | 0 |
| k__Bacteria;p__Proteobacteria;c__Betaproteobacteria;o__Burkholderiales;f__Comamonadaceae;g__Tepidimonas | | 0 | 0 | 0 | 0 | 0 | 0 | 0 | 0 | 0 | 0 | 0 | 0 | 0 | 0 | 0 |
| k__Bacteria;p__Proteobacteria;c__Betaproteobacteria;o__Burkholderiales;f__Comamonadaceae;g__Variovorax | | 0.00011542 | 0 | 6.01E-05 | 0 | 0 | 0 | 0.00097491 | 0 | 0 | 0 | 0 | 0 | 0 | 0 | 0 |
| k__Bacteria;p__Proteobacteria;c__Betaproteobacteria;o__Burkholderiales;f__Oxalobacteraceae;g__ | | 0 | 4.47E-05 | 0 | 0 | 0.00020254 | 0.00061653 | 0.0021123 | 3.36E-05 | 0.00064127 | 0.00502232 | 0.00021044 | 9.39E-06 | 0 | 1.64E-05 | 0 |
| k__Bacteria;p__Proteobacteria;c__Betaproteobacteria;o__Burkholderiales;f__Oxalobacteraceae;g__Cupriavidus | | 0 | 0 | 0 | 0 | 0 | 0 | 0 | 0 | 0 | 0 | 0 | 0 | 0 | 3.28E-05 | 0 |
| k__Bacteria;p__Proteobacteria;c__Betaproteobacteria;o__Burkholderiales;f__Oxalobacteraceae;g__Herbaspirillum | | 0 | 1.49E-05 | 0 | 0 | 0 | 0 | 0 | 0 | 0.0001425 | 0 | 0 | 0 | 0 | 0 | 0 |
| k__Bacteria;p__Proteobacteria;c__Betaproteobacteria;o__Burkholderiales;f__Oxalobacteraceae;g__Janthinobacterium | | 0 | 0 | 0 | 0 | 0 | 0.00026305 | 0.00280866 | 0 | 0 | 0 | 0.00042088 | 0 | 0 | 0 | 0 |
| k__Bacteria;p__Proteobacteria;c__Betaproteobacteria;o__Burkholderiales;f__Oxalobacteraceae;g__Massilia | | 0 | 0 | 0 | 0 | 0 | 0 | 2.32E-05 | 0 | 0 | 0 | 0.01746633 | 0 | 0 | 0 | 0 |

| Taxon | | 1180 | 1182 | 1183 | 1203 | 1211 | 1242 | 6475 | 6476 | 6477 | 1220 | 1221 | 1248 | 2032 | 2033 | 2034 |
|---|---|---|---|---|---|---|---|---|---|---|---|---|---|---|---|---|
| | Copepod genus | Acartia | Acartia | Acartia | Acartia | Acartia | Acartia | Acartia | Acartia | Acartia | Acartia | Acartia | Acartia | Acartia | Acartia | Acartia |
| | Type of sample | FullGut | FullGut | FullGut | FullGut | FullGut | FullGut | FullGut | FullGut | FullGut | Starved | Starved | Starved | Starved | Starved | Starved |
| k__Bacteria;p__Proteobacteria;c__Betaproteobacteria;o__Burkholderiales;f__Oxalobacteraceae;g__Polynucleobacter | | 0 | 0 | 0 | 0 | 0 | 0 | 0 | 0 | 0 | 0 | 0 | 0 | 0 | 0 | 0 |
| k__Bacteria;p__Proteobacteria;c__Betaproteobacteria;o__Burkholderiales;f__Oxalobacteraceae;g__Ralstonia | | 8.66E-05 | 4.47E-05 | 0.00020036 | 0.00013877 | 0.00037976 | 0.00027127 | 0.00104454 | 2.52E-05 | 0.00061752 | 0.00176711 | 0.00063131 | 2.82E-05 | 0 | 1.64E-05 | 1.72E-05 |
| k__Bacteria;p__Proteobacteria;c__Betaproteobacteria;o__Ellin6067;f__;g__ | | 0 | 0 | 0 | 0 | 0 | 0 | 0 | 0 | 0 | 0 | 0 | 0 | 0 | 0 | 0 |
| k__Bacteria;p__Proteobacteria;c__Betaproteobacteria;o__Methylophilales;f__Methylophilaceae;g__ | | 0.00190435 | 0.00144474 | 0.00026046 | 0 | 0 | 0.00512955 | 2.32E-05 | 0.00046236 | 0.00167838 | 0 | 0 | 0.00039458 | 0.00089972 | 0.00031153 | 0.00086016 |
| k__Bacteria;p__Proteobacteria;c__Betaproteobacteria;o__Methylophilales;f__Methylophilaceae;g__Methylotenera | | 0 | 0 | 0 | 0 | 0 | 0 | 0 | 0 | 0.00015042 | 0 | 0 | 0 | 0 | 0 | 0 |
| k__Bacteria;p__Proteobacteria;c__Betaproteobacteria;o__Neisseriales;f__Neisseriaceae;g__ | | 0.00067806 | 0.00038725 | 0 | 0 | 0.00037976 | 0 | 0.002298 | 8.41E-05 | 0.00016625 | 0.00483631 | 0.00210438 | 2.82E-05 | 0 | 1.64E-05 | 0 |
| k__Bacteria;p__Proteobacteria;c__Betaproteobacteria;o__Neisseriales;f__Neisseriaceae;g__Kingella | | 0 | 0 | 0 | 0 | 0 | 0 | 0 | 0 | 0.00023751 | 0 | 0.00589226 | 0 | 0 | 0 | 0 |
| k__Bacteria;p__Proteobacteria;c__Betaproteobacteria;o__Neisseriales;f__Neisseriaceae;g__Microvirgula | | 0 | 0 | 0 | 0 | 0 | 0 | 0 | 0 | 0 | 0 | 0 | 0 | 0 | 0 | 0 |
| k__Bacteria;p__Proteobacteria;c__Betaproteobacteria;o__Neisseriales;f__Neisseriaceae;g__Neisseria | | 0 | 0.00028299 | 0.00022039 | 0 | 0 | 0 | 0.0019266 | 0 | 0.00049876 | 0.00325521 | 0 | 0 | 0 | 0 | 3.44E-05 |
| k__Bacteria;p__Proteobacteria;c__Betaproteobacteria;o__Nitrosomonadales;f__Nitrosomonadaceae;g__ | | 0 | 0 | 0 | 0 | 0 | 0 | 0 | 0 | 0 | 0 | 0 | 0 | 0 | 0 | 0 |
| k__Bacteria;p__Proteobacteria;c__Betaproteobacteria;o__Procabacteriales;f__Procabacteriaceae;g__ | | 0 | 0 | 0.00012021 | 0 | 0 | 0 | 0 | 0 | 0 | 0 | 0 | 0 | 0 | 0 | 0 |
| k__Bacteria;p__Proteobacteria;c__Betaproteobacteria;o__Rhodocyclales;f__Rhodocyclaceae;g__ | | 0.00502056 | 0.0067471 | 0.00194346 | 0 | 0 | 0.00499803 | 0.0019266 | 0.00030263 | 0.00188422 | 0 | 0.00736532 | 0.00328821 | 0.00508686 | 0.00365634 | 0.00576304 |
| k__Bacteria;p__Proteobacteria;c__Betaproteobacteria;o__Rhodocyclales;f__Rhodocyclaceae;g__Dechloromonas | | 0 | 0 | 0 | 0 | 0 | 0 | 0 | 0 | 0 | 0 | 0 | 0 | 0 | 0 | 0 |
| k__Bacteria;p__Proteobacteria;c__Betaproteobacteria;o__Rhodocyclales;f__Rhodocyclaceae;g__Hydrogenophilus | | 0 | 0 | 0 | 0 | 0 | 0.00051789 | 0 | 0 | 0 | 0.00297619 | 0 | 0 | 0 | 0 | 0 |
| k__Bacteria;p__Proteobacteria;c__Betaproteobacteria;o__Rhodocyclales;f__Rhodocyclaceae;g__Methyloversatilis | | 0 | 0 | 0 | 0 | 0 | 0 | 0 | 0 | 0 | 0 | 0 | 0 | 0 | 0 | 0 |
| k__Bacteria;p__Proteobacteria;c__Betaproteobacteria;o__Rhodocyclales;f__Rhodocyclaceae;g__Propionivibrio | | 0 | 0 | 0 | 0 | 0 | 0 | 0 | 8.41E-05 | 0 | 0 | 0 | 0 | 0 | 0 | 0 |
| k__Bacteria;p__Proteobacteria;c__Betaproteobacteria;o__Rhodocyclales;f__Rhodocyclaceae;g__Uliginosibacterium | | 0 | 0 | 0 | 0 | 0 | 0 | 0 | 0 | 0 | 0 | 0 | 0 | 0 | 0 | 0 |
| k__Bacteria;p__Proteobacteria;c__Betaproteobacteria;o__Rhodocyclales;f__Rhodocyclaceae;g__Zoogloea | | 0 | 0 | 0 | 0 | 0 | 0 | 0 | 0 | 0 | 0 | 0 | 6.58E-05 | 0 | 0 | 0 |
| k__Bacteria;p__Proteobacteria;c__Betaproteobacteria;o__SC-I-84;f__;g__ | | 0 | 0 | 0 | 0 | 0 | 0 | 0 | 0 | 0 | 0 | 0 | 0 | 0 | 0 | 0 |
| k__Bacteria;p__Proteobacteria;c__Betaproteobacteria;o__Tremblayales;f__;g__ | | 0 | 0 | 0 | 0 | 0 | 0 | 0 | 0 | 0 | 0 | 0 | 0 | 0 | 0 | 0 |
| k__Bacteria;p__Proteobacteria;c__Deltaproteobacteria;o__;f__;g__ | | 0 | 0 | 0 | 0 | 0 | 0 | 0 | 0 | 0 | 0 | 0 | 0 | 0 | 0 | 0 |
| k__Bacteria;p__Proteobacteria;c__Deltaproteobacteria;o__Bdellovibrionales;f__Bacteriovoracaceae;g__ | | 0 | 0 | 0 | 0 | 0 | 0 | 0 | 0 | 0 | 0 | 0 | 1.88E-05 | 5.19E-05 | 3.28E-05 | 6.88E-05 |
| k__Bacteria;p__Proteobacteria;c__Deltaproteobacteria;o__Bdellovibrionales;f__Bacteriovoracaceae;g__Bacteriovorax | | 0 | 0 | 0 | 0 | 0 | 0.00083848 | 0 | 3.36E-05 | 0.00011875 | 0 | 0 | 0.00060127 | 0 | 0 | 3.44E-05 |
| k__Bacteria;p__Proteobacteria;c__Deltaproteobacteria;o__Bdellovibrionales;f__Bdellovibrionaceae;g__Bdellovibrio | | 0 | 7.45E-05 | 0 | 0 | 0 | 0.00014797 | 0 | 0 | 0 | 0 | 0 | 0 | 0 | 0 | 0 |
| k__Bacteria;p__Proteobacteria;c__Deltaproteobacteria;o__Desulfarculales;f__Desulfarculaceae;g__ | | 0 | 0 | 0 | 0 | 0 | 0 | 0 | 0 | 0 | 0 | 0 | 0 | 0 | 0 | 0 |
| k__Bacteria;p__Proteobacteria;c__Deltaproteobacteria;o__Desulfobacterales;f__Desulfobacteraceae;g__ | | 0 | 0 | 0 | 0 | 0 | 0 | 0 | 0 | 0 | 0 | 0 | 0 | 0 | 0 | 0 |
| k__Bacteria;p__Proteobacteria;c__Deltaproteobacteria;o__Desulfobacterales;f__Desulfobacteraceae;g__Desulfococcus | | 0 | 0 | 0 | 0 | 0 | 0 | 0 | 0 | 0 | 0 | 0 | 0 | 0 | 0 | 0 |
| k__Bacteria;p__Proteobacteria;c__Deltaproteobacteria;o__Desulfobacterales;f__Desulfobacteraceae;g__Desulfofrigus | | 0 | 0 | 0 | 0 | 0 | 0 | 0 | 0 | 0 | 0 | 0 | 0 | 0 | 0 | 0 |
| k__Bacteria;p__Proteobacteria;c__Deltaproteobacteria;o__Desulfobacterales;f__Desulfobacteraceae;g__Desulfosarcina | | 0 | 0 | 0 | 0 | 0 | 0 | 0 | 0 | 0 | 0 | 0 | 0 | 0 | 0 | 0 |
| k__Bacteria;p__Proteobacteria;c__Deltaproteobacteria;o__Desulfobacterales;f__Desulfobulbaceae;g__ | | 0 | 0 | 0 | 0 | 0 | 0 | 0 | 0 | 0 | 0 | 0 | 0 | 0 | 0 | 0 |
| k__Bacteria;p__Proteobacteria;c__Deltaproteobacteria;o__Desulfobacterales;f__Nitrospinaceae;g__Nitrospina | | 0.00017312 | 0 | 0 | 0 | 0 | 0 | 0 | 0 | 0 | 0 | 0 | 0 | 0 | 0 | 0 |
| k__Bacteria;p__Proteobacteria;c__Deltaproteobacteria;o__Desulfovibrionales;f__Desulfovibrionaceae;g__ | | 0 | 0 | 0 | 0 | 0 | 0 | 0 | 0 | 0 | 0 | 0 | 0 | 0 | 0 | 0 |
| k__Bacteria;p__Proteobacteria;c__Deltaproteobacteria;o__Desulfovibrionales;f__Desulfovibrionaceae;g__Desulfovibrio | | 0 | 0 | 0 | 0 | 0 | 0 | 0 | 0 | 0 | 0 | 0 | 0 | 0 | 0 | 0 |
| k__Bacteria;p__Proteobacteria;c__Deltaproteobacteria;o__Desulfuromonadales;f__Desulfuromonadaceae;g__ | | 0 | 0 | 0 | 0 | 0 | 0 | 0 | 0 | 0 | 0 | 0 | 0 | 0 | 0 | 0 |
| k__Bacteria;p__Proteobacteria;c__Deltaproteobacteria;o__Desulfuromonadales;f__Desulfuromonadaceae;g__Desulfuromusa | | 0 | 0 | 0 | 0 | 0 | 0 | 0 | 0 | 0 | 0 | 0 | 0 | 0 | 0 | 0 |
| k__Bacteria;p__Proteobacteria;c__Deltaproteobacteria;o__FAC87;f__;g__ | | 0 | 0 | 0 | 0 | 0 | 0.00015619 | 0 | 0 | 0 | 0 | 0 | 0 | 0 | 0 | 0 |
| k__Bacteria;p__Proteobacteria;c__Deltaproteobacteria;o__MIZ46;f__;g__ | | 0 | 0 | 0 | 0 | 0 | 0.00018907 | 0 | 0 | 0 | 0 | 0 | 0 | 0 | 0 | 0 |
| k__Bacteria;p__Proteobacteria;c__Deltaproteobacteria;o__Myxococcales;f__;g__ | | 0 | 0 | 0 | 1.98E-05 | 0 | 0.00016441 | 0.00157842 | 0 | 0 | 0 | 0 | 0 | 0 | 0 | 0 |
| k__Bacteria;p__Proteobacteria;c__Deltaproteobacteria;o__Myxococcales;f__0319-6G20;g__ | | 0 | 0 | 0 | 0 | 0.00053166 | 0 | 0 | 0 | 0 | 0 | 0 | 0 | 0 | 0 | 0 |
| k__Bacteria;p__Proteobacteria;c__Deltaproteobacteria;o__Myxococcales;f__OM27;g__ | | 0 | 0 | 0 | 5.95E-05 | 0 | 0 | 0 | 0 | 0 | 0 | 0 | 0 | 0 | 0 | 1.72E-05 |
| k__Bacteria;p__Proteobacteria;c__Deltaproteobacteria;o__NB1-j;f__;g__ | | 0 | 0 | 0 | 0 | 0 | 0 | 0 | 0 | 0 | 0 | 0 | 0 | 0 | 0 | 0 |
| k__Bacteria;p__Proteobacteria;c__Deltaproteobacteria;o__NB1-j;f__JTB38;g__ | | 0 | 0 | 0 | 0 | 0 | 0 | 0 | 0 | 0 | 0 | 0 | 0 | 0 | 0 | 0 |
| k__Bacteria;p__Proteobacteria;c__Deltaproteobacteria;o__PB19;f__;g__ | | 0 | 0 | 0 | 0 | 0 | 0 | 0 | 0.00021857 | 0 | 0 | 0 | 0 | 0 | 0 | 0 |
| k__Bacteria;p__Proteobacteria;c__Deltaproteobacteria;o__Sva0853;f__;g__ | | 0 | 0 | 0 | 0 | 0 | 0 | 0 | 0 | 0 | 0 | 0 | 0 | 0 | 0 | 0 |
| k__Bacteria;p__Proteobacteria;c__Deltaproteobacteria;o__Sva0853;f__S25_1238;g__ | | 0 | 0 | 0 | 0 | 0 | 0 | 0 | 0 | 0 | 0 | 0 | 0 | 0 | 0 | 0 |
| k__Bacteria;p__Proteobacteria;c__Deltaproteobacteria;o__Sva0853;f__SAR324;g__ | | 0 | 0 | 0.00010018 | 0 | 0 | 0 | 0 | 0 | 0 | 0 | 0 | 0 | 0 | 0 | 0 |
| k__Bacteria;p__Proteobacteria;c__Deltaproteobacteria;o__Syntrophobacterales;f__Syntrophobacteraceae;g__ | | 0 | 0 | 0 | 0 | 0 | 0 | 0 | 0 | 0 | 0 | 0 | 0 | 0 | 0 | 0 |
| k__Bacteria;p__Proteobacteria;c__Epsilonproteobacteria;o__Campylobacterales;f__Campylobacteraceae;g__Arcobacter | | 0.00158696 | 0.00087876 | 0.00108193 | 0 | 0 | 0.00101933 | 0 | 0 | 0 | 0 | 0 | 0 | 0.00010381 | 4.92E-05 | 3.44E-05 |
| k__Bacteria;p__Proteobacteria;c__Epsilonproteobacteria;o__Campylobacterales;f__Campylobacteraceae;g__Campylobacter | | 0 | 0 | 0 | 0.0001586 | 0 | 0 | 0 | 0 | 0 | 0 | 0 | 0 | 0 | 0 | 0 |
| k__Bacteria;p__Proteobacteria;c__Epsilonproteobacteria;o__Campylobacterales;f__Campylobacteraceae;g__Sulfurospirillum | | 0 | 0 | 0 | 0 | 0 | 0 | 0 | 0 | 0 | 0 | 0 | 0 | 0 | 0 | 0 |
| k__Bacteria;p__Proteobacteria;c__Epsilonproteobacteria;o__Campylobacterales;f__Helicobacteraceae;g__ | | 0 | 0 | 0 | 0 | 0 | 0.00013975 | 0 | 0 | 0 | 0 | 0 | 0 | 0 | 0 | 0 |
| k__Bacteria;p__Proteobacteria;c__Epsilonproteobacteria;o__Campylobacterales;f__Helicobacteraceae;g__Sulfurimonas | | 0 | 0 | 0 | 0 | 0 | 0 | 0 | 0 | 0 | 0 | 0 | 0 | 0 | 0 | 0 |
| k__Bacteria;p__Proteobacteria;c__Epsilonproteobacteria;o__Campylobacterales;f__Helicobacteraceae;g__Wolinella | | 0 | 0 | 0 | 0 | 0 | 0 | 0 | 0 | 0 | 0 | 0 | 0 | 0 | 0 | 0 |
| k__Bacteria;p__Proteobacteria;c__Gammaproteobacteria;o__;f__;g__ | | 0.0018755 | 0.0010426 | 0 | 5.95E-05 | 0.00101268 | 0.00039458 | 0 | 0 | 0 | 0 | 0.00462963 | 0.00945125 | 0.00029414 | 0.00013117 | 0.00048169 |
| k__Bacteria;p__Proteobacteria;c__Gammaproteobacteria;o__Aeromonadales;f__Aeromonadaceae;g__ | | 0 | 0 | 0 | 0 | 0 | 0 | 0 | 0 | 0 | 0 | 0 | 0 | 0 | 0 | 0 |
| k__Bacteria;p__Proteobacteria;c__Gammaproteobacteria;o__Aeromonadales;f__Aeromonadaceae;g__Aeromonas | | 0 | 0 | 0 | 0 | 0 | 0 | 0 | 0 | 0 | 0 | 0 | 0 | 0 | 0 | 0 |
| k__Bacteria;p__Proteobacteria;c__Gammaproteobacteria;o__Aeromonadales;f__Aeromonadaceae;g__Tolumonas | | 0 | 0 | 0 | 0 | 0 | 0 | 0 | 0 | 0 | 0 | 0 | 0 | 0 | 0 | 0 |
| k__Bacteria;p__Proteobacteria;c__Gammaproteobacteria;o__Alteromonadales;f__;g__ | | 0.00027411 | 0 | 0 | 1.98E-05 | 0 | 0 | 0 | 0 | 0 | 0 | 0 | 0.00011274 | 0 | 0 | 3.44E-05 |
| k__Bacteria;p__Proteobacteria;c__Gammaproteobacteria;o__Alteromonadales;f__Alteromonadaceae;g__ | | 0.0001587 | 1.49E-05 | 0.00018032 | 0.00033702 | 0.00189878 | 0.01762462 | 0 | 0.00079861 | 0.00185256 | 0 | 0.00841751 | 0.00316607 | 0.0077687 | 0.00623053 | 0.00856715 |
| k__Bacteria;p__Proteobacteria;c__Gammaproteobacteria;o__Alteromonadales;f__Alteromonadaceae;g__Alteromonas | | 0 | 1.49E-05 | 0 | 3.96E-05 | 0.0007342 | 0.01175523 | 0 | 0.00058845 | 0.00158338 | 0 | 0 | 0.00094888 | 0.00989688 | 0.00880472 | 0.00844673 |
| k__Bacteria;p__Proteobacteria;c__Gammaproteobacteria;o__Alteromonadales;f__Alteromonadaceae;g__BD2-13 | | 0 | 0 | 0 | 0 | 0 | 0.0012084 | 0 | 0 | 0 | 0 | 0 | 0.00049793 | 0 | 0 | 1.72E-05 |
| k__Bacteria;p__Proteobacteria;c__Gammaproteobacteria;o__Alteromonadales;f__Alteromonadaceae;g__Candidatus Endobugula | | 0 | 0.00031278 | 0 | 0 | 0 | 0 | 0 | 0 | 0 | 0 | 0 | 1.88E-05 | 0 | 0 | 0 |
| k__Bacteria;p__Proteobacteria;c__Gammaproteobacteria;o__Alteromonadales;f__Alteromonadaceae;g__Cellvibrio | | 0 | 0.00087876 | 0 | 0 | 0 | 0 | 0 | 0 | 0 | 0 | 0 | 0 | 0 | 0 | 0 |
| k__Bacteria;p__Proteobacteria;c__Gammaproteobacteria;o__Alteromonadales;f__Alteromonadaceae;g__Glaciecola | | 0.0406117 | 0.05074471 | 0.01458596 | 0.00049562 | 0.00086078 | 0.07584177 | 0.00120703 | 0.10734221 | 0.27654538 | 0 | 0.00989057 | 0.22480059 | 0.19921794 | 0.07399574 | 0.17875759 |
| k__Bacteria;p__Proteobacteria;c__Gammaproteobacteria;o__Alteromonadales;f__Alteromonadaceae;g__HTCC2207 | | 0.01809132 | 0.0191689 | 0.00322574 | 9.91E-05 | 0.00070888 | 0.00642838 | 0 | 0 | 0.00029293 | 0 | 0 | 0.00106162 | 0.00034604 | 0.00018036 | 0.00025805 |
| k__Bacteria;p__Proteobacteria;c__Gammaproteobacteria;o__Alteromonadales;f__Alteromonadaceae;g__Marinobacter | | 0 | 0 | 0 | 0 | 0 | 0.00211265 | 0 | 0.0002606 | 0.00238299 | 0.00223214 | 0 | 0.00071401 | 0.00583085 | 0.00723069 | 0.02306938 |
| k__Bacteria;p__Proteobacteria;c__Gammaproteobacteria;o__Alteromonadales;f__Alteromonadaceae;g__Porticoccus | | 0 | 0 | 0 | 0 | 0 | 0 | 0 | 0 | 0 | 0 | 0 | 0 | 0 | 0 | 0 |
| k__Bacteria;p__Proteobacteria;c__Gammaproteobacteria;o__Alteromonadales;f__Alteromonadaceae;g__Spongiibacter | | 0 | 0 | 0 | 0 | 0 | 0 | 0 | 0 | 0 | 0 | 0 | 0 | 0 | 0 | 0 |
| k__Bacteria;p__Proteobacteria;c__Gammaproteobacteria;o__Alteromonadales;f__Alteromonadaceae;g__ZD0117 | | 0.00040395 | 0 | 0 | 0 | 0 | 0.00120018 | 0 | 0 | 0 | 0 | 0 | 0.00033822 | 0.00295868 | 0.00021315 | 0.00216759 |
| k__Bacteria;p__Proteobacteria;c__Gammaproteobacteria;o__Alteromonadales;f__Alteromonadaceae;g__nsmpVI18 | | 0 | 0 | 0 | 0 | 0 | 0 | 0 | 0 | 0 | 0 | 0 | 0 | 0 | 0 | 0 |
| k__Bacteria;p__Proteobacteria;c__Gammaproteobacteria;o__Alteromonadales;f__Colwelliaceae;g__ | | 0.00991127 | 0.00758117 | 0.01839274 | 0.00204195 | 0.00951923 | 0.02961002 | 0 | 0.00036988 | 0 | 0.01227679 | 0.01830808 | 0.03937393 | 0.02681846 | 0.01141171 | 0.03249669 |
| k__Bacteria;p__Proteobacteria;c__Gammaproteobacteria;o__Alteromonadales;f__Colwelliaceae;g__Colwellia | | 1.44E-05 | 0 | 0 | 0.0003172 | 0.00027849 | 3.29E-05 | 0 | 0 | 0 | 0 | 0 | 0 | 1.73E-05 | 6.56E-05 | 3.44E-05 |
| k__Bacteria;p__Proteobacteria;c__Gammaproteobacteria;o__Alteromonadales;f__Colwelliaceae;g__Thalassomonas | | 0.00024526 | 0 | 0 | 0 | 0 | 8.22E-06 | 0 | 0 | 0 | 0 | 0 | 3.76E-05 | 0 | 0 | 0 |
| k__Bacteria;p__Proteobacteria;c__Gammaproteobacteria;o__Alteromonadales;f__HTCC2188;g__ | | 0 | 0 | 0 | 5.95E-05 | 0 | 0.00041924 | 0 | 0 | 0 | 0 | 0 | 2.82E-05 | 0 | 0 | 0 |

| Taxon | 1180 | 1182 | 1183 | 1203 | 1211 | 1242 | 6475 | 6476 | 6477 | 1220 | 1221 | 1248 | 2032 | 2033 | 2034 |
|---|---|---|---|---|---|---|---|---|---|---|---|---|---|---|---|
| Copepod genus | Acartia | Acartia | Acartia | Acartia | Acartia | Acartia | Acartia | Acartia | Acartia | Acartia | Acartia | Acartia | Acartia | Acartia | Acartia |
| Type of sample | FullGut | FullGut | FullGut | FullGut | FullGut | FullGut | FullGut | FullGut | FullGut | Starved | Starved | Starved | Starved | Starved | Starved |
| k__Bacteria;p__Proteobacteria;c__Gammaproteobacteria;o__Alteromonadales;f__HTCC2188;g__HTCC | 0.00040395 | 7.45E-05 | 0.00036064 | 0 | 0 | 0.00043568 | 0 | 0 | 0 | 0 | 0 | 0.00029124 | 0.00051907 | 1.64E-05 | 0.00075694 |
| k__Bacteria;p__Proteobacteria;c__Gammaproteobacteria;o__Alteromonadales;f__Idiomarinaceae;g__Idiomarina | 7.21E-05 | 0 | 0 | 0 | 0 | 0.00244969 | 0 | 0.00029423 | 0.00044335 | 0 | 0 | 0.00027245 | 8.65E-05 | 0.00026234 | 0.00030966 |
| k__Bacteria;p__Proteobacteria;c__Gammaproteobacteria;o__Alteromonadales;f__Moritellaceae;g__Moritella | 0 | 0 | 0.00010018 | 0 | 0.00040507 | 0.00046034 | 0 | 0 | 0 | 0 | 0 | 0 | 3.46E-05 | 0 | 0 |
| k__Bacteria;p__Proteobacteria;c__Gammaproteobacteria;o__Alteromonadales;f__OM60;g__ | 0.00275554 | 0.00190646 | 0.00144257 | 9.91E-05 | 0 | 0.00457878 | 0.00603514 | 0.00169811 | 0.00258883 | 0 | 0.003367 | 0.00098646 | 0.00057097 | 0.00090179 | 0.00043008 |
| k__Bacteria;p__Proteobacteria;c__Gammaproteobacteria;o__Alteromonadales;f__OM60;g__Congregibacter | 0 | 0 | 0 | 0 | 0 | 0 | 0 | 0 | 0 | 0 | 0 | 0 | 0 | 0 | 0 |
| k__Bacteria;p__Proteobacteria;c__Gammaproteobacteria;o__Alteromonadales;f__Psychromonadaceae;g__Psychromonas | 0.00038953 | 0 | 0.00042075 | 0.00019825 | 0 | 0.00082204 | 0 | 0 | 0 | 0 | 0 | 0 | 0.00010381 | 0 | 3.44E-05 |
| k__Bacteria;p__Proteobacteria;c__Gammaproteobacteria;o__Alteromonadales;f__Shewanellaceae;g__Shewanella | 0.00030296 | 0 | 0.0042676 | 0 | 0 | 0.00028772 | 0 | 0 | 0 | 0 | 0 | 5.64E-05 | 0.00022493 | 9.84E-05 | 8.60E-05 |
| k__Bacteria;p__Proteobacteria;c__Gammaproteobacteria;o__Alteromonadales;f__[Chromatiaceae];g__ | 0 | 0 | 0 | 0 | 0 | 0 | 0 | 0 | 0 | 0 | 0 | 0 | 0 | 0 | 0 |
| k__Bacteria;p__Proteobacteria;c__Gammaproteobacteria;o__Alteromonadales;f__[Chromatiaceae];g__Rheinheimera | 0 | 0.00434912 | 0 | 5.95E-05 | 0 | 0 | 0 | 0 | 0 | 0 | 0 | 9.39E-06 | 0 | 0 | 0 |
| k__Bacteria;p__Proteobacteria;c__Gammaproteobacteria;o__Cardiobacteriales;f__Cardiobacteriaceae;g__Cardiobacterium | 0 | 0 | 0 | 0 | 0 | 0 | 0 | 0 | 0 | 0 | 0 | 0 | 0 | 0 | 0 |
| k__Bacteria;p__Proteobacteria;c__Gammaproteobacteria;o__Chromatiales;f__;g__ | 0.00014427 | 0 | 0 | 0 | 0 | 0 | 0 | 0 | 7.92E-05 | 0 | 0 | 0 | 0 | 0 | 0 |
| k__Bacteria;p__Proteobacteria;c__Gammaproteobacteria;o__Chromatiales;f__Chromatiaceae;g__ | 0 | 0 | 0 | 0 | 0 | 0 | 0 | 0 | 0 | 0 | 0 | 0 | 0 | 0 | 0 |
| k__Bacteria;p__Proteobacteria;c__Gammaproteobacteria;o__Enterobacteriales;f__Enterobacteriaceae;g__ | 5.77E-05 | 0 | 0 | 0.00021807 | 7.60E-05 | 9.04E-05 | 0.00276224 | 2.52E-05 | 1.58E-05 | 0.00297619 | 0.00273569 | 0 | 0 | 0 | 0 |
| k__Bacteria;p__Proteobacteria;c__Gammaproteobacteria;o__Enterobacteriales;f__Enterobacteriaceae;g__Enterobacter | 2.89E-05 | 0 | 0 | 0 | 0 | 0 | 0 | 0 | 0 | 0 | 0 | 0 | 0 | 0 | 0 |
| k__Bacteria;p__Proteobacteria;c__Gammaproteobacteria;o__Enterobacteriales;f__Enterobacteriaceae;g__Erwinia | 0.00012984 | 0 | 0 | 0 | 0 | 0 | 0 | 0 | 0 | 0 | 0 | 0 | 0 | 0 | 0 |
| k__Bacteria;p__Proteobacteria;c__Gammaproteobacteria;o__Enterobacteriales;f__Enterobacteriaceae;g__Escherichia | 0 | 0 | 0 | 0 | 0 | 0 | 0 | 0 | 0 | 0 | 0 | 0 | 0 | 0 | 0 |
| k__Bacteria;p__Proteobacteria;c__Gammaproteobacteria;o__Enterobacteriales;f__Enterobacteriaceae;g__Ewingella | 0 | 0 | 0 | 0 | 0 | 0 | 0 | 0 | 0 | 0 | 0 | 0 | 0 | 0 | 1.72E-05 |
| k__Bacteria;p__Proteobacteria;c__Gammaproteobacteria;o__Enterobacteriales;f__Enterobacteriaceae;g__Gluconacetobacter | 0 | 0 | 0 | 0 | 0 | 0.00058365 | 0.00408533 | 0.00028582 | 0.00088669 | 0 | 0 | 4.70E-05 | 0 | 3.28E-05 | 0 |
| k__Bacteria;p__Proteobacteria;c__Gammaproteobacteria;o__Enterobacteriales;f__Enterobacteriaceae;g__Klebsiella | 0 | 0 | 0 | 0 | 0 | 0 | 0 | 0 | 0 | 0 | 0 | 0 | 0 | 0 | 0 |
| k__Bacteria;p__Proteobacteria;c__Gammaproteobacteria;o__Enterobacteriales;f__Enterobacteriaceae;g__Pantoea | 0 | 0 | 0 | 0 | 0 | 0 | 0 | 0 | 0 | 0 | 0 | 0 | 0 | 0 | 0 |
| k__Bacteria;p__Proteobacteria;c__Gammaproteobacteria;o__Enterobacteriales;f__Enterobacteriaceae;g__Salmonella | 0 | 0 | 0 | 0 | 0 | 0 | 0 | 0 | 0 | 0 | 0 | 0 | 0 | 0 | 0 |
| k__Bacteria;p__Proteobacteria;c__Gammaproteobacteria;o__Enterobacteriales;f__Enterobacteriaceae;g__Serratia | 0 | 0 | 0 | 0 | 0 | 0 | 0 | 1.68E-05 | 0 | 0 | 0 | 0 | 0 | 0 | 0 |
| k__Bacteria;p__Proteobacteria;c__Gammaproteobacteria;o__HTCC2188;f__HTCC2089;g__ | 0.00148597 | 0.00177242 | 0.00014025 | 0 | 0 | 0.00187426 | 0.00106776 | 0 | 0.00050668 | 0 | 0.00021044 | 0.0003664 | 0.00027684 | 0.00026234 | 0.00020644 |
| k__Bacteria;p__Proteobacteria;c__Gammaproteobacteria;o__HTCC2188;f__HTCC2089;g__Acinetobacter | 0 | 0 | 0 | 0 | 0 | 0 | 0 | 0 | 0 | 0 | 0 | 0 | 0 | 0 | 0 |
| k__Bacteria;p__Proteobacteria;c__Gammaproteobacteria;o__Legionellales;f__;g__ | 0 | 0 | 0 | 0 | 0 | 0 | 0 | 0 | 0 | 0 | 0 | 0 | 0 | 0 | 0 |
| k__Bacteria;p__Proteobacteria;c__Gammaproteobacteria;o__Legionellales;f__Coxiellaceae;g__ | 0 | 0 | 0 | 0 | 0 | 0 | 0 | 0 | 0 | 0 | 0 | 0 | 0 | 0 | 0 |
| k__Bacteria;p__Proteobacteria;c__Gammaproteobacteria;o__Legionellales;f__Francisellaceae;g__ | 0.00025968 | 0 | 0 | 0 | 0 | 0.00028772 | 0 | 0 | 0 | 0 | 0 | 0 | 0 | 0 | 0 |
| k__Bacteria;p__Proteobacteria;c__Gammaproteobacteria;o__Legionellales;f__Legionellaceae;g__ | 0 | 0 | 0 | 0 | 0 | 0 | 0 | 5.88E-05 | 0.00037209 | 0 | 0 | 5.64E-05 | 0 | 1.64E-05 | 0 |
| k__Bacteria;p__Proteobacteria;c__Gammaproteobacteria;o__Legionellales;f__Legionellaceae;g__Legionella | 0 | 0 | 0 | 0 | 0 | 0 | 0 | 0 | 0 | 0 | 0 | 0 | 0 | 0 | 0 |
| k__Bacteria;p__Proteobacteria;c__Gammaproteobacteria;o__Methylococcales;f__;g__ | 0 | 0 | 0 | 0 | 0 | 0 | 0 | 0 | 0 | 0 | 0 | 0 | 0 | 0 | 0 |
| k__Bacteria;p__Proteobacteria;c__Gammaproteobacteria;o__Oceanospirillales;f__;g__ | 0.00272668 | 0.00463211 | 0.00138246 | 0 | 0 | 0.00016441 | 0 | 0 | 0.00045126 | 0 | 0 | 1.88E-05 | 0.00022493 | 0.00031153 | 0.00020644 |
| k__Bacteria;p__Proteobacteria;c__Gammaproteobacteria;o__Oceanospirillales;f__Alcanivoracaceae;g__Alcanivorax | 0 | 0 | 0 | 0 | 0 | 0.00039458 | 0 | 0.00025219 | 0 | 0 | 0 | 9.39E-06 | 0.00010381 | 6.56E-05 | 6.88E-05 |
| k__Bacteria;p__Proteobacteria;c__Gammaproteobacteria;o__Oceanospirillales;f__Endozoicimonaceae;g__Endozoicomonas | 0 | 0 | 0 | 0 | 0 | 0 | 0 | 0 | 0 | 0 | 0 | 0 | 0 | 0 | 0 |
| k__Bacteria;p__Proteobacteria;c__Gammaproteobacteria;o__Oceanospirillales;f__Halomonadaceae;g__ | 0 | 0 | 0 | 0 | 0 | 0.00320597 | 0 | 0.00117691 | 0.002668 | 0 | 0 | 0.00096767 | 0.00238771 | 0.00444335 | 0.00213319 |
| k__Bacteria;p__Proteobacteria;c__Gammaproteobacteria;o__Oceanospirillales;f__Halomonadaceae;g__Candidatus Portiera | 0.07509197 | 0.08585046 | 0.01711046 | 0 | 0 | 0.01778081 | 0.01158283 | 0.00287501 | 0.00615143 | 0 | 0.00441919 | 0.00675492 | 0.0061596 | 0.00180357 | 0.00562542 |
| k__Bacteria;p__Proteobacteria;c__Gammaproteobacteria;o__Oceanospirillales;f__Halomonadaceae;g__Cobetia | 0 | 0 | 0 | 0 | 0 | 0 | 0 | 0 | 0 | 0 | 0 | 0 | 0 | 0 | 0 |
| k__Bacteria;p__Proteobacteria;c__Gammaproteobacteria;o__Oceanospirillales;f__Halomonadaceae;g__Haererehalobacter | 0 | 0 | 0 | 0 | 0 | 0.00013975 | 0 | 0 | 0 | 0 | 0 | 0 | 0 | 0 | 0 |
| k__Bacteria;p__Proteobacteria;c__Gammaproteobacteria;o__Oceanospirillales;f__Halomonadaceae;g__Halomonas | 0.00024526 | 0 | 0 | 0 | 0.00116459 | 0.00466921 | 0 | 0.00040351 | 0.00412471 | 0 | 0 | 0.00035701 | 0.00031144 | 0.00059026 | 0.00693286 |
| k__Bacteria;p__Proteobacteria;c__Gammaproteobacteria;o__Oceanospirillales;f__Oceanospirillaceae;g__ | 0.00780495 | 0.00612154 | 0.00254453 | 0.00027755 | 0 | 0.00041102 | 0 | 0 | 0 | 0 | 0.00904882 | 0.00179442 | 0.00058828 | 0.00022955 | 0.00104939 |
| k__Bacteria;p__Proteobacteria;c__Gammaproteobacteria;o__Oceanospirillales;f__Oceanospirillaceae;g__Amphritea | 0 | 0 | 0 | 0 | 0 | 0 | 0 | 0 | 0 | 0 | 0 | 0 | 0 | 0 | 0 |
| k__Bacteria;p__Proteobacteria;c__Gammaproteobacteria;o__Oceanospirillales;f__Oceanospirillaceae;g__Marinobacterium | 0 | 0 | 0 | 0 | 0 | 0 | 0 | 0 | 0 | 0 | 0 | 0 | 0 | 0 | 0 |
| k__Bacteria;p__Proteobacteria;c__Gammaproteobacteria;o__Oceanospirillales;f__Oceanospirillaceae;g__Marinomonas | 0.00108202 | 0.00090855 | 0.00116207 | 9.91E-05 | 0.00549381 | 0.00287715 | 0.0034586 | 0.00258079 | 0.00590601 | 0 | 0.00862795 | 0.14174989 | 0.00337394 | 0.00304968 | 0.00485128 |
| k__Bacteria;p__Proteobacteria;c__Gammaproteobacteria;o__Oceanospirillales;f__Oceanospirillaceae;g__Neptunomonas | 0 | 0 | 0 | 0 | 0 | 0 | 0 | 0 | 0 | 0 | 0 | 0 | 0 | 0 | 0 |
| k__Bacteria;p__Proteobacteria;c__Gammaproteobacteria;o__Oceanospirillales;f__Oceanospirillaceae;g__Oceaniserpentilla | 0.00024526 | 0.00031278 | 8.01E-05 | 0 | 0 | 0 | 0 | 0 | 0 | 0 | 0 | 0 | 0 | 0 | 0 |
| k__Bacteria;p__Proteobacteria;c__Gammaproteobacteria;o__Oceanospirillales;f__Oceanospirillaceae;g__Oleibacter | 0.0007502 | 1.49E-05 | 0 | 0.04656834 | 0.01202562 | 0.0310075 | 0 | 0.0003951 | 0.00076002 | 0.17317708 | 0.1635101 | 0.00954519 | 0.00098623 | 0.00059026 | 0.00103219 |
| k__Bacteria;p__Proteobacteria;c__Gammaproteobacteria;o__Oceanospirillales;f__Oceanospirillaceae;g__Oleispira | 0.00786266 | 0.00695562 | 0.00390695 | 0 | 0 | 0.00017263 | 0 | 0 | 0 | 0 | 0 | 0.00217022 | 0.00019032 | 0.00024594 | 0.00027525 |
| k__Bacteria;p__Proteobacteria;c__Gammaproteobacteria;o__Oceanospirillales;f__Oceanospirillaceae;g__Spongiispira | 0 | 0 | 0 | 0 | 0 | 0 | 0 | 0 | 0 | 0 | 0 | 0 | 0 | 0 | 0 |
| k__Bacteria;p__Proteobacteria;c__Gammaproteobacteria;o__Oceanospirillales;f__Oleiphilaceae;g__ | 0 | 0 | 0 | 0 | 0 | 0.00145502 | 0 | 8.41E-05 | 0 | 0.00251116 | 0 | 0.00050732 | 3.46E-05 | 6.56E-05 | 0.00012042 |
| k__Bacteria;p__Proteobacteria;c__Gammaproteobacteria;o__Oceanospirillales;f__SUP05;g__ | 0.00942076 | 0.01042598 | 0.00366653 | 0 | 0 | 7.40E-05 | 0 | 2.52E-05 | 0.00036418 | 0 | 0 | 0.00010334 | 0 | 1.64E-05 | 3.44E-05 |
| k__Bacteria;p__Proteobacteria;c__Gammaproteobacteria;o__Oceanospirillales;f__Saccharospirillaceae;g__Reinekea | 0 | 0 | 0 | 0 | 0 | 0 | 0 | 0 | 0 | 0 | 0 | 3.76E-05 | 0 | 0 | 0 |
| k__Bacteria;p__Proteobacteria;c__Gammaproteobacteria;o__Oceanospirillales;f__Saccharospirillaceae;g__Saccharospirillum | 0 | 0 | 0 | 0 | 0 | 0 | 0 | 0 | 0 | 0 | 0 | 0 | 0 | 0 | 0 |
| k__Bacteria;p__Proteobacteria;c__Gammaproteobacteria;o__Pasteurellales;f__Pasteurellaceae;g__Actinobacillus | 0 | 0 | 0 | 3.96E-05 | 0 | 0 | 0 | 0 | 0 | 0 | 0 | 0 | 0 | 0 | 3.44E-05 |
| k__Bacteria;p__Proteobacteria;c__Gammaproteobacteria;o__Pasteurellales;f__Pasteurellaceae;g__Aggregatibacter | 0 | 0 | 0 | 0 | 0 | 0 | 0 | 8.41E-05 | 0 | 0 | 0 | 0 | 0 | 0 | 0 |
| k__Bacteria;p__Proteobacteria;c__Gammaproteobacteria;o__Pasteurellales;f__Pasteurellaceae;g__Haemophilus | 0.00057708 | 0 | 0 | 0 | 0.00025317 | 0 | 0 | 5.04E-05 | 0.00011084 | 0 | 0.00252525 | 2.82E-05 | 0 | 0 | 0.00018923 |
| k__Bacteria;p__Proteobacteria;c__Gammaproteobacteria;o__Pseudomonadales;f__Moraxellaceae;g__ | 0.00056265 | 0.00017873 | 0 | 0.00267634 | 0.02291197 | 0.03777292 | 0.04466006 | 0.00166448 | 0.00792482 | 0.140625 | 0.1121633 | 0.00492292 | 0.00264724 | 0.00477127 | 0.00175472 |
| k__Bacteria;p__Proteobacteria;c__Gammaproteobacteria;o__Pseudomonadales;f__Moraxellaceae;g__Acinetobacter | 0.00028854 | 0.00728329 | 0.00040071 | 3.96E-05 | 0.00045571 | 0.00042746 | 0.00304078 | 8.41E-06 | 0 | 0 | 0.00589226 | 1.88E-05 | 1.73E-05 | 0 | 5.16E-05 |
| k__Bacteria;p__Proteobacteria;c__Gammaproteobacteria;o__Pseudomonadales;f__Moraxellaceae;g__Alkanindiges | 0 | 0 | 0 | 0 | 0 | 0 | 0 | 0 | 0 | 0 | 0 | 0 | 0 | 0 | 0 |
| k__Bacteria;p__Proteobacteria;c__Gammaproteobacteria;o__Pseudomonadales;f__Moraxellaceae;g__Enhydrobacter | 0 | 0 | 0 | 3.96E-05 | 0 | 0 | 0 | 0.00031104 | 0 | 0 | 0 | 0 | 0 | 0 | 0 |
| k__Bacteria;p__Proteobacteria;c__Gammaproteobacteria;o__Pseudomonadales;f__Moraxellaceae;g__Perlucidibaca | 0 | 1.49E-05 | 0 | 0 | 0 | 0 | 0 | 0 | 0 | 0 | 0 | 0 | 0 | 0 | 0 |
| k__Bacteria;p__Proteobacteria;c__Gammaproteobacteria;o__Pseudomonadales;f__Moraxellaceae;g__Psychrobacter | 0 | 0 | 0 | 0.00120931 | 0.02438036 | 0.02074017 | 0.00933126 | 0.00036988 | 0.00218507 | 0.07710193 | 0.02546296 | 0.00795746 | 0.00221469 | 0.00831284 | 0.00153108 |
| k__Bacteria;p__Proteobacteria;c__Gammaproteobacteria;o__Pseudomonadales;f__Pseudomonadaceae;g__ | 0.00028854 | 0.00034257 | 2.00E-05 | 0.00025772 | 0.00086078 | 0.00048501 | 0 | 0 | 0 | 0.00688244 | 0.00399832 | 0 | 0 | 0 | 0 |
| k__Bacteria;p__Proteobacteria;c__Gammaproteobacteria;o__Pseudomonadales;f__Pseudomonadaceae;g__Pseudomonas | 0.0002164 | 0.00165326 | 0.00038068 | 0.00019825 | 0.00136712 | 0.0033786 | 0.00805459 | 0.00089109 | 0.00301634 | 0.01488095 | 0.00441919 | 0.00040398 | 0 | 0 | 6.88E-05 |
| k__Bacteria;p__Proteobacteria;c__Gammaproteobacteria;o__Salinisphaerales;f__Salinisphaeraceae;g__Salinisphaera | 0 | 0 | 0 | 0 | 0 | 0 | 0 | 7.57E-05 | 0 | 0 | 0 | 0 | 0 | 0 | 0 |
| k__Bacteria;p__Proteobacteria;c__Gammaproteobacteria;o__Thiohalorhabdales;f__;g__ | 0 | 0 | 0 | 0.00227985 | 0 | 0 | 0 | 0 | 0 | 0 | 0 | 0 | 0 | 0 | 0 |
| k__Bacteria;p__Proteobacteria;c__Gammaproteobacteria;o__Thiohalorhabdales;f__Thiohalorhabdaceae;g__ | 0 | 0 | 0 | 5.95E-05 | 0 | 0.00026305 | 0 | 0 | 0 | 0 | 0 | 9.39E-06 | 0 | 0 | 0 |
| k__Bacteria;p__Proteobacteria;c__Gammaproteobacteria;o__Thiotrichales;f__Piscirickettsiaceae;g__ | 0.00121186 | 0.00215967 | 0.0011821 | 0 | 0 | 0 | 0 | 0.00020176 | 0.00190797 | 0 | 0 | 0.00012213 | 0.00027684 | 0.00013117 | 0.00020644 |
| k__Bacteria;p__Proteobacteria;c__Gammaproteobacteria;o__Thiotrichales;f__Piscirickettsiaceae;g__Methylophaga | 0 | 0 | 0 | 0 | 0 | 0 | 0 | 7.57E-05 | 0 | 0 | 0 | 0 | 0 | 0 | 0.00015483 |
| k__Bacteria;p__Proteobacteria;c__Gammaproteobacteria;o__Thiotrichales;f__Thiotrichaceae;g__ | 0 | 0 | 0 | 0 | 0 | 0 | 0 | 0 | 0 | 0 | 0 | 0 | 0 | 0 | 0 |
| k__Bacteria;p__Proteobacteria;c__Gammaproteobacteria;o__Thiotrichales;f__Thiotrichaceae;g__Cocleimonas | 0 | 0 | 0 | 0 | 0 | 1.64E-05 | 0 | 0 | 0 | 0 | 0 | 0 | 0 | 0 | 6.88E-05 |
| k__Bacteria;p__Proteobacteria;c__Gammaproteobacteria;o__Thiotrichales;f__Thiotrichaceae;g__E8 | 0 | 0 | 0 | 0 | 0 | 0 | 0 | 0 | 0 | 0 | 0 | 0 | 0 | 0 | 0 |
| k__Bacteria;p__Proteobacteria;c__Gammaproteobacteria;o__Thiotrichales;f__Thiotrichaceae;g__Leucothrix | 0 | 0 | 0 | 0.06861346 | 0 | 0.00074806 | 0 | 0.00056323 | 0 | 0 | 0 | 0 | 0 | 0 | 0 |
| k__Bacteria;p__Proteobacteria;c__Gammaproteobacteria;o__Thiotrichales;f__Thiotrichaceae;g__Thiothrix | 5.77E-05 | 0 | 0 | 3.96E-05 | 0 | 0.00082204 | 0 | 0 | 0 | 0 | 0 | 0 | 0 | 0 | 0 |
| k__Bacteria;p__Proteobacteria;c__Gammaproteobacteria;o__Vibrionales;f__;g__ | 0.12495131 | 0.10564492 | 0.35739617 | 0.00085246 | 0.00124054 | 0.00552414 | 0.00399248 | 0.00128619 | 0.00292134 | 0 | 0.00610269 | 0.01701412 | 0.01408402 | 0.00947696 | 0.00760378 |
| k__Bacteria;p__Proteobacteria;c__Gammaproteobacteria;o__Vibrionales;f__Pseudoalteromonadaceae;g__Pseudoalteromonas | 0.07278367 | 0.06051534 | 0.0630923 | 0.03294873 | 0.43494772 | 0.1111239 | 0.0165038 | 0.00614513 | 0.01121825 | 0.10063244 | 0.08333333 | 0.20276021 | 0.41663783 | 0.49985243 | 0.41265117 |

| Taxon | | 1180 | 1182 | 1183 | 1203 | 1211 | 1242 | 6475 | 6476 | 6477 | 1220 | 1221 | 1248 | 2032 | 2033 | 2034 |
|---|---|---|---|---|---|---|---|---|---|---|---|---|---|---|---|---|
| | Copepod genus | Acartia | Acartia | Acartia | Acartia | Acartia | Acartia | Acartia | Acartia | Acartia | Acartia | Acartia | Acartia | Acartia | Acartia | Acartia |
| | Type of sample | FullGut | FullGut | FullGut | FullGut | FullGut | FullGut | FullGut | FullGut | FullGut | Starved | Starved | Starved | Starved | Starved | Starved |
| k__Bacteria;p__Proteobacteria;c__Gammaproteobacteria;o__Vibrionales;f__Vibrionaceae;g__ | | 0.11801197 | 0.07283289 | 0.33429505 | 0.0002379 | 0.00048102 | 0.0052693 | 0.00148557 | 0.00187464 | 0.00285009 | 0 | 0.00021044 | 0.00791049 | 0.00335663 | 0.00137727 | 0.00240844 |
| k__Bacteria;p__Proteobacteria;c__Gammaproteobacteria;o__Vibrionales;f__Vibrionaceae;g__Aliivibrio | | 0.01719685 | 0.01587727 | 0.0466831 | 0.00043614 | 0 | 0.00088781 | 0 | 0.00042873 | 0.0006571 | 0 | 0 | 0.00158773 | 0.00257803 | 0.00103296 | 0.0011182 |
| k__Bacteria;p__Proteobacteria;c__Gammaproteobacteria;o__Vibrionales;f__Vibrionaceae;g__Enterovibrio | | 0 | 0 | 0 | 5.95E-05 | 0 | 0.00038636 | 0 | 0 | 0 | 0.0046503 | 0 | 7.52E-05 | 0.00012112 | 0 | 0 |
| k__Bacteria;p__Proteobacteria;c__Gammaproteobacteria;o__Vibrionales;f__Vibrionaceae;g__Listonella | | 0.00036067 | 0.0002681 | 0.001122 | 0 | 0 | 0 | 0 | 8.41E-06 | 0 | 0 | 0 | 7.52E-05 | 0 | 1.64E-05 | 1.72E-05 |
| k__Bacteria;p__Proteobacteria;c__Gammaproteobacteria;o__Vibrionales;f__Vibrionaceae;g__Photobacterium | | 0 | 0 | 0 | 0 | 0 | 0 | 0 | 0 | 0 | 0 | 0 | 5.64E-05 | 0.00019032 | 0.00013117 | 5.16E-05 |
| k__Bacteria;p__Proteobacteria;c__Gammaproteobacteria;o__Vibrionales;f__Vibrionaceae;g__Vibrio | | 0 | 0.00044683 | 0 | 0 | 0.00111395 | 0 | 0 | 0 | 0 | 0.00279018 | 0 | 2.82E-05 | 0 | 0 | 0 |
| k__Bacteria;p__Proteobacteria;c__Gammaproteobacteria;o__Xanthomonadales;f__Sinobacteraceae;g__ | | 1.44E-05 | 0.00014894 | 0 | 1.98E-05 | 0.00012659 | 4.11E-05 | 0.0001857 | 1.68E-05 | 7.92E-06 | 0.00018601 | 0 | 6.58E-05 | 0 | 1.64E-05 | 0 |
| k__Bacteria;p__Proteobacteria;c__Gammaproteobacteria;o__Xanthomonadales;f__Xanthomonadaceae;g__ | | 0.00030296 | 0.00011915 | 0.00010018 | 0.00013877 | 0.00078483 | 0.00054255 | 0 | 8.41E-06 | 0 | 0 | 0 | 0 | 6.92E-05 | 9.84E-05 | 0 |
| k__Bacteria;p__Proteobacteria;c__Gammaproteobacteria;o__Xanthomonadales;f__Xanthomonadaceae;g__Aspromonas | | 0 | 0 | 0 | 0 | 0 | 0 | 0 | 8.41E-06 | 0 | 0 | 0 | 0 | 0 | 0 | 0 |
| k__Bacteria;p__Proteobacteria;c__Gammaproteobacteria;o__Xanthomonadales;f__Xanthomonadaceae;g__Ignatzschineria | | 0 | 0 | 0 | 0 | 0 | 0 | 0 | 0 | 0 | 0 | 0 | 0 | 0 | 0 | 0 |
| k__Bacteria;p__Proteobacteria;c__Gammaproteobacteria;o__Xanthomonadales;f__Xanthomonadaceae;g__Lysobacter | | 0 | 0 | 0 | 0 | 0.00025317 | 0 | 0 | 0 | 0 | 0 | 0 | 0 | 0 | 0 | 0 |
| k__Bacteria;p__Proteobacteria;c__Gammaproteobacteria;o__Xanthomonadales;f__Xanthomonadaceae;g__Pseudoxanthomonas | | 0 | 0.00016384 | 0 | 0 | 0 | 0 | 0 | 0 | 0 | 0 | 0 | 0 | 0 | 0 | 0 |
| k__Bacteria;p__Proteobacteria;c__Gammaproteobacteria;o__Xanthomonadales;f__Xanthomonadaceae;g__Stenotrophomonas | | 0.00025968 | 0 | 0.00064114 | 7.93E-05 | 0.00156966 | 0.00096179 | 0 | 0 | 0 | 0.00558036 | 0 | 0 | 0 | 9.84E-05 | 1.72E-05 |
| k__Bacteria;p__Proteobacteria;c__Gammaproteobacteria;o__Xanthomonadales;f__Xanthomonadaceae;g__Thermomonas | | 0 | 0 | 0 | 0 | 0 | 0 | 0 | 0.00016813 | 0 | 0 | 0 | 0 | 0 | 0 | 0 |
| k__Bacteria;p__Proteobacteria;c__Gammaproteobacteria;o__[Marinicellales];f__[Marinicellaceae];g__ | | 0 | 0 | 0 | 0 | 0 | 0 | 0 | 0 | 0 | 0 | 0 | 0 | 0 | 0 | 0 |
| k__Bacteria;p__Proteobacteria;c__Gammaproteobacteria;o__[Marinicellales];f__[Marinicellaceae];g__Marinicella | | 0 | 0 | 0 | 0 | 0 | 0 | 0 | 2.52E-05 | 0 | 0 | 0 | 0 | 0 | 0 | 1.72E-05 |
| k__Bacteria;p__Proteobacteria;c__TA18;o__CV90;f__;g__ | | 0 | 0 | 0 | 0 | 0 | 0 | 0 | 0 | 0 | 0 | 0 | 0 | 0 | 0 | 0 |
| k__Bacteria;p__Proteobacteria;c__Zetaproteobacteria;o__Mariprofundales;f__Mariprofundaceae;g__Mariprofundus | | 0 | 0 | 0 | 0 | 0 | 0 | 0 | 0 | 0 | 0 | 0 | 0 | 0 | 0 | 0 |
| k__Bacteria;p__SAR406;c__AB16;o__Arctic96B-7;f__A714017;g__SGSH944 | | 0.00017312 | 0 | 0.00030053 | 0 | 0 | 0 | 0 | 0 | 0 | 0 | 0 | 0 | 0 | 0 | 0 |
| k__Bacteria;p__SAR406;c__AB16;o__Arctic96B-7;f__A714017;g__SargSea-WGS | | 0.00010099 | 0 | 0 | 0 | 0 | 0 | 0 | 0 | 0 | 0 | 0 | 0 | 0 | 0 | 0 |
| k__Bacteria;p__SAR406;c__AB16;o__Arctic96B-7;f__A714017;g__ZA3312c | | 0.00012984 | 0.00064045 | 0 | 0 | 0.00063293 | 0 | 0 | 0 | 0 | 0 | 0 | 0 | 0 | 0 | 0 |
| k__Bacteria;p__SAR406;c__AB16;o__ZA3648c;f__AEGEAN_185;g__ | | 1.44E-05 | 0 | 0 | 0 | 0 | 0 | 0 | 0 | 0 | 0 | 0 | 0 | 0 | 0 | 0 |
| k__Bacteria;p__SBR1093;c__A712011;o__;f__;g__ | | 0 | 0 | 0 | 0 | 0 | 0 | 0 | 0 | 0 | 0 | 0 | 0 | 0 | 0 | 0 |
| k__Bacteria;p__SR1;c__;o__;f__;g__ | | 0 | 0 | 0 | 0 | 0 | 0 | 0 | 0 | 0 | 0 | 0 | 0 | 0 | 0 | 0 |
| k__Bacteria;p__Spirochaetes;c__Spirochaetes;o__Sphaerochaetales;f__Sphaerochaetaceae;g__wall-less | | 0 | 0 | 0 | 0 | 0 | 0 | 0 | 0 | 0 | 0 | 0 | 0 | 0 | 0 | 0 |
| k__Bacteria;p__Spirochaetes;c__Spirochaetes;o__Spirochaetales;f__Spirochaetaceae;g__Spirochaeta | | 0 | 0 | 0 | 0 | 0 | 0 | 0 | 0 | 0 | 0 | 0.00126263 | 0 | 0 | 0 | 0 |
| k__Bacteria;p__Spirochaetes;c__Spirochaetes;o__Spirochaetales;f__Spirochaetaceae;g__Treponema | | 0 | 0 | 0 | 0 | 0 | 0 | 0 | 0 | 0 | 0 | 0 | 0 | 0 | 0 | 0 |
| k__Bacteria;p__TM6;c__SJA-4;o__;f__;g__ | | 0 | 0 | 0 | 0 | 0 | 0 | 0 | 0 | 0 | 0 | 0 | 0 | 0 | 0 | 0 |
| k__Bacteria;p__TM7;c__TM7-1;o__;f__;g__ | | 0 | 0.0002532 | 0 | 0 | 0 | 0 | 0 | 6.73E-05 | 0 | 0 | 0 | 0 | 0 | 0 | 0 |
| k__Bacteria;p__TM7;c__TM7-3;o__;f__;g__ | | 0 | 0.00016384 | 0 | 0 | 0 | 0 | 0.00304078 | 0.00025219 | 0 | 0 | 0 | 0 | 0 | 0 | 0 |
| k__Bacteria;p__TM7;c__TM7-3;o__CW040;f__F16;g__ | | 0 | 0 | 0 | 0 | 0 | 0 | 0 | 0 | 0 | 0 | 0 | 0 | 0 | 0 | 0 |
| k__Bacteria;p__TM7;c__TM7-3;o__EW055;f__;g__ | | 0 | 0.00011915 | 0 | 9.91E-05 | 0 | 0 | 0 | 0 | 0 | 0 | 0 | 0 | 0 | 0 | 0 |
| k__Bacteria;p__TM7;c__TM7-3;o__I025;f__Rs-045;g__ | | 0 | 0 | 0 | 0 | 0 | 0 | 0 | 0 | 0 | 0 | 0 | 0 | 0 | 0 | 0 |
| k__Bacteria;p__Tenericutes;c__Mollicutes;o__Acholeplasmatales;f__Acholeplasmataceae;g__Acholeplasma | | 0 | 0 | 0 | 0 | 0 | 0 | 0 | 0 | 0 | 0 | 0 | 0 | 0 | 0 | 1.72E-05 |
| k__Bacteria;p__Tenericutes;c__Mollicutes;o__Mycoplasmatales;f__Mycoplasmataceae;g__Mycoplasma | | 0 | 0 | 0 | 0 | 0 | 0 | 0 | 0 | 0 | 0 | 0 | 0 | 0 | 0 | 0 |
| k__Bacteria;p__Thermotogae;c__Thermotogae;o__Thermotogales;f__Thermotogaceae;g__Fervidobacterium | | 0 | 0 | 0 | 0 | 0 | 0 | 0 | 0 | 0 | 0 | 0 | 0 | 0 | 0 | 0 |
| k__Bacteria;p__Verrucomicrobia;c__Opitutae;o__Opitutales;f__Opitutaceae;g__Opitutus | | 0 | 0 | 0 | 0 | 0 | 0 | 0 | 0 | 0 | 0 | 0 | 0 | 0 | 0 | 0 |
| k__Bacteria;p__Verrucomicrobia;c__Opitutae;o__Puniceicoccales;f__Puniceicoccaceae;g__Coraliomargarita | | 0.00014427 | 0 | 0 | 9.91E-05 | 0 | 0.00041102 | 0 | 0 | 0 | 0 | 0 | 3.76E-05 | 5.19E-05 | 0 | 0 |
| k__Bacteria;p__Verrucomicrobia;c__Opitutae;o__Puniceicoccales;f__Puniceicoccaceae;g__MB11C04 | | 0.00010099 | 0.00019363 | 0.00018032 | 0 | 0 | 0.00186604 | 0 | 0.00048758 | 0.00049085 | 0 | 0 | 0.00025366 | 0.00032874 | 0 | 0.00034406 |
| k__Bacteria;p__Verrucomicrobia;c__Verrucomicrobiae;o__Verrucomicrobiales;f__Verrucomicrobiaceae;g__ | | 0 | 0 | 0 | 0 | 0 | 0.00020551 | 0.00304078 | 0.00010928 | 0.00035626 | 0.00511533 | 0 | 0 | 0.00112465 | 0.0008362 | 0.00043008 |
| k__Bacteria;p__Verrucomicrobia;c__Verrucomicrobiae;o__Verrucomicrobiales;f__Verrucomicrobiaceae;g__Akkermansia | | 0 | 0.00016384 | 0 | 0 | 0 | 0 | 0 | 0 | 0 | 0 | 0 | 0 | 0 | 0 | 0 |
| k__Bacteria;p__Verrucomicrobia;c__Verrucomicrobiae;o__Verrucomicrobiales;f__Verrucomicrobiaceae;g__Luteolibacter | | 0 | 0 | 0 | 0 | 0 | 0.00036992 | 0.00276224 | 0 | 0 | 0 | 0 | 0 | 0 | 4.92E-05 | 0 |
| k__Bacteria;p__Verrucomicrobia;c__Verrucomicrobiae;o__Verrucomicrobiales;f__Verrucomicrobiaceae;g__MSBL3 | | 0 | 0 | 0 | 0 | 0 | 0 | 0 | 0 | 0 | 0 | 0 | 0 | 0 | 0 | 0 |
| k__Bacteria;p__Verrucomicrobia;c__Verrucomicrobiae;o__Verrucomicrobiales;f__Verrucomicrobiaceae;g__Persicirhabdus | | 0 | 0 | 0 | 0 | 0 | 0.00105222 | 0.00148557 | 7.57E-05 | 0 | 0 | 0 | 0.00025366 | 0.00017302 | 0.00013117 | 0.00017203 |
| k__Bacteria;p__Verrucomicrobia;c__Verrucomicrobiae;o__Verrucomicrobiales;f__Verrucomicrobiaceae;g__Prosthecobacter | | 0 | 0 | 0 | 0 | 0 | 0 | 0 | 0 | 0 | 0 | 0 | 0 | 0 | 0 | 0 |
| k__Bacteria;p__Verrucomicrobia;c__Verrucomicrobiae;o__Verrucomicrobiales;f__Verrucomicrobiaceae;g__Rubritalea | | 0 | 0 | 0 | 0 | 0 | 0 | 0.00083564 | 0 | 0 | 0 | 0 | 0 | 0 | 0 | 0 |
| k__Bacteria;p__Verrucomicrobia;c__Verrucomicrobiae;o__Verrucomicrobiales;f__Verrucomicrobiaceae;g__Verrucomicrobium | | 0 | 0 | 0 | 0 | 0 | 0.00051789 | 0 | 0.00010928 | 0.00038793 | 0 | 0 | 0.00013153 | 0.00103813 | 0.00072143 | 0.00075694 |
| k__Bacteria;p__Verrucomicrobia;c__[Spartobacteria];o__[Chthoniobacterales];f__[Chthoniobacteraceae];g__ | | 0 | 0 | 0 | 3.96E-05 | 0 | 0 | 0 | 0 | 0 | 0 | 0 | 0 | 0 | 0 | 0 |
| k__Bacteria;p__Verrucomicrobia;c__[Spartobacteria];o__[Chthoniobacterales];f__[Chthoniobacteraceae];g__Candidatus Xiphinematobacter | | 0 | 0 | 0 | 0 | 0 | 0 | 0 | 0 | 0 | 0.00297619 | 0 | 0 | 0 | 0 | 0 |
| k__Bacteria;p__Verrucomicrobia;c__[Spartobacteria];o__[Chthoniobacterales];f__[Chthoniobacteraceae];g__DA101 | | 0.00018755 | 0 | 0 | 0 | 0 | 0 | 0 | 0 | 0 | 0 | 0 | 0 | 0 | 0 | 0 |
| k__Bacteria;p__Verrucomicrobia;c__[Spartobacteria];o__[Chthoniobacterales];f__[Chthoniobacteraceae];g__Ellin506 | | 0 | 0 | 0 | 0 | 0 | 0 | 0 | 0 | 0 | 0 | 0 | 0 | 0 | 0 | 0 |
| k__Bacteria;p__WS3;c__PRR-12;o__GN03;f__KSB4;g__ | | 0 | 0 | 0 | 0 | 0 | 0 | 0 | 0 | 0 | 0 | 0 | 0 | 0 | 0 | 0 |
| k__Bacteria;p__WS3;c__PRR-12;o__Sediment-1;f__;g__ | | 0 | 0 | 0 | 0 | 0 | 0 | 0 | 0 | 0 | 0 | 0 | 0 | 0 | 0 | 0 |
| k__Bacteria;p__ZB3;c__BS119;o__;f__;g__ | | 0 | 0 | 0 | 0 | 0 | 0 | 0 | 0 | 0 | 0 | 0 | 0 | 0 | 0 | 0 |
| k__Bacteria;p__[Thermi];c__Deinococci;o__Deinococcales;f__Deinococcaceae;g__Deinococcus | | 0 | 0 | 0 | 0 | 0.00030381 | 0 | 0 | 0.00174014 | 0 | 0 | 0 | 0 | 0 | 0 | 0 |
| k__Bacteria;p__[Thermi];c__Deinococci;o__Deinococcales;f__Trueperaceae;g__Truepera | | 0 | 0 | 0 | 0 | 0 | 0 | 0 | 0 | 0 | 0 | 0 | 0 | 0 | 0 | 0 |
| k__Bacteria;p__[Thermi];c__Deinococci;o__Thermales;f__Thermaceae;g__Thermus | | 0 | 0 | 0 | 0 | 0 | 0.00029594 | 0 | 0 | 0 | 0 | 0 | 0 | 0 | 0 | 0 |

S3 Table. Genus-level sequence data shown as proportions of all sequences.

| Taxon | | 1231 | 1233 | 1234 | 1235 | 1236 | 1243 | 1244 | 6473 | 6474 | 1188 | 1189 | 1190 | 1226 | 1227 | 2027 |
|---|---|---|---|---|---|---|---|---|---|---|---|---|---|---|---|---|
| | Copepod genus | Calanus | Calanus | Calanus | Calanus | Calanus | Centropages | Centropages | Centropages | Centropages | Centropages | Centropages | Centropages | Centropages | Centropages | Centropages |
| | Type of sample | FullGut | FullGut | FullGut | FullGut | FullGut | FullGut | FullGut | FullGut | FullGut | Starved | Starved | Starved | Starved | Starved | Starved |
| k__Archaea;p__Crenarchaeota;c__Thaumarchaeota;o__Cenarchaeales;f__Cenarchaeaceae;g__Nitrosopumilus | | 0 | 0 | 0 | 0 | 0 | 0 | 0 | 0 | 0 | 0 | 0 | 0 | 0 | 0 | 0 |
| k__Archaea;p__Euryarchaeota;c__Thermoplasmata;o__E2;f__Marine group II;g__ | | 0 | 9.38E-05 | 5.04E-05 | 0 | 0 | 7.04E-05 | 0 | 0 | 0 | 0 | 0.00018455 | 0 | 0 | 0 | 0 |
| k__Bacteria;p__AC1;c__SHA-114;o__;f__;g__ | | 0 | 0 | 0 | 0 | 0 | 0 | 0 | 0 | 0 | 0 | 0 | 0 | 0 | 0 | 0 |
| k__Bacteria;p__Acidobacteria;c__;o__;f__;g__ | | 0 | 0 | 0 | 0 | 0 | 0 | 0 | 0 | 0 | 0 | 0 | 0 | 0 | 0 | 0 |
| k__Bacteria;p__Acidobacteria;c__Acidobacteria-6;o__iii1-15;f__;g__ | | 0 | 0 | 0 | 0 | 0 | 0.00014084 | 0 | 0 | 0 | 0 | 0 | 0 | 0 | 0 | 0 |
| k__Bacteria;p__Acidobacteria;c__Acidobacteriia;o__Acidobacteriales;f__Acidobacteriaceae;g__ | | 0 | 0 | 0 | 0 | 0 | 0 | 0 | 0 | 0 | 0 | 0 | 0 | 0 | 0 | 0 |
| k__Bacteria;p__Acidobacteria;c__Acidobacteriia;o__Acidobacteriales;f__Koribacteraceae;g__ | | 0 | 0 | 0 | 0.00103177 | 0 | 0.00035209 | 0 | 0 | 0 | 0 | 0 | 0 | 0 | 0 | 0 |
| k__Bacteria;p__Acidobacteria;c__Acidobacteriia;o__Acidobacteriales;f__Koribacteraceae;g__Candidatus Koribacter | | 0 | 0 | 0 | 0 | 0 | 0 | 0 | 0 | 0 | 0 | 0 | 0 | 0 | 0 | 0 |
| k__Bacteria;p__Acidobacteria;c__OS-K;o__;f__;g__ | | 0 | 0 | 0 | 0 | 0 | 0 | 0 | 0 | 0 | 0 | 0 | 0 | 0 | 0 | 0 |
| k__Bacteria;p__Acidobacteria;c__Solibacteres;o__Solibacterales;f__;g__ | | 9.34E-05 | 0 | 0.00026436 | 0 | 0 | 0 | 0 | 0 | 0 | 0 | 0 | 0 | 0.00130948 | 0 | 0 |
| k__Bacteria;p__Acidobacteria;c__Solibacteres;o__Solibacterales;f__PAUC26f;g__ | | 0 | 0 | 0 | 0 | 0 | 0 | 0 | 0 | 0 | 0 | 0 | 0 | 0 | 0 | 0 |
| k__Bacteria;p__Acidobacteria;c__Sva0725;o__Sva0725;f__;g__ | | 0 | 0 | 0 | 0 | 0 | 0 | 0 | 0 | 0 | 0 | 0 | 0 | 0 | 0 | 0 |
| k__Bacteria;p__Acidobacteria;c__[Chloracidobacteria];o__PK29;f__;g__ | | 0 | 0 | 0 | 0 | 0 | 0 | 0 | 0 | 0 | 0 | 0 | 0 | 5.78E-05 | 0.00053517 | 0 |
| k__Bacteria;p__Acidobacteria;c__[Chloracidobacteria];o__RB41;f__;g__ | | 0 | 0 | 0 | 0 | 0 | 0 | 0.00011141 | 0 | 0 | 0 | 0 | 0 | 0 | 0 | 0 |
| k__Bacteria;p__Actinobacteria;c__Acidimicrobiia;o__Acidimicrobiales;f__;g__ | | 0 | 0 | 0.00061684 | 0 | 0 | 0.00031688 | 0 | 0 | 0.00626602 | 0 | 0 | 0 | 0 | 0 | 0 |
| k__Bacteria;p__Actinobacteria;c__Acidimicrobiia;o__Acidimicrobiales;f__C111;g__ | | 0.00014671 | 0.00032162 | 0.00016365 | 0 | 0 | 0.00015844 | 0.00012378 | 0 | 0 | 0 | 0.00044292 | 0 | 0 | 0.00088508 | 1.64E-05 |
| k__Bacteria;p__Actinobacteria;c__Acidimicrobiia;o__Acidimicrobiales;f__JdFBGBact;g__ | | 0 | 0 | 0 | 0 | 0 | 0 | 0 | 0 | 0 | 0 | 0 | 0 | 0 | 0 | 0 |
| k__Bacteria;p__Actinobacteria;c__Acidimicrobiia;o__Acidimicrobiales;f__Microthrixaceae;g__ | | 0 | 0 | 0 | 0 | 0 | 0 | 0 | 0 | 0 | 0 | 0 | 0 | 0 | 0 | 0 |
| k__Bacteria;p__Actinobacteria;c__Acidimicrobiia;o__Acidimicrobiales;f__OCS155;g__ | | 0.00192059 | 0.00062984 | 0.00114556 | 0 | 0.0006418 | 0.00165481 | 0.00039611 | 0 | 0 | 0 | 0.00125494 | 0.00037045 | 7.70E-05 | 0.00117325 | 0.00040947 |
| k__Bacteria;p__Actinobacteria;c__Acidimicrobiia;o__Acidimicrobiales;f__SC3-41;g__ | | 0.00285421 | 0.00223795 | 0.00122109 | 0 | 0.00250302 | 0.00491163 | 0.00243854 | 0 | 0 | 0 | 1.85E-05 | 0 | 0 | 0 | 0.00398002 |
| k__Bacteria;p__Actinobacteria;c__Acidimicrobiia;o__Acidimicrobiales;f__TK06;g__ | | 0 | 0 | 0.00026436 | 0 | 0 | 0 | 0 | 0 | 0 | 0 | 0 | 0 | 0 | 0 | 0 |
| k__Bacteria;p__Actinobacteria;c__Acidimicrobiia;o__Acidimicrobiales;f__ZA3409c;g__ | | 0 | 0 | 0.00015106 | 0 | 0 | 0 | 0 | 0 | 0 | 0 | 9.23E-05 | 0 | 0 | 0 | 0 |
| k__Bacteria;p__Actinobacteria;c__Acidimicrobiia;o__Acidimicrobiales;f__koll13;g__ | | 0 | 0 | 0 | 0 | 0 | 0 | 0 | 0 | 0 | 0 | 0 | 0 | 0 | 0 | 0 |
| k__Bacteria;p__Actinobacteria;c__Acidimicrobiia;o__Acidimicrobiales;f__ntu14;g__ | | 0 | 0 | 0 | 0 | 0 | 0 | 0 | 0 | 0 | 0 | 0 | 0 | 0 | 0 | 0 |
| k__Bacteria;p__Actinobacteria;c__Acidimicrobiia;o__Acidimicrobiales;f__wb1_P06;g__ | | 2.67E-05 | 0.00013401 | 0 | 0 | 0.00011552 | 1.76E-05 | 8.66E-05 | 0 | 0 | 0 | 5.54E-05 | 0 | 0 | 0 | 6.55E-05 |
| k__Bacteria;p__Actinobacteria;c__Actinobacteria;o__Actinomycetales;f__;g__ | | 0.00036011 | 0.00084426 | 0 | 0.0015748 | 0.00015403 | 0.00137314 | 0 | 0 | 0 | 0.00079334 | 0.0005721 | 0 | 0 | 2.06E-05 | 0.00027844 |
| k__Bacteria;p__Actinobacteria;c__Actinobacteria;o__Actinomycetales;f__ACK-M1;g__ | | 0 | 0 | 0 | 0 | 0 | 0 | 0 | 0 | 0.00320422 | 0 | 0 | 0 | 0 | 0 | 0 |
| k__Bacteria;p__Actinobacteria;c__Actinobacteria;o__Actinomycetales;f__Actinomycetaceae;g__ | | 0 | 2.68E-05 | 0 | 0 | 0 | 0 | 0 | 0 | 0 | 0 | 0 | 0 | 0 | 0 | 0 |
| k__Bacteria;p__Actinobacteria;c__Actinobacteria;o__Actinomycetales;f__Actinomycetaceae;g__Actinomyces | | 9.34E-05 | 6.70E-05 | 0.00017624 | 0.00059734 | 1.28E-05 | 0 | 0 | 0.02581311 | 0 | 0 | 0.00018455 | 0 | 0 | 0 | 0 |
| k__Bacteria;p__Actinobacteria;c__Actinobacteria;o__Actinomycetales;f__Actinomycetaceae;g__Mobiluncus | | 0 | 0 | 0 | 0.00021721 | 0 | 0 | 0 | 0 | 0.00548277 | 0 | 0 | 0 | 0 | 0 | 0 |
| k__Bacteria;p__Actinobacteria;c__Actinobacteria;o__Actinomycetales;f__Actinomycetaceae;g__N09 | | 0 | 0 | 0 | 0.03383112 | 0 | 0 | 0 | 0 | 0 | 0.00061484 | 0 | 0 | 0 | 0 | 0 |
| k__Bacteria;p__Actinobacteria;c__Actinobacteria;o__Actinomycetales;f__Actinomycetaceae;g__Varibaculum | | 0 | 0 | 0 | 0 | 0 | 0 | 0 | 0 | 0 | 0 | 1.85E-05 | 0 | 0 | 0 | 0 |
| k__Bacteria;p__Actinobacteria;c__Actinobacteria;o__Actinomycetales;f__Brevibacteriaceae;g__Brevibacterium | | 0.00020006 | 0 | 0 | 0 | 0 | 0 | 0 | 0 | 0.00042723 | 0.00061484 | 0 | 0 | 0 | 0 | 0 |
| k__Bacteria;p__Actinobacteria;c__Actinobacteria;o__Actinomycetales;f__Cellulomonadaceae;g__Actinotalea | | 0 | 0 | 0 | 0 | 0 | 0 | 0 | 0 | 0 | 0 | 0 | 0 | 0 | 0 | 0 |
| k__Bacteria;p__Actinobacteria;c__Actinobacteria;o__Actinomycetales;f__Cellulomonadaceae;g__Cellulomonas | | 0 | 0 | 0 | 0 | 0 | 1.76E-05 | 0 | 0 | 0 | 0 | 0 | 0 | 0 | 0 | 0 |
| k__Bacteria;p__Actinobacteria;c__Actinobacteria;o__Actinomycetales;f__Cellulomonadaceae;g__Demequina | | 0 | 0 | 0 | 0 | 0 | 0 | 0 | 0 | 0 | 0 | 0 | 0 | 0 | 0 | 0 |
| k__Bacteria;p__Actinobacteria;c__Actinobacteria;o__Actinomycetales;f__Corynebacteriaceae;g__Corynebacterium | | 0.00058685 | 0.00143389 | 0.00095673 | 0.11588379 | 7.70E-05 | 0.00107387 | 0.00014854 | 0.00722767 | 0.05005696 | 0.00166601 | 0.00077511 | 0.00022381 | 0.0030041 | 0.00265525 | 3.28E-05 |
| k__Bacteria;p__Actinobacteria;c__Actinobacteria;o__Actinomycetales;f__Dermabacteraceae;g__Brachybacterium | | 0 | 0 | 0 | 0 | 0 | 0 | 0.00040849 | 0 | 0 | 0 | 0 | 0 | 0 | 0 | 0 |
| k__Bacteria;p__Actinobacteria;c__Actinobacteria;o__Actinomycetales;f__Dermacoccaceae;g__Dermacoccus | | 0 | 0 | 0 | 0 | 0 | 0 | 0 | 0 | 0 | 0 | 0 | 0 | 0 | 0 | 0 |
| k__Bacteria;p__Actinobacteria;c__Actinobacteria;o__Actinomycetales;f__Dietziaceae;g__Dietzia | | 0 | 0 | 0 | 0 | 5.13E-05 | 0 | 0 | 0 | 0 | 0 | 0 | 0 | 0 | 0 | 0 |
| k__Bacteria;p__Actinobacteria;c__Actinobacteria;o__Actinomycetales;f__Frankiaceae;g__ | | 0.00033344 | 0 | 0 | 0 | 0 | 0.00014084 | 0 | 0 | 0 | 0 | 0 | 0 | 0 | 0 | 0 |
| k__Bacteria;p__Actinobacteria;c__Actinobacteria;o__Actinomycetales;f__Geodermatophilaceae;g__ | | 0 | 0 | 0.00026436 | 0 | 0 | 0 | 0 | 0 | 0 | 0 | 0 | 0 | 0 | 0 | 0 |
| k__Bacteria;p__Actinobacteria;c__Actinobacteria;o__Actinomycetales;f__Geodermatophilaceae;g__Blastococcus | | 0 | 0 | 0 | 0 | 0 | 0 | 0 | 0 | 0 | 0 | 0 | 0 | 0 | 0 | 0 |
| k__Bacteria;p__Actinobacteria;c__Actinobacteria;o__Actinomycetales;f__Gordoniaceae;g__Gordonia | | 0 | 0 | 6.29E-05 | 0 | 0 | 0 | 0 | 0 | 0 | 0 | 0 | 0 | 0 | 0 | 0 |
| k__Bacteria;p__Actinobacteria;c__Actinobacteria;o__Actinomycetales;f__Intrasporangiaceae;g__ | | 0 | 0 | 0 | 0 | 0 | 0 | 0 | 0 | 0 | 0 | 1.85E-05 | 0 | 0 | 0 | 0 |
| k__Bacteria;p__Actinobacteria;c__Actinobacteria;o__Actinomycetales;f__Intrasporangiaceae;g__Knoellia | | 0 | 0 | 0 | 0 | 0 | 0 | 0 | 0.00051626 | 0 | 0 | 0 | 0 | 0 | 0 | 0 |
| k__Bacteria;p__Actinobacteria;c__Actinobacteria;o__Actinomycetales;f__Intrasporangiaceae;g__Oryzihumus | | 0 | 0 | 0 | 0 | 0 | 0 | 0 | 0 | 0 | 0 | 0 | 0 | 0 | 0 | 0 |
| k__Bacteria;p__Actinobacteria;c__Actinobacteria;o__Actinomycetales;f__Intrasporangiaceae;g__Phycicoccus | | 0 | 0 | 0 | 0 | 0 | 1.76E-05 | 0 | 0.01600413 | 0 | 0 | 0.00016609 | 0 | 0 | 0 | 0 |
| k__Bacteria;p__Actinobacteria;c__Actinobacteria;o__Actinomycetales;f__Intrasporangiaceae;g__Tetrasphaera | | 0 | 0 | 0 | 0 | 0 | 0 | 0 | 0 | 0 | 0 | 0 | 0 | 0 | 0 | 0 |
| k__Bacteria;p__Actinobacteria;c__Actinobacteria;o__Actinomycetales;f__Kineosporiaceae;g__ | | 0 | 0 | 0 | 0 | 0 | 0 | 0 | 0 | 0 | 0 | 0 | 0 | 0 | 0 | 0 |
| k__Bacteria;p__Actinobacteria;c__Actinobacteria;o__Actinomycetales;f__Microbacteriaceae;g__ | | 0.00034677 | 0.00060304 | 1.26E-05 | 0.01688841 | 0.00020538 | 0.0008098 | 0.000458 | 0.01290656 | 0.05561094 | 0.00150734 | 0.00018455 | 0.00013892 | 0 | 0 | 0.00021292 |
| k__Bacteria;p__Actinobacteria;c__Actinobacteria;o__Actinomycetales;f__Microbacteriaceae;g__Agrococcus | | 0 | 0 | 0 | 0 | 0 | 0 | 0 | 0 | 0 | 0 | 0 | 0 | 0 | 0 | 0 |
| k__Bacteria;p__Actinobacteria;c__Actinobacteria;o__Actinomycetales;f__Microbacteriaceae;g__Candidatus Aquiluna | | 0.00096029 | 0.00020101 | 0 | 0 | 0.00057762 | 0.00140835 | 0.00080459 | 0 | 0 | 0.00329234 | 0.00274979 | 0.00016979 | 0 | 0 | 0.00072066 |
| k__Bacteria;p__Actinobacteria;c__Actinobacteria;o__Actinomycetales;f__Microbacteriaceae;g__Candidatus Rhodoluna | | 0 | 0 | 0 | 0 | 0 | 0 | 0 | 0 | 0 | 0 | 0 | 0 | 0 | 0 | 0 |
| k__Bacteria;p__Actinobacteria;c__Actinobacteria;o__Actinomycetales;f__Microbacteriaceae;g__Cryocola | | 0 | 0 | 0 | 0 | 0 | 0 | 0 | 0 | 0 | 0 | 0 | 0 | 0 | 0 | 0 |
| k__Bacteria;p__Actinobacteria;c__Actinobacteria;o__Actinomycetales;f__Microbacteriaceae;g__Curtobacterium | | 0 | 0 | 0 | 0 | 0 | 0 | 0 | 0 | 0.00042723 | 0 | 0 | 0 | 0 | 0 | 0 |
| k__Bacteria;p__Actinobacteria;c__Actinobacteria;o__Actinomycetales;f__Microbacteriaceae;g__Frigoribacterium | | 0 | 0 | 0 | 0 | 0 | 0 | 0 | 0 | 0 | 0 | 0 | 0 | 0 | 0 | 0 |
| k__Bacteria;p__Actinobacteria;c__Actinobacteria;o__Actinomycetales;f__Microbacteriaceae;g__Herbiconiux | | 0 | 0 | 0 | 0 | 0 | 0 | 0 | 0 | 0 | 0 | 0 | 0 | 0 | 0.0001235 | 0 |
| k__Bacteria;p__Actinobacteria;c__Actinobacteria;o__Actinomycetales;f__Microbacteriaceae;g__Microbacterium | | 0 | 4.02E-05 | 0.00018883 | 0.00168341 | 0 | 0.00015844 | 0 | 0 | 0 | 0 | 5.54E-05 | 0 | 0 | 0 | 1.64E-05 |
| k__Bacteria;p__Actinobacteria;c__Actinobacteria;o__Actinomycetales;f__Microbacteriaceae;g__Mycetocola | | 0 | 0 | 0 | 0 | 0 | 0 | 0 | 0 | 0 | 0 | 0 | 0 | 0 | 2.06E-05 | 0 |
| k__Bacteria;p__Actinobacteria;c__Actinobacteria;o__Actinomycetales;f__Microbacteriaceae;g__Pseudoclavibacter | | 0 | 0 | 0 | 0 | 0 | 0 | 0 | 0 | 0 | 0 | 0 | 0 | 0 | 0 | 0 |
| k__Bacteria;p__Actinobacteria;c__Actinobacteria;o__Actinomycetales;f__Microbacteriaceae;g__Salinibacterium | | 0 | 0 | 0 | 0 | 0 | 0 | 0 | 0 | 0 | 0 | 0 | 0 | 0 | 0 | 0 |
| k__Bacteria;p__Actinobacteria;c__Actinobacteria;o__Actinomycetales;f__Microbacteriaceae;g__Yonghaparkia | | 0 | 0 | 0 | 0 | 0 | 0 | 0 | 0 | 0 | 0 | 0 | 0 | 0 | 0 | 0 |
| k__Bacteria;p__Actinobacteria;c__Actinobacteria;o__Actinomycetales;f__Micrococcaceae;g__ | | 0 | 0 | 0 | 0 | 0 | 0.00010563 | 0 | 0 | 0.01972373 | 0.00093217 | 5.54E-05 | 0 | 0 | 0 | 1.64E-05 |
| k__Bacteria;p__Actinobacteria;c__Actinobacteria;o__Actinomycetales;f__Micrococcaceae;g__Arthrobacter | | 0 | 0 | 0 | 0 | 0 | 7.04E-05 | 0.00012378 | 0 | 0 | 0 | 0 | 0 | 0 | 0 | 0 |
| k__Bacteria;p__Actinobacteria;c__Actinobacteria;o__Actinomycetales;f__Micrococcaceae;g__Kocuria | | 0.00022674 | 0 | 0 | 0 | 0 | 0 | 0 | 0 | 0.00904301 | 0 | 0.00038755 | 0 | 0 | 0 | 1.64E-05 |
| k__Bacteria;p__Actinobacteria;c__Actinobacteria;o__Actinomycetales;f__Micrococcaceae;g__Microbispora | | 0 | 0 | 0 | 0 | 0 | 0.00014084 | 0 | 0 | 0 | 0 | 0 | 0 | 0 | 0 | 0 |
| k__Bacteria;p__Actinobacteria;c__Actinobacteria;o__Actinomycetales;f__Micrococcaceae;g__Micrococcus | | 0 | 0 | 0 | 0 | 0 | 1.76E-05 | 0 | 0 | 0.00028482 | 0 | 0 | 0 | 0 | 0 | 0 |
| k__Bacteria;p__Actinobacteria;c__Actinobacteria;o__Actinomycetales;f__Micrococcaceae;g__Renibacterium | | 0 | 4.02E-05 | 0 | 0 | 0 | 0 | 0 | 0 | 0 | 0 | 0 | 0 | 0 | 0 | 0 |
| k__Bacteria;p__Actinobacteria;c__Actinobacteria;o__Actinomycetales;f__Micrococcaceae;g__Rothia | | 0.00012004 | 0 | 0 | 0.00173771 | 1.28E-05 | 0 | 0 | 0 | 0.00947024 | 0 | 0 | 0 | 0 | 0 | 0 |
| k__Bacteria;p__Actinobacteria;c__Actinobacteria;o__Actinomycetales;f__Micromonosporaceae;g__ | | 0 | 0 | 0 | 0 | 0 | 0 | 0 | 0 | 0 | 0 | 0 | 0 | 0 | 0 | 0 |
| k__Bacteria;p__Actinobacteria;c__Actinobacteria;o__Actinomycetales;f__Micromonosporaceae;g__Phytohabitans | | 0 | 0 | 0 | 0 | 0 | 0 | 0 | 0 | 0 | 0 | 0.00014764 | 0 | 0 | 0 | 0 |
| k__Bacteria;p__Actinobacteria;c__Actinobacteria;o__Actinomycetales;f__Micromonosporaceae;g__Verrucosispora | | 0 | 0 | 0 | 0 | 0 | 0 | 0 | 0 | 0 | 0 | 0 | 0 | 0 | 0 | 0 |

| Taxon | 1231 | 1233 | 1234 | 1235 | 1236 | 1243 | 1244 | 6473 | 6474 | 1188 | 1189 | 1190 | 1226 | 1227 | 2027 |
|---|---|---|---|---|---|---|---|---|---|---|---|---|---|---|---|
| Copepod genus | Calanus | Calanus | Calanus | Calanus | Calanus | Centropages | Centropages | Centropages | Centropages | Centropages | Centropages | Centropages | Centropages | Centropages | Centropages |
| Type of sample | FullGut | FullGut | FullGut | FullGut | FullGut | FullGut | FullGut | FullGut | FullGut | Starved | Starved | Starved | Starved | Starved | Starved |
| k__Bacteria;p__Actinobacteria;c__Actinobacteria;o__Actinomycetales;f__Mycobacteriaceae;g__Mycobacterium | 0 | 0 | 0 | 0 | 0 | 3.52E-05 | 0 | 0 | 0 | 0 | 0 | 0 | 0 | 0 | 0 |
| k__Bacteria;p__Actinobacteria;c__Actinobacteria;o__Actinomycetales;f__Nakamurellaceae;g__ | 0 | 0 | 0 | 0 | 0 | 0 | 0 | 0 | 0.00099687 | 0 | 0 | 0 | 0 | 0 | 0 |
| k__Bacteria;p__Actinobacteria;c__Actinobacteria;o__Actinomycetales;f__Nocardiaceae;g__ | 0.00016005 | 0 | 0 | 0 | 0 | 0 | 0 | 0 | 0 | 0 | 0 | 0 | 0 | 0 | 0 |
| k__Bacteria;p__Actinobacteria;c__Actinobacteria;o__Actinomycetales;f__Nocardiaceae;g__Rhodococcus | 6.67E-05 | 0 | 0 | 0 | 5.13E-05 | 0 | 0 | 0 | 0 | 0 | 0 | 0 | 0 | 0 | 0 |
| k__Bacteria;p__Actinobacteria;c__Actinobacteria;o__Actinomycetales;f__Nocardioidaceae;g__ | 0 | 0 | 0 | 0.00059734 | 2.57E-05 | 0 | 0 | 0 | 0 | 0.0006545 | 0 | 0 | 0.00065474 | 0 | 1.64E-05 |
| k__Bacteria;p__Actinobacteria;c__Actinobacteria;o__Actinomycetales;f__Nocardioidaceae;g__Aeromicrobium | 0 | 0 | 0 | 0 | 0 | 0 | 0 | 0 | 0 | 0 | 0 | 0 | 0.00142502 | 0 | 0 |
| k__Bacteria;p__Actinobacteria;c__Actinobacteria;o__Actinomycetales;f__Nocardioidaceae;g__Friedmanniella | 0 | 0 | 0 | 0 | 0 | 0 | 0 | 0 | 0 | 0 | 1.85E-05 | 0 | 0 | 0 | 0 |
| k__Bacteria;p__Actinobacteria;c__Actinobacteria;o__Actinomycetales;f__Nocardioidaceae;g__Nocardioides | 0 | 0 | 0 | 0 | 0 | 8.80E-05 | 0 | 0 | 0 | 0 | 0 | 0 | 0 | 0 | 0 |
| k__Bacteria;p__Actinobacteria;c__Actinobacteria;o__Actinomycetales;f__Nocardioidaceae;g__Propionicimonas | 0 | 0 | 0 | 0 | 0 | 5.28E-05 | 0 | 0 | 0.00341783 | 0 | 0 | 0 | 0 | 0 | 0 |
| k__Bacteria;p__Actinobacteria;c__Actinobacteria;o__Actinomycetales;f__Promicromonosporaceae;g__Promicromonospora | 5.33E-05 | 0 | 0 | 0 | 0 | 0 | 0 | 0 | 0 | 0 | 0 | 0 | 0 | 0 | 0 |
| k__Bacteria;p__Actinobacteria;c__Actinobacteria;o__Actinomycetales;f__Propionibacteriaceae;g__ | 0 | 0 | 0 | 0.00054304 | 0 | 0 | 0 | 0 | 0 | 0 | 0 | 0 | 0 | 0 | 0 |
| k__Bacteria;p__Actinobacteria;c__Actinobacteria;o__Actinomycetales;f__Propionibacteriaceae;g__Propionibacterium | 0.00089361 | 0.00062984 | 0.00079308 | 0.00559327 | 0.00021821 | 0.00070418 | 0.00025995 | 0.06608157 | 0.09171176 | 0.00160651 | 0.00079356 | 0 | 0.00342776 | 0.00277875 | 3.28E-05 |
| k__Bacteria;p__Actinobacteria;c__Actinobacteria;o__Actinomycetales;f__Pseudonocardiaceae;g__Pseudonocardia | 8.00E-05 | 0 | 0 | 0 | 0 | 0 | 0 | 0 | 0 | 0 | 0 | 0 | 0 | 0 | 0 |
| k__Bacteria;p__Actinobacteria;c__Actinobacteria;o__Actinomycetales;f__Pseudonocardiaceae;g__Saccharopolyspora | 0 | 0 | 0 | 0.00222645 | 0 | 0 | 0 | 0 | 0 | 0 | 0 | 0 | 0 | 0 | 0 |
| k__Bacteria;p__Actinobacteria;c__Actinobacteria;o__Actinomycetales;f__Sanguibacteraceae;g__Sanguibacter | 0 | 0 | 0 | 0 | 0 | 0 | 0 | 0 | 0 | 0 | 0 | 0 | 0 | 0 | 0 |
| k__Bacteria;p__Actinobacteria;c__Actinobacteria;o__Actinomycetales;f__Sporichthyaceae;g__ | 0 | 5.36E-05 | 0 | 0 | 0 | 0 | 0 | 0 | 0 | 0 | 0 | 0 | 0 | 0 | 0 |
| k__Bacteria;p__Actinobacteria;c__Actinobacteria;o__Actinomycetales;f__Streptomycetaceae;g__ | 0 | 0 | 0 | 0 | 0 | 0 | 0 | 0 | 0 | 1.98E-05 | 0 | 0 | 0 | 0 | 0 |
| k__Bacteria;p__Actinobacteria;c__Actinobacteria;o__Actinomycetales;f__Williamsiaceae;g__Williamsia | 0 | 0 | 0 | 0.00065164 | 0 | 0 | 0 | 0 | 0 | 0 | 0 | 0 | 0 | 0 | 0 |
| k__Bacteria;p__Actinobacteria;c__Actinobacteria;o__Actinomycetales;f__Yaniellaceae;g__Auritibacter | 0 | 0 | 0 | 0 | 0 | 0 | 0 | 0 | 0 | 0 | 0 | 0 | 0 | 0 | 0 |
| k__Bacteria;p__Actinobacteria;c__Actinobacteria;o__Bifidobacteriales;f__Bifidobacteriaceae;g__ | 0 | 0 | 0 | 0 | 0 | 0 | 0 | 0 | 0 | 0.00031733 | 0 | 0 | 0 | 0 | 0 |
| k__Bacteria;p__Actinobacteria;c__Actinobacteria;o__Bifidobacteriales;f__Bifidobacteriaceae;g__Alloscardovia | 0 | 0 | 0 | 0 | 0 | 0 | 0 | 0 | 0 | 0.0001785 | 0 | 0 | 0 | 0 | 0 |
| k__Bacteria;p__Actinobacteria;c__Actinobacteria;o__Bifidobacteriales;f__Bifidobacteriaceae;g__Bifidobacterium | 0 | 0 | 0 | 0 | 0 | 0 | 0 | 0 | 0 | 0.0002975 | 0 | 0 | 0 | 0 | 0 |
| k__Bacteria;p__Actinobacteria;c__Actinobacteria;o__Bifidobacteriales;f__Bifidobacteriaceae;g__Gardnerella | 0 | 0 | 0 | 0 | 0 | 0 | 0 | 0 | 0 | 0.0001785 | 0 | 0 | 0 | 0 | 0 |
| k__Bacteria;p__Actinobacteria;c__Actinobacteria;o__Bifidobacteriales;f__Bifidobacteriaceae;g__Scardovia | 0 | 0 | 0 | 0 | 0 | 0 | 0 | 0 | 0 | 0 | 5.54E-05 | 0 | 0 | 0 | 0 |
| k__Bacteria;p__Actinobacteria;c__Actinobacteria;o__WCHB1-81;f__At425_EubF1;g__ | 0 | 0 | 0 | 0 | 0 | 0 | 0 | 0 | 0 | 0 | 0 | 0 | 0 | 0 | 0 |
| k__Bacteria;p__Actinobacteria;c__Coriobacteriia;o__Coriobacteriales;f__Coriobacteriaceae;g__Atopobium | 0 | 0 | 0 | 0 | 0 | 0 | 0 | 0 | 0 | 0 | 0 | 0 | 0 | 0 | 0 |
| k__Bacteria;p__Actinobacteria;c__Rubrobacteria;o__Rubrobacterales;f__Rubrobacteraceae;g__Rubrobacter | 0 | 0 | 0 | 0 | 0 | 0 | 0 | 0 | 0 | 0 | 0 | 0 | 0 | 0 | 0 |
| k__Bacteria;p__Actinobacteria;c__Thermoleophilia;o__Gaiellales;f__Gaiellaceae;g__ | 0 | 0 | 0 | 0 | 0 | 0 | 0 | 0 | 0 | 0 | 0 | 0 | 0 | 0 | 0 |
| k__Bacteria;p__Actinobacteria;c__Thermoleophilia;o__Solirubrobacterales;f__Conexibacteraceae;g__ | 0 | 0 | 0 | 0 | 0 | 0 | 2.48E-05 | 0 | 0 | 0 | 0 | 0 | 0 | 0 | 0 |
| k__Bacteria;p__Actinobacteria;c__Thermoleophilia;o__Solirubrobacterales;f__Patulibacteraceae;g__ | 0 | 0.00014741 | 0 | 0 | 0 | 0 | 0 | 0 | 0 | 0 | 0 | 0 | 0 | 0 | 0 |
| k__Bacteria;p__Actinobacteria;c__Thermoleophilia;o__Solirubrobacterales;f__Solirubrobacteraceae;g__ | 0 | 0 | 0 | 0 | 0 | 0 | 0 | 0 | 0 | 0 | 0 | 0 | 0 | 0 | 0 |
| k__Bacteria;p__Actinobacteria;c__Thermoleophilia;o__Solirubrobacterales;f__Solirubrobacteraceae;g__Solirubrobacter | 0 | 0 | 0 | 0 | 0 | 5.28E-05 | 0 | 0 | 0 | 0 | 0 | 0 | 0 | 0 | 0 |
| k__Bacteria;p__Bacteroidetes;c__Bacteroidia;o__Bacteroidales;f__;g__ | 0 | 0 | 0 | 0 | 0 | 0.00012323 | 0 | 0 | 0 | 0 | 0 | 0 | 0 | 0 | 0 |
| k__Bacteria;p__Bacteroidetes;c__Bacteroidia;o__Bacteroidales;f__Bacteroidaceae;g__Bacteroides | 0 | 0 | 0 | 0 | 0 | 0 | 0 | 0 | 0 | 0 | 0 | 0 | 0 | 0 | 0 |
| k__Bacteria;p__Bacteroidetes;c__Bacteroidia;o__Bacteroidales;f__Porphyromonadaceae;g__ | 0 | 0 | 0 | 0.00781971 | 0 | 7.04E-05 | 0 | 0 | 0 | 0 | 0 | 0 | 0 | 0 | 0 |
| k__Bacteria;p__Bacteroidetes;c__Bacteroidia;o__Bacteroidales;f__Porphyromonadaceae;g__Paludibacter | 0 | 2.68E-05 | 0 | 0 | 0 | 0 | 0 | 0 | 0 | 0 | 0 | 0 | 0 | 0 | 0 |
| k__Bacteria;p__Bacteroidetes;c__Bacteroidia;o__Bacteroidales;f__Porphyromonadaceae;g__Parabacteroides | 0 | 0 | 0 | 0 | 0 | 0 | 0 | 0 | 0 | 0 | 0 | 0 | 0 | 0 | 0 |
| k__Bacteria;p__Bacteroidetes;c__Bacteroidia;o__Bacteroidales;f__Porphyromonadaceae;g__Porphyromonas | 0.00056017 | 0.00017421 | 8.81E-05 | 0.00070595 | 0 | 5.28E-05 | 0 | 0.03613836 | 0.01352891 | 0.00134867 | 0 | 0 | 0.00125171 | 0.00115267 | 8.19E-05 |
| k__Bacteria;p__Bacteroidetes;c__Bacteroidia;o__Bacteroidales;f__Prevotellaceae;g__Prevotella | 0 | 0.00010721 | 0 | 0.01449905 | 0 | 0 | 0 | 0 | 0 | 0 | 0 | 0.00011577 | 0.00096285 | 0 | 0 |
| k__Bacteria;p__Bacteroidetes;c__Bacteroidia;o__Bacteroidales;f__Rikenellaceae;g__ | 0 | 0 | 0 | 0 | 0 | 0 | 0 | 0 | 0 | 0 | 0 | 0 | 0 | 0 | 0 |
| k__Bacteria;p__Bacteroidetes;c__Bacteroidia;o__Bacteroidales;f__SB-1;g__ | 0 | 0 | 0 | 0 | 0 | 0 | 0 | 0 | 0 | 0 | 0 | 0 | 0 | 0 | 0 |
| k__Bacteria;p__Bacteroidetes;c__Bacteroidia;o__Bacteroidales;f__[Odoribacteraceae];g__Butyricimonas | 0 | 0 | 0 | 0 | 0 | 0 | 0 | 0 | 0 | 0 | 0 | 0 | 0 | 0 | 0 |
| k__Bacteria;p__Bacteroidetes;c__Bacteroidia;o__Bacteroidales;f__[Odoribacteraceae];g__Odoribacter | 0 | 0 | 0 | 0 | 0 | 0 | 0 | 0 | 0 | 0 | 0 | 0 | 0 | 0 | 0 |
| k__Bacteria;p__Bacteroidetes;c__Bacteroidia;o__Bacteroidales;f__[Paraprevotellaceae];g__[Prevotella] | 0 | 0 | 0 | 0 | 0 | 0 | 0 | 0 | 0 | 0 | 0 | 0 | 0 | 0 | 0 |
| k__Bacteria;p__Bacteroidetes;c__Cytophagia;o__Cytophagales;f__Cyclobacteriaceae;g__ | 0 | 0 | 0 | 0 | 0 | 0 | 0 | 0 | 0 | 0 | 0 | 0 | 0 | 0 | 0 |
| k__Bacteria;p__Bacteroidetes;c__Cytophagia;o__Cytophagales;f__Cytophagaceae;g__ | 0 | 0 | 0 | 0 | 0 | 0 | 0 | 0 | 0 | 0 | 0 | 0 | 0 | 0 | 0 |
| k__Bacteria;p__Bacteroidetes;c__Cytophagia;o__Cytophagales;f__Cytophagaceae;g__Adhaeribacter | 0 | 0 | 0 | 0 | 0 | 0.00051053 | 0 | 0 | 0 | 0 | 0 | 0 | 0 | 0 | 0 |
| k__Bacteria;p__Bacteroidetes;c__Cytophagia;o__Cytophagales;f__Cytophagaceae;g__Cytophaga | 0 | 0 | 0 | 0 | 0 | 0 | 0 | 0 | 0 | 0 | 0 | 0 | 0 | 0 | 0 |
| k__Bacteria;p__Bacteroidetes;c__Cytophagia;o__Cytophagales;f__Cytophagaceae;g__Hymenobacter | 0 | 0 | 0.00028954 | 0 | 0 | 0 | 0 | 0 | 0 | 0 | 0 | 0 | 0 | 0 | 0 |
| k__Bacteria;p__Bacteroidetes;c__Cytophagia;o__Cytophagales;f__Cytophagaceae;g__Leadbetterella | 0 | 0 | 0 | 0 | 0 | 0.00010563 | 0 | 0 | 0 | 0 | 0 | 0 | 0 | 0 | 0 |
| k__Bacteria;p__Bacteroidetes;c__Cytophagia;o__Cytophagales;f__Cytophagaceae;g__Spirosoma | 0 | 0 | 0 | 0 | 0 | 0 | 0 | 0 | 0 | 0 | 0 | 0 | 0 | 0 | 0 |
| k__Bacteria;p__Bacteroidetes;c__Cytophagia;o__Cytophagales;f__Flammeovirgaceae;g__ | 0.002174 | 0.00104527 | 0.00081826 | 0 | 0.00233615 | 0.00172523 | 0.00222811 | 0 | 0 | 0.0011305 | 0.0003691 | 0 | 0.00065474 | 0.00100858 | 0.00067153 |
| k__Bacteria;p__Bacteroidetes;c__Cytophagia;o__Cytophagales;f__Flammeovirgaceae;g__JTB248 | 0 | 0.00044223 | 0 | 0 | 0 | 0 | 0 | 0 | 0 | 0 | 0 | 0 | 0 | 0 | 0 |
| k__Bacteria;p__Bacteroidetes;c__Cytophagia;o__Cytophagales;f__Flammeovirgaceae;g__Roseivirga | 0 | 0 | 0 | 0 | 0 | 0 | 0 | 0 | 0 | 0 | 0 | 0 | 0 | 0 | 6.55E-05 |
| k__Bacteria;p__Bacteroidetes;c__Cytophagia;o__Cytophagales;f__[Amoebophilaceae];g__SC3-56 | 0 | 0 | 0 | 0 | 0 | 0 | 0 | 0 | 0 | 0 | 0 | 0 | 0 | 0 | 0 |
| k__Bacteria;p__Bacteroidetes;c__Flavobacteriia;o__Flavobacteriales;f__;g__ | 0.00444136 | 0.0046501 | 0.00196382 | 0 | 0.00408184 | 0.00468277 | 0.00193103 | 0 | 0 | 0.00355018 | 0.00749271 | 0.0029636 | 0 | 0.00212008 | 0.00288265 |
| k__Bacteria;p__Bacteroidetes;c__Flavobacteriia;o__Flavobacteriales;f__Cryomorphaceae;g__ | 0.0006402 | 0.00089786 | 0.00070496 | 0 | 0.00240033 | 0.00580945 | 0.00362687 | 0 | 7.12E-05 | 0.00583102 | 0.01345366 | 0.00661408 | 0.00140577 | 2.06E-05 | 0.00108099 |
| k__Bacteria;p__Bacteroidetes;c__Flavobacteriia;o__Flavobacteriales;f__Cryomorphaceae;g__Crocinitomix | 0.00061352 | 0.00095146 | 0 | 0 | 0.00109106 | 0.00061615 | 0.00071795 | 0 | 0 | 0 | 0 | 0 | 0 | 0 | 0.00026206 |
| k__Bacteria;p__Bacteroidetes;c__Flavobacteriia;o__Flavobacteriales;f__Cryomorphaceae;g__Fluviicola | 0.0066287 | 0.00545416 | 0.00523685 | 0.0015205 | 0.00559649 | 0.0128336 | 0.00537222 | 0 | 0 | 0 | 0.00226996 | 0.0002624 | 0.0010784 | 0 | 0.00471706 |
| k__Bacteria;p__Bacteroidetes;c__Flavobacteriia;o__Flavobacteriales;f__Flavobacteriaceae;g__ | 0.04634755 | 0.02334432 | 0.01207246 | 0.00124898 | 0.04823762 | 0.07719527 | 0.08078132 | 0.0232318 | 0.02399601 | 0.01842523 | 0.03382793 | 0.02157874 | 0.00591192 | 0.02768458 | 0.0469249 |
| k__Bacteria;p__Bacteroidetes;c__Flavobacteriia;o__Flavobacteriales;f__Flavobacteriaceae;g__Algibacter | 0 | 0 | 0 | 0 | 0 | 0 | 0 | 0 | 0 | 0 | 0 | 0 | 0 | 0.00366383 | 0 |
| k__Bacteria;p__Bacteroidetes;c__Flavobacteriia;o__Flavobacteriales;f__Flavobacteriaceae;g__Aquimarina | 0 | 0 | 0 | 0 | 0 | 0 | 0 | 0 | 0 | 0 | 0 | 0 | 0 | 0 | 0 |
| k__Bacteria;p__Bacteroidetes;c__Flavobacteriia;o__Flavobacteriales;f__Flavobacteriaceae;g__Bizionia | 0 | 0 | 0 | 0 | 0 | 0 | 0 | 0 | 0 | 0 | 0 | 0 | 0 | 0 | 0 |
| k__Bacteria;p__Bacteroidetes;c__Flavobacteriia;o__Flavobacteriales;f__Flavobacteriaceae;g__Capnocytophaga | 0 | 0.00010721 | 0 | 0 | 0 | 0 | 0 | 0 | 0 | 0 | 0 | 0.00010805 | 0 | 0 | 0 |
| k__Bacteria;p__Bacteroidetes;c__Flavobacteriia;o__Flavobacteriales;f__Flavobacteriaceae;g__Cellulophaga | 0 | 0 | 0 | 0 | 0 | 0 | 1.24E-05 | 0 | 0 | 0 | 0 | 0 | 0 | 0 | 0 |
| k__Bacteria;p__Bacteroidetes;c__Flavobacteriia;o__Flavobacteriales;f__Flavobacteriaceae;g__Coccinimonas | 0 | 0 | 0 | 0 | 0 | 0 | 0 | 0 | 0 | 0 | 0 | 0 | 0 | 0 | 0 |
| k__Bacteria;p__Bacteroidetes;c__Flavobacteriia;o__Flavobacteriales;f__Flavobacteriaceae;g__Flavobacterium | 0.00806914 | 0.00704886 | 0.00349963 | 0 | 0.01379868 | 0.03274417 | 0.01814671 | 0 | 0.00633723 | 0.00953987 | 0.01203263 | 0.00926126 | 0.01030253 | 0.02286808 | 0.01439686 |
| k__Bacteria;p__Bacteroidetes;c__Flavobacteriia;o__Flavobacteriales;f__Flavobacteriaceae;g__Formosa | 0 | 0 | 0 | 0 | 0 | 0 | 0 | 0 | 0 | 0 | 0 | 0 | 0 | 0 | 0 |
| k__Bacteria;p__Bacteroidetes;c__Flavobacteriia;o__Flavobacteriales;f__Flavobacteriaceae;g__Gaetbulibacter | 0 | 0 | 0 | 0 | 0 | 0 | 0 | 0 | 0 | 0 | 0 | 0 | 0 | 0 | 0 |
| k__Bacteria;p__Bacteroidetes;c__Flavobacteriia;o__Flavobacteriales;f__Flavobacteriaceae;g__Gillisia | 0 | 0 | 0 | 0 | 0 | 0.00077459 | 0 | 0 | 0 | 0 | 0 | 0 | 0 | 0 | 8.19E-05 |
| k__Bacteria;p__Bacteroidetes;c__Flavobacteriia;o__Flavobacteriales;f__Flavobacteriaceae;g__Gilvibacter | 0 | 0 | 0 | 0 | 0 | 0 | 0 | 0 | 0 | 0 | 0 | 0 | 0 | 0 | 0 |
| k__Bacteria;p__Bacteroidetes;c__Flavobacteriia;o__Flavobacteriales;f__Flavobacteriaceae;g__Gramella | 0 | 0 | 0 | 0 | 0 | 0.00059855 | 0 | 0 | 0 | 0 | 0 | 0 | 0 | 0 | 9.83E-05 |
| k__Bacteria;p__Bacteroidetes;c__Flavobacteriia;o__Flavobacteriales;f__Flavobacteriaceae;g__Kordia | 0 | 0 | 0 | 0 | 0 | 0 | 0 | 0 | 0 | 0 | 0 | 0 | 0 | 0 | 0 |
| k__Bacteria;p__Bacteroidetes;c__Flavobacteriia;o__Flavobacteriales;f__Flavobacteriaceae;g__Krokinobacter | 0 | 0 | 0 | 0 | 0 | 0.00132033 | 0 | 0 | 0 | 0 | 0 | 0 | 0 | 0 | 0.00024568 |

| Taxon | 1231 | 1233 | 1234 | 1235 | 1236 | 1243 | 1244 | 6473 | 6474 | 1188 | 1189 | 1190 | 1226 | 1227 | 2027 |
|---|---|---|---|---|---|---|---|---|---|---|---|---|---|---|---|
| Copepod genus | Calanus | Calanus | Calanus | Calanus | Calanus | Centropages | Centropages | Centropages | Centropages | Centropages | Centropages | Centropages | Centropages | Centropages | Centropages |
| Type of sample | FullGut | FullGut | FullGut | FullGut | FullGut | FullGut | FullGut | FullGut | FullGut | Starved | Starved | Starved | Starved | Starved | Starved |
| k__Bacteria;p__Bacteroidetes;c__Flavobacteriia;o__Flavobacteriales;f__Flavobacteriaceae;g__Lacinutrix | 0 | 0 | 0 | 0 | 1.28E-05 | 3.52E-05 | 0.00013616 | 0 | 0 | 0 | 0 | 7.72E-06 | 0 | 0 | 1.64E-05 |
| k__Bacteria;p__Bacteroidetes;c__Flavobacteriia;o__Flavobacteriales;f__Flavobacteriaceae;g__Leeuwenhoekiella | 0 | 0 | 0.00067978 | 0 | 0 | 0.00100345 | 0 | 0 | 0 | 0 | 0 | 0 | 0 | 0 | 6.55E-05 |
| k__Bacteria;p__Bacteroidetes;c__Flavobacteriia;o__Flavobacteriales;f__Flavobacteriaceae;g__Lutimonas | 0 | 0 | 0 | 0 | 0 | 0 | 0 | 0 | 0 | 0 | 0 | 0 | 0 | 0 | 0 |
| k__Bacteria;p__Bacteroidetes;c__Flavobacteriia;o__Flavobacteriales;f__Flavobacteriaceae;g__Maribacter | 0 | 0 | 0.0003273 | 0 | 0.00015403 | 0.00022886 | 0 | 0 | 0 | 0 | 0 | 0 | 0 | 0 | 0 |
| k__Bacteria;p__Bacteroidetes;c__Flavobacteriia;o__Flavobacteriales;f__Flavobacteriaceae;g__Mesonia | 0 | 0 | 0 | 0 | 0 | 0.00029927 | 0 | 0 | 0 | 0 | 0 | 0 | 0 | 0 | 0.00011465 |
| k__Bacteria;p__Bacteroidetes;c__Flavobacteriia;o__Flavobacteriales;f__Flavobacteriaceae;g__Olleya | 0.00029342 | 0 | 0.00133439 | 0 | 0.00202809 | 8.80E-05 | 0.00050751 | 0 | 0 | 0 | 0 | 0.00077177 | 0.0008088 | 0 | 0.0026861 |
| k__Bacteria;p__Bacteroidetes;c__Flavobacteriia;o__Flavobacteriales;f__Flavobacteriaceae;g__Persicivirga | 0 | 0 | 0 | 0 | 0.00020538 | 0 | 0 | 0 | 0 | 0 | 0 | 0 | 0 | 0 | 0.00044222 |
| k__Bacteria;p__Bacteroidetes;c__Flavobacteriia;o__Flavobacteriales;f__Flavobacteriaceae;g__Polaribacter | 0.00344106 | 0.00345743 | 0.00122109 | 0.00141189 | 0.00732935 | 0.00352088 | 0.00342881 | 0 | 0 | 0.00928203 | 0.02201676 | 0.04094249 | 0.01303703 | 0.00880967 | 0.0022275 |
| k__Bacteria;p__Bacteroidetes;c__Flavobacteriia;o__Flavobacteriales;f__Flavobacteriaceae;g__Pontirhabdus | 0 | 0 | 0 | 0 | 0 | 0 | 0 | 0 | 0 | 0 | 0 | 0 | 0 | 0 | 0 |
| k__Bacteria;p__Bacteroidetes;c__Flavobacteriia;o__Flavobacteriales;f__Flavobacteriaceae;g__Psychroserpens | 0 | 0 | 0 | 0.00070595 | 0.0001797 | 0 | 1.24E-05 | 0 | 0 | 0 | 0 | 0.0013506 | 0 | 0 | 1.64E-05 |
| k__Bacteria;p__Bacteroidetes;c__Flavobacteriia;o__Flavobacteriales;f__Flavobacteriaceae;g__Robiginitalea | 0 | 0 | 0 | 0 | 0 | 0 | 0 | 0 | 0 | 0 | 0 | 0 | 0 | 0 | 0 |
| k__Bacteria;p__Bacteroidetes;c__Flavobacteriia;o__Flavobacteriales;f__Flavobacteriaceae;g__Sediminicola | 0.01125679 | 0.00735708 | 0.00216524 | 0.00114037 | 0.01746977 | 0.03461024 | 0.04083628 | 0 | 0 | 0.05037683 | 0.0434245 | 0.03924459 | 0.00023108 | 0.00051458 | 0.02103022 |
| k__Bacteria;p__Bacteroidetes;c__Flavobacteriia;o__Flavobacteriales;f__Flavobacteriaceae;g__Tenacibaculum | 0.00202729 | 0.00219774 | 0.00085602 | 0.00130329 | 0.00295228 | 0.00649602 | 0.00646152 | 0 | 0 | 0.00983737 | 0.00609013 | 0.01023369 | 0.00231085 | 0.00203775 | 0.00332487 |
| k__Bacteria;p__Bacteroidetes;c__Flavobacteriia;o__Flavobacteriales;f__Flavobacteriaceae;g__Ulvibacter | 1.33E-05 | 0 | 0.00018883 | 0 | 6.42E-05 | 0 | 0 | 0 | 0 | 0 | 0 | 0.00023153 | 0.00063548 | 0 | 0.00029482 |
| k__Bacteria;p__Bacteroidetes;c__Flavobacteriia;o__Flavobacteriales;f__Flavobacteriaceae;g__Winogradskyella | 0 | 0 | 0 | 0 | 0 | 0.00021125 | 0.00016092 | 0 | 0 | 0 | 0 | 0 | 0 | 0.00137908 | 3.28E-05 |
| k__Bacteria;p__Bacteroidetes;c__Flavobacteriia;o__Flavobacteriales;f__NS9;g__ | 0.00013337 | 0.00029482 | 8.81E-05 | 0 | 0 | 0 | 0 | 0 | 0.00697807 | 0 | 0.00047983 | 0.0003473 | 0 | 0.00090567 | 0.0006879 |
| k__Bacteria;p__Bacteroidetes;c__Flavobacteriia;o__Flavobacteriales;f__[Weeksellaceae];g__ | 2.67E-05 | 0 | 6.29E-05 | 0 | 0 | 0 | 0 | 0.00361384 | 0 | 0 | 0 | 0 | 0 | 0 | 0 |
| k__Bacteria;p__Bacteroidetes;c__Flavobacteriia;o__Flavobacteriales;f__[Weeksellaceae];g__Chryseobacterium | 0 | 0 | 0.00010071 | 0 | 0 | 0 | 0 | 0.00980898 | 0 | 0 | 0 | 0 | 0 | 0.00045283 | 0 |
| k__Bacteria;p__Bacteroidetes;c__Flavobacteriia;o__Flavobacteriales;f__[Weeksellaceae];g__Cloacibacterium | 1.33E-05 | 0.00017421 | 0 | 0.06853109 | 2.57E-05 | 8.80E-05 | 2.48E-05 | 0 | 0 | 0.0011305 | 0.00012918 | 0 | 0 | 0 | 0 |
| k__Bacteria;p__Bacteroidetes;c__Flavobacteriia;o__Flavobacteriales;f__[Weeksellaceae];g__Weeksella | 0 | 0 | 0 | 0 | 0 | 0 | 0 | 0 | 0.00655084 | 0 | 0 | 0 | 0 | 0 | 0 |
| k__Bacteria;p__Bacteroidetes;c__Sphingobacteriia;o__Sphingobacteriales;f__;g__ | 0.00089361 | 0.00044223 | 0.00010071 | 0 | 0.00056478 | 0.00413703 | 0.00558265 | 0 | 0 | 0 | 0 | 0 | 0 | 0 | 0.00763246 |
| k__Bacteria;p__Bacteroidetes;c__Sphingobacteriia;o__Sphingobacteriales;f__NS11-12;g__ | 4.00E-05 | 0 | 7.55E-05 | 0 | 0.0001412 | 0 | 0 | 0 | 0 | 0.00589052 | 0.00175322 | 0.00059426 | 0.0034085 | 0 | 4.91E-05 |
| k__Bacteria;p__Bacteroidetes;c__Sphingobacteriia;o__Sphingobacteriales;f__Sphingobacteriaceae;g__Pedobacter | 0 | 0 | 0 | 0 | 6.42E-05 | 7.04E-05 | 0 | 0 | 0 | 0 | 0 | 0 | 0 | 0 | 4.91E-05 |
| k__Bacteria;p__Bacteroidetes;c__Sphingobacteriia;o__Sphingobacteriales;f__Sphingobacteriaceae;g__Sphingobacterium | 0 | 0.00026802 | 0.00020142 | 0 | 0.00012836 | 0 | 0 | 0 | 0 | 0 | 0 | 0 | 0 | 0 | 0 |
| k__Bacteria;p__Bacteroidetes;c__VC2_1_Bac22;o__;f__;g__ | 0 | 0 | 0 | 0.00124898 | 0 | 0 | 0 | 0 | 0 | 0 | 0 | 0 | 0 | 0 | 0 |
| k__Bacteria;p__Bacteroidetes;c__[Rhodothermi];o__[Rhodothermales];f__[Balneolaceae];g__Balneola | 0 | 0 | 7.55E-05 | 0 | 0 | 0 | 0 | 0 | 0 | 0 | 0 | 0 | 0 | 0 | 0 |
| k__Bacteria;p__Bacteroidetes;c__[Saprospirae];o__[Saprospirales];f__;g__ | 0.00020006 | 0.00022781 | 0.00031471 | 0 | 0 | 0 | 0 | 0 | 0 | 0 | 0 | 0 | 0 | 0 | 0 |
| k__Bacteria;p__Bacteroidetes;c__[Saprospirae];o__[Saprospirales];f__Chitinophagaceae;g__ | 0.00016005 | 0 | 0 | 0 | 0 | 1.76E-05 | 0 | 0 | 0 | 0 | 0.00014764 | 0 | 0 | 0 | 0 |
| k__Bacteria;p__Bacteroidetes;c__[Saprospirae];o__[Saprospirales];f__Chitinophagaceae;g__Chitinophaga | 0 | 0 | 0 | 0 | 0 | 0 | 0 | 0 | 0 | 0 | 0 | 0 | 0 | 0 | 0 |
| k__Bacteria;p__Bacteroidetes;c__[Saprospirae];o__[Saprospirales];f__Chitinophagaceae;g__Niabella | 0 | 0 | 0 | 0 | 0 | 0 | 0 | 0 | 0 | 0 | 0 | 0 | 0 | 0 | 0 |
| k__Bacteria;p__Bacteroidetes;c__[Saprospirae];o__[Saprospirales];f__Chitinophagaceae;g__Sediminibacterium | 0 | 0 | 0 | 0.00054304 | 0 | 0 | 0 | 0.00722767 | 0 | 0 | 0 | 0 | 0 | 0 | 0 |
| k__Bacteria;p__Bacteroidetes;c__[Saprospirae];o__[Saprospirales];f__Saprospiraceae;g__ | 2.67E-05 | 0.00013401 | 0.00037766 | 0 | 0.00012836 | 0 | 0 | 0 | 0 | 0 | 0 | 7.72E-05 | 0.00050068 | 0 | 8.19E-05 |
| k__Bacteria;p__Bacteroidetes;c__[Saprospirae];o__[Saprospirales];f__Saprospiraceae;g__Lewinella | 0 | 0 | 0 | 0 | 0 | 0 | 0 | 0 | 0 | 0 | 0 | 0 | 0 | 0 | 0 |
| k__Bacteria;p__Bacteroidetes;c__[Saprospirae];o__[Saprospirales];f__Saprospiraceae;g__Saprospira | 0 | 0 | 0.00010071 | 0 | 0 | 0 | 0 | 0 | 0 | 0 | 0 | 0 | 0 | 0 | 0 |
| k__Bacteria;p__Chlamydiae;c__Chlamydiia;o__Chlamydiales;f__;g__ | 0 | 0 | 0 | 0 | 0 | 0 | 0 | 0 | 0 | 0 | 0 | 0 | 0 | 0 | 0 |
| k__Bacteria;p__Chloroflexi;c__Anaerolineae;o__Caldilineales;f__Caldilineaceae;g__ | 0 | 0 | 0 | 0 | 0 | 0 | 4.95E-05 | 0 | 0 | 0 | 0 | 0 | 0 | 0 | 0 |
| k__Bacteria;p__Chloroflexi;c__Anaerolineae;o__GCA004;f__;g__ | 0 | 0 | 0 | 0 | 0 | 0 | 0 | 0 | 0 | 0 | 0 | 0 | 0 | 0 | 0 |
| k__Bacteria;p__Chloroflexi;c__Chloroflexi;o__[Roseiflexales];f__;g__ | 0 | 0 | 0 | 0 | 0 | 0 | 0 | 0 | 0 | 0 | 0 | 0 | 0 | 0 | 0 |
| k__Bacteria;p__Chloroflexi;c__Ellin6529;o__;f__;g__ | 0 | 0.00021441 | 0 | 0 | 0 | 0.00010563 | 0 | 0 | 0 | 0 | 0 | 0 | 0 | 0 | 0 |
| k__Bacteria;p__Chloroflexi;c__Gitt-GS-136;o__;f__;g__ | 0 | 0 | 0 | 0 | 0 | 0 | 1.24E-05 | 0 | 0 | 0 | 0 | 0 | 0 | 0 | 0 |
| k__Bacteria;p__Chloroflexi;c__S085;o__;f__;g__ | 0 | 0.00025462 | 0 | 0 | 0 | 0 | 0 | 0 | 0 | 0 | 0 | 0 | 0 | 0 | 0 |
| k__Bacteria;p__Chloroflexi;c__SAR202;o__;f__;g__ | 0 | 0 | 0 | 0 | 2.57E-05 | 0 | 0 | 0 | 0 | 0 | 0 | 0 | 0 | 0 | 3.28E-05 |
| k__Bacteria;p__Chloroflexi;c__TK10;o__AKYG885;f__Dolo_23;g__ | 0 | 0 | 0 | 0 | 0 | 0 | 0 | 0 | 0 | 0 | 0 | 0 | 0 | 0 | 0 |
| k__Bacteria;p__Cyanobacteria;c__4C0d-2;o__MLE1-12;f__;g__ | 0 | 0 | 0 | 0 | 0 | 0 | 0 | 0 | 0 | 0 | 0 | 0 | 0 | 0.0006175 | 0 |
| k__Bacteria;p__Cyanobacteria;c__4C0d-2;o__YS2;f__;g__ | 0 | 0 | 0 | 0 | 0 | 0 | 0 | 0 | 0 | 0 | 0 | 0 | 0 | 0 | 0 |
| k__Bacteria;p__Cyanobacteria;c__Chloroplast;o__;f__;g__ | 0.00018672 | 0 | 0 | 0 | 0.00020538 | 0 | 0 | 0 | 0 | 0 | 0 | 0 | 0 | 0 | 0 |
| k__Bacteria;p__Cyanobacteria;c__Chloroplast;o__Cercozoa;f__;g__ | 0 | 0 | 0 | 0 | 0 | 0 | 0 | 0 | 0 | 0 | 0 | 0 | 0 | 0 | 0 |
| k__Bacteria;p__Cyanobacteria;c__Chloroplast;o__Chlorophyta;f__;g__ | 0.00097363 | 0.00054944 | 0.00035248 | 0 | 6.42E-05 | 0.00461235 | 0.00334216 | 0 | 0 | 0 | 0 | 0 | 0 | 0 | 0.00461879 |
| k__Bacteria;p__Cyanobacteria;c__Chloroplast;o__Chlorophyta;f__Mamiellaceae;g__ | 0.01048321 | 0.00950122 | 0.00417941 | 0 | 0.00284959 | 0.01781565 | 0.01387617 | 0 | 0.00462831 | 0 | 7.38E-05 | 0 | 0 | 0.00010292 | 0.00530669 |
| k__Bacteria;p__Cyanobacteria;c__Chloroplast;o__Chlorophyta;f__Mamiellaceae;g__Micromonas | 0 | 0 | 0 | 0 | 0 | 0 | 1.24E-05 | 0 | 0 | 0 | 0 | 0 | 0 | 6.17E-05 | 3.28E-05 |
| k__Bacteria;p__Cyanobacteria;c__Chloroplast;o__Chlorophyta;f__Trebouxiophyceae;g__ | 0 | 0 | 0 | 0 | 0 | 0 | 0 | 0 | 0 | 0 | 0 | 0 | 0 | 0 | 0 |
| k__Bacteria;p__Cyanobacteria;c__Chloroplast;o__Cryptophyta;f__;g__ | 0 | 0.00024122 | 0 | 0 | 0 | 0.00258785 | 0 | 0 | 0 | 0 | 0 | 0 | 0 | 0 | 1.64E-05 |
| k__Bacteria;p__Cyanobacteria;c__Chloroplast;o__Haptophyceae;f__;g__ | 0 | 0.00057624 | 0.00035248 | 0 | 0 | 0.00781635 | 0 | 0 | 0 | 0 | 0 | 0 | 0 | 0 | 4.91E-05 |
| k__Bacteria;p__Cyanobacteria;c__Chloroplast;o__Rhodophyta;f__;g__ | 0 | 0 | 0 | 0 | 0 | 0 | 0 | 0 | 0 | 0 | 0 | 0 | 0 | 0 | 0 |
| k__Bacteria;p__Cyanobacteria;c__Chloroplast;o__Stramenopiles;f__;g__ | 0.00016005 | 0 | 0 | 0 | 0 | 0.0016196 | 0.00054465 | 0 | 0 | 0 | 0.00105193 | 0.00046306 | 0.00115542 | 0 | 0.00109737 |
| k__Bacteria;p__Cyanobacteria;c__Chloroplast;o__Streptophyta;f__;g__ | 0.00157382 | 0.00010721 | 0.00017624 | 0 | 7.70E-05 | 1.76E-05 | 0 | 0.00051626 | 0.02570493 | 0.00616819 | 0.00051674 | 0 | 0.000674 | 0.00057633 | 3.28E-05 |
| k__Bacteria;p__Cyanobacteria;c__Nostocophycideae;o__Nostocales;f__Nostocaceae;g__ | 0 | 0 | 0 | 0 | 0 | 0 | 0 | 0 | 0.0118912 | 0 | 0 | 0 | 0 | 0 | 0 |
| k__Bacteria;p__Cyanobacteria;c__Nostocophycideae;o__Stigonematales;f__Rivulariaceae;g__Calothrix | 0 | 5.36E-05 | 0 | 0 | 0 | 0 | 9.90E-05 | 0.04801239 | 0.00306181 | 0 | 0 | 0 | 0 | 0.00041167 | 8.19E-05 |
| k__Bacteria;p__Cyanobacteria;c__Oscillatoriophycideae;o__Chroococcales;f__Cyanobacteriaceae;g__ | 0 | 0 | 0 | 0 | 0 | 0 | 0 | 0 | 0 | 0 | 0 | 0 | 0 | 0 | 0 |
| k__Bacteria;p__Cyanobacteria;c__Oscillatoriophycideae;o__Chroococcales;f__Microcystaceae;g__Microcystis | 0 | 0 | 0 | 0 | 0 | 0 | 0 | 0 | 0 | 0 | 0 | 0 | 0 | 0 | 1.64E-05 |
| k__Bacteria;p__Cyanobacteria;c__Oscillatoriophycideae;o__Chroococcales;f__Xenococcaceae;g__ | 0.00026675 | 0.00030822 | 0.00037766 | 0.00407277 | 7.70E-05 | 0.00019365 | 0.00023519 | 0 | 0 | 0.000833 | 0.00068283 | 0 | 0.0009436 | 0 | 0.00013103 |
| k__Bacteria;p__Cyanobacteria;c__Synechococcophycideae;o__Pseudanabaenales;f__Pseudanabaenaceae;g__ | 0 | 0 | 0 | 0 | 0 | 0 | 0 | 0 | 0 | 0 | 0 | 0 | 0 | 0 | 0 |
| k__Bacteria;p__Cyanobacteria;c__Synechococcophycideae;o__Pseudanabaenales;f__Pseudanabaenaceae;g__Pseudanabaena | 0 | 0 | 0 | 0 | 0 | 0.00019365 | 0 | 0 | 0 | 0 | 0 | 0 | 0 | 0 | 0 |
| k__Bacteria;p__Cyanobacteria;c__Synechococcophycideae;o__Synechococcales;f__Synechococcaceae;g__Prochlorococcus | 0 | 0.00029482 | 0.00081826 | 0 | 1.28E-05 | 0.00019365 | 0 | 0 | 0 | 0 | 0.00012918 | 0 | 0.00042366 | 0 | 0 |
| k__Bacteria;p__Cyanobacteria;c__Synechococcophycideae;o__Synechococcales;f__Synechococcaceae;g__Synechococcus | 0.00058685 | 0 | 0.00119592 | 0 | 0.00037224 | 0.00327442 | 0.00012378 | 0 | 0.00526915 | 0 | 0.00060901 | 0 | 0 | 0 | 0.00039309 |
| k__Bacteria;p__FBP;c__;o__;f__;g__ | 0 | 0 | 0 | 0 | 0 | 0 | 0 | 0 | 0 | 0 | 0 | 0 | 0 | 0 | 0 |
| k__Bacteria;p__Fibrobacteres;c__Fibrobacteria;o__Fibrobacterales;f__;g__ | 0 | 0 | 0 | 0 | 0 | 0 | 0 | 0 | 0 | 0 | 0.00031373 | 0 | 0 | 0 | 0 |
| k__Bacteria;p__Firmicutes;c__Bacilli;o__Bacillales;f__;g__ | 0 | 0 | 0 | 0 | 0 | 0 | 0 | 0 | 0 | 0 | 3.69E-05 | 0 | 0 | 0 | 0 |
| k__Bacteria;p__Firmicutes;c__Bacilli;o__Bacillales;f__Bacillaceae;g__Anoxybacillus | 0.00022674 | 0.00010721 | 0.00017624 | 0 | 0 | 0 | 0 | 0 | 0 | 0 | 0 | 0 | 0 | 0 | 0 |
| k__Bacteria;p__Firmicutes;c__Bacilli;o__Bacillales;f__Bacillaceae;g__Bacillus | 0 | 0 | 0 | 0.00217214 | 0 | 7.04E-05 | 0 | 0 | 0.00541156 | 0 | 0 | 0 | 0 | 0 | 0 |
| k__Bacteria;p__Firmicutes;c__Bacilli;o__Bacillales;f__Bacillaceae;g__Geobacillus | 0 | 0 | 0 | 0.00385555 | 0 | 0 | 0 | 0 | 0 | 0 | 0 | 0 | 0 | 0 | 0 |
| k__Bacteria;p__Firmicutes;c__Bacilli;o__Bacillales;f__Bacillaceae;g__Marinococcus | 0 | 0 | 0 | 0 | 0 | 0 | 0 | 0 | 0 | 0 | 0 | 0 | 0 | 0 | 3.28E-05 |
| k__Bacteria;p__Firmicutes;c__Bacilli;o__Bacillales;f__Listeriaceae;g__ | 0 | 0 | 0 | 0 | 0 | 0 | 0 | 0 | 0 | 0 | 0.00023991 | 0 | 0 | 0 | 0 |
| k__Bacteria;p__Firmicutes;c__Bacilli;o__Bacillales;f__Listeriaceae;g__Brochothrix | 0 | 0 | 0 | 0 | 0 | 0 | 0 | 0 | 0 | 0 | 1.85E-05 | 0 | 0 | 0 | 0 |

| Taxon | 1231 | 1233 | 1234 | 1235 | 1236 | 1243 | 1244 | 6473 | 6474 | 1188 | 1189 | 1190 | 1226 | 1227 | 2027 |
|---|---|---|---|---|---|---|---|---|---|---|---|---|---|---|---|
| Copepod genus | Calanus | Calanus | Calanus | Calanus | Calanus | Centropages | Centropages | Centropages | Centropages | Centropages | Centropages | Centropages | Centropages | Centropages | Centropages |
| Type of sample | FullGut | FullGut | FullGut | FullGut | FullGut | FullGut | FullGut | FullGut | FullGut | Starved | Starved | Starved | Starved | Starved | Starved |
| k__Bacteria;p__Firmicutes;c__Bacilli;o__Bacillales;f__Paenibacillaceae;g__Paenibacillus | 0 | 0 | 0 | 0 | 0 | 0 | 0 | 0 | 0 | 0 | 0 | 0 | 0 | 0.00065867 | 0 |
| k__Bacteria;p__Firmicutes;c__Bacilli;o__Bacillales;f__Planococcaceae;g__ | 0.0103365 | 0.02661414 | 0.02676335 | 0.00418137 | 0.00025672 | 0.03659954 | 0 | 0 | 0 | 0.00146767 | 0.00027682 | 0 | 0.09345452 | 0.11314657 | 1.64E-05 |
| k__Bacteria;p__Firmicutes;c__Bacilli;o__Bacillales;f__Planococcaceae;g__Bacillus | 0.00018672 | 0 | 0 | 0 | 0 | 0 | 0 | 0 | 0 | 0 | 0 | 0 | 0 | 0 | 0 |
| k__Bacteria;p__Firmicutes;c__Bacilli;o__Bacillales;f__Planococcaceae;g__Planococcus | 0 | 0 | 0 | 0 | 0 | 0 | 0 | 0 | 0 | 0 | 0 | 0 | 0 | 0 | 0 |
| k__Bacteria;p__Firmicutes;c__Bacilli;o__Bacillales;f__Planococcaceae;g__Planomicrobium | 0 | 0 | 0 | 0 | 0 | 0 | 0 | 0 | 0 | 0 | 0 | 0 | 0 | 0.00113208 | 0 |
| k__Bacteria;p__Firmicutes;c__Bacilli;o__Bacillales;f__Staphylococcaceae;g__Jeotgalicoccus | 0 | 0 | 0 | 0 | 0 | 0 | 0 | 0 | 0 | 0 | 0 | 0 | 0.00061623 | 0 | 0 |
| k__Bacteria;p__Firmicutes;c__Bacilli;o__Bacillales;f__Staphylococcaceae;g__Staphylococcus | 0.00089361 | 0.00054944 | 0.0005539 | 0.00314961 | 0.00029523 | 0.00042251 | 0.00012378 | 0 | 0.03090288 | 0 | 0.00025837 | 0 | 0.00437135 | 0.00129675 | 0.00016379 |
| k__Bacteria;p__Firmicutes;c__Bacilli;o__Bacillales;f__Thermoactinomycetaceae;g__ | 0 | 0 | 0 | 0.00059734 | 0 | 0 | 0 | 0 | 0 | 0 | 0 | 0 | 0 | 0 | 0 |
| k__Bacteria;p__Firmicutes;c__Bacilli;o__Bacillales;f__Thermoactinomycetaceae;g__Planifilum | 0 | 0 | 0 | 0 | 0 | 0 | 0 | 0 | 0 | 0 | 0 | 0 | 0 | 0 | 0 |
| k__Bacteria;p__Firmicutes;c__Bacilli;o__Bacillales;f__[Exiguobacteraceae];g__Exiguobacterium | 0 | 0 | 0 | 0 | 0 | 0 | 0 | 0 | 0 | 0 | 0 | 0 | 0 | 0 | 0 |
| k__Bacteria;p__Firmicutes;c__Bacilli;o__Bacillales;f__[Thermicanaceae];g__Thermicanus | 0 | 0 | 0 | 0 | 0 | 0 | 0 | 0 | 0 | 0 | 0 | 0 | 0 | 0 | 0 |
| k__Bacteria;p__Firmicutes;c__Bacilli;o__Gemellales;f__Gemellaceae;g__ | 0 | 0 | 0.00015106 | 0 | 0 | 0 | 0 | 0 | 0 | 0 | 0.00042446 | 0 | 0 | 0 | 0 |
| k__Bacteria;p__Firmicutes;c__Bacilli;o__Gemellales;f__Gemellaceae;g__Gemella | 0 | 0 | 0 | 0 | 0 | 0 | 0 | 0 | 0 | 0 | 0 | 0 | 0 | 0 | 0 |
| k__Bacteria;p__Firmicutes;c__Bacilli;o__Lactobacillales;f__Aerococcaceae;g__ | 0.0002134 | 0 | 0 | 0 | 3.85E-05 | 0 | 0 | 0 | 0 | 0 | 0 | 0 | 0 | 0 | 0 |
| k__Bacteria;p__Firmicutes;c__Bacilli;o__Lactobacillales;f__Aerococcaceae;g__Aerococcus | 0 | 0 | 0 | 0 | 0 | 0 | 0 | 0 | 0 | 0 | 0 | 0 | 0 | 0 | 0 |
| k__Bacteria;p__Firmicutes;c__Bacilli;o__Lactobacillales;f__Aerococcaceae;g__Alloiococcus | 0 | 0 | 0 | 0 | 0 | 0.00105626 | 0 | 0 | 0 | 0 | 0 | 0 | 0.00046217 | 0.00051458 | 3.28E-05 |
| k__Bacteria;p__Firmicutes;c__Bacilli;o__Lactobacillales;f__Aerococcaceae;g__Facklamia | 0 | 0 | 0 | 0.00249796 | 0 | 0 | 0 | 0 | 0 | 0 | 0 | 0 | 0 | 0 | 0 |
| k__Bacteria;p__Firmicutes;c__Bacilli;o__Lactobacillales;f__Aerococcaceae;g__Marinilactibacillus | 0 | 0.00033502 | 0 | 0 | 0 | 0 | 0 | 0 | 0 | 0 | 0 | 0 | 0 | 0 | 0 |
| k__Bacteria;p__Firmicutes;c__Bacilli;o__Lactobacillales;f__Carnobacteriaceae;g__ | 0 | 0 | 0 | 0 | 0 | 0 | 0 | 0 | 0 | 0 | 0 | 0 | 0 | 0 | 0 |
| k__Bacteria;p__Firmicutes;c__Bacilli;o__Lactobacillales;f__Carnobacteriaceae;g__Granulicatella | 0 | 0 | 0 | 0 | 0 | 0 | 0.00012378 | 0 | 0 | 0 | 0 | 9.26E-05 | 0.0008088 | 0 | 0 |
| k__Bacteria;p__Firmicutes;c__Bacilli;o__Lactobacillales;f__Carnobacteriaceae;g__Trichococcus | 0 | 0 | 0 | 0 | 0 | 0 | 0 | 0 | 0 | 0 | 0 | 0 | 0 | 0 | 0 |
| k__Bacteria;p__Firmicutes;c__Bacilli;o__Lactobacillales;f__Enterococcaceae;g__Enterococcus | 0 | 0 | 0 | 0.00695086 | 0 | 0 | 0 | 0 | 0 | 0.00079334 | 0 | 0 | 0.00140577 | 0 | 0 |
| k__Bacteria;p__Firmicutes;c__Bacilli;o__Lactobacillales;f__Lactobacillaceae;g__Lactobacillus | 0 | 0 | 0 | 0 | 0 | 0.00049292 | 8.66E-05 | 0 | 0.00797494 | 0 | 0.00031373 | 0 | 0 | 0 | 0 |
| k__Bacteria;p__Firmicutes;c__Bacilli;o__Lactobacillales;f__Leuconostocaceae;g__ | 0 | 0 | 0 | 0.00385555 | 0 | 0 | 0 | 0 | 0 | 0 | 0 | 0 | 0 | 0 | 0 |
| k__Bacteria;p__Firmicutes;c__Bacilli;o__Lactobacillales;f__Leuconostocaceae;g__Leuconostoc | 0 | 0 | 0.00017624 | 0 | 0 | 0 | 0 | 0 | 0 | 0 | 0 | 0 | 0 | 0 | 0 |
| k__Bacteria;p__Firmicutes;c__Bacilli;o__Lactobacillales;f__Leuconostocaceae;g__Weissella | 0 | 0 | 0 | 0 | 0 | 0 | 0 | 0 | 0.01317289 | 0 | 0 | 0 | 0 | 0 | 0 |
| k__Bacteria;p__Firmicutes;c__Bacilli;o__Lactobacillales;f__Streptococcaceae;g__Lactococcus | 0 | 6.70E-05 | 0.00012589 | 0 | 0 | 0 | 6.19E-05 | 0 | 0 | 0 | 0 | 0 | 0 | 0 | 0 |
| k__Bacteria;p__Firmicutes;c__Bacilli;o__Lactobacillales;f__Streptococcaceae;g__Streptococcus | 0.00060018 | 0 | 5.04E-05 | 0.00439859 | 7.70E-05 | 0.00112668 | 3.71E-05 | 0.03975219 | 0.03154372 | 0.00212217 | 0.00079356 | 0 | 0.00970556 | 0.00193483 | 4.91E-05 |
| k__Bacteria;p__Firmicutes;c__Clostridia;o__Clostridiales;f__;g__ | 0 | 0 | 0 | 0 | 0 | 0.00014084 | 0 | 0 | 0 | 0 | 0 | 0 | 0 | 0 | 0 |
| k__Bacteria;p__Firmicutes;c__Clostridia;o__Clostridiales;f__Clostridiaceae;g__ | 0 | 0 | 0 | 0 | 0 | 0 | 0 | 0 | 0 | 0 | 0 | 0 | 0 | 0 | 0 |
| k__Bacteria;p__Firmicutes;c__Clostridia;o__Clostridiales;f__Clostridiaceae;g__Clostridium | 0 | 0 | 0 | 0 | 0 | 0.00026407 | 0 | 0 | 0 | 0 | 0 | 0 | 0 | 0 | 3.28E-05 |
| k__Bacteria;p__Firmicutes;c__Clostridia;o__Clostridiales;f__Lachnospiraceae;g__ | 0 | 0 | 0 | 0 | 0 | 0 | 0 | 0 | 0 | 0 | 0 | 0 | 0 | 0 | 0 |
| k__Bacteria;p__Firmicutes;c__Clostridia;o__Clostridiales;f__Lachnospiraceae;g__Blautia | 0 | 0 | 0 | 0 | 0 | 7.04E-05 | 0 | 0 | 0 | 0 | 0 | 0 | 0 | 0.00094683 | 0 |
| k__Bacteria;p__Firmicutes;c__Clostridia;o__Clostridiales;f__Lachnospiraceae;g__Dorea | 0 | 0 | 0 | 0 | 0 | 0 | 0 | 0 | 0 | 0 | 0 | 0 | 0 | 0 | 0 |
| k__Bacteria;p__Firmicutes;c__Clostridia;o__Clostridiales;f__Lachnospiraceae;g__Moryella | 0 | 0 | 0 | 0 | 0 | 0 | 0 | 0 | 0 | 0 | 0 | 0 | 0 | 0 | 0 |
| k__Bacteria;p__Firmicutes;c__Clostridia;o__Clostridiales;f__Lachnospiraceae;g__Oribacterium | 0 | 0 | 0 | 0 | 0 | 0 | 0 | 0 | 0 | 0 | 9.23E-05 | 0 | 0 | 0 | 0 |
| k__Bacteria;p__Firmicutes;c__Clostridia;o__Clostridiales;f__Lachnospiraceae;g__[Ruminococcus] | 0 | 0 | 0 | 0 | 0 | 0 | 0 | 0 | 0 | 0 | 0 | 0 | 0 | 0 | 0 |
| k__Bacteria;p__Firmicutes;c__Clostridia;o__Clostridiales;f__Peptostreptococcaceae;g__ | 0 | 0 | 0 | 0 | 0 | 0 | 0 | 0 | 0 | 0 | 0 | 0 | 0 | 0 | 0 |
| k__Bacteria;p__Firmicutes;c__Clostridia;o__Clostridiales;f__Ruminococcaceae;g__ | 0 | 0 | 0 | 0.00065164 | 0 | 0 | 0 | 0 | 0 | 0 | 0 | 0 | 0 | 0 | 0 |
| k__Bacteria;p__Firmicutes;c__Clostridia;o__Clostridiales;f__Ruminococcaceae;g__Clostridium | 0 | 0 | 0 | 0 | 0 | 0 | 0 | 0 | 0 | 0 | 0 | 0 | 0 | 0 | 0 |
| k__Bacteria;p__Firmicutes;c__Clostridia;o__Clostridiales;f__Ruminococcaceae;g__Faecalibacterium | 0 | 0 | 0 | 0 | 0 | 0 | 0 | 0 | 0 | 0 | 0 | 0 | 0 | 0 | 0 |
| k__Bacteria;p__Firmicutes;c__Clostridia;o__Clostridiales;f__Ruminococcaceae;g__Oscillospira | 0 | 0 | 0 | 0 | 0 | 0 | 0 | 0 | 0 | 0 | 0 | 0 | 0 | 0 | 0 |
| k__Bacteria;p__Firmicutes;c__Clostridia;o__Clostridiales;f__Veillonellaceae;g__ | 0 | 0 | 0 | 0 | 0 | 0 | 0 | 0 | 0 | 0 | 0 | 0 | 0 | 0 | 0 |
| k__Bacteria;p__Firmicutes;c__Clostridia;o__Clostridiales;f__Veillonellaceae;g__Anaeromusa | 0 | 0 | 0 | 0 | 0 | 0 | 0 | 0 | 0 | 0 | 0 | 0 | 0 | 0 | 0 |
| k__Bacteria;p__Firmicutes;c__Clostridia;o__Clostridiales;f__Veillonellaceae;g__Dialister | 0 | 0 | 0 | 0 | 0 | 0 | 0 | 0 | 0 | 0 | 0 | 0 | 0 | 0 | 0 |
| k__Bacteria;p__Firmicutes;c__Clostridia;o__Clostridiales;f__Veillonellaceae;g__Pelosinus | 0 | 0 | 0 | 0 | 0 | 0 | 0 | 0 | 0 | 0 | 0 | 0 | 0 | 0 | 0 |
| k__Bacteria;p__Firmicutes;c__Clostridia;o__Clostridiales;f__Veillonellaceae;g__Phascolarctobacterium | 0 | 0 | 0 | 0 | 0 | 0 | 0 | 0 | 0 | 0 | 0 | 0 | 0 | 0 | 0 |
| k__Bacteria;p__Firmicutes;c__Clostridia;o__Clostridiales;f__Veillonellaceae;g__Selenomonas | 0 | 0 | 0 | 0 | 0 | 0 | 0 | 0 | 0 | 0 | 0 | 0 | 0 | 0 | 0 |
| k__Bacteria;p__Firmicutes;c__Clostridia;o__Clostridiales;f__Veillonellaceae;g__Veillonella | 0 | 0 | 0 | 0.00097746 | 0 | 0.00012323 | 0.00040849 | 0.10789881 | 0.01295927 | 0 | 0.00016609 | 0 | 0 | 0 | 0 |
| k__Bacteria;p__Firmicutes;c__Clostridia;o__Clostridiales;f__[Acidaminobacteraceae];g__Fusibacter | 0 | 0 | 0 | 0 | 0 | 0 | 0 | 0 | 0 | 0 | 0 | 0 | 0 | 0 | 0 |
| k__Bacteria;p__Firmicutes;c__Clostridia;o__Clostridiales;f__[Acidaminobacteraceae];g__WH1-8 | 0 | 0 | 0 | 0 | 0 | 0 | 0 | 0 | 0 | 0 | 0 | 0 | 0 | 0 | 0 |
| k__Bacteria;p__Firmicutes;c__Clostridia;o__Clostridiales;f__[Mogibacteriaceae];g__ | 0 | 0 | 0 | 0 | 0 | 0 | 0 | 0 | 0 | 0 | 0 | 0 | 0 | 0 | 0 |
| k__Bacteria;p__Firmicutes;c__Clostridia;o__Clostridiales;f__[Mogibacteriaceae];g__Mogibacterium | 0 | 0 | 0 | 0 | 0 | 0 | 0 | 0 | 0 | 0 | 0 | 0 | 0 | 0 | 1.64E-05 |
| k__Bacteria;p__Firmicutes;c__Clostridia;o__Clostridiales;f__[Tissierellaceae];g__Anaerococcus | 0.00038679 | 0 | 0 | 0.00293239 | 0 | 0 | 6.19E-05 | 0 | 0 | 0.00061484 | 0 | 0 | 0 | 0 | 0 |
| k__Bacteria;p__Firmicutes;c__Clostridia;o__Clostridiales;f__[Tissierellaceae];g__Finegoldia | 0 | 0 | 0 | 0 | 0 | 0 | 0 | 0 | 0 | 0 | 0 | 0 | 0 | 0 | 0 |
| k__Bacteria;p__Firmicutes;c__Clostridia;o__Clostridiales;f__[Tissierellaceae];g__Gallicola | 0 | 0 | 0 | 0 | 0 | 0 | 0 | 0 | 0 | 0 | 0 | 0 | 0.00177165 | 0 | 0 |
| k__Bacteria;p__Firmicutes;c__Clostridia;o__Clostridiales;f__[Tissierellaceae];g__Parvimonas | 0 | 0 | 0 | 0 | 0 | 0 | 0 | 0 | 0 | 0 | 0 | 0 | 0 | 0 | 0 |
| k__Bacteria;p__Firmicutes;c__Clostridia;o__Clostridiales;f__[Tissierellaceae];g__Peptoniphilus | 0 | 0 | 0 | 0 | 0 | 0 | 0 | 0 | 0 | 0 | 0 | 0 | 0 | 0 | 0 |
| k__Bacteria;p__Firmicutes;c__Clostridia;o__Clostridiales;f__[Tissierellaceae];g__WAL_1855D | 0.00100031 | 0 | 0 | 0 | 0 | 0 | 0 | 0 | 0 | 0 | 0 | 0 | 0 | 0 | 0 |
| k__Bacteria;p__Firmicutes;c__Clostridia;o__Thermoanaerobacterales;f__Caldicellulosiruptoraceae;g__Caldicellulosiruptor | 0 | 0 | 0 | 0 | 0 | 0 | 0 | 0 | 0 | 0 | 0 | 0 | 0 | 0 | 0 |
| k__Bacteria;p__Firmicutes;c__Erysipelotrichi;o__Erysipelotrichales;f__Erysipelotrichaceae;g__ | 0 | 0 | 0 | 0 | 0 | 0 | 0 | 0 | 0 | 0 | 0 | 0 | 0 | 0 | 0 |
| k__Bacteria;p__Firmicutes;c__Erysipelotrichi;o__Erysipelotrichales;f__Erysipelotrichaceae;g__Coprobacillus | 0 | 0 | 0 | 0 | 0 | 0 | 0 | 0 | 0.00121048 | 0 | 0 | 0 | 0 | 0 | 0 |
| k__Bacteria;p__Firmicutes;c__Erysipelotrichi;o__Erysipelotrichales;f__Erysipelotrichaceae;g__RFN20 | 0 | 0 | 0 | 0 | 0 | 0 | 0 | 0 | 0 | 0 | 0 | 0 | 0 | 0 | 0 |
| k__Bacteria;p__Firmicutes;c__Erysipelotrichi;o__Erysipelotrichales;f__Erysipelotrichaceae;g__[Eubacterium] | 0 | 0 | 0 | 0 | 0 | 0 | 0 | 0 | 0 | 0 | 0 | 0 | 0 | 0 | 0 |
| k__Bacteria;p__Fusobacteria;c__Fusobacteriia;o__Fusobacteriales;f__;g__ | 0 | 0 | 0 | 0 | 0 | 0 | 0 | 0 | 0 | 0 | 0 | 0 | 0 | 0 | 0 |
| k__Bacteria;p__Fusobacteria;c__Fusobacteriia;o__Fusobacteriales;f__Fusobacteriaceae;g__Cetobacterium | 0 | 0 | 0 | 0 | 0 | 0 | 0 | 0 | 0 | 0 | 0 | 0 | 0 | 0 | 0 |
| k__Bacteria;p__Fusobacteria;c__Fusobacteriia;o__Fusobacteriales;f__Fusobacteriaceae;g__Fusobacterium | 2.67E-05 | 0 | 0 | 0.00070595 | 0 | 0 | 0 | 0 | 0 | 0 | 0.00022146 | 0 | 0.00055845 | 0.00055575 | 0 |
| k__Bacteria;p__Fusobacteria;c__Fusobacteriia;o__Fusobacteriales;f__Fusobacteriaceae;g__Propionigenium | 0 | 0 | 0 | 0 | 0 | 0 | 0 | 0 | 0 | 0 | 0 | 0 | 0 | 0 | 0 |
| k__Bacteria;p__Fusobacteria;c__Fusobacteriia;o__Fusobacteriales;f__Fusobacteriaceae;g__Psychrilyobacter | 0 | 0 | 0 | 0 | 0 | 0 | 0 | 0 | 0 | 0 | 0 | 0 | 0 | 0 | 0 |
| k__Bacteria;p__Fusobacteria;c__Fusobacteriia;o__Fusobacteriales;f__Fusobacteriaceae;g__u114 | 0 | 0 | 0 | 0 | 0 | 0 | 0 | 0 | 0 | 0 | 0 | 0 | 0 | 0 | 0 |
| k__Bacteria;p__Fusobacteria;c__Fusobacteriia;o__Fusobacteriales;f__Leptotrichiaceae;g__Leptotrichia | 0 | 0 | 0 | 0.00190062 | 0 | 0 | 0 | 0 | 0 | 0 | 0 | 0 | 0 | 0 | 0 |
| k__Bacteria;p__GN02;c__;o__;f__;g__ | 0 | 0 | 0 | 0 | 0 | 0 | 0 | 0 | 0 | 0 | 0 | 0 | 0 | 0 | 0 |
| k__Bacteria;p__GN02;c__BD1-5;o__;f__;g__ | 0.00062686 | 0 | 0.00203935 | 0 | 0.00012836 | 0 | 0.00076746 | 0 | 0 | 0 | 0 | 0 | 0 | 0 | 0.00586357 |
| k__Bacteria;p__GN02;c__IIB17;o__;f__;g__ | 0 | 0 | 0 | 0 | 0 | 0 | 0 | 0 | 0 | 0 | 0 | 0 | 0 | 0 | 0 |

| Taxon | 1231 | 1233 | 1234 | 1235 | 1236 | 1243 | 1244 | 6473 | 6474 | 1188 | 1189 | 1190 | 1226 | 1227 | 2027 |
|---|---|---|---|---|---|---|---|---|---|---|---|---|---|---|---|
| Copepod genus | Calanus | Calanus | Calanus | Calanus | Calanus | Centropages | Centropages | Centropages | Centropages | Centropages | Centropages | Centropages | Centropages | Centropages | Centropages |
| Type of sample | FullGut | FullGut | FullGut | FullGut | FullGut | FullGut | FullGut | FullGut | FullGut | Starved | Starved | Starved | Starved | Starved | Starved |
| k__Bacteria;p__Gemmatimonadetes;c__Gemmatimonadetes;o__;f__;g__ | 0 | 0 | 0 | 0 | 0 | 0 | 0 | 0 | 0 | 0 | 0 | 0 | 0 | 0 | 0 |
| k__Bacteria;p__Gemmatimonadetes;c__Gemmatimonadetes;o__Gemmatimonadales;f__;g__ | 0 | 0 | 0 | 0.00043443 | 0 | 3.52E-05 | 0 | 0 | 0 | 0 | 0 | 7.72E-06 | 0 | 0 | 0 |
| k__Bacteria;p__Lentisphaerae;c__[Lentisphaeria];o__Lentisphaerales;f__;g__ | 0 | 0 | 0 | 0 | 0 | 0 | 0 | 0 | 0 | 0 | 0 | 0 | 0 | 0 | 0 |
| k__Bacteria;p__Lentisphaerae;c__[Lentisphaeria];o__Lentisphaerales;f__Lentisphaeraceae;g__ | 0 | 0 | 0 | 0 | 0 | 0 | 0 | 0 | 0 | 0 | 0 | 0 | 0 | 0 | 0 |
| k__Bacteria;p__Lentisphaerae;c__[Lentisphaeria];o__Lentisphaerales;f__Lentisphaeraceae;g__Lentisphaera | 0 | 0 | 0 | 0 | 0 | 0 | 0 | 0 | 0 | 0 | 0 | 0 | 0 | 0 | 0 |
| k__Bacteria;p__Lentisphaerae;c__[Lentisphaeria];o__Victivallales;f__Victivallaceae;g__ | 0 | 0 | 0 | 0 | 0 | 0 | 0 | 0 | 0 | 0 | 0 | 0 | 0 | 0 | 0 |
| k__Bacteria;p__NKB19;c__SHAB590;o__;f__;g__ | 0 | 0 | 0 | 0 | 0 | 0 | 0 | 0 | 0 | 0 | 0 | 0 | 0 | 0 | 0 |
| k__Bacteria;p__Nitrospirae;c__Nitrospira;o__Nitrospirales;f__Nitrospiraceae;g__ | 0 | 0 | 0 | 0 | 0 | 0 | 0 | 0 | 0 | 0 | 0 | 0 | 0 | 0 | 0 |
| k__Bacteria;p__OD1;c__;o__;f__;g__ | 0 | 0 | 0 | 0 | 0 | 0 | 0 | 0 | 0 | 0 | 0 | 0 | 0 | 0.00032933 | 0 |
| k__Bacteria;p__OD1;c__ABY1;o__;f__;g__ | 0 | 0 | 0 | 0 | 0 | 0 | 0 | 0 | 0 | 0 | 0 | 0 | 0 | 0 | 0 |
| k__Bacteria;p__OD1;c__SM2F11;o__;f__;g__ | 0.0001067 | 0 | 0 | 0 | 0 | 0 | 3.71E-05 | 0 | 0 | 0 | 0 | 0 | 0.00011554 | 0.0001235 | 0 |
| k__Bacteria;p__OD1;c__ZB2;o__;f__;g__ | 2.67E-05 | 0 | 0 | 5.43E-05 | 0 | 0 | 0 | 0 | 0 | 0 | 0 | 0 | 0 | 0 | 1.64E-05 |
| k__Bacteria;p__OP8;c__OP8_1;o__HMMVPog-54;f__;g__ | 0 | 0 | 0 | 0 | 0 | 0 | 0 | 0 | 0 | 0 | 0 | 0 | 0 | 0 | 0 |
| k__Bacteria;p__Planctomycetes;c__OM190;o__CL500-15;f__;g__ | 1.33E-05 | 0 | 0 | 0 | 0 | 0 | 0 | 0 | 0 | 0 | 0 | 0 | 0 | 0 | 0 |
| k__Bacteria;p__Planctomycetes;c__OM190;o__agg27;f__;g__ | 0 | 0 | 0 | 0 | 0 | 0.00012323 | 0 | 0 | 0 | 0 | 0 | 0 | 0 | 0 | 0 |
| k__Bacteria;p__Planctomycetes;c__Phycisphaerae;o__Phycisphaerales;f__;g__ | 0.00016005 | 0 | 0 | 0 | 0 | 0 | 0 | 0 | 0 | 0 | 0 | 0 | 0 | 0.00133792 | 1.64E-05 |
| k__Bacteria;p__Planctomycetes;c__Phycisphaerae;o__Phycisphaerales;f__Phycisphaeraceae;g__ | 0 | 0 | 0 | 0 | 0 | 0 | 0 | 0 | 0 | 0 | 0 | 0 | 0 | 0 | 0 |
| k__Bacteria;p__Planctomycetes;c__Planctomycetia;o__Gemmatales;f__Gemmataceae;g__ | 0 | 0 | 0 | 0 | 0 | 0 | 0 | 0 | 0 | 0 | 0 | 0 | 0 | 0 | 0 |
| k__Bacteria;p__Planctomycetes;c__Planctomycetia;o__Gemmatales;f__Isosphaeraceae;g__ | 0 | 6.70E-05 | 0 | 0 | 0 | 0 | 0 | 0 | 0 | 0 | 0 | 0 | 0 | 0 | 0 |
| k__Bacteria;p__Planctomycetes;c__Planctomycetia;o__Pirellulales;f__Pirellulaceae;g__ | 0.00076023 | 5.36E-05 | 0.00016365 | 0 | 0.00070598 | 0.00190127 | 0.00022281 | 0.07279298 | 0.01011108 | 0 | 0.00012918 | 0 | 0.0004044 | 0.0030875 | 0.00024568 |
| k__Bacteria;p__Planctomycetes;c__Planctomycetia;o__Pirellulales;f__Pirellulaceae;g__Planctomycete | 0 | 0 | 0 | 0 | 0 | 0 | 0 | 0 | 0 | 0 | 0 | 0 | 0 | 0 | 0 |
| k__Bacteria;p__Planctomycetes;c__Planctomycetia;o__Pirellulales;f__Pirellulaceae;g__Rhodopirellula | 0 | 0 | 0 | 0 | 0 | 0 | 0 | 0 | 0 | 0 | 0 | 0 | 0 | 0 | 0 |
| k__Bacteria;p__Planctomycetes;c__Planctomycetia;o__Planctomycetales;f__Planctomycetaceae;g__Planctomyces | 0 | 0 | 0 | 0 | 0.00011552 | 5.28E-05 | 0 | 0 | 0 | 0 | 0 | 0 | 0 | 0 | 0 |
| k__Bacteria;p__Planctomycetes;c__vadinHA49;o__;f__;g__ | 0 | 0 | 0 | 0 | 0 | 0 | 0 | 0 | 0 | 0 | 0 | 0 | 0 | 0 | 0 |
| k__Bacteria;p__Proteobacteria;c__Alphaproteobacteria;o__;f__;g__ | 0.00774904 | 0.00518614 | 0.00305903 | 0 | 0.00419737 | 0.01197099 | 0.01329438 | 0 | 0.00534036 | 0.00279651 | 0.01005795 | 0.00314111 | 0 | 0.00142025 | 0.0073704 |
| k__Bacteria;p__Proteobacteria;c__Alphaproteobacteria;o__BD7-3;f__;g__ | 0.00022674 | 6.70E-05 | 0 | 0 | 0.00130927 | 0.00339765 | 0.00720422 | 0 | 0 | 0 | 0 | 0 | 0 | 0 | 0.00021292 |
| k__Bacteria;p__Proteobacteria;c__Alphaproteobacteria;o__Caulobacterales;f__Caulobacteraceae;g__ | 6.67E-05 | 0 | 0 | 0.00190062 | 0 | 0 | 1.24E-05 | 0.02891069 | 0.0192965 | 0 | 0 | 0 | 0 | 0.00034992 | 8.19E-05 |
| k__Bacteria;p__Proteobacteria;c__Alphaproteobacteria;o__Caulobacterales;f__Caulobacteraceae;g__Brevundimonas | 0 | 0 | 0 | 0 | 0 | 8.80E-05 | 0 | 0 | 0 | 0 | 0 | 0 | 0 | 0 | 0 |
| k__Bacteria;p__Proteobacteria;c__Alphaproteobacteria;o__Caulobacterales;f__Caulobacteraceae;g__Mycoplana | 0 | 0 | 0 | 0 | 0 | 0 | 0 | 0 | 0 | 0 | 0 | 0 | 0 | 0 | 0 |
| k__Bacteria;p__Proteobacteria;c__Alphaproteobacteria;o__Caulobacterales;f__Caulobacteraceae;g__Phenylobacterium | 0 | 0 | 0 | 0 | 0 | 0 | 0 | 0 | 0 | 0 | 0 | 0 | 0 | 0 | 0 |
| k__Bacteria;p__Proteobacteria;c__Alphaproteobacteria;o__Ellin329;f__;g__ | 0 | 0 | 0 | 0 | 0 | 0 | 0 | 0 | 0 | 0 | 0 | 0 | 0 | 0 | 0 |
| k__Bacteria;p__Proteobacteria;c__Alphaproteobacteria;o__Kiloniellales;f__;g__ | 1.33E-05 | 0 | 0 | 0 | 0.00011552 | 0.00033448 | 0 | 0 | 0 | 0 | 0 | 0 | 0 | 0 | 0 |
| k__Bacteria;p__Proteobacteria;c__Alphaproteobacteria;o__Kiloniellales;f__Kiloniellaceae;g__ | 5.33E-05 | 0.00017421 | 0.00041542 | 0 | 0 | 0 | 0 | 0 | 0 | 0 | 0.00125494 | 0.00037045 | 0 | 0 | 6.55E-05 |
| k__Bacteria;p__Proteobacteria;c__Alphaproteobacteria;o__Kiloniellales;f__Kiloniellaceae;g__Thalassospira | 0 | 0 | 0 | 0 | 0 | 0 | 0 | 0 | 0 | 0 | 0 | 0 | 0 | 0 | 0 |
| k__Bacteria;p__Proteobacteria;c__Alphaproteobacteria;o__Kordiimonadales;f__Kordiimonadaceae;g__ | 0 | 0 | 0 | 0 | 0 | 0 | 0 | 0 | 0 | 0 | 0 | 0 | 0 | 0 | 0 |
| k__Bacteria;p__Proteobacteria;c__Alphaproteobacteria;o__Rhizobiales;f__;g__ | 0.00014671 | 0.00022781 | 0.00018883 | 0.00086886 | 0 | 0 | 0 | 0 | 0.00469952 | 0 | 0.00012918 | 0 | 0 | 0.00055575 | 9.83E-05 |
| k__Bacteria;p__Proteobacteria;c__Alphaproteobacteria;o__Rhizobiales;f__Aurantimonadaceae;g__Martelella | 0 | 0 | 0 | 0 | 0 | 0 | 0 | 0 | 0 | 0 | 0 | 0 | 0 | 0 | 0 |
| k__Bacteria;p__Proteobacteria;c__Alphaproteobacteria;o__Rhizobiales;f__Beijerinckiaceae;g__Chelatococcus | 0 | 0 | 0 | 0.00032582 | 0 | 0 | 0 | 0 | 0 | 0 | 0 | 0 | 0 | 0 | 0 |
| k__Bacteria;p__Proteobacteria;c__Alphaproteobacteria;o__Rhizobiales;f__Bradyrhizobiaceae;g__ | 0.00037345 | 2.68E-05 | 0 | 0 | 7.70E-05 | 0.00019365 | 6.19E-05 | 0.01032525 | 0.01594987 | 0.00220151 | 0 | 0.0002624 | 0 | 0 | 0 |
| k__Bacteria;p__Proteobacteria;c__Alphaproteobacteria;o__Rhizobiales;f__Bradyrhizobiaceae;g__Balneimonas | 0 | 0 | 0 | 0 | 0 | 0 | 0 | 0 | 0 | 0 | 0 | 0 | 0 | 0 | 0 |
| k__Bacteria;p__Proteobacteria;c__Alphaproteobacteria;o__Rhizobiales;f__Bradyrhizobiaceae;g__Pseudomonas | 0 | 0 | 0 | 0 | 0 | 0 | 0 | 0 | 0 | 0 | 0 | 0 | 0 | 0 | 0 |
| k__Bacteria;p__Proteobacteria;c__Alphaproteobacteria;o__Rhizobiales;f__Cohaesibacteraceae;g__Cohaesibacter | 0 | 0 | 0 | 0 | 0 | 0 | 0 | 0 | 0 | 0 | 0 | 0 | 0 | 0 | 0 |
| k__Bacteria;p__Proteobacteria;c__Alphaproteobacteria;o__Rhizobiales;f__Hyphomicrobiaceae;g__ | 0 | 0 | 0 | 0 | 0 | 0 | 7.43E-05 | 0 | 0 | 0 | 0 | 0 | 0 | 0 | 0 |
| k__Bacteria;p__Proteobacteria;c__Alphaproteobacteria;o__Rhizobiales;f__Hyphomicrobiaceae;g__Devosia | 0 | 0 | 0 | 0 | 0.00012836 | 0.00570382 | 0.00147303 | 0 | 0 | 0 | 0 | 7.72E-06 | 0 | 0 | 0.00055687 |
| k__Bacteria;p__Proteobacteria;c__Alphaproteobacteria;o__Rhizobiales;f__Hyphomicrobiaceae;g__Rhodoplanes | 0 | 0 | 0 | 0 | 0 | 0 | 0 | 0 | 0 | 0 | 0 | 0 | 0 | 0 | 0 |
| k__Bacteria;p__Proteobacteria;c__Alphaproteobacteria;o__Rhizobiales;f__Methylobacteriaceae;g__ | 0 | 0 | 0 | 0 | 1.28E-05 | 0 | 0 | 0 | 0 | 0 | 0 | 0 | 0 | 0 | 0 |
| k__Bacteria;p__Proteobacteria;c__Alphaproteobacteria;o__Rhizobiales;f__Methylobacteriaceae;g__Methylobacterium | 0 | 0.00026802 | 0 | 0 | 0 | 0 | 0 | 0 | 0.00341783 | 0.00073384 | 0.00014764 | 3.09E-05 | 0 | 2.06E-05 | 0 |
| k__Bacteria;p__Proteobacteria;c__Alphaproteobacteria;o__Rhizobiales;f__Methylocystaceae;g__ | 0 | 0.00079065 | 0 | 0 | 0 | 0.00029927 | 0 | 0 | 0 | 0 | 0 | 0 | 0 | 0 | 0 |
| k__Bacteria;p__Proteobacteria;c__Alphaproteobacteria;o__Rhizobiales;f__Methylocystaceae;g__Methylosinus | 0 | 0 | 0 | 0 | 0 | 0 | 0 | 0 | 0 | 0 | 0 | 0 | 0 | 0 | 0 |
| k__Bacteria;p__Proteobacteria;c__Alphaproteobacteria;o__Rhizobiales;f__Methylocystaceae;g__Pleomorphomonas | 0 | 0 | 0 | 0.00211784 | 0 | 0 | 0 | 0 | 0 | 0 | 0 | 0 | 0 | 0 | 0 |
| k__Bacteria;p__Proteobacteria;c__Alphaproteobacteria;o__Rhizobiales;f__Phyllobacteriaceae;g__ | 0 | 0.00017421 | 0 | 0 | 0 | 0.00054574 | 0.00030946 | 0 | 0 | 0 | 0 | 0 | 0 | 0 | 0 |
| k__Bacteria;p__Proteobacteria;c__Alphaproteobacteria;o__Rhizobiales;f__Phyllobacteriaceae;g__Chelativorans | 0 | 0 | 0 | 0 | 0 | 1.76E-05 | 0 | 0 | 0 | 0 | 0 | 0 | 0 | 0 | 0 |
| k__Bacteria;p__Proteobacteria;c__Alphaproteobacteria;o__Rhizobiales;f__Phyllobacteriaceae;g__Hoeflea | 0 | 0 | 0 | 0 | 5.13E-05 | 0.00183086 | 8.66E-05 | 0 | 0 | 0 | 0 | 0 | 0 | 0 | 0.00019654 |
| k__Bacteria;p__Proteobacteria;c__Alphaproteobacteria;o__Rhizobiales;f__Phyllobacteriaceae;g__Mesorhizobium | 0 | 0 | 0 | 0 | 0 | 0 | 0 | 0 | 0 | 0 | 0 | 0 | 0 | 0 | 0 |
| k__Bacteria;p__Proteobacteria;c__Alphaproteobacteria;o__Rhizobiales;f__Phyllobacteriaceae;g__Phyllobacterium | 0 | 0 | 0 | 0 | 0 | 0 | 0 | 0 | 0 | 0 | 0 | 0 | 0 | 0 | 0 |
| k__Bacteria;p__Proteobacteria;c__Alphaproteobacteria;o__Rhizobiales;f__Rhizobiaceae;g__ | 0 | 0 | 0 | 0 | 0 | 0 | 0 | 0 | 0 | 0 | 0 | 0 | 0 | 0 | 0 |
| k__Bacteria;p__Proteobacteria;c__Alphaproteobacteria;o__Rhizobiales;f__Rhizobiaceae;g__Agrobacterium | 0 | 0 | 7.55E-05 | 0 | 0 | 0 | 0 | 0 | 0.09498718 | 0 | 0 | 0 | 0 | 0 | 0 |
| k__Bacteria;p__Proteobacteria;c__Alphaproteobacteria;o__Rhizobiales;f__Rhizobiaceae;g__Rhizobium | 0 | 0 | 0 | 0 | 0 | 0 | 0 | 0 | 0 | 0 | 0 | 0 | 0 | 0 | 0 |
| k__Bacteria;p__Proteobacteria;c__Alphaproteobacteria;o__Rhizobiales;f__Rhizobiaceae;g__Shinella | 0 | 0 | 0 | 0 | 0 | 0 | 0 | 0 | 7.12E-05 | 0 | 0 | 0 | 0 | 0 | 0 |
| k__Bacteria;p__Proteobacteria;c__Alphaproteobacteria;o__Rhizobiales;f__Xanthobacteraceae;g__Xanthobacter | 0 | 0 | 0 | 0 | 0 | 0 | 0 | 0 | 0 | 0 | 0 | 0 | 0 | 0 | 0 |
| k__Bacteria;p__Proteobacteria;c__Alphaproteobacteria;o__Rhodobacterales;f__Hyphomonadaceae;g__ | 8.00E-05 | 0 | 0 | 0 | 0.0001412 | 0.00144356 | 0.0001733 | 0 | 0 | 0.00182467 | 0.00064592 | 0 | 0 | 0 | 0.00019654 |
| k__Bacteria;p__Proteobacteria;c__Alphaproteobacteria;o__Rhodobacterales;f__Hyphomonadaceae;g__Maricaulis | 0 | 0 | 0 | 0 | 0 | 0 | 0 | 0 | 0 | 0 | 0 | 0 | 0 | 0 | 0 |
| k__Bacteria;p__Proteobacteria;c__Alphaproteobacteria;o__Rhodobacterales;f__Hyphomonadaceae;g__Robiginitomaculum | 0 | 0 | 0 | 0 | 0 | 0 | 0 | 0 | 0 | 0 | 0 | 0 | 0 | 0 | 0 |
| k__Bacteria;p__Proteobacteria;c__Alphaproteobacteria;o__Rhodobacterales;f__Rhodobacteraceae;g__ | 0.01765875 | 0.00786631 | 0.00309679 | 0.0015205 | 0.01417092 | 0.06744243 | 0.02705914 | 0.05420754 | 0.03375107 | 0.12625942 | 0.12645333 | 0.03865804 | 0.00895453 | 0.013832 | 0.0638277 |
| k__Bacteria;p__Proteobacteria;c__Alphaproteobacteria;o__Rhodobacterales;f__Rhodobacteraceae;g__Amaricoccus | 0 | 0 | 0 | 0 | 0 | 0 | 0 | 0 | 0 | 0 | 0 | 0 | 0 | 0.00018525 | 0 |
| k__Bacteria;p__Proteobacteria;c__Alphaproteobacteria;o__Rhodobacterales;f__Rhodobacteraceae;g__Anaerospora | 0 | 0 | 0 | 0 | 0 | 0 | 0.00033422 | 0 | 0 | 0 | 0 | 0 | 0 | 0 | 3.28E-05 |
| k__Bacteria;p__Proteobacteria;c__Alphaproteobacteria;o__Rhodobacterales;f__Rhodobacteraceae;g__Celeribacter | 0 | 0 | 0 | 0 | 0 | 0 | 0 | 0 | 0 | 0 | 0 | 0 | 0 | 0 | 0 |
| k__Bacteria;p__Proteobacteria;c__Alphaproteobacteria;o__Rhodobacterales;f__Rhodobacteraceae;g__Dinoroseobacter | 0 | 0 | 0 | 0 | 1.28E-05 | 0 | 7.43E-05 | 0 | 0 | 0 | 0 | 0 | 0 | 0 | 0.00037671 |
| k__Bacteria;p__Proteobacteria;c__Alphaproteobacteria;o__Rhodobacterales;f__Rhodobacteraceae;g__Donghicola | 0 | 0 | 0 | 0 | 0 | 0 | 0 | 0 | 0 | 0 | 0 | 0 | 0 | 0 | 0 |
| k__Bacteria;p__Proteobacteria;c__Alphaproteobacteria;o__Rhodobacterales;f__Rhodobacteraceae;g__Jannaschia | 0 | 0 | 0 | 0 | 0 | 0 | 0 | 0 | 0 | 0 | 0 | 0 | 0 | 0 | 0 |
| k__Bacteria;p__Proteobacteria;c__Alphaproteobacteria;o__Rhodobacterales;f__Rhodobacteraceae;g__Loktanella | 0 | 0 | 0 | 0 | 0.00019254 | 0.0003873 | 0.00034659 | 0 | 0 | 0.00249901 | 0 | 0 | 0 | 0 | 0.00027844 |
| k__Bacteria;p__Proteobacteria;c__Alphaproteobacteria;o__Rhodobacterales;f__Rhodobacteraceae;g__Maribius | 0 | 0 | 0 | 0 | 0 | 0 | 0 | 0 | 0 | 0 | 0 | 0 | 0 | 0 | 0 |
| k__Bacteria;p__Proteobacteria;c__Alphaproteobacteria;o__Rhodobacterales;f__Rhodobacteraceae;g__Marivita | 0 | 0 | 0 | 0 | 0 | 0 | 0 | 0 | 0 | 0 | 0 | 0 | 0 | 0 | 0 |
| k__Bacteria;p__Proteobacteria;c__Alphaproteobacteria;o__Rhodobacterales;f__Rhodobacteraceae;g__Nautella | 0 | 0 | 0 | 0 | 0 | 0 | 0 | 0 | 0 | 1.98E-05 | 0 | 0 | 0 | 0 | 0 |

| Taxon | | 1231 | 1233 | 1234 | 1235 | 1236 | 1243 | 1244 | 6473 | 6474 | 1188 | 1189 | 1190 | 1226 | 1227 | 2027 |
|---|---|---|---|---|---|---|---|---|---|---|---|---|---|---|---|---|
| | Copepod genus | Calanus | Calanus | Calanus | Calanus | Calanus | Centropages | Centropages | Centropages | Centropages | Centropages | Centropages | Centropages | Centropages | Centropages | Centropages |
| | Type of sample | FullGut | FullGut | FullGut | FullGut | FullGut | FullGut | FullGut | FullGut | FullGut | Starved | Starved | Starved | Starved | Starved | Starved |
| k__Bacteria;p__Proteobacteria;c__Alphaproteobacteria;o__Rhodobacterales;f__Rhodobacteraceae;g__Oceanicola | | 0 | 0 | 0 | 0 | 0 | 0 | 0 | 0 | 0 | 0 | 0 | 0 | 0 | 0 | 0 |
| k__Bacteria;p__Proteobacteria;c__Alphaproteobacteria;o__Rhodobacterales;f__Rhodobacteraceae;g__Octadecabacter | | 0.03899863 | 0.0276728 | 0.01043594 | 0.00287809 | 0.07561677 | 0.14099359 | 0.07669646 | 0.00309757 | 0.01317289 | 0.04779849 | 0.06422323 | 0.02380144 | 0.00344701 | 0.01836033 | 0.06099419 |
| k__Bacteria;p__Proteobacteria;c__Alphaproteobacteria;o__Rhodobacterales;f__Rhodobacteraceae;g__Paracoccus | | 1.33E-05 | 0 | 0 | 0 | 0 | 0 | 6.19E-05 | 0.00103252 | 0.00548277 | 0 | 0 | 0 | 0 | 0 | 0 |
| k__Bacteria;p__Proteobacteria;c__Alphaproteobacteria;o__Rhodobacterales;f__Rhodobacteraceae;g__Phaeobacter | | 0.0002134 | 0.00053603 | 7.55E-05 | 0 | 0.00164301 | 0.02873037 | 0.01445795 | 0 | 0 | 0 | 0 | 0 | 0.00055845 | 0 | 0.0026861 |
| k__Bacteria;p__Proteobacteria;c__Alphaproteobacteria;o__Rhodobacterales;f__Rhodobacteraceae;g__Pseudoroseobacter | | 0 | 0 | 0 | 0 | 0 | 0 | 0 | 0 | 0 | 0 | 0 | 0 | 0 | 0 | 0.00013103 |
| k__Bacteria;p__Proteobacteria;c__Alphaproteobacteria;o__Rhodobacterales;f__Rhodobacteraceae;g__Pseudoruegeria | | 0 | 0.00069685 | 0 | 0 | 0 | 0.00019365 | 0.00042087 | 0 | 0 | 0 | 0.00047983 | 0 | 0 | 0 | 0.00026206 |
| k__Bacteria;p__Proteobacteria;c__Alphaproteobacteria;o__Rhodobacterales;f__Rhodobacteraceae;g__Rhodobacter | | 2.67E-05 | 0 | 0 | 0.00010861 | 5.13E-05 | 0 | 0 | 0 | 0 | 0 | 0 | 0 | 0 | 0 | 0 |
| k__Bacteria;p__Proteobacteria;c__Alphaproteobacteria;o__Rhodobacterales;f__Rhodobacteraceae;g__Roseicyclus | | 0 | 0 | 0 | 0 | 0 | 0 | 0 | 0 | 0 | 0 | 0 | 0 | 0 | 0 | 0 |
| k__Bacteria;p__Proteobacteria;c__Alphaproteobacteria;o__Rhodobacterales;f__Rhodobacteraceae;g__Roseobacter | | 0 | 0 | 0 | 0 | 0 | 0 | 0 | 0 | 0 | 0 | 0 | 0 | 0 | 0 | 0 |
| k__Bacteria;p__Proteobacteria;c__Alphaproteobacteria;o__Rhodobacterales;f__Rhodobacteraceae;g__Rubellimicrobium | | 0 | 0 | 0 | 0 | 0 | 0 | 0.00021043 | 0 | 0 | 0 | 0 | 0 | 0 | 0 | 0 |
| k__Bacteria;p__Proteobacteria;c__Alphaproteobacteria;o__Rhodobacterales;f__Rhodobacteraceae;g__Shimia | | 0 | 0 | 0 | 0 | 0 | 0 | 0 | 0 | 0 | 0 | 0 | 0 | 0 | 0 | 0 |
| k__Bacteria;p__Proteobacteria;c__Alphaproteobacteria;o__Rhodobacterales;f__Rhodobacteraceae;g__Sulfitobacter | | 4.00E-05 | 0 | 0 | 5.43E-05 | 0.00026956 | 0.00334483 | 0.00102741 | 0 | 0 | 0 | 0 | 0 | 0 | 0 | 0.00375072 |
| k__Bacteria;p__Proteobacteria;c__Alphaproteobacteria;o__Rhodobacterales;f__Rhodobacteraceae;g__Thalassobacter | | 0 | 0 | 0 | 0 | 0 | 0 | 0 | 0 | 0 | 0 | 0 | 0 | 0 | 0 | 1.64E-05 |
| k__Bacteria;p__Proteobacteria;c__Alphaproteobacteria;o__Rhodobacterales;f__Rhodobacteraceae;g__Thalassobius | | 0 | 0 | 0 | 0 | 3.85E-05 | 1.76E-05 | 0.00025995 | 0 | 0 | 0 | 0 | 0 | 0 | 0 | 0.00055687 |
| k__Bacteria;p__Proteobacteria;c__Alphaproteobacteria;o__Rhodobacterales;f__Rhodobacteraceae;g__Tropicibacter | | 0 | 0 | 0 | 0 | 0 | 0 | 1.24E-05 | 0 | 0 | 0 | 0 | 0 | 0 | 0 | 6.55E-05 |
| k__Bacteria;p__Proteobacteria;c__Alphaproteobacteria;o__Rhodospirillales;f__;g__ | | 0 | 6.70E-05 | 0 | 0 | 0 | 0.00042251 | 0 | 0 | 0 | 0 | 0 | 0 | 0 | 0 | 0 |
| k__Bacteria;p__Proteobacteria;c__Alphaproteobacteria;o__Rhodospirillales;f__Acetobacteraceae;g__ | | 0 | 0 | 0 | 0.00048873 | 0 | 0 | 0 | 0 | 0 | 0.0006545 | 0 | 0 | 0 | 0 | 0 |
| k__Bacteria;p__Proteobacteria;c__Alphaproteobacteria;o__Rhodospirillales;f__Acetobacteraceae;g__Roseococcus | | 0 | 0 | 0 | 0 | 0 | 0 | 0 | 0 | 0 | 0 | 0 | 0 | 0 | 0 | 0 |
| k__Bacteria;p__Proteobacteria;c__Alphaproteobacteria;o__Rhodospirillales;f__Rhodospirillaceae;g__ | | 0.00352108 | 0.00176892 | 0.00166169 | 0.00190062 | 0.00175853 | 0.00255264 | 0.00298319 | 0 | 0 | 0 | 0.00108884 | 0.00023925 | 0 | 0 | 0.00434035 |
| k__Bacteria;p__Proteobacteria;c__Alphaproteobacteria;o__Rhodospirillales;f__Rhodospirillaceae;g__Skermanella | | 0 | 0 | 0 | 0 | 0 | 0 | 0 | 0 | 0 | 0 | 0 | 0 | 0 | 0 | 0 |
| k__Bacteria;p__Proteobacteria;c__Alphaproteobacteria;o__Rickettsiales;f__;g__ | | 0.0006402 | 2.68E-05 | 0 | 0 | 0.00029523 | 0.00044011 | 0.00011141 | 0 | 0 | 0 | 0.00035064 | 0.00050165 | 0 | 0.00152317 | 9.83E-05 |
| k__Bacteria;p__Proteobacteria;c__Alphaproteobacteria;o__Rickettsiales;f__AEGEAN_112;g__ | | 0 | 1.34E-05 | 0 | 0 | 0 | 0 | 0 | 0 | 0 | 0 | 0.00033219 | 0 | 0 | 0 | 0 |
| k__Bacteria;p__Proteobacteria;c__Alphaproteobacteria;o__Rickettsiales;f__Pelagibacteraceae;g__ | | 0.0196727 | 0.01456675 | 0.01451465 | 5.43E-05 | 0.00584037 | 0.04862334 | 0.02380363 | 0 | 0.04564227 | 0.01443871 | 0.05228288 | 0.0129812 | 0.0102255 | 0.01368792 | 0.02628777 |
| k__Bacteria;p__Proteobacteria;c__Alphaproteobacteria;o__Rickettsiales;f__Rickettsiaceae;g__ | | 0 | 1.34E-05 | 0 | 0 | 0 | 0 | 0 | 0 | 0 | 0 | 0 | 0 | 0 | 0 | 0 |
| k__Bacteria;p__Proteobacteria;c__Alphaproteobacteria;o__Rickettsiales;f__mitochondria;g__ | | 8.00E-05 | 0 | 0 | 0 | 0 | 0 | 0 | 0 | 0 | 0 | 0 | 0 | 0 | 0 | 0 |
| k__Bacteria;p__Proteobacteria;c__Alphaproteobacteria;o__Rickettsiales;f__mitochondria;g__Carludovica | | 0 | 0 | 0 | 0 | 0 | 0 | 0 | 0 | 0 | 0 | 0 | 0 | 0 | 0 | 0 |
| k__Bacteria;p__Proteobacteria;c__Alphaproteobacteria;o__Rickettsiales;f__mitochondria;g__Citrullus | | 0 | 0 | 0 | 0 | 0 | 0 | 0 | 0 | 0 | 0 | 0 | 0 | 0 | 0 | 0 |
| k__Bacteria;p__Proteobacteria;c__Alphaproteobacteria;o__Rickettsiales;f__mitochondria;g__Lupinus | | 0 | 0 | 0 | 0 | 0 | 0 | 0 | 0 | 0 | 0 | 0 | 0 | 0 | 0 | 0 |
| k__Bacteria;p__Proteobacteria;c__Alphaproteobacteria;o__Rickettsiales;f__mitochondria;g__Nageia | | 0 | 0 | 0 | 0 | 0 | 5.28E-05 | 0 | 0.02065049 | 0 | 0 | 0 | 0 | 0 | 0 | 0 |
| k__Bacteria;p__Proteobacteria;c__Alphaproteobacteria;o__Rickettsiales;f__mitochondria;g__Nelumbo | | 0.00025341 | 0 | 0 | 0 | 0 | 0 | 0 | 0 | 0 | 0 | 0 | 0 | 0 | 0 | 0 |
| k__Bacteria;p__Proteobacteria;c__Alphaproteobacteria;o__Rickettsiales;f__mitochondria;g__Oenothera | | 0 | 0 | 0 | 0 | 0 | 0 | 0 | 0 | 0 | 0 | 0 | 0 | 0 | 0 | 0 |
| k__Bacteria;p__Proteobacteria;c__Alphaproteobacteria;o__Rickettsiales;f__mitochondria;g__Phyllocladus | | 0 | 0 | 0 | 0 | 0 | 0 | 0 | 0.00051626 | 0 | 0 | 0 | 0 | 0 | 0 | 0 |
| k__Bacteria;p__Proteobacteria;c__Alphaproteobacteria;o__Rickettsiales;f__mitochondria;g__Zea | | 0.00065353 | 0 | 0 | 0.00119468 | 0 | 0 | 0 | 0 | 0 | 0 | 0 | 0 | 0 | 0 | 0 |
| k__Bacteria;p__Proteobacteria;c__Alphaproteobacteria;o__Sphingomonadales;f__;g__ | | 0 | 0 | 0 | 0 | 0 | 0 | 0 | 0 | 7.12E-05 | 0 | 0 | 0 | 0 | 0 | 6.55E-05 |
| k__Bacteria;p__Proteobacteria;c__Alphaproteobacteria;o__Sphingomonadales;f__Erythrobacteraceae;g__ | | 0 | 0 | 0 | 0 | 0 | 0 | 0 | 0 | 0 | 0 | 0 | 0 | 0 | 0 | 0 |
| k__Bacteria;p__Proteobacteria;c__Alphaproteobacteria;o__Sphingomonadales;f__Erythrobacteraceae;g__Altererythrobacter | | 0 | 0 | 0 | 0 | 0 | 0 | 0 | 0 | 0 | 0 | 0 | 0 | 0 | 0 | 0 |
| k__Bacteria;p__Proteobacteria;c__Alphaproteobacteria;o__Sphingomonadales;f__Erythrobacteraceae;g__Erythrobacter | | 0 | 0 | 0 | 0 | 0 | 0 | 0 | 0 | 0 | 0 | 0 | 0 | 0 | 0 | 0 |
| k__Bacteria;p__Proteobacteria;c__Alphaproteobacteria;o__Sphingomonadales;f__Erythrobacteraceae;g__Lutibacterium | | 0 | 0 | 0 | 0 | 0 | 0 | 0 | 0 | 0 | 0 | 0 | 0 | 0 | 0 | 0 |
| k__Bacteria;p__Proteobacteria;c__Alphaproteobacteria;o__Sphingomonadales;f__Sphingomonadaceae;g__ | | 0.00013337 | 4.02E-05 | 5.04E-05 | 0.00135759 | 0 | 0.00026407 | 0 | 0.02219928 | 0.00056964 | 9.92E-05 | 1.85E-05 | 0.00030099 | 5.78E-05 | 4.12E-05 | 6.55E-05 |
| k__Bacteria;p__Proteobacteria;c__Alphaproteobacteria;o__Sphingomonadales;f__Sphingomonadaceae;g__Kaistobacter | | 8.00E-05 | 0 | 0 | 0 | 0 | 0 | 0 | 0 | 0 | 0 | 0 | 0 | 0 | 0.000494 | 0 |
| k__Bacteria;p__Proteobacteria;c__Alphaproteobacteria;o__Sphingomonadales;f__Sphingomonadaceae;g__Novosphingobium | | 0 | 0 | 0 | 0 | 0 | 1.76E-05 | 0 | 0.03097574 | 0.01602108 | 0.00339151 | 0 | 0.00016979 | 0 | 0 | 0 |
| k__Bacteria;p__Proteobacteria;c__Alphaproteobacteria;o__Sphingomonadales;f__Sphingomonadaceae;g__Sphingobium | | 0 | 0 | 8.81E-05 | 0.00043443 | 0 | 0 | 0 | 0 | 0 | 0 | 0 | 0 | 0 | 0 | 0 |
| k__Bacteria;p__Proteobacteria;c__Alphaproteobacteria;o__Sphingomonadales;f__Sphingomonadaceae;g__Sphingomonas | | 0.00030676 | 0.00068344 | 0.00105744 | 0.00206354 | 0.0001797 | 0.00128512 | 0.00023519 | 0.04594734 | 0.03702649 | 0.00259818 | 0.00053519 | 0.00053252 | 0.00358181 | 0.00277875 | 9.83E-05 |
| k__Bacteria;p__Proteobacteria;c__Alphaproteobacteria;o__Sphingomonadales;f__Sphingomonadaceae;g__Sphingopyxis | | 0 | 0 | 0 | 0 | 0 | 0 | 0 | 0 | 0 | 0 | 0 | 0 | 0 | 0 | 0 |
| k__Bacteria;p__Proteobacteria;c__Alphaproteobacteria;o__Sphingomonadales;f__Sphingomonadaceae;g__Zymomonas | | 0 | 0 | 0 | 0 | 0 | 0 | 0 | 0 | 0 | 0 | 0 | 0 | 0 | 0 | 1.64E-05 |
| k__Bacteria;p__Proteobacteria;c__Betaproteobacteria;o__;f__;g__ | | 0.00129373 | 0.00203693 | 0.0013218 | 0.00076025 | 0.0006418 | 0.00135554 | 0.00155968 | 0 | 0.01217602 | 0 | 0 | 0 | 0.00084731 | 0.0386555 | 0.00232577 |
| k__Bacteria;p__Proteobacteria;c__Betaproteobacteria;o__Burkholderiales;Comamonadaceae;g__ | | 5.33E-05 | 0.00053603 | 5.04E-05 | 0.0091773 | 3.85E-05 | 0.00029927 | 4.95E-05 | 0.02891069 | 0.00477072 | 0.00249901 | 0.00014764 | 0.00013892 | 0.000674 | 0.00034992 | 1.64E-05 |
| k__Bacteria;p__Proteobacteria;c__Betaproteobacteria;o__Burkholderiales;f__;g__ | | 0.00034677 | 0.00087106 | 0.00083085 | 0 | 0 | 0.00059855 | 6.19E-05 | 0.0191017 | 0.00683566 | 0 | 0 | 0 | 0.00088582 | 0.0016055 | 0 |
| k__Bacteria;p__Proteobacteria;c__Betaproteobacteria;o__Burkholderiales;f__Alcaligenaceae;g__ | | 0.00016005 | 0.00018761 | 0 | 0 | 0 | 0 | 0 | 0 | 0 | 0 | 0 | 0 | 0 | 0 | 0 |
| k__Bacteria;p__Proteobacteria;c__Betaproteobacteria;o__Burkholderiales;f__Alcaligenaceae;g__Achromobacter | | 9.34E-05 | 0.00032162 | 0.00042801 | 0 | 0 | 0.00024646 | 0 | 0 | 0 | 0 | 0 | 0 | 0.00310039 | 0.00053517 | 0 |
| k__Bacteria;p__Proteobacteria;c__Betaproteobacteria;o__Burkholderiales;f__Alcaligenaceae;g__Sutterella | | 0 | 0 | 0 | 0 | 0 | 0 | 0 | 0 | 0 | 0 | 0 | 0 | 0 | 0 | 0 |
| k__Bacteria;p__Proteobacteria;c__Betaproteobacteria;o__Burkholderiales;f__Burkholderiaceae;g__ | | 0 | 0.00056284 | 0.00018883 | 0 | 0 | 0.00054574 | 3.71E-05 | 0 | 0 | 0 | 0 | 0 | 0.00046217 | 0.0003705 | 0 |
| k__Bacteria;p__Proteobacteria;c__Betaproteobacteria;o__Burkholderiales;f__Burkholderiaceae;g__Burkholderia | | 0 | 0 | 0 | 0 | 0 | 0 | 0 | 0 | 0.00911421 | 0 | 0 | 0 | 0 | 0 | 0 |
| k__Bacteria;p__Proteobacteria;c__Betaproteobacteria;o__Burkholderiales;f__Burkholderiaceae;g__Lautropia | | 0 | 0 | 0 | 0 | 0 | 0 | 0 | 0 | 0 | 0 | 0 | 0 | 0 | 0 | 0 |
| k__Bacteria;p__Proteobacteria;c__Betaproteobacteria;o__Burkholderiales;f__Comamonadaceae;g__Acidovorax | | 0.00161383 | 0.00026802 | 0.00224077 | 0 | 0.0001412 | 0 | 0 | 0 | 0 | 0 | 0 | 0 | 0 | 0 | 0 |
| k__Bacteria;p__Proteobacteria;c__Betaproteobacteria;o__Burkholderiales;f__Comamonadaceae;g__Comamonas | | 0.0001067 | 0 | 0 | 0 | 0 | 0 | 0 | 0.01806918 | 0 | 0 | 0 | 0 | 0 | 0 | 0 |
| k__Bacteria;p__Proteobacteria;c__Betaproteobacteria;o__Burkholderiales;f__Comamonadaceae;g__Curvibacter | | 0.00013337 | 0 | 0.00012589 | 0.00124898 | 7.70E-05 | 0 | 0 | 0 | 0.00939903 | 0.00075367 | 0 | 0 | 0.00044291 | 0.00043225 | 0 |
| k__Bacteria;p__Proteobacteria;c__Betaproteobacteria;o__Burkholderiales;f__Comamonadaceae;g__Delftia | | 0.00029342 | 0.00067004 | 0.00174981 | 0 | 0 | 0.00100345 | 3.71E-05 | 0 | 0 | 0.00126934 | 9.23E-05 | 0.00011577 | 0.00504535 | 0.00191425 | 0 |
| k__Bacteria;p__Proteobacteria;c__Betaproteobacteria;o__Burkholderiales;f__Comamonadaceae;g__Hydrogenophaga | | 0 | 0 | 0 | 0.00086886 | 0 | 0 | 0 | 0 | 0 | 0 | 0.00066438 | 0 | 0 | 0 | 3.28E-05 |
| k__Bacteria;p__Proteobacteria;c__Betaproteobacteria;o__Burkholderiales;f__Comamonadaceae;g__Leptothrix | | 0 | 0 | 0 | 5.43E-05 | 0 | 0 | 0 | 0 | 0 | 0 | 0 | 0 | 7.70E-05 | 0 | 0 |
| k__Bacteria;p__Proteobacteria;c__Betaproteobacteria;o__Burkholderiales;f__Comamonadaceae;g__Limnohabitans | | 0 | 0 | 0 | 0 | 0 | 0 | 0 | 0 | 0 | 0 | 0 | 0 | 0 | 0 | 0 |
| k__Bacteria;p__Proteobacteria;c__Betaproteobacteria;o__Burkholderiales;f__Comamonadaceae;g__Methylibium | | 9.34E-05 | 0 | 0 | 0 | 0 | 0 | 0 | 0 | 0 | 0 | 0 | 0 | 0 | 0 | 0 |
| k__Bacteria;p__Proteobacteria;c__Betaproteobacteria;o__Burkholderiales;f__Comamonadaceae;g__Polaromonas | | 0 | 0 | 0 | 0 | 0 | 0 | 0 | 0 | 0 | 0 | 0 | 0 | 0 | 0 | 0 |
| k__Bacteria;p__Proteobacteria;c__Betaproteobacteria;o__Burkholderiales;f__Comamonadaceae;g__RS62 | | 0 | 0 | 0 | 0 | 0 | 0 | 9.90E-05 | 0 | 0 | 0 | 0.00040601 | 0 | 0 | 0 | 0 |
| k__Bacteria;p__Proteobacteria;c__Betaproteobacteria;o__Burkholderiales;f__Comamonadaceae;g__Rhodoferax | | 0 | 0 | 0 | 0 | 0 | 0 | 0 | 0 | 0 | 0 | 0 | 0 | 0 | 0 | 3.28E-05 |
| k__Bacteria;p__Proteobacteria;c__Betaproteobacteria;o__Burkholderiales;f__Comamonadaceae;g__Roseateles | | 0 | 0 | 0 | 0 | 0 | 0 | 0 | 0 | 0 | 0 | 0 | 0 | 1.93E-05 | 0 | 0 |
| k__Bacteria;p__Proteobacteria;c__Betaproteobacteria;o__Burkholderiales;f__Comamonadaceae;g__Schlegelella | | 0 | 0 | 0 | 0 | 0 | 0 | 0 | 0 | 0 | 0 | 0 | 0 | 0 | 0 | 0 |
| k__Bacteria;p__Proteobacteria;c__Betaproteobacteria;o__Burkholderiales;f__Comamonadaceae;g__Tepidimonas | | 0 | 0 | 0 | 5.43E-05 | 0 | 0 | 0 | 0 | 0 | 0 | 0 | 0 | 0 | 0 | 0 |
| k__Bacteria;p__Proteobacteria;c__Betaproteobacteria;o__Burkholderiales;f__Comamonadaceae;g__Variovorax | | 5.33E-05 | 0 | 0.00012589 | 0 | 0 | 0 | 9.90E-05 | 0 | 0 | 0 | 7.38E-05 | 7.72E-05 | 0.00217605 | 0 | 4.91E-05 |
| k__Bacteria;p__Proteobacteria;c__Betaproteobacteria;o__Burkholderiales;f__Oxalobacteraceae;g__ | | 0 | 2.68E-05 | 0.00431789 | 0 | 3.85E-05 | 0.00012323 | 8.66E-05 | 0 | 0.00056964 | 0 | 0 | 1.54E-05 | 1.93E-05 | 0.02072741 | 0 |
| k__Bacteria;p__Proteobacteria;c__Betaproteobacteria;o__Burkholderiales;f__Oxalobacteraceae;g__Cupriavidus | | 0 | 0 | 0 | 0 | 0 | 0 | 0 | 0 | 0 | 0 | 0 | 0 | 0 | 0 | 0 |
| k__Bacteria;p__Proteobacteria;c__Betaproteobacteria;o__Burkholderiales;f__Oxalobacteraceae;g__Herbaspirillum | | 0 | 0 | 0 | 0 | 0 | 0 | 0 | 0 | 0 | 0 | 5.54E-05 | 0 | 0 | 0 | 0 |
| k__Bacteria;p__Proteobacteria;c__Betaproteobacteria;o__Burkholderiales;f__Oxalobacteraceae;g__Janthinobacterium | | 0 | 0 | 5.04E-05 | 0 | 0.00025672 | 0 | 2.48E-05 | 0 | 0.00519795 | 0 | 0 | 0 | 0 | 0.00471358 | 0 |
| k__Bacteria;p__Proteobacteria;c__Betaproteobacteria;o__Burkholderiales;f__Oxalobacteraceae;g__Massilia | | 0 | 0 | 0 | 0 | 0 | 0 | 0 | 0 | 0.00014241 | 0 | 0 | 0 | 0 | 0.00014408 | 0 |

| Taxon | 1231 | 1233 | 1234 | 1235 | 1236 | 1243 | 1244 | 6473 | 6474 | 1188 | 1189 | 1190 | 1226 | 1227 | 2027 |
|---|---|---|---|---|---|---|---|---|---|---|---|---|---|---|---|
| Copepod genus | Calanus | Calanus | Calanus | Calanus | Calanus | Centropages | Centropages | Centropages | Centropages | Centropages | Centropages | Centropages | Centropages | Centropages | Centropages |
| Type of sample | FullGut | FullGut | FullGut | FullGut | FullGut | FullGut | FullGut | FullGut | FullGut | Starved | Starved | Starved | Starved | Starved | Starved |
| k__Bacteria;p__Proteobacteria;c__Betaproteobacteria;o__Burkholderiales;f__Oxalobacteraceae;g__Polynucleobacter | 0 | 0 | 0 | 0 | 0 | 0 | 0 | 0 | 0 | 0 | 0 | 0 | 0 | 0 | 0 |
| k__Bacteria;p__Proteobacteria;c__Betaproteobacteria;o__Burkholderiales;f__Oxalobacteraceae;g__Ralstonia | 5.33E-05 | 0.00026802 | 3.78E-05 | 0.00103177 | 2.57E-05 | 0.00014084 | 0 | 0.01703665 | 0.01181999 | 0.00067434 | 1.85E-05 | 0.00020066 | 0.00030811 | 0.00034992 | 0 |
| k__Bacteria;p__Proteobacteria;c__Betaproteobacteria;o__Ellin6067;f__;g__ | 8.00E-05 | 0 | 0 | 0 | 0 | 0 | 0 | 0 | 0 | 0 | 0 | 0 | 0 | 0 | 0 |
| k__Bacteria;p__Proteobacteria;c__Betaproteobacteria;o__Methylophilales;f__Methylophilaceae;g__ | 0.00130707 | 0.00013401 | 0.00101968 | 0.00059734 | 0.00062896 | 0.00174284 | 0.00122546 | 0 | 0 | 0 | 0.00097811 | 0.00018523 | 0.00098211 | 0 | 0.00127754 |
| k__Bacteria;p__Proteobacteria;c__Betaproteobacteria;o__Methylophilales;f__Methylophilaceae;g__Methylotenera | 0 | 0 | 0 | 0 | 0 | 0 | 0 | 0 | 0 | 0 | 0 | 0 | 0 | 0 | 0 |
| k__Bacteria;p__Proteobacteria;c__Betaproteobacteria;o__Neisseriales;f__Neisseriaceae;g__ | 0 | 0.00018761 | 0 | 0.00076025 | 6.42E-05 | 0.00022886 | 0 | 0 | 0 | 0.00150734 | 0 | 4.63E-05 | 0 | 0.00039108 | 0 |
| k__Bacteria;p__Proteobacteria;c__Betaproteobacteria;o__Neisseriales;f__Neisseriaceae;g__Kingella | 0 | 0 | 0 | 0 | 0 | 0 | 0 | 0 | 0 | 0 | 0 | 0 | 0 | 0 | 0 |
| k__Bacteria;p__Proteobacteria;c__Betaproteobacteria;o__Neisseriales;f__Neisseriaceae;g__Microvirgula | 0 | 0 | 0 | 0 | 0 | 0 | 0 | 0 | 0 | 0 | 0 | 0 | 0 | 0 | 0 |
| k__Bacteria;p__Proteobacteria;c__Betaproteobacteria;o__Neisseriales;f__Neisseriaceae;g__Neisseria | 6.67E-05 | 0.00089786 | 0 | 0.00076025 | 0.00010269 | 0 | 3.71E-05 | 0.01342282 | 0.00825976 | 0 | 0 | 0 | 0.00063548 | 0 | 0 |
| k__Bacteria;p__Proteobacteria;c__Betaproteobacteria;o__Nitrosomonadales;f__Nitrosomonadaceae;g__ | 0 | 0 | 0 | 0 | 0 | 0 | 0 | 0 | 0 | 0 | 0 | 0 | 0 | 0 | 0 |
| k__Bacteria;p__Proteobacteria;c__Betaproteobacteria;o__Procabacteriales;f__Procabacteriaceae;g__ | 0 | 0 | 0.0001133 | 0 | 0 | 0 | 0 | 0 | 0 | 0 | 0 | 0 | 0 | 0 | 0 |
| k__Bacteria;p__Proteobacteria;c__Betaproteobacteria;o__Rhodocyclales;f__Rhodocyclaceae;g__ | 0.00325433 | 0.00191632 | 0.00127145 | 0 | 0.00223346 | 0.00741145 | 0.00516179 | 0 | 0 | 0.0005355 | 0.00498284 | 0.0028324 | 0 | 0 | 0.0059127 |
| k__Bacteria;p__Proteobacteria;c__Betaproteobacteria;o__Rhodocyclales;f__Rhodocyclaceae;g__Dechloromonas | 0 | 0 | 0 | 0 | 0 | 0 | 0 | 0 | 0 | 0 | 0 | 0 | 0 | 0 | 0 |
| k__Bacteria;p__Proteobacteria;c__Betaproteobacteria;o__Rhodocyclales;f__Rhodocyclaceae;g__Hydrogenophilus | 0 | 0 | 0 | 0.00043443 | 0 | 0 | 0 | 0 | 0 | 0 | 0.00018455 | 0 | 0 | 0 | 0 |
| k__Bacteria;p__Proteobacteria;c__Betaproteobacteria;o__Rhodocyclales;f__Rhodocyclaceae;g__Methyloversatilis | 0 | 0 | 0 | 0 | 0 | 0 | 1.24E-05 | 0 | 0 | 0 | 0 | 0 | 0 | 0 | 0 |
| k__Bacteria;p__Proteobacteria;c__Betaproteobacteria;o__Rhodocyclales;f__Rhodocyclaceae;g__Propionivibrio | 0 | 0 | 0 | 0 | 0 | 0 | 0 | 0 | 0 | 0 | 0 | 0 | 0 | 0 | 0 |
| k__Bacteria;p__Proteobacteria;c__Betaproteobacteria;o__Rhodocyclales;f__Rhodocyclaceae;g__Uliginosibacterium | 0 | 0 | 0 | 0 | 0 | 7.04E-05 | 0 | 0 | 0 | 0 | 0 | 0 | 0 | 0 | 0 |
| k__Bacteria;p__Proteobacteria;c__Betaproteobacteria;o__Rhodocyclales;f__Rhodocyclaceae;g__Zoogloea | 0 | 0 | 0 | 0 | 0 | 0 | 0 | 0 | 0 | 0 | 0 | 0 | 0 | 0 | 0 |
| k__Bacteria;p__Proteobacteria;c__Betaproteobacteria;o__SC-I-84;f__;g__ | 0.0001067 | 0 | 0 | 0 | 0 | 0 | 0 | 0 | 0 | 0 | 0 | 0 | 0 | 0 | 0 |
| k__Bacteria;p__Proteobacteria;c__Betaproteobacteria;o__Tremblayales;f__;g__ | 0 | 0 | 0 | 0 | 0 | 0 | 0 | 0 | 0 | 0 | 0 | 0 | 0 | 0 | 0 |
| k__Bacteria;p__Proteobacteria;c__Deltaproteobacteria;o__;f__;g__ | 0 | 0 | 0 | 0 | 0 | 0 | 0 | 0 | 0 | 0 | 0 | 0 | 0 | 0 | 0 |
| k__Bacteria;p__Proteobacteria;c__Deltaproteobacteria;o__Bdellovibrionales;f__Bacteriovoracaceae;g__ | 0.00037345 | 0.00012061 | 0 | 0 | 0 | 0.00017604 | 0 | 0 | 0 | 0 | 0 | 0 | 0 | 0 | 0.00021292 |
| k__Bacteria;p__Proteobacteria;c__Deltaproteobacteria;o__Bdellovibrionales;f__Bacteriovoracaceae;g__Bacteriovorax | 0.00018672 | 0.00026802 | 0.00022659 | 0 | 0.00075732 | 0.00077459 | 0.00179487 | 0 | 0 | 0 | 0 | 0 | 0 | 2.06E-05 | 6.55E-05 |
| k__Bacteria;p__Proteobacteria;c__Deltaproteobacteria;o__Bdellovibrionales;f__Bdellovibrionaceae;g__Bdellovibrio | 0 | 0 | 0 | 0 | 0 | 0 | 0 | 0 | 0 | 0 | 0 | 0 | 0 | 0 | 0 |
| k__Bacteria;p__Proteobacteria;c__Deltaproteobacteria;o__Desulfarculales;f__Desulfarculaceae;g__ | 0 | 0 | 0 | 0 | 0 | 0 | 0 | 0 | 0 | 0 | 0 | 0 | 0 | 0 | 0 |
| k__Bacteria;p__Proteobacteria;c__Deltaproteobacteria;o__Desulfobacterales;f__Desulfobacteraceae;g__ | 0 | 0 | 0 | 0 | 0 | 0 | 0 | 0 | 0 | 0 | 0 | 0 | 0 | 0 | 0 |
| k__Bacteria;p__Proteobacteria;c__Deltaproteobacteria;o__Desulfobacterales;f__Desulfobacteraceae;g__Desulfococcus | 0 | 0 | 0 | 0 | 0 | 0 | 0 | 0 | 0 | 0 | 0 | 0 | 0 | 0 | 0 |
| k__Bacteria;p__Proteobacteria;c__Deltaproteobacteria;o__Desulfobacterales;f__Desulfobacteraceae;g__Desulfofrigus | 0 | 0 | 0 | 0 | 0 | 0 | 0 | 0 | 0 | 0 | 0 | 0 | 0 | 0 | 0 |
| k__Bacteria;p__Proteobacteria;c__Deltaproteobacteria;o__Desulfobacterales;f__Desulfobacteraceae;g__Desulfosarcina | 0 | 0 | 0 | 0 | 0 | 0 | 0 | 0 | 0 | 0 | 0 | 0 | 0 | 0 | 0 |
| k__Bacteria;p__Proteobacteria;c__Deltaproteobacteria;o__Desulfobacterales;f__Desulfobulbaceae;g__ | 0 | 0 | 0 | 0 | 0 | 5.28E-05 | 0 | 0 | 0 | 0 | 0 | 0 | 0 | 0 | 0 |
| k__Bacteria;p__Proteobacteria;c__Deltaproteobacteria;o__Desulfobacterales;f__Nitrospinaceae;g__Nitrospina | 0 | 0 | 0 | 0 | 0 | 0 | 0 | 0 | 0 | 0 | 0 | 0 | 0 | 0 | 0 |
| k__Bacteria;p__Proteobacteria;c__Deltaproteobacteria;o__Desulfovibrionales;f__Desulfovibrionaceae;g__ | 0 | 0 | 0 | 0 | 0 | 0 | 0 | 0 | 0 | 0 | 0 | 0 | 0 | 0 | 0 |
| k__Bacteria;p__Proteobacteria;c__Deltaproteobacteria;o__Desulfovibrionales;f__Desulfovibrionaceae;g__Desulfovibrio | 0 | 0 | 0 | 0 | 0 | 0 | 0 | 0 | 0 | 0 | 0 | 0 | 0 | 0 | 0 |
| k__Bacteria;p__Proteobacteria;c__Deltaproteobacteria;o__Desulfuromonadales;f__Desulfuromonadaceae;g__ | 0 | 0 | 0 | 0 | 0 | 0 | 0 | 0 | 0 | 0 | 0 | 0 | 0 | 0 | 0 |
| k__Bacteria;p__Proteobacteria;c__Deltaproteobacteria;o__Desulfuromonadales;f__Desulfuromonadaceae;g__Desulfuromusa | 0 | 0 | 0 | 0 | 0 | 0 | 0 | 0 | 0 | 0 | 0 | 0 | 0 | 0 | 0 |
| k__Bacteria;p__Proteobacteria;c__Deltaproteobacteria;o__FAC87;f__;g__ | 0 | 0 | 0 | 0 | 0 | 0 | 0 | 0 | 0 | 0 | 0 | 0 | 0 | 0 | 0 |
| k__Bacteria;p__Proteobacteria;c__Deltaproteobacteria;o__MIZ46;f__;g__ | 0 | 0 | 0 | 0 | 0 | 0 | 0 | 0 | 0 | 0 | 0 | 0 | 0 | 0 | 0 |
| k__Bacteria;p__Proteobacteria;c__Deltaproteobacteria;o__Myxococcales;f__;g__ | 0 | 0 | 0 | 0.00048873 | 0 | 3.52E-05 | 0 | 0 | 0 | 0 | 0 | 0 | 0 | 0 | 0 |
| k__Bacteria;p__Proteobacteria;c__Deltaproteobacteria;o__Myxococcales;f__0319-6G20;g__ | 0 | 0 | 0 | 0 | 0 | 0 | 0 | 0 | 0 | 0 | 5.54E-05 | 0.00014664 | 0 | 0 | 0 |
| k__Bacteria;p__Proteobacteria;c__Deltaproteobacteria;o__Myxococcales;f__OM27;g__ | 0 | 0 | 0.00023918 | 0 | 0 | 0.00012323 | 0 | 0 | 0 | 0 | 0 | 0 | 0 | 0 | 0 |
| k__Bacteria;p__Proteobacteria;c__Deltaproteobacteria;o__NB1-j;f__;g__ | 0 | 0 | 0 | 0 | 0 | 0 | 0 | 0 | 0 | 0 | 0 | 0 | 0 | 0 | 0 |
| k__Bacteria;p__Proteobacteria;c__Deltaproteobacteria;o__NB1-j;f__JTB38;g__ | 0 | 0 | 8.81E-05 | 0 | 0 | 0 | 0 | 0 | 0 | 0 | 9.23E-05 | 0 | 0 | 0 | 1.64E-05 |
| k__Bacteria;p__Proteobacteria;c__Deltaproteobacteria;o__PB19;f__;g__ | 0 | 0 | 0 | 0 | 0 | 0 | 0 | 0 | 0 | 0 | 1.85E-05 | 0 | 0 | 0 | 0 |
| k__Bacteria;p__Proteobacteria;c__Deltaproteobacteria;o__Sva0853;f__;g__ | 0 | 1.34E-05 | 0 | 0 | 0 | 0 | 0 | 0 | 0 | 0 | 0.00016609 | 0 | 0 | 0 | 0 |
| k__Bacteria;p__Proteobacteria;c__Deltaproteobacteria;o__Sva0853;f__S25_1238;g__ | 0 | 0 | 0 | 0 | 0 | 0 | 0 | 0 | 0 | 0 | 0 | 0 | 0 | 0 | 0 |
| k__Bacteria;p__Proteobacteria;c__Deltaproteobacteria;o__Sva0853;f__SAR324;g__ | 0 | 0 | 7.55E-05 | 0 | 0 | 0 | 0 | 0 | 0 | 0 | 3.69E-05 | 0 | 0 | 0 | 3.28E-05 |
| k__Bacteria;p__Proteobacteria;c__Deltaproteobacteria;o__Syntrophobacterales;f__Syntrophobacteraceae;g__ | 0 | 0 | 0 | 0 | 0 | 0 | 0 | 0 | 0 | 0 | 0 | 0 | 0 | 0 | 0 |
| k__Bacteria;p__Proteobacteria;c__Epsilonproteobacteria;o__Campylobacterales;f__Campylobacteraceae;g__Arcobacter | 0 | 0 | 0.00020142 | 0.00097746 | 3.85E-05 | 0.00345046 | 0 | 0 | 0 | 0 | 0.00291588 | 0.0012194 | 0 | 0 | 8.19E-05 |
| k__Bacteria;p__Proteobacteria;c__Epsilonproteobacteria;o__Campylobacterales;f__Campylobacteraceae;g__Campylobacter | 0.0002134 | 0 | 0 | 0 | 0 | 0 | 0 | 0 | 0 | 0 | 0 | 0 | 0 | 0 | 0 |
| k__Bacteria;p__Proteobacteria;c__Epsilonproteobacteria;o__Campylobacterales;f__Campylobacteraceae;g__Sulfurospirillum | 0 | 0 | 0 | 0 | 0 | 0 | 0 | 0 | 0 | 0 | 0.00014764 | 0 | 0 | 0 | 0 |
| k__Bacteria;p__Proteobacteria;c__Epsilonproteobacteria;o__Campylobacterales;f__Helicobacteraceae;g__ | 0 | 0 | 0 | 0 | 0 | 0.00077459 | 0 | 0 | 0 | 0 | 0 | 0 | 0 | 0 | 0 |
| k__Bacteria;p__Proteobacteria;c__Epsilonproteobacteria;o__Campylobacterales;f__Helicobacteraceae;g__Sulfurimonas | 0 | 0 | 0 | 0 | 0 | 0 | 0 | 0 | 0 | 0 | 0 | 0 | 0 | 0 | 0 |
| k__Bacteria;p__Proteobacteria;c__Epsilonproteobacteria;o__Campylobacterales;f__Helicobacteraceae;g__Wolinella | 0 | 0 | 0 | 0.00070595 | 0 | 0 | 0 | 0 | 0 | 0 | 0 | 0 | 0 | 0 | 0 |
| k__Bacteria;p__Proteobacteria;c__Gammaproteobacteria;o__;f__;g__ | 0.00166718 | 0.0030822 | 0.00270655 | 0 | 0.00332452 | 0.00028167 | 0.00212908 | 0 | 0 | 0 | 0.00046137 | 0.00231532 | 0.00025034 | 0 | 0.00037671 |
| k__Bacteria;p__Proteobacteria;c__Gammaproteobacteria;o__Aeromonadales;f__Aeromonadaceae;g__ | 1.33E-05 | 0 | 0 | 0 | 0 | 0 | 0 | 0 | 0 | 0 | 0 | 0 | 0 | 0 | 0 |
| k__Bacteria;p__Proteobacteria;c__Gammaproteobacteria;o__Aeromonadales;f__Aeromonadaceae;g__Aeromonas | 0 | 0 | 0 | 0 | 0 | 0 | 0 | 0 | 0 | 0 | 0 | 0 | 0 | 0 | 0 |
| k__Bacteria;p__Proteobacteria;c__Gammaproteobacteria;o__Aeromonadales;f__Aeromonadaceae;g__Tolumonas | 0 | 0 | 0 | 0.00016291 | 0 | 0 | 0 | 0 | 0 | 0 | 0 | 0 | 0 | 0 | 0 |
| k__Bacteria;p__Proteobacteria;c__Gammaproteobacteria;o__Alteromonadales;f__;g__ | 0 | 0 | 0 | 0 | 0 | 0.00052813 | 0.00025995 | 0 | 0 | 0 | 0.00014764 | 0.00324916 | 0 | 0 | 3.28E-05 |
| k__Bacteria;p__Proteobacteria;c__Gammaproteobacteria;o__Alteromonadales;f__Alteromonadaceae;g__ | 0.00098697 | 0.00379245 | 0.00207712 | 0.00494162 | 0.02012682 | 0.00533413 | 0.00250043 | 0 | 0 | 1.98E-05 | 0.00011073 | 6.17E-05 | 0.0001348 | 4.12E-05 | 0.05598231 |
| k__Bacteria;p__Proteobacteria;c__Gammaproteobacteria;o__Alteromonadales;f__Alteromonadaceae;g__Alteromonas | 0.00129373 | 0.00104527 | 5.04E-05 | 0 | 0.00037224 | 0.01221745 | 0.00138638 | 0 | 0 | 1.98E-05 | 0.000203 | 3.86E-05 | 0.00071251 | 0.00072042 | 0.01714847 |
| k__Bacteria;p__Proteobacteria;c__Gammaproteobacteria;o__Alteromonadales;f__Alteromonadaceae;g__BD2-13 | 0.00060018 | 0 | 0.00065461 | 0 | 0.00102688 | 0.00029927 | 0.00053227 | 0 | 0 | 0 | 0 | 0 | 0 | 0 | 0 |
| k__Bacteria;p__Proteobacteria;c__Gammaproteobacteria;o__Alteromonadales;f__Alteromonadaceae;g__Candidatus Endobugula | 0 | 0 | 0 | 0 | 0 | 0 | 0 | 0 | 0 | 0 | 0 | 0 | 0 | 0 | 0 |
| k__Bacteria;p__Proteobacteria;c__Gammaproteobacteria;o__Alteromonadales;f__Alteromonadaceae;g__Cellvibrio | 0 | 0 | 0 | 0 | 0 | 0 | 0 | 0 | 0 | 0 | 0 | 0 | 0 | 0 | 0 |
| k__Bacteria;p__Proteobacteria;c__Gammaproteobacteria;o__Alteromonadales;f__Alteromonadaceae;g__Glaciecola | 0.02292703 | 0.03398462 | 0.01965079 | 0.00108607 | 0.01540318 | 0.10872474 | 0.24088332 | 0 | 0 | 0.1304641 | 0.24820064 | 0.17824839 | 0.00338924 | 0.0035815 | 0.21537958 |
| k__Bacteria;p__Proteobacteria;c__Gammaproteobacteria;o__Alteromonadales;f__Alteromonadaceae;g__HTCC2207 | 0.02727503 | 0.00239876 | 0.00261843 | 0.00233505 | 0.01989577 | 0.00228857 | 0.000916 | 0 | 0.0148106 | 0.00696152 | 0.01096224 | 0.00663724 | 0.00146354 | 0.00090567 | 0.00055687 |
| k__Bacteria;p__Proteobacteria;c__Gammaproteobacteria;o__Alteromonadales;f__Alteromonadaceae;g__Marinobacter | 0.00054683 | 0.00115248 | 0.00119592 | 0.01096932 | 0.00364542 | 0.00227097 | 0.00123784 | 0 | 0 | 0 | 0 | 0 | 0.01831347 | 0 | 0.00759971 |
| k__Bacteria;p__Proteobacteria;c__Gammaproteobacteria;o__Alteromonadales;f__Alteromonadaceae;g__Porticoccus | 0 | 0 | 0 | 0 | 0 | 0 | 0 | 0 | 0 | 0 | 0 | 0 | 0 | 0 | 0 |
| k__Bacteria;p__Proteobacteria;c__Gammaproteobacteria;o__Alteromonadales;f__Alteromonadaceae;g__Spongiibacter | 0 | 0 | 0 | 0 | 0 | 0 | 0 | 0 | 0 | 0 | 0 | 0 | 0 | 0 | 0 |
| k__Bacteria;p__Proteobacteria;c__Gammaproteobacteria;o__Alteromonadales;f__Alteromonadaceae;g__ZD0117 | 0.00058685 | 0.00038863 | 0.00103226 | 0 | 0.00016687 | 0.0008098 | 0.00106454 | 0 | 0 | 0 | 0 | 0 | 0 | 0 | 0.00298092 |
| k__Bacteria;p__Proteobacteria;c__Gammaproteobacteria;o__Alteromonadales;f__Alteromonadaceae;g__nsmpVI18 | 0 | 0 | 0 | 0 | 5.13E-05 | 0 | 0 | 0 | 0 | 0 | 0 | 0 | 0 | 0 | 1.64E-05 |
| k__Bacteria;p__Proteobacteria;c__Gammaproteobacteria;o__Alteromonadales;f__Colwelliaceae;g__ | 0.1110874 | 0.08730669 | 0.06573763 | 0.00048873 | 0.09819526 | 0.02874798 | 0.04540391 | 0 | 0 | 0.02717176 | 0.02297641 | 0.07906801 | 0.02177974 | 0.02634666 | 0.03398575 |
| k__Bacteria;p__Proteobacteria;c__Gammaproteobacteria;o__Alteromonadales;f__Colwelliaceae;g__Colwellia | 9.34E-05 | 1.34E-05 | 0.00040283 | 0 | 0.00028239 | 0 | 0.00013616 | 0 | 0 | 0.00226101 | 5.54E-05 | 0.00189084 | 0 | 0 | 1.64E-05 |
| k__Bacteria;p__Proteobacteria;c__Gammaproteobacteria;o__Alteromonadales;f__Colwelliaceae;g__Thalassomonas | 0.00030676 | 0 | 0 | 0 | 1.28E-05 | 0 | 0 | 0 | 0 | 0 | 0 | 0 | 0 | 0 | 0 |
| k__Bacteria;p__Proteobacteria;c__Gammaproteobacteria;o__Alteromonadales;f__HTCC2188;g__ | 0 | 0 | 0 | 0 | 0 | 0.0004049 | 0 | 0 | 0 | 0 | 0 | 0 | 0 | 0 | 0 |

| Taxon | 1231 | 1233 | 1234 | 1235 | 1236 | 1243 | 1244 | 6473 | 6474 | 1188 | 1189 | 1190 | 1226 | 1227 | 2027 |
|---|---|---|---|---|---|---|---|---|---|---|---|---|---|---|---|
| Copepod genus | Calanus | Calanus | Calanus | Calanus | Calanus | Centropages | Centropages | Centropages | Centropages | Centropages | Centropages | Centropages | Centropages | Centropages | Centropages |
| Type of sample | FullGut | FullGut | FullGut | FullGut | FullGut | FullGut | FullGut | FullGut | FullGut | Starved | Starved | Starved | Starved | Starved | Starved |
| k__Bacteria;p__Proteobacteria;c__Gammaproteobacteria;o__Alteromonadales;f__HTCC2188;g__HTCC | 0.00016005 | 1.34E-05 | 0.00018883 | 0 | 0 | 0.00077459 | 0.00081697 | 0 | 0 | 0 | 1.85E-05 | 2.32E-05 | 0 | 0 | 0.00047498 |
| k__Bacteria;p__Proteobacteria;c__Gammaproteobacteria;o__Alteromonadales;f__Idiomarinaceae;g__Idiomarina | 0 | 0.00013401 | 0 | 0 | 0 | 0.00202451 | 0.00021043 | 0 | 0 | 0 | 0 | 0 | 0 | 0 | 0.00011465 |
| k__Bacteria;p__Proteobacteria;c__Gammaproteobacteria;o__Alteromonadales;f__Moritellaceae;g__Moritella | 0 | 0 | 0 | 0 | 0 | 0.00035209 | 0 | 0 | 0 | 0 | 0.00025837 | 0.00044763 | 0 | 0 | 0 |
| k__Bacteria;p__Proteobacteria;c__Gammaproteobacteria;o__Alteromonadales;f__OM60;g__ | 0.00093362 | 0.00163491 | 0.00084344 | 0.00190062 | 0.00115524 | 0.00338004 | 0.00094076 | 0 | 0 | 0.00170567 | 0.00151331 | 0.00080264 | 0 | 0 | 0.00093358 |
| k__Bacteria;p__Proteobacteria;c__Gammaproteobacteria;o__Alteromonadales;f__OM60;g__Congregibacter | 0 | 0 | 0 | 0 | 0 | 0 | 0 | 0 | 0 | 0 | 0 | 0 | 0 | 0 | 0 |
| k__Bacteria;p__Proteobacteria;c__Gammaproteobacteria;o__Alteromonadales;f__Psychromonadaceae;g__Psychromonas | 0 | 0 | 0.0005539 | 0 | 0 | 0 | 9.90E-05 | 0 | 0 | 0 | 0.00053519 | 0 | 0 | 0 | 0.00011465 |
| k__Bacteria;p__Proteobacteria;c__Gammaproteobacteria;o__Alteromonadales;f__Shewanellaceae;g__Shewanella | 0 | 0 | 0 | 0 | 0.00030806 | 0.00052813 | 3.71E-05 | 0 | 0 | 0.00162634 | 0.00018455 | 0.00290186 | 0 | 0 | 0.00027844 |
| k__Bacteria;p__Proteobacteria;c__Gammaproteobacteria;o__Alteromonadales;f__[Chromatiaceae];g__ | 0 | 0 | 0 | 0 | 0 | 0 | 0 | 0 | 0.00021361 | 0 | 0 | 0 | 0 | 0 | 0 |
| k__Bacteria;p__Proteobacteria;c__Gammaproteobacteria;o__Alteromonadales;f__[Chromatiaceae];g__Rheinheimera | 5.33E-05 | 0 | 0 | 0 | 0 | 0 | 0 | 0 | 0 | 0 | 0 | 0 | 0 | 0 | 0 |
| k__Bacteria;p__Proteobacteria;c__Gammaproteobacteria;o__Cardiobacteriales;f__Cardiobacteriaceae;g__Cardiobacterium | 0 | 0 | 0 | 0 | 0 | 0 | 0 | 0 | 0 | 0 | 0 | 0 | 0 | 0 | 0 |
| k__Bacteria;p__Proteobacteria;c__Gammaproteobacteria;o__Chromatiales;f__;g__ | 8.00E-05 | 0 | 0 | 0 | 0 | 0.00022886 | 0 | 0 | 0 | 0 | 0 | 0 | 0 | 0 | 0 |
| k__Bacteria;p__Proteobacteria;c__Gammaproteobacteria;o__Chromatiales;f__Chromatiaceae;g__ | 0 | 0 | 0 | 0 | 0 | 0 | 0 | 0 | 0 | 0 | 0 | 0 | 0 | 0 | 0 |
| k__Bacteria;p__Proteobacteria;c__Gammaproteobacteria;o__Enterobacteriales;f__Enterobacteriaceae;g__ | 0.00016005 | 0.00012061 | 0.00036507 | 0 | 0 | 0 | 0 | 0 | 7.12E-05 | 0 | 0 | 1.54E-05 | 0.00227233 | 0.00230533 | 0 |
| k__Bacteria;p__Proteobacteria;c__Gammaproteobacteria;o__Enterobacteriales;f__Enterobacteriaceae;g__Enterobacter | 0 | 0 | 0 | 0 | 0 | 0 | 0 | 0 | 0 | 0 | 0.00022146 | 0 | 0 | 8.23E-05 | 0 |
| k__Bacteria;p__Proteobacteria;c__Gammaproteobacteria;o__Enterobacteriales;f__Enterobacteriaceae;g__Erwinia | 0 | 0 | 0 | 0 | 0 | 0 | 0 | 0 | 0 | 0 | 0 | 0 | 0 | 0 | 1.64E-05 |
| k__Bacteria;p__Proteobacteria;c__Gammaproteobacteria;o__Enterobacteriales;f__Enterobacteriaceae;g__Escherichia | 0 | 0 | 0 | 0 | 0 | 0 | 0 | 0 | 0 | 0 | 0 | 0 | 1.93E-05 | 0 | 0 |
| k__Bacteria;p__Proteobacteria;c__Gammaproteobacteria;o__Enterobacteriales;f__Enterobacteriaceae;g__Ewingella | 0 | 0 | 0 | 0 | 0 | 0 | 0 | 0 | 0 | 0 | 0 | 0 | 0 | 0 | 0 |
| k__Bacteria;p__Proteobacteria;c__Gammaproteobacteria;o__Enterobacteriales;f__Enterobacteriaceae;g__Gluconacetobacter | 0 | 0 | 0 | 0 | 0 | 0 | 0 | 0 | 0 | 0 | 0 | 0.00013892 | 0 | 0 | 0 |
| k__Bacteria;p__Proteobacteria;c__Gammaproteobacteria;o__Enterobacteriales;f__Enterobacteriaceae;g__Klebsiella | 0 | 0 | 0 | 0 | 0 | 0 | 0 | 0 | 0 | 0 | 0 | 0 | 0 | 0 | 0 |
| k__Bacteria;p__Proteobacteria;c__Gammaproteobacteria;o__Enterobacteriales;f__Enterobacteriaceae;g__Pantoea | 0 | 0 | 0 | 0 | 0 | 0.00012323 | 0 | 0 | 0 | 0 | 0 | 0 | 0 | 0 | 0 |
| k__Bacteria;p__Proteobacteria;c__Gammaproteobacteria;o__Enterobacteriales;f__Enterobacteriaceae;g__Salmonella | 0 | 0 | 0 | 0 | 0 | 0 | 0 | 0 | 0 | 0 | 0 | 0 | 0 | 0 | 0 |
| k__Bacteria;p__Proteobacteria;c__Gammaproteobacteria;o__Enterobacteriales;f__Enterobacteriaceae;g__Serratia | 0 | 0 | 0 | 0 | 0 | 0 | 0 | 0.00103252 | 0 | 0 | 0 | 0 | 0 | 0 | 0 |
| k__Bacteria;p__Proteobacteria;c__Gammaproteobacteria;o__HTCC2188;f__HTCC2089;g__ | 0.00024007 | 0.00026802 | 0.00061684 | 0 | 0.00039792 | 0.00165481 | 0.00075508 | 0 | 0 | 0 | 0.00156867 | 0.00062514 | 0.000674 | 0 | 0.00039309 |
| k__Bacteria;p__Proteobacteria;c__Gammaproteobacteria;o__HTCC2188;f__HTCC2089;g__Acinetobacter | 0 | 0 | 0 | 0 | 0 | 0 | 0 | 0 | 0 | 0 | 0 | 0 | 0 | 0 | 0 |
| k__Bacteria;p__Proteobacteria;c__Gammaproteobacteria;o__Legionellales;f__;g__ | 0 | 0 | 0 | 0 | 0 | 0 | 0 | 0 | 0 | 0 | 0 | 0 | 0 | 0 | 0 |
| k__Bacteria;p__Proteobacteria;c__Gammaproteobacteria;o__Legionellales;f__Coxiellaceae;g__ | 0 | 0 | 0 | 0 | 0 | 0 | 0 | 0 | 0 | 0 | 0 | 0 | 0 | 0 | 0 |
| k__Bacteria;p__Proteobacteria;c__Gammaproteobacteria;o__Legionellales;f__Francisellaceae;g__ | 0 | 0 | 0 | 0 | 0 | 0 | 0 | 0 | 0 | 0 | 0 | 0 | 0 | 0 | 0 |
| k__Bacteria;p__Proteobacteria;c__Gammaproteobacteria;o__Legionellales;f__Legionellaceae;g__ | 2.67E-05 | 0 | 2.52E-05 | 0 | 6.42E-05 | 0 | 3.71E-05 | 0 | 0 | 0 | 0 | 0 | 0 | 0 | 0 |
| k__Bacteria;p__Proteobacteria;c__Gammaproteobacteria;o__Legionellales;f__Legionellaceae;g__Legionella | 0 | 0 | 0 | 0 | 0 | 0 | 0 | 0 | 0 | 0 | 0 | 0 | 0 | 0 | 0 |
| k__Bacteria;p__Proteobacteria;c__Gammaproteobacteria;o__Methylococcales;f__;g__ | 0 | 0 | 0 | 0 | 0 | 0 | 0.0001733 | 0 | 0 | 0 | 0 | 0 | 0 | 0 | 0 |
| k__Bacteria;p__Proteobacteria;c__Gammaproteobacteria;o__Oceanospirillales;f__;g__ | 0.00030676 | 0.00044223 | 0 | 0 | 2.57E-05 | 0.0015844 | 9.90E-05 | 0 | 0.02420963 | 0 | 0.00121803 | 0.00062514 | 0 | 0 | 0.00026206 |
| k__Bacteria;p__Proteobacteria;c__Gammaproteobacteria;o__Oceanospirillales;f__Alcanivoracaceae;g__Alcanivorax | 0 | 0 | 0 | 0 | 6.42E-05 | 0 | 0 | 0 | 0 | 0 | 0 | 0 | 0.00082805 | 0 | 0.00016379 |
| k__Bacteria;p__Proteobacteria;c__Gammaproteobacteria;o__Oceanospirillales;f__Endozoicimonaceae;g__Endozoicomonas | 0 | 0 | 0 | 0 | 0 | 0 | 0 | 0 | 0 | 0 | 0 | 0 | 0 | 0 | 0 |
| k__Bacteria;p__Proteobacteria;c__Gammaproteobacteria;o__Oceanospirillales;f__Halomonadaceae;g__ | 0.00748229 | 0.00560157 | 0.01406146 | 0.00108607 | 0.0025672 | 0.00059855 | 0.00038373 | 0 | 0 | 0 | 0 | 0 | 0 | 0 | 0.0165916 |
| k__Bacteria;p__Proteobacteria;c__Gammaproteobacteria;o__Oceanospirillales;f__Halomonadaceae;g__Candidatus Portiera | 0.01070995 | 0.01277103 | 0.00944144 | 0.00048873 | 0.00962699 | 0.01297444 | 0.01572055 | 0 | 0.02584734 | 0.00652519 | 0.04185583 | 0.00825024 | 0.00242639 | 0.00312867 | 0.00771436 |
| k__Bacteria;p__Proteobacteria;c__Gammaproteobacteria;o__Oceanospirillales;f__Halomonadaceae;g__Cobetia | 0 | 0 | 0 | 0 | 0 | 0 | 0 | 0 | 0 | 0 | 0 | 0 | 0 | 0 | 1.64E-05 |
| k__Bacteria;p__Proteobacteria;c__Gammaproteobacteria;o__Oceanospirillales;f__Halomonadaceae;g__Haererehalobacter | 0.00184056 | 0.00029482 | 0.00064202 | 0 | 0 | 0 | 0 | 0 | 0 | 0 | 0 | 0 | 0 | 0 | 0 |
| k__Bacteria;p__Proteobacteria;c__Gammaproteobacteria;o__Oceanospirillales;f__Halomonadaceae;g__Halomonas | 0.00054683 | 0.00062984 | 0.00071755 | 0 | 0.00039792 | 0.00293993 | 3.71E-05 | 0 | 0 | 0 | 0 | 0 | 0.00109765 | 0 | 0.00158873 |
| k__Bacteria;p__Proteobacteria;c__Gammaproteobacteria;o__Oceanospirillales;f__Oceanospirillaceae;g__ | 0.00037345 | 0.00143389 | 0.00164911 | 0 | 0.00037224 | 0.00012323 | 0.00035897 | 0 | 0 | 0.00678302 | 0.00941203 | 0.00995585 | 0 | 0.00168783 | 0.00018017 |
| k__Bacteria;p__Proteobacteria;c__Gammaproteobacteria;o__Oceanospirillales;f__Oceanospirillaceae;g__Amphritea | 0 | 0 | 0 | 0 | 0 | 0 | 0 | 0 | 0 | 0 | 0 | 2.32E-05 | 0 | 0 | 1.64E-05 |
| k__Bacteria;p__Proteobacteria;c__Gammaproteobacteria;o__Oceanospirillales;f__Oceanospirillaceae;g__Marinobacterium | 0 | 0 | 0 | 0 | 0 | 0 | 0 | 0 | 0 | 0 | 0 | 0 | 0 | 0 | 0 |
| k__Bacteria;p__Proteobacteria;c__Gammaproteobacteria;o__Oceanospirillales;f__Oceanospirillaceae;g__Marinomonas | 0.00973632 | 0.01713972 | 0.01626446 | 5.43E-05 | 0.03950915 | 0.00137314 | 0.03353304 | 0 | 0 | 0.02742959 | 0.07009191 | 0.03002964 | 0.01993106 | 0.00829508 | 0.03788388 |
| k__Bacteria;p__Proteobacteria;c__Gammaproteobacteria;o__Oceanospirillales;f__Oceanospirillaceae;g__Neptunomonas | 0 | 0 | 0 | 0 | 0 | 0 | 0 | 0 | 0 | 0 | 0 | 0 | 0 | 0 | 0 |
| k__Bacteria;p__Proteobacteria;c__Gammaproteobacteria;o__Oceanospirillales;f__Oceanospirillaceae;g__Oceaniserpentilla | 0 | 0 | 0 | 0 | 0.00015403 | 0 | 0.00014854 | 0 | 0 | 0.00247917 | 0.00012918 | 0.0003936 | 0 | 0 | 0 |
| k__Bacteria;p__Proteobacteria;c__Gammaproteobacteria;o__Oceanospirillales;f__Oceanospirillaceae;g__Oleibacter | 0.05680409 | 0.06892069 | 0.04873044 | 0 | 0.0565425 | 0.025632 | 0.02437303 | 0 | 0 | 0 | 0 | 0.00025468 | 0.02678657 | 0.04019925 | 0.00260421 |
| k__Bacteria;p__Proteobacteria;c__Gammaproteobacteria;o__Oceanospirillales;f__Oceanospirillaceae;g__Oleispira | 0.00378783 | 0.00134009 | 0.00096932 | 0 | 0.00059046 | 0.00059855 | 0.00199292 | 0 | 0 | 0.00829036 | 0.00941203 | 0.04492483 | 0.00134799 | 0.00675133 | 6.55E-05 |
| k__Bacteria;p__Proteobacteria;c__Gammaproteobacteria;o__Oceanospirillales;f__Oceanospirillaceae;g__Spongiispira | 0 | 0 | 0 | 0 | 0 | 0 | 0 | 0 | 0 | 0 | 0 | 0 | 0 | 0 | 0 |
| k__Bacteria;p__Proteobacteria;c__Gammaproteobacteria;o__Oceanospirillales;f__Oleiphilaceae;g__ | 0.00086693 | 0 | 0.00028954 | 0 | 0.00057762 | 0.00063376 | 0.0012007 | 0 | 0 | 0 | 0 | 0 | 0 | 0 | 9.83E-05 |
| k__Bacteria;p__Proteobacteria;c__Gammaproteobacteria;o__Oceanospirillales;f__SUP05;g__ | 0 | 0.00058964 | 0 | 0 | 0 | 0 | 0 | 0 | 0 | 0.00146767 | 0.00442919 | 0.00081036 | 0.00109765 | 0 | 0 |
| k__Bacteria;p__Proteobacteria;c__Gammaproteobacteria;o__Oceanospirillales;f__Saccharospirillaceae;g__Reinekea | 0 | 0 | 0 | 0 | 0 | 7.04E-05 | 0 | 0 | 0 | 0 | 0 | 0 | 0 | 0 | 0 |
| k__Bacteria;p__Proteobacteria;c__Gammaproteobacteria;o__Oceanospirillales;f__Saccharospirillaceae;g__Saccharospirillum | 0 | 0 | 0 | 0 | 0 | 0 | 0 | 0 | 0 | 0 | 0 | 0 | 0 | 0 | 1.64E-05 |
| k__Bacteria;p__Proteobacteria;c__Gammaproteobacteria;o__Pasteurellales;f__Pasteurellaceae;g__Actinobacillus | 4.00E-05 | 0 | 0 | 0 | 0 | 0 | 0 | 0 | 0 | 0 | 0 | 0 | 0 | 0 | 0 |
| k__Bacteria;p__Proteobacteria;c__Gammaproteobacteria;o__Pasteurellales;f__Pasteurellaceae;g__Aggregatibacter | 0 | 0 | 0.00017624 | 0 | 0 | 0 | 0 | 0 | 0 | 0 | 9.23E-05 | 0 | 0 | 0 | 0 |
| k__Bacteria;p__Proteobacteria;c__Gammaproteobacteria;o__Pasteurellales;f__Pasteurellaceae;g__Haemophilus | 0 | 0.00018761 | 0 | 0 | 0 | 8.80E-05 | 4.95E-05 | 0 | 0.00712048 | 0 | 0.00092275 | 0 | 0 | 0.00028817 | 1.64E-05 |
| k__Bacteria;p__Proteobacteria;c__Gammaproteobacteria;o__Pseudomonadales;f__Moraxellaceae;g__ | 0.01428438 | 0.00695505 | 0.02020469 | 0.00021721 | 0.00953713 | 0.0152102 | 0.0020053 | 0.00206505 | 0.00028482 | 3.97E-05 | 0 | 7.72E-06 | 0.11767991 | 0.06531091 | 0.01279175 |
| k__Bacteria;p__Proteobacteria;c__Gammaproteobacteria;o__Pseudomonadales;f__Moraxellaceae;g__Acinetobacter | 0.00116036 | 0.00041543 | 0.00049096 | 0 | 6.42E-05 | 5.28E-05 | 0 | 0.00258131 | 0.0252777 | 0 | 0.00018455 | 0 | 0 | 0 | 0 |
| k__Bacteria;p__Proteobacteria;c__Gammaproteobacteria;o__Pseudomonadales;f__Moraxellaceae;g__Alkanindiges | 0 | 0 | 0.00027695 | 0 | 0 | 0 | 0 | 0 | 0 | 0 | 0 | 0 | 0 | 0 | 0 |
| k__Bacteria;p__Proteobacteria;c__Gammaproteobacteria;o__Pseudomonadales;f__Moraxellaceae;g__Enhydrobacter | 0 | 0 | 0 | 0.00363834 | 0 | 0 | 0 | 0 | 0 | 0 | 0 | 0 | 0 | 0 | 0 |
| k__Bacteria;p__Proteobacteria;c__Gammaproteobacteria;o__Pseudomonadales;f__Moraxellaceae;g__Perlucidibaca | 0 | 0 | 0 | 0 | 0 | 0 | 0 | 0 | 0 | 0 | 0 | 0 | 0 | 0 | 0 |
| k__Bacteria;p__Proteobacteria;c__Gammaproteobacteria;o__Pseudomonadales;f__Moraxellaceae;g__Psychrobacter | 0.00664204 | 0.0015947 | 0.03145889 | 0 | 0.00726517 | 0.00820365 | 0.00199292 | 0 | 0 | 0 | 0 | 0 | 0.01132315 | 0.01529342 | 0.00398002 |
| k__Bacteria;p__Proteobacteria;c__Gammaproteobacteria;o__Pseudomonadales;f__Pseudomonadaceae;g__ | 0.00020006 | 0.00025462 | 0.0004406 | 0.11876188 | 0 | 0.00028167 | 0 | 0 | 0 | 0 | 0.00014764 | 0 | 0.00155982 | 0.00090567 | 0 |
| k__Bacteria;p__Proteobacteria;c__Gammaproteobacteria;o__Pseudomonadales;f__Pseudomonadaceae;g__Pseudomonas | 0.00133374 | 0.00188952 | 0.00214006 | 0.46532718 | 1.28E-05 | 0.00096824 | 8.66E-05 | 0.1997935 | 0.04927371 | 0.00515668 | 0.00023991 | 0.00107276 | 0.00311964 | 0.01833975 | 0.00019654 |
| k__Bacteria;p__Proteobacteria;c__Gammaproteobacteria;o__Salinisphaerales;f__Salinisphaeraceae;g__Salinisphaera | 0 | 0 | 0 | 0 | 0 | 0 | 0 | 0 | 0 | 0 | 0 | 0 | 0 | 0 | 0 |
| k__Bacteria;p__Proteobacteria;c__Gammaproteobacteria;o__Thiohalorhabdales;f__;g__ | 0 | 0 | 0 | 0 | 0 | 0 | 0 | 0 | 0 | 0 | 0 | 0 | 0 | 0 | 0 |
| k__Bacteria;p__Proteobacteria;c__Gammaproteobacteria;o__Thiohalorhabdales;f__Thiohalorhabdaceae;g__ | 0 | 0 | 0 | 0 | 0 | 0 | 2.48E-05 | 0 | 0 | 0 | 0 | 0 | 0 | 0 | 0 |
| k__Bacteria;p__Proteobacteria;c__Gammaproteobacteria;o__Thiotrichales;f__Piscirickettsiaceae;g__ | 0.07746376 | 0.00167511 | 0.03463122 | 0.00809123 | 0.03903422 | 0.00014084 | 0 | 0 | 0.00384506 | 0 | 0.00108884 | 0.00070231 | 0.00046217 | 0 | 0.00029482 |
| k__Bacteria;p__Proteobacteria;c__Gammaproteobacteria;o__Thiotrichales;f__Piscirickettsiaceae;g__Methylophaga | 0 | 0 | 0 | 0.00097746 | 8.99E-05 | 0 | 0 | 0 | 0 | 0 | 0 | 0 | 0 | 0 | 6.55E-05 |
| k__Bacteria;p__Proteobacteria;c__Gammaproteobacteria;o__Thiotrichales;f__Thiotrichaceae;g__ | 0 | 0 | 0 | 0 | 0 | 0 | 0 | 0 | 0 | 0 | 0 | 0 | 0 | 0 | 0 |
| k__Bacteria;p__Proteobacteria;c__Gammaproteobacteria;o__Thiotrichales;f__Thiotrichaceae;g__Cocleimonas | 0 | 0 | 0 | 0 | 0 | 0 | 0 | 0 | 0 | 0 | 0 | 0 | 0 | 0 | 0 |
| k__Bacteria;p__Proteobacteria;c__Gammaproteobacteria;o__Thiotrichales;f__Thiotrichaceae;g__E8 | 0 | 0 | 0 | 0 | 0 | 0.00100345 | 0 | 0 | 0 | 0 | 0 | 0 | 0 | 0 | 0 |
| k__Bacteria;p__Proteobacteria;c__Gammaproteobacteria;o__Thiotrichales;f__Thiotrichaceae;g__Leucothrix | 0 | 0 | 0 | 0 | 0.00016687 | 0 | 0 | 0 | 0 | 0 | 0 | 0 | 0.00046217 | 0 | 0 |
| k__Bacteria;p__Proteobacteria;c__Gammaproteobacteria;o__Thiotrichales;f__Thiotrichaceae;g__Thiothrix | 0 | 0 | 0 | 0 | 0 | 0 | 0 | 0 | 0 | 0 | 0 | 0 | 0 | 0 | 4.91E-05 |
| k__Bacteria;p__Proteobacteria;c__Gammaproteobacteria;o__Vibrionales;f__;g__ | 0.01556477 | 0.02035593 | 0.01781286 | 0.00119468 | 0.10850255 | 0.00269347 | 0.01961974 | 0 | 0 | 0.13288378 | 0.03602407 | 0.02323805 | 0.00015406 | 0.006916 | 0.00420932 |
| k__Bacteria;p__Proteobacteria;c__Gammaproteobacteria;o__Vibrionales;f__Pseudoalteromonadaceae;g__Pseudoalteromonas | 0.3677261 | 0.52241966 | 0.55362111 | 0.01151235 | 0.20958591 | 0.11960425 | 0.20800634 | 0 | 7.12E-05 | 0.15228084 | 0.09114901 | 0.36126632 | 0.5312831 | 0.4287508 | 0.25174023 |

| Taxon | 1231 | 1233 | 1234 | 1235 | 1236 | 1243 | 1244 | 6473 | 6474 | 1188 | 1189 | 1190 | 1226 | 1227 | 2027 |
|---|---|---|---|---|---|---|---|---|---|---|---|---|---|---|---|
| Copepod genus | Calanus | Calanus | Calanus | Calanus | Calanus | Centropages | Centropages | Centropages | Centropages | Centropages | Centropages | Centropages | Centropages | Centropages | Centropages |
| Type of sample | FullGut | FullGut | FullGut | FullGut | FullGut | FullGut | FullGut | FullGut | FullGut | Starved | Starved | Starved | Starved | Starved | Starved |
| k__Bacteria;p__Proteobacteria;c__Gammaproteobacteria;o__Vibrionales;f__Vibrionaceae;g__ | 0.0091228 | 0.01405752 | 0.01402369 | 0.00076025 | 0.03542731 | 0.00153158 | 0.00982844 | 0 | 0 | 0.10989687 | 0.01280774 | 0.01563609 | 0.00063548 | 0.00384908 | 0.00235853 |
| k__Bacteria;p__Proteobacteria;c__Gammaproteobacteria;o__Vibrionales;f__Vibrionaceae;g__Aliivibrio | 0.00309428 | 0.00337702 | 0.0025429 | 0 | 0.08046877 | 0.00056334 | 0.00248805 | 0 | 0.00220735 | 0.01771123 | 0.00420773 | 0.00355787 | 0.0008088 | 0.00203775 | 0.0006879 |
| k__Bacteria;p__Proteobacteria;c__Gammaproteobacteria;o__Vibrionales;f__Vibrionaceae;g__Enterovibrio | 0 | 0 | 0 | 0 | 0 | 0 | 0.00013616 | 0 | 0 | 0 | 0 | 0 | 0 | 0 | 4.91E-05 |
| k__Bacteria;p__Proteobacteria;c__Gammaproteobacteria;o__Vibrionales;f__Vibrionaceae;g__Listonella | 8.00E-05 | 9.38E-05 | 1.26E-05 | 0 | 0.00033374 | 0 | 6.19E-05 | 0 | 0 | 0.00037683 | 0.00011073 | 7.72E-05 | 0 | 2.06E-05 | 0 |
| k__Bacteria;p__Proteobacteria;c__Gammaproteobacteria;o__Vibrionales;f__Vibrionaceae;g__Photobacterium | 0.0066287 | 0 | 0.00245478 | 0.00168341 | 0.00015403 | 0.00021125 | 0.00034659 | 0 | 0 | 0 | 0.00083047 | 0.00047078 | 0.0165033 | 0.03015458 | 0.00021292 |
| k__Bacteria;p__Proteobacteria;c__Gammaproteobacteria;o__Vibrionales;f__Vibrionaceae;g__Vibrio | 0.00033344 | 0 | 0.00027695 | 0 | 0.00198958 | 0.0004049 | 0 | 0 | 0 | 0.00075367 | 0 | 7.72E-06 | 1.93E-05 | 0 | 0 |
| k__Bacteria;p__Proteobacteria;c__Gammaproteobacteria;o__Xanthomonadales;f__Sinobacteraceae;g__ | 0 | 6.70E-05 | 0 | 0.00059734 | 0 | 0 | 0 | 0.00258131 | 0.00078325 | 7.93E-05 | 3.69E-05 | 1.54E-05 | 3.85E-05 | 4.12E-05 | 0 |
| k__Bacteria;p__Proteobacteria;c__Gammaproteobacteria;o__Xanthomonadales;f__Xanthomonadaceae;g__ | 0.00017339 | 0.00013401 | 0.0003273 | 0.00032582 | 0 | 0.00022886 | 0.00011141 | 0 | 0 | 1.98E-05 | 9.23E-05 | 0 | 0.00123245 | 0.00115267 | 1.64E-05 |
| k__Bacteria;p__Proteobacteria;c__Gammaproteobacteria;o__Xanthomonadales;f__Xanthomonadaceae;g__Aspromonas | 0 | 0 | 0 | 0 | 0 | 0 | 0 | 0 | 0 | 0 | 0 | 0 | 0 | 0 | 0 |
| k__Bacteria;p__Proteobacteria;c__Gammaproteobacteria;o__Xanthomonadales;f__Xanthomonadaceae;g__Ignatzschineria | 0 | 0 | 0 | 0 | 0 | 0 | 0 | 0 | 0 | 0 | 0 | 0 | 0 | 0 | 0 |
| k__Bacteria;p__Proteobacteria;c__Gammaproteobacteria;o__Xanthomonadales;f__Xanthomonadaceae;g__Lysobacter | 0 | 0 | 0 | 0 | 0 | 0 | 0 | 0 | 0 | 0 | 0 | 0 | 0 | 0 | 0 |
| k__Bacteria;p__Proteobacteria;c__Gammaproteobacteria;o__Xanthomonadales;f__Xanthomonadaceae;g__Pseudoxanthomonas | 0 | 0 | 0 | 0 | 0 | 0 | 0 | 0 | 0 | 0 | 0 | 0 | 0 | 0 | 0 |
| k__Bacteria;p__Proteobacteria;c__Gammaproteobacteria;o__Xanthomonadales;f__Xanthomonadaceae;g__Stenotrophomonas | 0.00018672 | 0.00057624 | 0.00090638 | 0 | 0 | 0.00042251 | 1.24E-05 | 0 | 0 | 0.00075367 | 0.00027682 | 0 | 0.00036588 | 0.00047342 | 0 |
| k__Bacteria;p__Proteobacteria;c__Gammaproteobacteria;o__Xanthomonadales;f__Xanthomonadaceae;g__Thermomonas | 0 | 9.38E-05 | 0 | 0 | 0 | 0 | 0 | 0 | 0 | 0 | 0 | 0 | 0 | 0 | 0 |
| k__Bacteria;p__Proteobacteria;c__Gammaproteobacteria;o__[Marinicellales];f__[Marinicellaceae];g__ | 0 | 0 | 0 | 0 | 0 | 0 | 0 | 0 | 0 | 0 | 0 | 0 | 0 | 0 | 0 |
| k__Bacteria;p__Proteobacteria;c__Gammaproteobacteria;o__[Marinicellales];f__[Marinicellaceae];g__Marinicella | 0 | 0 | 0 | 0 | 0 | 0 | 0 | 0 | 0 | 0 | 0 | 0 | 0 | 0 | 0 |
| k__Bacteria;p__Proteobacteria;c__TA18;o__CV90;f__;g__ | 0 | 0 | 0 | 0 | 0 | 0 | 0 | 0 | 0 | 0 | 0 | 0 | 0 | 0 | 0 |
| k__Bacteria;p__Proteobacteria;c__Zetaproteobacteria;o__Mariprofundales;f__Mariprofundaceae;g__Mariprofundus | 0 | 0 | 0 | 0 | 0 | 0 | 0 | 0 | 0 | 0 | 0 | 0 | 0 | 0 | 0 |
| k__Bacteria;p__SAR406;c__AB16;o__Arctic96B-7;f__A714017;g__SGSH944 | 0 | 0 | 0 | 0 | 0 | 0 | 0 | 0 | 0 | 0.00055534 | 0 | 0 | 0 | 0 | 0 |
| k__Bacteria;p__SAR406;c__AB16;o__Arctic96B-7;f__A714017;g__SargSea-WGS | 0 | 0 | 0 | 0 | 0 | 0 | 0 | 0 | 0 | 0 | 0 | 0 | 0 | 0 | 0 |
| k__Bacteria;p__SAR406;c__AB16;o__Arctic96B-7;f__A714017;g__ZA3312c | 0 | 0.00010721 | 0.00013847 | 0 | 0 | 0 | 0 | 0 | 0 | 0 | 0.00042446 | 0 | 0 | 0 | 0 |
| k__Bacteria;p__SAR406;c__AB16;o__ZA3648c;f__AEGEAN_185;g__ | 0 | 0 | 0 | 0 | 0 | 0 | 0 | 0 | 0 | 0 | 0 | 0 | 0 | 0 | 0 |
| k__Bacteria;p__SBR1093;c__A712011;o__;f__;g__ | 0 | 0 | 0 | 0 | 1.28E-05 | 0 | 0 | 0 | 0 | 0 | 0 | 0 | 0 | 0 | 0 |
| k__Bacteria;p__SR1;c__;o__;f__;g__ | 0 | 0 | 0 | 0 | 0 | 0 | 0 | 0 | 0 | 0 | 0 | 0 | 0 | 0 | 0 |
| k__Bacteria;p__Spirochaetes;c__Spirochaetes;o__Sphaerochaetales;f__Sphaerochaetaceae;g__wall-less | 0 | 0 | 0 | 0 | 0 | 0 | 0 | 0 | 0 | 0 | 0 | 0 | 0 | 0 | 0 |
| k__Bacteria;p__Spirochaetes;c__Spirochaetes;o__Spirochaetales;f__Spirochaetaceae;g__Spirochaeta | 0 | 0 | 0 | 0 | 0 | 0.00012323 | 0 | 0 | 0 | 0 | 0 | 0 | 0 | 0 | 0 |
| k__Bacteria;p__Spirochaetes;c__Spirochaetes;o__Spirochaetales;f__Spirochaetaceae;g__Treponema | 0 | 0 | 0 | 0 | 8.99E-05 | 0 | 0 | 0 | 0 | 0 | 0 | 0 | 0 | 0 | 0 |
| k__Bacteria;p__TM6;c__SJA-4;o__;f__;g__ | 0 | 0 | 0 | 0 | 0 | 0 | 0 | 0 | 0 | 0 | 0 | 0 | 0 | 0 | 0 |
| k__Bacteria;p__TM7;c__TM7-1;o__;f__;g__ | 0 | 0 | 0 | 0 | 0 | 0 | 0 | 0 | 0 | 0 | 0 | 0 | 0 | 0 | 0 |
| k__Bacteria;p__TM7;c__TM7-3;o__;f__;g__ | 0 | 0 | 0 | 0.00076025 | 0 | 0 | 0 | 0 | 0 | 0 | 0 | 0 | 0 | 0 | 0 |
| k__Bacteria;p__TM7;c__TM7-3;o__CW040;f__F16;g__ | 0 | 4.02E-05 | 0 | 0 | 0 | 0 | 0 | 0 | 0 | 0 | 0 | 0 | 0 | 0 | 0 |
| k__Bacteria;p__TM7;c__TM7-3;o__EW055;f__;g__ | 0 | 0 | 0 | 0 | 0 | 0 | 0 | 0 | 0 | 0 | 0 | 0 | 0 | 0 | 0 |
| k__Bacteria;p__TM7;c__TM7-3;o__I025;f__Rs-045;g__ | 0 | 0 | 0 | 0 | 0 | 0 | 0 | 0 | 0 | 0 | 0 | 0 | 0 | 0 | 0 |
| k__Bacteria;p__Tenericutes;c__Mollicutes;o__Acholeplasmatales;f__Acholeplasmataceae;g__Acholeplasma | 0 | 0 | 0 | 0 | 0 | 0 | 0 | 0 | 0 | 0 | 0 | 0 | 0 | 0 | 0 |
| k__Bacteria;p__Tenericutes;c__Mollicutes;o__Mycoplasmatales;f__Mycoplasmataceae;g__Mycoplasma | 0 | 0 | 0 | 0 | 0 | 0 | 0 | 0 | 0 | 0 | 0 | 0 | 0 | 0 | 0 |
| k__Bacteria;p__Thermotogae;c__Thermotogae;o__Thermotogales;f__Thermotogaceae;g__Fervidobacterium | 0 | 0 | 0 | 0.00032582 | 0 | 0 | 0 | 0 | 0 | 0 | 0 | 0 | 0 | 0 | 0 |
| k__Bacteria;p__Verrucomicrobia;c__Opitutae;o__Opitutales;f__Opitutaceae;g__Opitutus | 0 | 0 | 0 | 0 | 0 | 0.00010563 | 0 | 0 | 0 | 0 | 0 | 0 | 0 | 0 | 0 |
| k__Bacteria;p__Verrucomicrobia;c__Opitutae;o__Puniceicoccales;f__Puniceicoccaceae;g__Coraliomargarita | 0.00014671 | 0.00025462 | 0 | 0 | 2.57E-05 | 0 | 0 | 0 | 0 | 0 | 0 | 0 | 0 | 0 | 0.00011465 |
| k__Bacteria;p__Verrucomicrobia;c__Opitutae;o__Puniceicoccales;f__Puniceicoccaceae;g__MB11C04 | 0.0014271 | 0.0014741 | 0.0001133 | 0 | 0.00047493 | 0.00140835 | 0.00043324 | 0 | 0 | 0 | 0.00029528 | 0 | 0 | 0 | 0.00019654 |
| k__Bacteria;p__Verrucomicrobia;c__Verrucomicrobiae;o__Verrucomicrobiales;f__Verrucomicrobiaceae;g__ | 0.00012004 | 0 | 0 | 0 | 0.00025672 | 0.00021125 | 7.43E-05 | 0 | 0 | 0 | 0 | 0 | 0 | 0 | 0.00131029 |
| k__Bacteria;p__Verrucomicrobia;c__Verrucomicrobiae;o__Verrucomicrobiales;f__Verrucomicrobiaceae;g__Akkermansia | 0 | 0 | 0 | 0 | 0 | 0 | 0 | 0 | 0 | 0 | 0 | 0 | 0 | 0 | 0 |
| k__Bacteria;p__Verrucomicrobia;c__Verrucomicrobiae;o__Verrucomicrobiales;f__Verrucomicrobiaceae;g__Luteolibacter | 0 | 0 | 0 | 0 | 0 | 0 | 0 | 0 | 0 | 0 | 0 | 0 | 0 | 0 | 1.64E-05 |
| k__Bacteria;p__Verrucomicrobia;c__Verrucomicrobiae;o__Verrucomicrobiales;f__Verrucomicrobiaceae;g__MSBL3 | 0 | 0 | 0 | 0 | 0 | 0 | 0 | 0 | 0 | 0 | 0 | 0 | 0 | 0 | 0 |
| k__Bacteria;p__Verrucomicrobia;c__Verrucomicrobiae;o__Verrucomicrobiales;f__Verrucomicrobiaceae;g__Persicirhabdus | 0.0039212 | 0.00180912 | 0 | 0 | 0.00105255 | 0.00253503 | 0.00049514 | 0 | 0 | 0 | 0 | 0 | 0 | 0.00131733 | 0.00026206 |
| k__Bacteria;p__Verrucomicrobia;c__Verrucomicrobiae;o__Verrucomicrobiales;f__Verrucomicrobiaceae;g__Prosthecobacter | 0 | 0 | 0 | 0 | 0 | 0 | 0 | 0 | 0 | 0 | 0 | 0 | 0 | 0 | 0 |
| k__Bacteria;p__Verrucomicrobia;c__Verrucomicrobiae;o__Verrucomicrobiales;f__Verrucomicrobiaceae;g__Rubritalea | 0 | 0 | 0 | 0 | 0 | 0.00026407 | 0 | 0 | 0 | 0.00152717 | 0 | 6.95E-05 | 0 | 0 | 0 |
| k__Bacteria;p__Verrucomicrobia;c__Verrucomicrobiae;o__Verrucomicrobiales;f__Verrucomicrobiaceae;g__Verrucomicrobium | 0.00065353 | 0.00131329 | 0 | 0 | 0.00051344 | 0.00172523 | 0.00021043 | 0 | 0 | 0 | 0 | 0 | 0 | 0 | 0.00067153 |
| k__Bacteria;p__Verrucomicrobia;c__[Spartobacteria];o__[Chthoniobacterales];f__[Chthoniobacteraceae];g__ | 0 | 0 | 0 | 0 | 0 | 0 | 0 | 0 | 0 | 0 | 0 | 0 | 0 | 0 | 0 |
| k__Bacteria;p__Verrucomicrobia;c__[Spartobacteria];o__[Chthoniobacterales];f__[Chthoniobacteraceae];g__Candidatus Xiphinematobacter | 0 | 0 | 0 | 0 | 0 | 0 | 0 | 0 | 0 | 0 | 0 | 0 | 0 | 0 | 0 |
| k__Bacteria;p__Verrucomicrobia;c__[Spartobacteria];o__[Chthoniobacterales];f__[Chthoniobacteraceae];g__DA101 | 0 | 0 | 0.0001133 | 0 | 0 | 0 | 0 | 0 | 0 | 0 | 0 | 0 | 0 | 0 | 0 |
| k__Bacteria;p__Verrucomicrobia;c__[Spartobacteria];o__[Chthoniobacterales];f__[Chthoniobacteraceae];g__Ellin506 | 0 | 0 | 0 | 0 | 0 | 0 | 0 | 0 | 0 | 0 | 0 | 0 | 0 | 0 | 0 |
| k__Bacteria;p__WS3;c__PRR-12;o__GN03;f__KSB4;g__ | 0 | 0 | 0 | 0 | 0 | 0 | 0 | 0 | 0 | 0 | 0 | 0 | 0 | 0 | 0 |
| k__Bacteria;p__WS3;c__PRR-12;o__Sediment-1;f__;g__ | 0 | 0 | 0 | 0 | 0 | 0 | 0 | 0 | 0 | 0 | 0 | 0 | 0 | 0 | 0 |
| k__Bacteria;p__ZB3;c__BS119;o__;f__;g__ | 0 | 0 | 0 | 0 | 0 | 0 | 0 | 0 | 0 | 0 | 0 | 0 | 0 | 0 | 0 |
| k__Bacteria;p__[Thermi];c__Deinococci;o__Deinococcales;f__Deinococcaceae;g__Deinococcus | 0 | 0 | 0 | 0 | 0 | 0 | 0 | 0 | 0 | 0 | 0 | 0 | 0 | 0 | 0 |
| k__Bacteria;p__[Thermi];c__Deinococci;o__Deinococcales;f__Trueperaceae;g__Truepera | 0 | 0 | 0 | 0 | 0 | 0 | 0 | 0 | 0.00477072 | 0.00119 | 0 | 0 | 0 | 0 | 0 |
| k__Bacteria;p__[Thermi];c__Deinococci;o__Thermales;f__Thermaceae;g__Thermus | 0 | 0 | 0 | 0 | 0 | 0 | 0 | 0 | 0 | 0 | 0 | 0 | 0 | 0 | 0 |

S3 Table. Genus-level sequence data shown as proportions of all sequences.

| Taxon | | 2028 | 2029 | 1184 | 1196 | 1245 | 1246 | 2019 |
|---|---|---|---|---|---|---|---|---|
| | Copepod genus | Centropages | Centropages | Water | Water | Water | Water | Water |
| | Type of sample | Starved | Starved | Water | Water | Water | Water | Water |
| k__Archaea;p__Crenarchaeota;c__Thaumarchaeota;o__Cenarchaeales;f__Cenarchaeaceae;g__Nitrosopumilus | | 0 | 0 | 6.77E-05 | 2.95E-05 | 0 | 0 | 0 |
| k__Archaea;p__Euryarchaeota;c__Thermoplasmata;o__E2;f__Marine group II;g__ | | 0 | 0 | 0.00355925 | 0.00098677 | 0.00083692 | 0.00136303 | 0.00174376 |
| k__Bacteria;p__AC1;c__SHA-114;o__;f__;g__ | | 0 | 0 | 0 | 0 | 0 | 1.32E-05 | 0 |
| k__Bacteria;p__Acidobacteria;c__;o__;f__;g__ | | 0 | 0 | 0 | 0 | 2.99E-05 | 0 | 0 |
| k__Bacteria;p__Acidobacteria;c__Acidobacteria-6;o__iii1-15;f__;g__ | | 0 | 0 | 0 | 0 | 0 | 0 | 0 |
| k__Bacteria;p__Acidobacteria;c__Acidobacteriia;o__Acidobacteriales;f__Acidobacteriaceae;g__ | | 0 | 0 | 0 | 0 | 0 | 0 | 0 |
| k__Bacteria;p__Acidobacteria;c__Acidobacteriia;o__Acidobacteriales;f__Koribacteraceae;g__ | | 0 | 0 | 0 | 0 | 0 | 0 | 0 |
| k__Bacteria;p__Acidobacteria;c__Acidobacteriia;o__Acidobacteriales;f__Koribacteraceae;g__Candidatus Koribacter | | 0 | 0 | 0 | 0 | 0 | 0 | 0 |
| k__Bacteria;p__Acidobacteria;c__OS-K;o__;f__;g__ | | 0 | 0 | 0 | 0 | 2.99E-05 | 0 | 0 |
| k__Bacteria;p__Acidobacteria;c__Solibacteres;o__Solibacterales;f__;g__ | | 0 | 0 | 0 | 0 | 0 | 0 | 0 |
| k__Bacteria;p__Acidobacteria;c__Solibacteres;o__Solibacterales;f__PAUC26f;g__ | | 0 | 0 | 0 | 0 | 0 | 1.32E-05 | 0 |
| k__Bacteria;p__Acidobacteria;c__Sva0725;o__Sva0725;f__;g__ | | 0 | 0 | 0 | 0 | 0 | 2.65E-05 | 0 |
| k__Bacteria;p__Acidobacteria;c__[Chloracidobacteria];o__PK29;f__;g__ | | 0 | 0 | 0 | 0 | 0 | 0 | 0 |
| k__Bacteria;p__Acidobacteria;c__[Chloracidobacteria];o__RB41;f__;g__ | | 0 | 0 | 0 | 0 | 0 | 0 | 0 |
| k__Bacteria;p__Actinobacteria;c__Acidimicrobiia;o__Acidimicrobiales;f__;g__ | | 0 | 0 | 0 | 0.0001031 | 0.00023912 | 0.00010587 | 6.12E-05 |
| k__Bacteria;p__Actinobacteria;c__Acidimicrobiia;o__Acidimicrobiales;f__C111;g__ | | 1.39E-05 | 8.55E-05 | 0.00041953 | 0.00086895 | 0.00071736 | 0.00082046 | 0.00030592 |
| k__Bacteria;p__Actinobacteria;c__Acidimicrobiia;o__Acidimicrobiales;f__JdFBGBact;g__ | | 0 | 0 | 0 | 4.42E-05 | 8.97E-05 | 2.65E-05 | 0 |
| k__Bacteria;p__Actinobacteria;c__Acidimicrobiia;o__Acidimicrobiales;f__Microthrixaceae;g__ | | 0 | 0 | 0 | 0 | 0 | 0 | 3.06E-05 |
| k__Bacteria;p__Actinobacteria;c__Acidimicrobiia;o__Acidimicrobiales;f__OCS155;g__ | | 0.00012546 | 0.00029909 | 0.00721323 | 0.00656868 | 0.00095648 | 0.0011116 | 0.00657734 |
| k__Bacteria;p__Actinobacteria;c__Acidimicrobiia;o__Acidimicrobiales;f__SC3-41;g__ | | 0.0028855 | 0.00447217 | 0.00219239 | 0.00334325 | 0.00274988 | 0.00181296 | 0.00128488 |
| k__Bacteria;p__Actinobacteria;c__Acidimicrobiia;o__Acidimicrobiales;f__TK06;g__ | | 0 | 0 | 2.71E-05 | 0 | 0 | 0 | 0 |
| k__Bacteria;p__Actinobacteria;c__Acidimicrobiia;o__Acidimicrobiales;f__ZA3409c;g__ | | 0 | 0 | 0 | 0 | 0 | 0 | 0 |
| k__Bacteria;p__Actinobacteria;c__Acidimicrobiia;o__Acidimicrobiales;f__koll13;g__ | | 0 | 0 | 0 | 2.95E-05 | 0 | 5.29E-05 | 0 |
| k__Bacteria;p__Actinobacteria;c__Acidimicrobiia;o__Acidimicrobiales;f__ntu14;g__ | | 0 | 0 | 0 | 0 | 2.99E-05 | 0 | 0 |
| k__Bacteria;p__Actinobacteria;c__Acidimicrobiia;o__Acidimicrobiales;f__wb1_P06;g__ | | 0.00011152 | 0.00021364 | 8.12E-05 | 1.47E-05 | 0 | 5.29E-05 | 0 |
| k__Bacteria;p__Actinobacteria;c__Actinobacteria;o__Actinomycetales;f__;g__ | | 1.39E-05 | 0 | 0.00127213 | 0.00301923 | 0.00218197 | 0.00201146 | 0.00088718 |
| k__Bacteria;p__Actinobacteria;c__Actinobacteria;o__Actinomycetales;f__ACK-M1;g__ | | 0 | 0 | 0 | 0 | 0 | 1.32E-05 | 0 |
| k__Bacteria;p__Actinobacteria;c__Actinobacteria;o__Actinomycetales;f__Actinomycetaceae;g__ | | 0 | 0 | 0 | 0 | 0 | 0 | 0 |
| k__Bacteria;p__Actinobacteria;c__Actinobacteria;o__Actinomycetales;f__Actinomycetaceae;g__Actinomyces | | 0 | 0 | 0 | 0 | 0 | 0 | 0 |
| k__Bacteria;p__Actinobacteria;c__Actinobacteria;o__Actinomycetales;f__Actinomycetaceae;g__Mobiluncus | | 0 | 0 | 0 | 0 | 0 | 0 | 0 |
| k__Bacteria;p__Actinobacteria;c__Actinobacteria;o__Actinomycetales;f__Actinomycetaceae;g__N09 | | 0 | 0 | 0 | 0 | 0 | 0 | 0 |
| k__Bacteria;p__Actinobacteria;c__Actinobacteria;o__Actinomycetales;f__Actinomycetaceae;g__Varibaculum | | 0 | 0 | 0 | 0 | 0 | 0 | 0 |
| k__Bacteria;p__Actinobacteria;c__Actinobacteria;o__Actinomycetales;f__Brevibacteriaceae;g__Brevibacterium | | 0 | 0 | 0 | 0 | 0 | 0 | 0 |
| k__Bacteria;p__Actinobacteria;c__Actinobacteria;o__Actinomycetales;f__Cellulomonadaceae;g__Actinotalea | | 0 | 0 | 0 | 0 | 0 | 1.32E-05 | 0 |
| k__Bacteria;p__Actinobacteria;c__Actinobacteria;o__Actinomycetales;f__Cellulomonadaceae;g__Cellulomonas | | 0 | 0 | 0 | 0 | 0 | 0 | 0 |
| k__Bacteria;p__Actinobacteria;c__Actinobacteria;o__Actinomycetales;f__Cellulomonadaceae;g__Demequina | | 0 | 0 | 0 | 0 | 0 | 0 | 0 |
| k__Bacteria;p__Actinobacteria;c__Actinobacteria;o__Actinomycetales;f__Corynebacteriaceae;g__Corynebacterium | | 0 | 2.85E-05 | 0 | 0 | 0 | 0 | 0 |
| k__Bacteria;p__Actinobacteria;c__Actinobacteria;o__Actinomycetales;f__Dermabacteraceae;g__Brachybacterium | | 0 | 0 | 0 | 0 | 0 | 0 | 0 |
| k__Bacteria;p__Actinobacteria;c__Actinobacteria;o__Actinomycetales;f__Dermacoccaceae;g__Dermacoccus | | 0 | 0 | 0 | 0 | 0 | 0 | 0 |
| k__Bacteria;p__Actinobacteria;c__Actinobacteria;o__Actinomycetales;f__Dietziaceae;g__Dietzia | | 0 | 0 | 0 | 0 | 0 | 0 | 0 |
| k__Bacteria;p__Actinobacteria;c__Actinobacteria;o__Actinomycetales;f__Frankiaceae;g__ | | 0 | 0 | 0 | 0 | 0 | 0 | 0 |
| k__Bacteria;p__Actinobacteria;c__Actinobacteria;o__Actinomycetales;f__Geodermatophilaceae;g__ | | 0 | 0 | 0 | 0 | 2.99E-05 | 0 | 0 |
| k__Bacteria;p__Actinobacteria;c__Actinobacteria;o__Actinomycetales;f__Geodermatophilaceae;g__Blastococcus | | 4.18E-05 | 0 | 0 | 0 | 0 | 0 | 0 |
| k__Bacteria;p__Actinobacteria;c__Actinobacteria;o__Actinomycetales;f__Gordoniaceae;g__Gordonia | | 0 | 0 | 0 | 0 | 0 | 0 | 0 |
| k__Bacteria;p__Actinobacteria;c__Actinobacteria;o__Actinomycetales;f__Intrasporangiaceae;g__ | | 0 | 0 | 0 | 0 | 0 | 0 | 0 |
| k__Bacteria;p__Actinobacteria;c__Actinobacteria;o__Actinomycetales;f__Intrasporangiaceae;g__Knoellia | | 0 | 0 | 0 | 0 | 0 | 0 | 0 |
| k__Bacteria;p__Actinobacteria;c__Actinobacteria;o__Actinomycetales;f__Intrasporangiaceae;g__Oryzihumus | | 0 | 0 | 0 | 0 | 0 | 0 | 0 |
| k__Bacteria;p__Actinobacteria;c__Actinobacteria;o__Actinomycetales;f__Intrasporangiaceae;g__Phycicoccus | | 0 | 0 | 0 | 0 | 0 | 0 | 0 |
| k__Bacteria;p__Actinobacteria;c__Actinobacteria;o__Actinomycetales;f__Intrasporangiaceae;g__Tetrasphaera | | 0 | 0 | 0 | 0 | 0 | 0 | 0 |
| k__Bacteria;p__Actinobacteria;c__Actinobacteria;o__Actinomycetales;f__Kineosporiaceae;g__ | | 0 | 0 | 0 | 0 | 2.99E-05 | 0 | 0 |
| k__Bacteria;p__Actinobacteria;c__Actinobacteria;o__Actinomycetales;f__Microbacteriaceae;g__ | | 0.00015334 | 0.00025637 | 0.00192172 | 0.00858641 | 0.03195242 | 0.02174229 | 0.01447014 |
| k__Bacteria;p__Actinobacteria;c__Actinobacteria;o__Actinomycetales;f__Microbacteriaceae;g__Agrococcus | | 0 | 0 | 0 | 0 | 0 | 0 | 0 |
| k__Bacteria;p__Actinobacteria;c__Actinobacteria;o__Actinomycetales;f__Microbacteriaceae;g__Candidatus Aquiluna | | 0.00029273 | 0.00049849 | 0.00274725 | 0.01005921 | 0.01835246 | 0.01175116 | 0.00951419 |
| k__Bacteria;p__Actinobacteria;c__Actinobacteria;o__Actinomycetales;f__Microbacteriaceae;g__Candidatus Rhodoluna | | 0 | 0 | 0 | 0 | 2.99E-05 | 0 | 0 |
| k__Bacteria;p__Actinobacteria;c__Actinobacteria;o__Actinomycetales;f__Microbacteriaceae;g__Cryocola | | 0 | 0 | 0 | 0 | 0 | 0 | 0 |
| k__Bacteria;p__Actinobacteria;c__Actinobacteria;o__Actinomycetales;f__Microbacteriaceae;g__Curtobacterium | | 0 | 0 | 0 | 0 | 0 | 0 | 0 |
| k__Bacteria;p__Actinobacteria;c__Actinobacteria;o__Actinomycetales;f__Microbacteriaceae;g__Frigoribacterium | | 0 | 0 | 0 | 0 | 0 | 0 | 0 |
| k__Bacteria;p__Actinobacteria;c__Actinobacteria;o__Actinomycetales;f__Microbacteriaceae;g__Herbiconiux | | 0 | 0 | 0 | 0 | 0 | 0 | 0 |
| k__Bacteria;p__Actinobacteria;c__Actinobacteria;o__Actinomycetales;f__Microbacteriaceae;g__Microbacterium | | 0 | 0 | 0 | 0 | 0 | 0 | 0 |
| k__Bacteria;p__Actinobacteria;c__Actinobacteria;o__Actinomycetales;f__Microbacteriaceae;g__Mycetocola | | 0 | 0 | 0 | 0 | 0 | 0 | 0 |
| k__Bacteria;p__Actinobacteria;c__Actinobacteria;o__Actinomycetales;f__Microbacteriaceae;g__Pseudoclavibacter | | 0 | 0 | 0 | 0 | 0 | 0 | 0 |
| k__Bacteria;p__Actinobacteria;c__Actinobacteria;o__Actinomycetales;f__Microbacteriaceae;g__Salinibacterium | | 0 | 0 | 0 | 0 | 0 | 0 | 0 |
| k__Bacteria;p__Actinobacteria;c__Actinobacteria;o__Actinomycetales;f__Microbacteriaceae;g__Yonghaparkia | | 1.39E-05 | 0 | 0 | 0 | 0 | 0 | 0 |
| k__Bacteria;p__Actinobacteria;c__Actinobacteria;o__Actinomycetales;f__Micrococcaceae;g__ | | 0 | 0 | 0 | 0 | 0 | 0 | 0 |
| k__Bacteria;p__Actinobacteria;c__Actinobacteria;o__Actinomycetales;f__Micrococcaceae;g__Arthrobacter | | 0 | 0 | 1.35E-05 | 0 | 0 | 0 | 0 |
| k__Bacteria;p__Actinobacteria;c__Actinobacteria;o__Actinomycetales;f__Micrococcaceae;g__Kocuria | | 0 | 1.42E-05 | 0 | 0 | 0 | 0 | 0 |
| k__Bacteria;p__Actinobacteria;c__Actinobacteria;o__Actinomycetales;f__Micrococcaceae;g__Microbispora | | 0 | 0 | 0 | 0 | 0 | 0 | 0 |
| k__Bacteria;p__Actinobacteria;c__Actinobacteria;o__Actinomycetales;f__Micrococcaceae;g__Micrococcus | | 0 | 0 | 0 | 0 | 0 | 0 | 0 |
| k__Bacteria;p__Actinobacteria;c__Actinobacteria;o__Actinomycetales;f__Micrococcaceae;g__Renibacterium | | 0 | 0 | 0 | 0 | 0 | 0 | 0 |
| k__Bacteria;p__Actinobacteria;c__Actinobacteria;o__Actinomycetales;f__Micrococcaceae;g__Rothia | | 0 | 2.85E-05 | 0 | 0 | 2.99E-05 | 1.32E-05 | 0 |
| k__Bacteria;p__Actinobacteria;c__Actinobacteria;o__Actinomycetales;f__Micromonosporaceae;g__ | | 0 | 0 | 0 | 0 | 0 | 0 | 0 |
| k__Bacteria;p__Actinobacteria;c__Actinobacteria;o__Actinomycetales;f__Micromonosporaceae;g__Phytohabitans | | 0 | 0 | 0 | 0 | 0 | 0 | 0 |
| k__Bacteria;p__Actinobacteria;c__Actinobacteria;o__Actinomycetales;f__Micromonosporaceae;g__Verrucosispora | | 0 | 0 | 0 | 0 | 0 | 0 | 0 |

| Taxon | | 2028 | 2029 | 1184 | 1196 | 1245 | 1246 | 2019 |
|---|---|---|---|---|---|---|---|---|
| | Copepod genus | Centropages | Centropages | Water | Water | Water | Water | Water |
| | Type of sample | Starved | Starved | Water | Water | Water | Water | Water |
| k__Bacteria;p__Actinobacteria;c__Actinobacteria;o__Actinomycetales;f__Mycobacteriaceae;g__Mycobacterium | | 0 | 0 | 0 | 0 | 0 | 0 | 0 |
| k__Bacteria;p__Actinobacteria;c__Actinobacteria;o__Actinomycetales;f__Nakamurellaceae;g__ | | 0 | 0 | 0 | 0 | 0 | 1.32E-05 | 0 |
| k__Bacteria;p__Actinobacteria;c__Actinobacteria;o__Actinomycetales;f__Nocardiaceae;g__ | | 0 | 0 | 0 | 0 | 0 | 0 | 0 |
| k__Bacteria;p__Actinobacteria;c__Actinobacteria;o__Actinomycetales;f__Nocardiaceae;g__Rhodococcus | | 0 | 0 | 0 | 0 | 0 | 0 | 0 |
| k__Bacteria;p__Actinobacteria;c__Actinobacteria;o__Actinomycetales;f__Nocardioidaceae;g__ | | 0 | 0 | 0 | 0 | 0 | 0 | 0 |
| k__Bacteria;p__Actinobacteria;c__Actinobacteria;o__Actinomycetales;f__Nocardioidaceae;g__Aeromicrobium | | 0 | 0 | 0 | 0 | 0 | 0 | 0 |
| k__Bacteria;p__Actinobacteria;c__Actinobacteria;o__Actinomycetales;f__Nocardioidaceae;g__Friedmanniella | | 0 | 0 | 0 | 0 | 0 | 0 | 0 |
| k__Bacteria;p__Actinobacteria;c__Actinobacteria;o__Actinomycetales;f__Nocardioidaceae;g__Nocardioides | | 0 | 0 | 0 | 0 | 0 | 0 | 0 |
| k__Bacteria;p__Actinobacteria;c__Actinobacteria;o__Actinomycetales;f__Nocardioidaceae;g__Propionicimonas | | 0 | 0 | 0 | 0 | 0 | 0 | 0 |
| k__Bacteria;p__Actinobacteria;c__Actinobacteria;o__Actinomycetales;f__Promicromonosporaceae;g__Promicromonospora | | 0 | 0 | 0 | 0 | 0 | 0 | 0 |
| k__Bacteria;p__Actinobacteria;c__Actinobacteria;o__Actinomycetales;f__Propionibacteriaceae;g__ | | 0 | 0 | 0 | 0 | 0 | 0 | 0 |
| k__Bacteria;p__Actinobacteria;c__Actinobacteria;o__Actinomycetales;f__Propionibacteriaceae;g__Propionibacterium | | 5.58E-05 | 1.42E-05 | 0 | 1.47E-05 | 0 | 1.32E-05 | 0 |
| k__Bacteria;p__Actinobacteria;c__Actinobacteria;o__Actinomycetales;f__Pseudonocardiaceae;g__Pseudonocardia | | 0 | 0 | 0 | 0 | 0 | 0 | 0 |
| k__Bacteria;p__Actinobacteria;c__Actinobacteria;o__Actinomycetales;f__Pseudonocardiaceae;g__Saccharopolyspora | | 0 | 0 | 0 | 0 | 0 | 0 | 0 |
| k__Bacteria;p__Actinobacteria;c__Actinobacteria;o__Actinomycetales;f__Sanguibacteraceae;g__Sanguibacter | | 0 | 0 | 0 | 0 | 0 | 0 | 0 |
| k__Bacteria;p__Actinobacteria;c__Actinobacteria;o__Actinomycetales;f__Sporichthyaceae;g__ | | 0 | 0 | 0 | 0 | 0 | 0 | 0 |
| k__Bacteria;p__Actinobacteria;c__Actinobacteria;o__Actinomycetales;f__Streptomycetaceae;g__ | | 0 | 0 | 0 | 0 | 0 | 0 | 0 |
| k__Bacteria;p__Actinobacteria;c__Actinobacteria;o__Actinomycetales;f__Williamsiaceae;g__Williamsia | | 0 | 0 | 0 | 0 | 0 | 0 | 0 |
| k__Bacteria;p__Actinobacteria;c__Actinobacteria;o__Actinomycetales;f__Yaniellaceae;g__Auritibacter | | 0 | 0 | 0 | 0 | 0 | 0 | 0 |
| k__Bacteria;p__Actinobacteria;c__Actinobacteria;o__Bifidobacteriales;f__Bifidobacteriaceae;g__ | | 0 | 0 | 0 | 0 | 0 | 0 | 0 |
| k__Bacteria;p__Actinobacteria;c__Actinobacteria;o__Bifidobacteriales;f__Bifidobacteriaceae;g__Alloscardovia | | 0 | 0 | 0 | 0 | 0 | 0 | 0 |
| k__Bacteria;p__Actinobacteria;c__Actinobacteria;o__Bifidobacteriales;f__Bifidobacteriaceae;g__Bifidobacterium | | 0 | 0 | 0 | 0 | 0 | 0 | 0 |
| k__Bacteria;p__Actinobacteria;c__Actinobacteria;o__Bifidobacteriales;f__Bifidobacteriaceae;g__Gardnerella | | 0 | 0 | 0 | 0 | 0 | 0 | 0 |
| k__Bacteria;p__Actinobacteria;c__Actinobacteria;o__Bifidobacteriales;f__Bifidobacteriaceae;g__Scardovia | | 0 | 0 | 0 | 0 | 0 | 0 | 0 |
| k__Bacteria;p__Actinobacteria;c__Actinobacteria;o__WCHB1-81;f__At425_EubF1;g__ | | 0 | 0 | 0 | 0 | 2.99E-05 | 0 | 0 |
| k__Bacteria;p__Actinobacteria;c__Coriobacteriia;o__Coriobacteriales;f__Coriobacteriaceae;g__Atopobium | | 0 | 0 | 0 | 0 | 0 | 0 | 0 |
| k__Bacteria;p__Actinobacteria;c__Rubrobacteria;o__Rubrobacterales;f__Rubrobacteraceae;g__Rubrobacter | | 0 | 0 | 0 | 0 | 0 | 0 | 0 |
| k__Bacteria;p__Actinobacteria;c__Thermoleophilia;o__Gaiellales;f__Gaiellaceae;g__ | | 0 | 0 | 0 | 0 | 0 | 0 | 0 |
| k__Bacteria;p__Actinobacteria;c__Thermoleophilia;o__Solirubrobacterales;f__Conexibacteraceae;g__ | | 0 | 0 | 0 | 0 | 0 | 0 | 0 |
| k__Bacteria;p__Actinobacteria;c__Thermoleophilia;o__Solirubrobacterales;f__Patulibacteraceae;g__ | | 0 | 0 | 0 | 0 | 0 | 0 | 0 |
| k__Bacteria;p__Actinobacteria;c__Thermoleophilia;o__Solirubrobacterales;f__Solirubrobacteraceae;g__ | | 0 | 0 | 0 | 0 | 0 | 0 | 0 |
| k__Bacteria;p__Actinobacteria;c__Thermoleophilia;o__Solirubrobacterales;f__Solirubrobacteraceae;g__Solirubrobacter | | 0 | 0 | 0 | 0 | 0 | 0 | 0 |
| k__Bacteria;p__Bacteroidetes;c__Bacteroidia;o__Bacteroidales;f__;g__ | | 0 | 0 | 0 | 4.42E-05 | 0.00026901 | 0.0001588 | 6.12E-05 |
| k__Bacteria;p__Bacteroidetes;c__Bacteroidia;o__Bacteroidales;f__Bacteroidaceae;g__Bacteroides | | 0 | 0 | 2.71E-05 | 0 | 0.00020923 | 0.0001985 | 0 |
| k__Bacteria;p__Bacteroidetes;c__Bacteroidia;o__Bacteroidales;f__Porphyromonadaceae;g__ | | 0 | 0 | 0 | 0 | 8.97E-05 | 5.29E-05 | 0 |
| k__Bacteria;p__Bacteroidetes;c__Bacteroidia;o__Bacteroidales;f__Porphyromonadaceae;g__Paludibacter | | 0 | 0 | 0 | 0 | 0 | 0 | 0 |
| k__Bacteria;p__Bacteroidetes;c__Bacteroidia;o__Bacteroidales;f__Porphyromonadaceae;g__Parabacteroides | | 0 | 0 | 0 | 0 | 2.99E-05 | 2.65E-05 | 0 |
| k__Bacteria;p__Bacteroidetes;c__Bacteroidia;o__Bacteroidales;f__Porphyromonadaceae;g__Porphyromonas | | 4.18E-05 | 0 | 0 | 0 | 0 | 0 | 0 |
| k__Bacteria;p__Bacteroidetes;c__Bacteroidia;o__Bacteroidales;f__Prevotellaceae;g__Prevotella | | 0 | 0 | 0 | 0 | 0.00014945 | 5.29E-05 | 3.06E-05 |
| k__Bacteria;p__Bacteroidetes;c__Bacteroidia;o__Bacteroidales;f__Rikenellaceae;g__ | | 0 | 0 | 0 | 0 | 0 | 2.65E-05 | 0 |
| k__Bacteria;p__Bacteroidetes;c__Bacteroidia;o__Bacteroidales;f__SB-1;g__ | | 0 | 0 | 0 | 0 | 0 | 2.65E-05 | 0 |
| k__Bacteria;p__Bacteroidetes;c__Bacteroidia;o__Bacteroidales;f__[Odoribacteraceae];g__Butyricimonas | | 0 | 0 | 0 | 0 | 2.99E-05 | 0 | 0 |
| k__Bacteria;p__Bacteroidetes;c__Bacteroidia;o__Bacteroidales;f__[Odoribacteraceae];g__Odoribacter | | 0 | 0 | 0 | 0 | 2.99E-05 | 3.97E-05 | 0 |
| k__Bacteria;p__Bacteroidetes;c__Bacteroidia;o__Bacteroidales;f__[Paraprevotellaceae];g__[Prevotella] | | 0 | 0 | 0 | 0 | 0 | 0 | 0 |
| k__Bacteria;p__Bacteroidetes;c__Cytophagia;o__Cytophagales;f__Cyclobacteriaceae;g__ | | 0 | 0 | 0 | 0 | 5.98E-05 | 0 | 0 |
| k__Bacteria;p__Bacteroidetes;c__Cytophagia;o__Cytophagales;f__Cytophagaceae;g__ | | 0 | 0 | 0 | 0 | 0 | 0 | 0 |
| k__Bacteria;p__Bacteroidetes;c__Cytophagia;o__Cytophagales;f__Cytophagaceae;g__Adhaeribacter | | 0 | 0 | 0 | 0 | 0 | 0 | 0 |
| k__Bacteria;p__Bacteroidetes;c__Cytophagia;o__Cytophagales;f__Cytophagaceae;g__Cytophaga | | 0 | 0 | 0 | 0 | 0 | 0 | 0 |
| k__Bacteria;p__Bacteroidetes;c__Cytophagia;o__Cytophagales;f__Cytophagaceae;g__Hymenobacter | | 0 | 0 | 0 | 0 | 2.99E-05 | 2.65E-05 | 0 |
| k__Bacteria;p__Bacteroidetes;c__Cytophagia;o__Cytophagales;f__Cytophagaceae;g__Leadbetterella | | 0 | 0 | 0 | 0 | 0 | 0 | 0 |
| k__Bacteria;p__Bacteroidetes;c__Cytophagia;o__Cytophagales;f__Cytophagaceae;g__Spirosoma | | 0 | 0 | 0 | 0 | 0 | 0 | 0 |
| k__Bacteria;p__Bacteroidetes;c__Cytophagia;o__Cytophagales;f__Flammeovirgaceae;g__ | | 0.00012546 | 0.00061243 | 0.00140746 | 0.00293087 | 0.00065758 | 0.00064843 | 0.00064244 |
| k__Bacteria;p__Bacteroidetes;c__Cytophagia;o__Cytophagales;f__Flammeovirgaceae;g__JTB248 | | 0 | 1.42E-05 | 0 | 0.00013255 | 0 | 0 | 9.18E-05 |
| k__Bacteria;p__Bacteroidetes;c__Cytophagia;o__Cytophagales;f__Flammeovirgaceae;g__Roseivirga | | 0 | 0 | 4.06E-05 | 0 | 0 | 0 | 0 |
| k__Bacteria;p__Bacteroidetes;c__Cytophagia;o__Cytophagales;f__[Amoebophilaceae];g__SC3-56 | | 0 | 0 | 0 | 0 | 2.99E-05 | 0 | 0 |
| k__Bacteria;p__Bacteroidetes;c__Flavobacteriia;o__Flavobacteriales;f__;g__ | | 0.00146366 | 0.00300518 | 0.00382991 | 0.00544935 | 0.01156743 | 0.01290246 | 0.0043441 |
| k__Bacteria;p__Bacteroidetes;c__Flavobacteriia;o__Flavobacteriales;f__Cryomorphaceae;g__ | | 0.00078062 | 0.00136729 | 0.02076003 | 0.00843913 | 0.04770445 | 0.03733111 | 0.01275698 |
| k__Bacteria;p__Bacteroidetes;c__Flavobacteriia;o__Flavobacteriales;f__Cryomorphaceae;g__Crocinitomix | | 4.18E-05 | 0.00012818 | 2.71E-05 | 8.84E-05 | 5.98E-05 | 5.29E-05 | 0.00018355 |
| k__Bacteria;p__Bacteroidetes;c__Flavobacteriia;o__Flavobacteriales;f__Cryomorphaceae;g__Fluviicola | | 0.00223034 | 0.00361762 | 0.00397878 | 0.00300451 | 0.00257054 | 0.00304366 | 0.00244738 |
| k__Bacteria;p__Bacteroidetes;c__Flavobacteriia;o__Flavobacteriales;f__Flavobacteriaceae;g__ | | 0.02208035 | 0.03996468 | 0.12399177 | 0.1216678 | 0.08721903 | 0.09456509 | 0.12160426 |
| k__Bacteria;p__Bacteroidetes;c__Flavobacteriia;o__Flavobacteriales;f__Flavobacteriaceae;g__Algibacter | | 0 | 0 | 2.71E-05 | 0.00011782 | 8.97E-05 | 1.32E-05 | 0 |
| k__Bacteria;p__Bacteroidetes;c__Flavobacteriia;o__Flavobacteriales;f__Flavobacteriaceae;g__Aquimarina | | 0 | 0 | 0 | 1.47E-05 | 2.99E-05 | 2.65E-05 | 6.12E-05 |
| k__Bacteria;p__Bacteroidetes;c__Flavobacteriia;o__Flavobacteriales;f__Flavobacteriaceae;g__Bizionia | | 0 | 0 | 6.77E-05 | 4.42E-05 | 0 | 5.29E-05 | 3.06E-05 |
| k__Bacteria;p__Bacteroidetes;c__Flavobacteriia;o__Flavobacteriales;f__Flavobacteriaceae;g__Capnocytophaga | | 0 | 0 | 0 | 0 | 0 | 0 | 0 |
| k__Bacteria;p__Bacteroidetes;c__Flavobacteriia;o__Flavobacteriales;f__Flavobacteriaceae;g__Cellulophaga | | 0 | 0 | 0 | 1.47E-05 | 0 | 1.32E-05 | 0 |
| k__Bacteria;p__Bacteroidetes;c__Flavobacteriia;o__Flavobacteriales;f__Flavobacteriaceae;g__Coccinimonas | | 1.39E-05 | 0.00011394 | 0 | 0 | 0 | 0 | 0 |
| k__Bacteria;p__Bacteroidetes;c__Flavobacteriia;o__Flavobacteriales;f__Flavobacteriaceae;g__Flavobacterium | | 0.00816861 | 0.01672079 | 0.0190413 | 0.02866064 | 0.02289574 | 0.02523588 | 0.01918135 |
| k__Bacteria;p__Bacteroidetes;c__Flavobacteriia;o__Flavobacteriales;f__Flavobacteriaceae;g__Formosa | | 0 | 2.85E-05 | 0.00027067 | 0.00085422 | 0.00056791 | 0.00038377 | 0.00018355 |
| k__Bacteria;p__Bacteroidetes;c__Flavobacteriia;o__Flavobacteriales;f__Flavobacteriaceae;g__Gaetbulibacter | | 0 | 0 | 1.35E-05 | 2.95E-05 | 2.99E-05 | 5.29E-05 | 0.00012237 |
| k__Bacteria;p__Bacteroidetes;c__Flavobacteriia;o__Flavobacteriales;f__Flavobacteriaceae;g__Gillisia | | 1.39E-05 | 2.85E-05 | 0 | 0 | 0 | 0 | 0 |
| k__Bacteria;p__Bacteroidetes;c__Flavobacteriia;o__Flavobacteriales;f__Flavobacteriaceae;g__Gilvibacter | | 0 | 0 | 0 | 2.95E-05 | 2.99E-05 | 0 | 3.06E-05 |
| k__Bacteria;p__Bacteroidetes;c__Flavobacteriia;o__Flavobacteriales;f__Flavobacteriaceae;g__Gramella | | 4.18E-05 | 7.12E-05 | 0 | 0 | 0 | 0 | 0 |
| k__Bacteria;p__Bacteroidetes;c__Flavobacteriia;o__Flavobacteriales;f__Flavobacteriaceae;g__Kordia | | 0 | 0 | 0 | 0 | 0 | 1.32E-05 | 0 |
| k__Bacteria;p__Bacteroidetes;c__Flavobacteriia;o__Flavobacteriales;f__Flavobacteriaceae;g__Krokinobacter | | 6.97E-05 | 0.00011394 | 0 | 0 | 0 | 1.32E-05 | 3.06E-05 |

| Taxon | 2028 | 2029 | 1184 | 1196 | 1245 | 1246 | 2019 |
|---|---|---|---|---|---|---|---|
| Copepod genus | Centropages | Centropages | Water | Water | Water | Water | Water |
| Type of sample | Starved | Starved | Water | Water | Water | Water | Water |
| k__Bacteria;p__Bacteroidetes;c__Flavobacteriia;o__Flavobacteriales;f__Flavobacteriaceae;g__Lacinutrix | 4.18E-05 | 1.42E-05 | 0.0001624 | 0.00013255 | 0.00017934 | 0.00026467 | 0.00535365 |
| k__Bacteria;p__Bacteroidetes;c__Flavobacteriia;o__Flavobacteriales;f__Flavobacteriaceae;g__Leeuwenhoekiella | 6.97E-05 | 2.85E-05 | 0 | 0 | 0 | 0 | 3.06E-05 |
| k__Bacteria;p__Bacteroidetes;c__Flavobacteriia;o__Flavobacteriales;f__Flavobacteriaceae;g__Lutimonas | 0 | 0 | 1.35E-05 | 5.89E-05 | 5.98E-05 | 5.29E-05 | 0 |
| k__Bacteria;p__Bacteroidetes;c__Flavobacteriia;o__Flavobacteriales;f__Flavobacteriaceae;g__Maribacter | 4.18E-05 | 8.55E-05 | 2.71E-05 | 8.84E-05 | 0 | 5.29E-05 | 9.18E-05 |
| k__Bacteria;p__Bacteroidetes;c__Flavobacteriia;o__Flavobacteriales;f__Flavobacteriaceae;g__Mesonia | 6.97E-05 | 2.85E-05 | 0 | 0 | 2.99E-05 | 3.97E-05 | 0 |
| k__Bacteria;p__Bacteroidetes;c__Flavobacteriia;o__Flavobacteriales;f__Flavobacteriaceae;g__Olleya | 0.0022861 | 0.00286276 | 4.06E-05 | 0 | 0.00011956 | 6.62E-05 | 0.00486417 |
| k__Bacteria;p__Bacteroidetes;c__Flavobacteriia;o__Flavobacteriales;f__Flavobacteriaceae;g__Persicivirga | 0.00011152 | 0.00022788 | 0 | 0 | 0 | 0 | 0 |
| k__Bacteria;p__Bacteroidetes;c__Flavobacteriia;o__Flavobacteriales;f__Flavobacteriaceae;g__Polaribacter | 0.00122669 | 0.00209366 | 0.01148974 | 0.01044213 | 0.02128168 | 0.02293329 | 0.01064611 |
| k__Bacteria;p__Bacteroidetes;c__Flavobacteriia;o__Flavobacteriales;f__Flavobacteriaceae;g__Pontirhabdus | 0 | 0 | 0 | 2.95E-05 | 0 | 3.97E-05 | 0 |
| k__Bacteria;p__Bacteroidetes;c__Flavobacteriia;o__Flavobacteriales;f__Flavobacteriaceae;g__Psychroserpens | 0.00016728 | 0.00021364 | 0.0008932 | 0.00145807 | 0.00062769 | 0.00124393 | 0.00718918 |
| k__Bacteria;p__Bacteroidetes;c__Flavobacteriia;o__Flavobacteriales;f__Flavobacteriaceae;g__Robiginitalea | 0 | 0 | 0 | 0 | 2.99E-05 | 3.97E-05 | 0 |
| k__Bacteria;p__Bacteroidetes;c__Flavobacteriia;o__Flavobacteriales;f__Flavobacteriaceae;g__Sediminicola | 0.00900499 | 0.01915627 | 0.00933795 | 0.00845386 | 0.02561573 | 0.02650628 | 0.00452766 |
| k__Bacteria;p__Bacteroidetes;c__Flavobacteriia;o__Flavobacteriales;f__Flavobacteriaceae;g__Tenacibaculum | 0.00160306 | 0.0036461 | 0.00403291 | 0.00550826 | 0.01099952 | 0.01423902 | 0.0035793 |
| k__Bacteria;p__Bacteroidetes;c__Flavobacteriia;o__Flavobacteriales;f__Flavobacteriaceae;g__Ulvibacter | 0.00016728 | 0.00014243 | 0.00147513 | 0.00176736 | 0.00107604 | 0.00116453 | 0.00048948 |
| k__Bacteria;p__Bacteroidetes;c__Flavobacteriia;o__Flavobacteriales;f__Flavobacteriaceae;g__Winogradskyella | 5.58E-05 | 2.85E-05 | 0.0001218 | 0.00029456 | 0.00035868 | 0.0008734 | 0.00045888 |
| k__Bacteria;p__Bacteroidetes;c__Flavobacteriia;o__Flavobacteriales;f__NS9;g__ | 0.00041819 | 0.00059819 | 0.00710496 | 0.00297505 | 0.00146461 | 0.00182619 | 0.00296745 |
| k__Bacteria;p__Bacteroidetes;c__Flavobacteriia;o__Flavobacteriales;f__[Weeksellaceae];g__ | 0 | 4.27E-05 | 0 | 0 | 0 | 0 | 0 |
| k__Bacteria;p__Bacteroidetes;c__Flavobacteriia;o__Flavobacteriales;f__[Weeksellaceae];g__Chryseobacterium | 0 | 0 | 0 | 0 | 0 | 0 | 0 |
| k__Bacteria;p__Bacteroidetes;c__Flavobacteriia;o__Flavobacteriales;f__[Weeksellaceae];g__Cloacibacterium | 0 | 0 | 0 | 0 | 0 | 0 | 0 |
| k__Bacteria;p__Bacteroidetes;c__Flavobacteriia;o__Flavobacteriales;f__[Weeksellaceae];g__Weeksella | 0 | 0 | 0 | 0 | 0 | 0 | 0 |
| k__Bacteria;p__Bacteroidetes;c__Sphingobacteriia;o__Sphingobacteriales;f__;g__ | 0.003694 | 0.00609582 | 1.35E-05 | 1.47E-05 | 0.00014945 | 0.00021173 | 0.00018355 |
| k__Bacteria;p__Bacteroidetes;c__Sphingobacteriia;o__Sphingobacteriales;f__NS11-12;g__ | 5.58E-05 | 0 | 0.00224652 | 0.00145807 | 0.00280966 | 0.00203793 | 0.00104014 |
| k__Bacteria;p__Bacteroidetes;c__Sphingobacteriia;o__Sphingobacteriales;f__Sphingobacteriaceae;g__Pedobacter | 0 | 0 | 0 | 0 | 2.99E-05 | 0 | 0 |
| k__Bacteria;p__Bacteroidetes;c__Sphingobacteriia;o__Sphingobacteriales;f__Sphingobacteriaceae;g__Sphingobacterium | 0 | 0 | 0 | 0 | 0 | 0 | 0 |
| k__Bacteria;p__Bacteroidetes;c__VC2_1_Bac22;o__;f__;g__ | 0 | 0 | 0 | 0 | 0 | 0 | 0 |
| k__Bacteria;p__Bacteroidetes;c__[Rhodothermi];o__[Rhodothermales];f__[Balneolaceae];g__Balneola | 0 | 0 | 0 | 0 | 0 | 1.32E-05 | 0 |
| k__Bacteria;p__Bacteroidetes;c__[Saprospirae];o__[Saprospirales];f__;g__ | 0 | 0 | 0 | 0 | 0 | 0 | 6.12E-05 |
| k__Bacteria;p__Bacteroidetes;c__[Saprospirae];o__[Saprospirales];f__Chitinophagaceae;g__ | 1.39E-05 | 0 | 0 | 1.47E-05 | 0 | 0 | 0 |
| k__Bacteria;p__Bacteroidetes;c__[Saprospirae];o__[Saprospirales];f__Chitinophagaceae;g__Chitinophaga | 0 | 0 | 0 | 0 | 0 | 0 | 0 |
| k__Bacteria;p__Bacteroidetes;c__[Saprospirae];o__[Saprospirales];f__Chitinophagaceae;g__Niabella | 0 | 0 | 0 | 0 | 0 | 0 | 0 |
| k__Bacteria;p__Bacteroidetes;c__[Saprospirae];o__[Saprospirales];f__Chitinophagaceae;g__Sediminibacterium | 0 | 0 | 0 | 0 | 0 | 0 | 0 |
| k__Bacteria;p__Bacteroidetes;c__[Saprospirae];o__[Saprospirales];f__Saprospiraceae;g__ | 0 | 2.85E-05 | 0.00078493 | 0.00076585 | 0.00068747 | 0.00041023 | 0.00055066 |
| k__Bacteria;p__Bacteroidetes;c__[Saprospirae];o__[Saprospirales];f__Saprospiraceae;g__Lewinella | 0 | 0 | 0 | 4.42E-05 | 5.98E-05 | 2.65E-05 | 0 |
| k__Bacteria;p__Bacteroidetes;c__[Saprospirae];o__[Saprospirales];f__Saprospiraceae;g__Saprospira | 0 | 0 | 0.00021653 | 0.00064803 | 5.98E-05 | 9.26E-05 | 3.06E-05 |
| k__Bacteria;p__Chlamydiae;c__Chlamydiia;o__Chlamydiales;f__;g__ | 0 | 0 | 0 | 0 | 0 | 1.32E-05 | 0 |
| k__Bacteria;p__Chloroflexi;c__Anaerolineae;o__Caldilineales;f__Caldilineaceae;g__ | 0 | 0 | 0 | 0 | 0 | 0 | 0 |
| k__Bacteria;p__Chloroflexi;c__Anaerolineae;o__GCA004;f__;g__ | 0 | 0 | 0 | 0 | 0.00011956 | 0 | 0 |
| k__Bacteria;p__Chloroflexi;c__Chloroflexi;o__[Roseiflexales];f__;g__ | 0 | 0 | 0 | 0 | 0 | 0 | 0 |
| k__Bacteria;p__Chloroflexi;c__Ellin6529;o__;f__;g__ | 0 | 0 | 0 | 0 | 0 | 0 | 0 |
| k__Bacteria;p__Chloroflexi;c__Gitt-GS-136;o__;f__;g__ | 0 | 0 | 0 | 0 | 0 | 0 | 0 |
| k__Bacteria;p__Chloroflexi;c__S085;o__;f__;g__ | 0 | 0 | 0 | 0 | 0 | 0 | 0 |
| k__Bacteria;p__Chloroflexi;c__SAR202;o__;f__;g__ | 0 | 0 | 1.35E-05 | 0 | 0 | 0 | 0 |
| k__Bacteria;p__Chloroflexi;c__TK10;o__AKYG885;f__Dolo_23;g__ | 0 | 0 | 0 | 0 | 0 | 0 | 0 |
| k__Bacteria;p__Cyanobacteria;c__4C0d-2;o__MLE1-12;f__;g__ | 0 | 0 | 0 | 0 | 0 | 0 | 0 |
| k__Bacteria;p__Cyanobacteria;c__4C0d-2;o__YS2;f__;g__ | 0 | 0 | 0 | 0 | 2.99E-05 | 1.32E-05 | 0 |
| k__Bacteria;p__Cyanobacteria;c__Chloroplast;o__;f__;g__ | 5.58E-05 | 0 | 0.00021653 | 0.0002651 | 0.00182329 | 0.00189236 | 0.00027533 |
| k__Bacteria;p__Cyanobacteria;c__Chloroplast;o__Cercozoa;f__;g__ | 0 | 0 | 0 | 0 | 0 | 5.29E-05 | 0 |
| k__Bacteria;p__Cyanobacteria;c__Chloroplast;o__Chlorophyta;f__;g__ | 0.0014079 | 0.00501339 | 0.00116386 | 0.001841 | 0.00140483 | 0.00210409 | 0.00024474 |
| k__Bacteria;p__Cyanobacteria;c__Chloroplast;o__Chlorophyta;f__Mamiellaceae;g__ | 0.00119881 | 0.00649462 | 0.00217886 | 0.00356417 | 0.00230153 | 0.00637845 | 0.00061185 |
| k__Bacteria;p__Cyanobacteria;c__Chloroplast;o__Chlorophyta;f__Mamiellaceae;g__Micromonas | 0 | 4.27E-05 | 5.41E-05 | 0.00011782 | 5.98E-05 | 0.0001588 | 9.18E-05 |
| k__Bacteria;p__Cyanobacteria;c__Chloroplast;o__Chlorophyta;f__Trebouxiophyceae;g__ | 0 | 0 | 0 | 1.47E-05 | 0 | 6.62E-05 | 0 |
| k__Bacteria;p__Cyanobacteria;c__Chloroplast;o__Cryptophyta;f__;g__ | 1.39E-05 | 0 | 0.0009744 | 0.00279832 | 0.01877092 | 0.02176876 | 0.00097895 |
| k__Bacteria;p__Cyanobacteria;c__Chloroplast;o__Haptophyceae;f__;g__ | 2.79E-05 | 7.12E-05 | 0.00108266 | 0.00572918 | 0.03634625 | 0.0497704 | 0.00195791 |
| k__Bacteria;p__Cyanobacteria;c__Chloroplast;o__Rhodophyta;f__;g__ | 0 | 0 | 0 | 1.47E-05 | 0 | 0 | 0 |
| k__Bacteria;p__Cyanobacteria;c__Chloroplast;o__Stramenopiles;f__;g__ | 0.0008782 | 0.00049849 | 0.18226601 | 0.05580429 | 0.0430715 | 0.04331256 | 0.007128 |
| k__Bacteria;p__Cyanobacteria;c__Chloroplast;o__Streptophyta;f__;g__ | 0 | 5.70E-05 | 0 | 0.00022092 | 5.98E-05 | 6.62E-05 | 0 |
| k__Bacteria;p__Cyanobacteria;c__Nostocophycideae;o__Nostocales;f__Nostocaceae;g__ | 0 | 0 | 0 | 0 | 0 | 0 | 0 |
| k__Bacteria;p__Cyanobacteria;c__Nostocophycideae;o__Stigonematales;f__Rivulariaceae;g__Calothrix | 0 | 0 | 0 | 0 | 0 | 0 | 0 |
| k__Bacteria;p__Cyanobacteria;c__Oscillatoriophycideae;o__Chroococcales;f__Cyanobacteriaceae;g__ | 0 | 0 | 0 | 2.95E-05 | 0.00050813 | 0.0004367 | 0.00052007 |
| k__Bacteria;p__Cyanobacteria;c__Oscillatoriophycideae;o__Chroococcales;f__Microcystaceae;g__Microcystis | 0 | 9.97E-05 | 0 | 0 | 0 | 0 | 0 |
| k__Bacteria;p__Cyanobacteria;c__Oscillatoriophycideae;o__Chroococcales;f__Xenococcaceae;g__ | 0 | 4.27E-05 | 0 | 8.84E-05 | 0 | 3.97E-05 | 0 |
| k__Bacteria;p__Cyanobacteria;c__Synechococcophycideae;o__Pseudanabaenales;f__Pseudanabaenaceae;g__ | 0 | 2.85E-05 | 0 | 0 | 2.99E-05 | 2.65E-05 | 0 |
| k__Bacteria;p__Cyanobacteria;c__Synechococcophycideae;o__Pseudanabaenales;f__Pseudanabaenaceae;g__Pseudanabaena | 0 | 0 | 0 | 0 | 0 | 0 | 0 |
| k__Bacteria;p__Cyanobacteria;c__Synechococcophycideae;o__Synechococcales;f__Synechococcaceae;g__Prochlorococcus | 0 | 0 | 0 | 0 | 0 | 0 | 0 |
| k__Bacteria;p__Cyanobacteria;c__Synechococcophycideae;o__Synechococcales;f__Synechococcaceae;g__Synechococcus | 0.00011152 | 0.00025637 | 0.00328858 | 0.00848331 | 0.01551291 | 0.02048513 | 0.06032795 |
| k__Bacteria;p__FBP;c__;o__;f__;g__ | 0 | 0 | 0 | 0 | 0 | 5.29E-05 | 0 |
| k__Bacteria;p__Fibrobacteres;c__Fibrobacteria;o__Fibrobacterales;f__;g__ | 0 | 0 | 0.00041953 | 0.00039766 | 2.99E-05 | 0 | 0 |
| k__Bacteria;p__Firmicutes;c__Bacilli;o__Bacillales;f__;g__ | 0 | 0 | 0 | 0 | 0 | 0 | 0 |
| k__Bacteria;p__Firmicutes;c__Bacilli;o__Bacillales;f__Bacillaceae;g__Anoxybacillus | 0 | 0 | 0 | 0 | 0 | 0 | 0 |
| k__Bacteria;p__Firmicutes;c__Bacilli;o__Bacillales;f__Bacillaceae;g__Bacillus | 2.79E-05 | 0 | 0 | 0 | 0 | 0 | 0 |
| k__Bacteria;p__Firmicutes;c__Bacilli;o__Bacillales;f__Bacillaceae;g__Geobacillus | 0 | 0 | 0 | 0 | 0 | 0 | 0 |
| k__Bacteria;p__Firmicutes;c__Bacilli;o__Bacillales;f__Bacillaceae;g__Marinococcus | 0 | 0 | 0 | 0 | 0 | 0 | 0 |
| k__Bacteria;p__Firmicutes;c__Bacilli;o__Bacillales;f__Listeriaceae;g__ | 0 | 0 | 0 | 0 | 2.99E-05 | 0 | 0 |
| k__Bacteria;p__Firmicutes;c__Bacilli;o__Bacillales;f__Listeriaceae;g__Brochothrix | 0 | 0 | 0 | 0 | 0 | 0 | 0 |

| Taxon | | 2028 | 2029 | 1184 | 1196 | 1245 | 1246 | 2019 |
|---|---|---|---|---|---|---|---|---|
| | Copepod genus | Centropages | Centropages | Water | Water | Water | Water | Water |
| | Type of sample | Starved | Starved | Water | Water | Water | Water | Water |
| k__Bacteria;p__Firmicutes;c__Bacilli;o__Bacillales;f__Paenibacillaceae;g__Paenibacillus | | 0 | 0 | 0 | 0 | 0 | 5.29E-05 | 0 |
| k__Bacteria;p__Firmicutes;c__Bacilli;o__Bacillales;f__Planococcaceae;g__ | | 0 | 0 | 0 | 0 | 0 | 0 | 0 |
| k__Bacteria;p__Firmicutes;c__Bacilli;o__Bacillales;f__Planococcaceae;g__Bacillus | | 0 | 0 | 0 | 0 | 0 | 0 | 0 |
| k__Bacteria;p__Firmicutes;c__Bacilli;o__Bacillales;f__Planococcaceae;g__Planococcus | | 0 | 0 | 0 | 0 | 0 | 0 | 0 |
| k__Bacteria;p__Firmicutes;c__Bacilli;o__Bacillales;f__Planococcaceae;g__Planomicrobium | | 0 | 0 | 0 | 0 | 0 | 0 | 0 |
| k__Bacteria;p__Firmicutes;c__Bacilli;o__Bacillales;f__Staphylococcaceae;g__Jeotgalicoccus | | 0 | 0 | 0 | 0 | 0 | 0 | 0 |
| k__Bacteria;p__Firmicutes;c__Bacilli;o__Bacillales;f__Staphylococcaceae;g__Staphylococcus | | 0 | 4.27E-05 | 0 | 0 | 0 | 1.32E-05 | 0 |
| k__Bacteria;p__Firmicutes;c__Bacilli;o__Bacillales;f__Thermoactinomycetaceae;g__ | | 0 | 0 | 0 | 0 | 0 | 0 | 0 |
| k__Bacteria;p__Firmicutes;c__Bacilli;o__Bacillales;f__Thermoactinomycetaceae;g__Planifilum | | 0 | 0 | 0 | 0 | 0 | 0 | 0 |
| k__Bacteria;p__Firmicutes;c__Bacilli;o__Bacillales;f__[Exiguobacteraceae];g__Exiguobacterium | | 0 | 0 | 0 | 0 | 0 | 0 | 0 |
| k__Bacteria;p__Firmicutes;c__Bacilli;o__Bacillales;f__[Thermicanaceae];g__Thermicanus | | 0 | 0 | 0 | 0 | 0 | 0 | 0 |
| k__Bacteria;p__Firmicutes;c__Bacilli;o__Gemellales;f__Gemellaceae;g__ | | 1.39E-05 | 1.42E-05 | 0 | 0 | 0 | 0 | 0 |
| k__Bacteria;p__Firmicutes;c__Bacilli;o__Gemellales;f__Gemellaceae;g__Gemella | | 0 | 0 | 0 | 0 | 0 | 0 | 0 |
| k__Bacteria;p__Firmicutes;c__Bacilli;o__Lactobacillales;f__Aerococcaceae;g__ | | 0 | 0 | 0 | 0 | 0 | 0 | 0 |
| k__Bacteria;p__Firmicutes;c__Bacilli;o__Lactobacillales;f__Aerococcaceae;g__Aerococcus | | 0 | 0 | 0 | 0 | 0 | 0 | 0 |
| k__Bacteria;p__Firmicutes;c__Bacilli;o__Lactobacillales;f__Aerococcaceae;g__Alloiococcus | | 0 | 0 | 0 | 0 | 0 | 0 | 0 |
| k__Bacteria;p__Firmicutes;c__Bacilli;o__Lactobacillales;f__Aerococcaceae;g__Facklamia | | 0 | 0 | 0 | 0 | 0 | 0 | 0 |
| k__Bacteria;p__Firmicutes;c__Bacilli;o__Lactobacillales;f__Aerococcaceae;g__Marinilactibacillus | | 0 | 0 | 0 | 0 | 0 | 0 | 0 |
| k__Bacteria;p__Firmicutes;c__Bacilli;o__Lactobacillales;f__Carnobacteriaceae;g__ | | 0 | 0 | 0 | 0 | 0 | 0 | 0 |
| k__Bacteria;p__Firmicutes;c__Bacilli;o__Lactobacillales;f__Carnobacteriaceae;g__Granulicatella | | 0 | 0 | 0 | 0 | 0 | 0 | 0 |
| k__Bacteria;p__Firmicutes;c__Bacilli;o__Lactobacillales;f__Carnobacteriaceae;g__Trichococcus | | 0 | 0 | 0 | 0 | 0 | 1.32E-05 | 0 |
| k__Bacteria;p__Firmicutes;c__Bacilli;o__Lactobacillales;f__Enterococcaceae;g__Enterococcus | | 0 | 0 | 0 | 1.47E-05 | 0 | 5.29E-05 | 0 |
| k__Bacteria;p__Firmicutes;c__Bacilli;o__Lactobacillales;f__Lactobacillaceae;g__Lactobacillus | | 0 | 0 | 0 | 0 | 0 | 0 | 0 |
| k__Bacteria;p__Firmicutes;c__Bacilli;o__Lactobacillales;f__Leuconostocaceae;g__ | | 0 | 0 | 0 | 0 | 0 | 0 | 0 |
| k__Bacteria;p__Firmicutes;c__Bacilli;o__Lactobacillales;f__Leuconostocaceae;g__Leuconostoc | | 0 | 0 | 0 | 0 | 0 | 0 | 0 |
| k__Bacteria;p__Firmicutes;c__Bacilli;o__Lactobacillales;f__Leuconostocaceae;g__Weissella | | 0 | 0 | 0 | 0 | 0 | 0 | 0 |
| k__Bacteria;p__Firmicutes;c__Bacilli;o__Lactobacillales;f__Streptococcaceae;g__Lactococcus | | 0 | 0 | 0 | 0 | 0 | 0 | 0 |
| k__Bacteria;p__Firmicutes;c__Bacilli;o__Lactobacillales;f__Streptococcaceae;g__Streptococcus | | 0 | 7.12E-05 | 0 | 0 | 2.99E-05 | 1.32E-05 | 0 |
| k__Bacteria;p__Firmicutes;c__Clostridia;o__Clostridiales;f__;g__ | | 0 | 0 | 0 | 0 | 0 | 2.65E-05 | 0 |
| k__Bacteria;p__Firmicutes;c__Clostridia;o__Clostridiales;f__Clostridiaceae;g__ | | 0 | 0 | 0 | 0 | 0 | 6.62E-05 | 0 |
| k__Bacteria;p__Firmicutes;c__Clostridia;o__Clostridiales;f__Clostridiaceae;g__Clostridium | | 0 | 0 | 1.35E-05 | 0 | 0 | 1.32E-05 | 0 |
| k__Bacteria;p__Firmicutes;c__Clostridia;o__Clostridiales;f__Lachnospiraceae;g__ | | 0 | 0 | 0 | 0 | 2.99E-05 | 5.29E-05 | 0 |
| k__Bacteria;p__Firmicutes;c__Clostridia;o__Clostridiales;f__Lachnospiraceae;g__Blautia | | 0 | 0 | 0 | 0 | 0 | 0 | 0 |
| k__Bacteria;p__Firmicutes;c__Clostridia;o__Clostridiales;f__Lachnospiraceae;g__Dorea | | 0 | 0 | 0 | 0 | 0 | 0 | 0 |
| k__Bacteria;p__Firmicutes;c__Clostridia;o__Clostridiales;f__Lachnospiraceae;g__Moryella | | 0 | 0 | 0 | 0 | 0 | 0 | 0 |
| k__Bacteria;p__Firmicutes;c__Clostridia;o__Clostridiales;f__Lachnospiraceae;g__Oribacterium | | 0 | 0 | 0 | 0 | 0 | 0 | 0 |
| k__Bacteria;p__Firmicutes;c__Clostridia;o__Clostridiales;f__Lachnospiraceae;g__[Ruminococcus] | | 0 | 0 | 0 | 0 | 5.98E-05 | 0 | 0 |
| k__Bacteria;p__Firmicutes;c__Clostridia;o__Clostridiales;f__Peptostreptococcaceae;g__ | | 0 | 0 | 0 | 0 | 2.99E-05 | 1.32E-05 | 0 |
| k__Bacteria;p__Firmicutes;c__Clostridia;o__Clostridiales;f__Ruminococcaceae;g__ | | 0 | 0 | 0 | 0 | 0.00014945 | 6.62E-05 | 0 |
| k__Bacteria;p__Firmicutes;c__Clostridia;o__Clostridiales;f__Ruminococcaceae;g__Clostridium | | 0 | 0 | 0 | 0 | 0 | 0 | 0 |
| k__Bacteria;p__Firmicutes;c__Clostridia;o__Clostridiales;f__Ruminococcaceae;g__Faecalibacterium | | 0 | 0 | 0 | 0 | 0 | 0.00010587 | 0 |
| k__Bacteria;p__Firmicutes;c__Clostridia;o__Clostridiales;f__Ruminococcaceae;g__Oscillospira | | 0 | 0 | 0 | 0 | 0 | 1.32E-05 | 0 |
| k__Bacteria;p__Firmicutes;c__Clostridia;o__Clostridiales;f__Veillonellaceae;g__ | | 0 | 0 | 0 | 0 | 0 | 6.62E-05 | 0 |
| k__Bacteria;p__Firmicutes;c__Clostridia;o__Clostridiales;f__Veillonellaceae;g__Anaeromusa | | 0 | 0 | 0 | 0 | 5.98E-05 | 3.97E-05 | 0 |
| k__Bacteria;p__Firmicutes;c__Clostridia;o__Clostridiales;f__Veillonellaceae;g__Dialister | | 0 | 0 | 0 | 0 | 0 | 0 | 0 |
| k__Bacteria;p__Firmicutes;c__Clostridia;o__Clostridiales;f__Veillonellaceae;g__Pelosinus | | 0 | 0 | 0 | 0 | 0 | 1.32E-05 | 0 |
| k__Bacteria;p__Firmicutes;c__Clostridia;o__Clostridiales;f__Veillonellaceae;g__Phascolarctobacterium | | 0 | 0 | 0 | 0 | 2.99E-05 | 1.32E-05 | 0 |
| k__Bacteria;p__Firmicutes;c__Clostridia;o__Clostridiales;f__Veillonellaceae;g__Selenomonas | | 0 | 0 | 0 | 0 | 0 | 0 | 0 |
| k__Bacteria;p__Firmicutes;c__Clostridia;o__Clostridiales;f__Veillonellaceae;g__Veillonella | | 0 | 0 | 0 | 0 | 0 | 0 | 0 |
| k__Bacteria;p__Firmicutes;c__Clostridia;o__Clostridiales;f__[Acidaminobacteraceae];g__Fusibacter | | 0 | 0 | 0 | 0 | 2.99E-05 | 1.32E-05 | 0 |
| k__Bacteria;p__Firmicutes;c__Clostridia;o__Clostridiales;f__[Acidaminobacteraceae];g__WH1-8 | | 0 | 0 | 0 | 1.47E-05 | 0 | 2.65E-05 | 0 |
| k__Bacteria;p__Firmicutes;c__Clostridia;o__Clostridiales;f__[Mogibacteriaceae];g__ | | 0 | 0 | 0 | 0 | 0 | 0 | 0 |
| k__Bacteria;p__Firmicutes;c__Clostridia;o__Clostridiales;f__[Mogibacteriaceae];g__Mogibacterium | | 0 | 0 | 0 | 0 | 0 | 0 | 0 |
| k__Bacteria;p__Firmicutes;c__Clostridia;o__Clostridiales;f__[Tissierellaceae];g__Anaerococcus | | 0 | 0 | 0 | 0 | 0 | 0 | 0 |
| k__Bacteria;p__Firmicutes;c__Clostridia;o__Clostridiales;f__[Tissierellaceae];g__Finegoldia | | 0 | 0 | 0 | 0 | 0 | 0 | 0 |
| k__Bacteria;p__Firmicutes;c__Clostridia;o__Clostridiales;f__[Tissierellaceae];g__Gallicola | | 0 | 0 | 0 | 0 | 0 | 0 | 0 |
| k__Bacteria;p__Firmicutes;c__Clostridia;o__Clostridiales;f__[Tissierellaceae];g__Parvimonas | | 0 | 0 | 0 | 0 | 0 | 0 | 0 |
| k__Bacteria;p__Firmicutes;c__Clostridia;o__Clostridiales;f__[Tissierellaceae];g__Peptoniphilus | | 0 | 0 | 0 | 0 | 0 | 0 | 0 |
| k__Bacteria;p__Firmicutes;c__Clostridia;o__Clostridiales;f__[Tissierellaceae];g__WAL_1855D | | 0 | 0 | 0 | 0 | 0 | 0 | 0 |
| k__Bacteria;p__Firmicutes;c__Clostridia;o__Thermoanaerobacterales;f__Caldicellulosiruptoraceae;g__Caldicellulosiruptor | | 0 | 0 | 0 | 0 | 0 | 0 | 0 |
| k__Bacteria;p__Firmicutes;c__Erysipelotrichi;o__Erysipelotrichales;f__Erysipelotrichaceae;g__ | | 0 | 0 | 0 | 0 | 0 | 0 | 0 |
| k__Bacteria;p__Firmicutes;c__Erysipelotrichi;o__Erysipelotrichales;f__Erysipelotrichaceae;g__Coprobacillus | | 0 | 0 | 0 | 0 | 0 | 0 | 0 |
| k__Bacteria;p__Firmicutes;c__Erysipelotrichi;o__Erysipelotrichales;f__Erysipelotrichaceae;g__RFN20 | | 0 | 0 | 0 | 0 | 0 | 0 | 0 |
| k__Bacteria;p__Firmicutes;c__Erysipelotrichi;o__Erysipelotrichales;f__Erysipelotrichaceae;g__[Eubacterium] | | 0 | 0 | 0 | 0 | 0 | 0 | 0 |
| k__Bacteria;p__Fusobacteria;c__Fusobacteriia;o__Fusobacteriales;f__;g__ | | 0 | 0 | 0 | 0 | 8.97E-05 | 0.00017203 | 0 |
| k__Bacteria;p__Fusobacteria;c__Fusobacteriia;o__Fusobacteriales;f__Fusobacteriaceae;g__Cetobacterium | | 0 | 0 | 0 | 2.95E-05 | 0 | 0 | 0 |
| k__Bacteria;p__Fusobacteria;c__Fusobacteriia;o__Fusobacteriales;f__Fusobacteriaceae;g__Fusobacterium | | 0 | 4.27E-05 | 0 | 2.95E-05 | 0 | 3.97E-05 | 0 |
| k__Bacteria;p__Fusobacteria;c__Fusobacteriia;o__Fusobacteriales;f__Fusobacteriaceae;g__Propionigenium | | 0 | 0 | 0 | 0 | 0 | 2.65E-05 | 0 |
| k__Bacteria;p__Fusobacteria;c__Fusobacteriia;o__Fusobacteriales;f__Fusobacteriaceae;g__Psychrilyobacter | | 0 | 0 | 0 | 4.42E-05 | 2.99E-05 | 7.94E-05 | 0 |
| k__Bacteria;p__Fusobacteria;c__Fusobacteriia;o__Fusobacteriales;f__Fusobacteriaceae;g__u114 | | 0 | 0 | 0 | 0 | 2.99E-05 | 6.62E-05 | 0 |
| k__Bacteria;p__Fusobacteria;c__Fusobacteriia;o__Fusobacteriales;f__Leptotrichiaceae;g__Leptotrichia | | 0 | 1.42E-05 | 0 | 0 | 5.98E-05 | 3.97E-05 | 3.06E-05 |
| k__Bacteria;p__GN02;c__;o__;f__;g__ | | 0 | 0 | 0 | 1.47E-05 | 2.99E-05 | 0 | 0 |
| k__Bacteria;p__GN02;c__BD1-5;o__;f__;g__ | | 0.01208564 | 0.01807383 | 1.35E-05 | 0.00013255 | 8.97E-05 | 9.26E-05 | 3.06E-05 |
| k__Bacteria;p__GN02;c__IIB17;o__;f__;g__ | | 0 | 0 | 0 | 1.47E-05 | 0 | 0 | 0 |

| Taxon | | 2028 | 2029 | 1184 | 1196 | 1245 | 1246 | 2019 |
|---|---|---|---|---|---|---|---|---|
| | Copepod genus | Centropages | Centropages | Water | Water | Water | Water | Water |
| | Type of sample | Starved | Starved | Water | Water | Water | Water | Water |
| k__Bacteria;p__Gemmatimonadetes;c__Gemmatimonadetes;o__;f__;g__ | | 0 | 0 | 0 | 0 | 0 | 0 | 0 |
| k__Bacteria;p__Gemmatimonadetes;c__Gemmatimonadetes;o__Gemmatimonadales;f__;g__ | | 0 | 0 | 0 | 0 | 0 | 0 | 0 |
| k__Bacteria;p__Lentisphaerae;c__[Lentisphaeria];o__Lentisphaerales;f__;g__ | | 0 | 0 | 0 | 0 | 2.99E-05 | 0 | 0 |
| k__Bacteria;p__Lentisphaerae;c__[Lentisphaeria];o__Lentisphaerales;f__Lentisphaeraceae;g__ | | 0 | 0 | 0 | 1.47E-05 | 0 | 0 | 0 |
| k__Bacteria;p__Lentisphaerae;c__[Lentisphaeria];o__Lentisphaerales;f__Lentisphaeraceae;g__Lentisphaera | | 0 | 0 | 0 | 0 | 0 | 0 | 0 |
| k__Bacteria;p__Lentisphaerae;c__[Lentisphaeria];o__Victivallales;f__Victivallaceae;g__ | | 0 | 0 | 0 | 0 | 0 | 1.32E-05 | 0 |
| k__Bacteria;p__NKB19;c__SHAB590;o__;f__;g__ | | 0 | 0 | 0 | 0 | 2.99E-05 | 0 | 0 |
| k__Bacteria;p__Nitrospirae;c__Nitrospira;o__Nitrospirales;f__Nitrospiraceae;g__ | | 0 | 0 | 0 | 0 | 0 | 1.32E-05 | 0 |
| k__Bacteria;p__OD1;c__;o__;f__;g__ | | 0 | 0 | 0 | 0 | 0 | 0 | 0 |
| k__Bacteria;p__OD1;c__ABY1;o__;f__;g__ | | 0 | 0 | 0 | 0 | 0 | 1.32E-05 | 0 |
| k__Bacteria;p__OD1;c__SM2F11;o__;f__;g__ | | 0 | 0 | 0 | 0 | 0 | 0 | 0 |
| k__Bacteria;p__OD1;c__ZB2;o__;f__;g__ | | 0 | 0 | 0 | 4.42E-05 | 5.98E-05 | 3.97E-05 | 0 |
| k__Bacteria;p__OP8;c__OP8_1;o__HMMVPog-54;f__;g__ | | 0 | 0 | 0 | 0 | 5.98E-05 | 0 | 0 |
| k__Bacteria;p__Planctomycetes;c__OM190;o__CL500-15;f__;g__ | | 0 | 0 | 2.71E-05 | 0.00013255 | 0 | 2.65E-05 | 3.06E-05 |
| k__Bacteria;p__Planctomycetes;c__OM190;o__agg27;f__;g__ | | 0 | 0 | 0.00043306 | 0.0022092 | 0.00062769 | 0.00054256 | 0.00091777 |
| k__Bacteria;p__Planctomycetes;c__Phycisphaerae;o__Phycisphaerales;f__;g__ | | 4.18E-05 | 2.85E-05 | 0.00039246 | 0.00296032 | 0.0005978 | 0.00041023 | 0.00107073 |
| k__Bacteria;p__Planctomycetes;c__Phycisphaerae;o__Phycisphaerales;f__Phycisphaeraceae;g__ | | 0 | 0 | 0 | 0 | 0 | 0 | 0 |
| k__Bacteria;p__Planctomycetes;c__Planctomycetia;o__Gemmatales;f__Gemmataceae;g__ | | 0 | 0 | 0 | 0 | 0 | 2.65E-05 | 0 |
| k__Bacteria;p__Planctomycetes;c__Planctomycetia;o__Gemmatales;f__Isosphaeraceae;g__ | | 0 | 0 | 0 | 0 | 0 | 2.65E-05 | 0 |
| k__Bacteria;p__Planctomycetes;c__Planctomycetia;o__Pirellulales;f__Pirellulaceae;g__ | | 6.97E-05 | 0.00015667 | 0.00331565 | 0.0176883 | 0.00454328 | 0.00346712 | 0.00333456 |
| k__Bacteria;p__Planctomycetes;c__Planctomycetia;o__Pirellulales;f__Pirellulaceae;g__Planctomycete | | 0 | 0 | 0 | 1.47E-05 | 0 | 0 | 0 |
| k__Bacteria;p__Planctomycetes;c__Planctomycetia;o__Pirellulales;f__Pirellulaceae;g__Rhodopirellula | | 0 | 0 | 0 | 0 | 0 | 0 | 0 |
| k__Bacteria;p__Planctomycetes;c__Planctomycetia;o__Planctomycetales;f__Planctomycetaceae;g__Planctomyces | | 0 | 1.42E-05 | 5.41E-05 | 2.95E-05 | 0.0002989 | 0.00017203 | 3.06E-05 |
| k__Bacteria;p__Planctomycetes;c__vadinHA49;o__;f__;g__ | | 0 | 0 | 0 | 0 | 0 | 0 | 0 |
| k__Bacteria;p__Proteobacteria;c__Alphaproteobacteria;o__;f__;g__ | | 0.00269035 | 0.00618128 | 0.01789098 | 0.01757047 | 0.03281923 | 0.03145553 | 0.05524963 |
| k__Bacteria;p__Proteobacteria;c__Alphaproteobacteria;o__BD7-3;f__;g__ | | 0.0001394 | 5.70E-05 | 0 | 2.95E-05 | 5.98E-05 | 0 | 0 |
| k__Bacteria;p__Proteobacteria;c__Alphaproteobacteria;o__Caulobacterales;f__Caulobacteraceae;g__ | | 0 | 0 | 0 | 0 | 0 | 1.32E-05 | 0 |
| k__Bacteria;p__Proteobacteria;c__Alphaproteobacteria;o__Caulobacterales;f__Caulobacteraceae;g__Brevundimonas | | 0 | 0 | 0 | 0 | 0 | 0 | 0 |
| k__Bacteria;p__Proteobacteria;c__Alphaproteobacteria;o__Caulobacterales;f__Caulobacteraceae;g__Mycoplana | | 0 | 0 | 0 | 0 | 0 | 0 | 0 |
| k__Bacteria;p__Proteobacteria;c__Alphaproteobacteria;o__Caulobacterales;f__Caulobacteraceae;g__Phenylobacterium | | 2.79E-05 | 5.70E-05 | 0 | 0 | 5.98E-05 | 0 | 0 |
| k__Bacteria;p__Proteobacteria;c__Alphaproteobacteria;o__Ellin329;f__;g__ | | 0 | 0 | 0 | 2.95E-05 | 2.99E-05 | 0 | 0 |
| k__Bacteria;p__Proteobacteria;c__Alphaproteobacteria;o__Kiloniellales;f__;g__ | | 0 | 0 | 0 | 0.0001031 | 0.0002989 | 0.0003176 | 9.18E-05 |
| k__Bacteria;p__Proteobacteria;c__Alphaproteobacteria;o__Kiloniellales;f__Kiloniellaceae;g__ | | 2.79E-05 | 4.27E-05 | 0.00232772 | 0.00078058 | 0.00038857 | 0.0003176 | 0.0011931 |
| k__Bacteria;p__Proteobacteria;c__Alphaproteobacteria;o__Kiloniellales;f__Kiloniellaceae;g__Thalassospira | | 0 | 0 | 0 | 0 | 0 | 0 | 0 |
| k__Bacteria;p__Proteobacteria;c__Alphaproteobacteria;o__Kordiimonadales;f__Kordiimonadaceae;g__ | | 0 | 0 | 0 | 0 | 0 | 0 | 0 |
| k__Bacteria;p__Proteobacteria;c__Alphaproteobacteria;o__Rhizobiales;f__;g__ | | 5.58E-05 | 0.00014243 | 0.00050073 | 0.00011782 | 0.0002989 | 0.00014557 | 0.00012237 |
| k__Bacteria;p__Proteobacteria;c__Alphaproteobacteria;o__Rhizobiales;f__Aurantimonadaceae;g__Martelella | | 0 | 0 | 0 | 1.47E-05 | 0 | 0 | 0 |
| k__Bacteria;p__Proteobacteria;c__Alphaproteobacteria;o__Rhizobiales;f__Beijerinckiaceae;g__Chelatococcus | | 0 | 0 | 0 | 0 | 0 | 0 | 0 |
| k__Bacteria;p__Proteobacteria;c__Alphaproteobacteria;o__Rhizobiales;f__Bradyrhizobiaceae;g__ | | 1.39E-05 | 0 | 0 | 0 | 0 | 1.32E-05 | 0 |
| k__Bacteria;p__Proteobacteria;c__Alphaproteobacteria;o__Rhizobiales;f__Bradyrhizobiaceae;g__Balneimonas | | 0 | 0 | 0 | 0 | 0 | 0 | 0 |
| k__Bacteria;p__Proteobacteria;c__Alphaproteobacteria;o__Rhizobiales;f__Bradyrhizobiaceae;g__Pseudomonas | | 0 | 0 | 0 | 0 | 0 | 0 | 0 |
| k__Bacteria;p__Proteobacteria;c__Alphaproteobacteria;o__Rhizobiales;f__Cohaesibacteraceae;g__Cohaesibacter | | 0 | 0 | 0 | 0 | 0 | 0 | 0 |
| k__Bacteria;p__Proteobacteria;c__Alphaproteobacteria;o__Rhizobiales;f__Hyphomicrobiaceae;g__ | | 0 | 0 | 4.06E-05 | 0.00022092 | 0.00014945 | 7.94E-05 | 9.18E-05 |
| k__Bacteria;p__Proteobacteria;c__Alphaproteobacteria;o__Rhizobiales;f__Hyphomicrobiaceae;g__Devosia | | 0.0001394 | 5.70E-05 | 0 | 1.47E-05 | 2.99E-05 | 3.97E-05 | 0 |
| k__Bacteria;p__Proteobacteria;c__Alphaproteobacteria;o__Rhizobiales;f__Hyphomicrobiaceae;g__Rhodoplanes | | 0 | 0 | 0 | 0 | 0 | 0 | 0 |
| k__Bacteria;p__Proteobacteria;c__Alphaproteobacteria;o__Rhizobiales;f__Methylobacteriaceae;g__ | | 0 | 0 | 0 | 0 | 0 | 0 | 0 |
| k__Bacteria;p__Proteobacteria;c__Alphaproteobacteria;o__Rhizobiales;f__Methylobacteriaceae;g__Methylobacterium | | 0 | 2.85E-05 | 0 | 0 | 0 | 0 | 0 |
| k__Bacteria;p__Proteobacteria;c__Alphaproteobacteria;o__Rhizobiales;f__Methylocystaceae;g__ | | 0 | 0 | 0 | 0 | 0 | 0 | 6.12E-05 |
| k__Bacteria;p__Proteobacteria;c__Alphaproteobacteria;o__Rhizobiales;f__Methylocystaceae;g__Methylosinus | | 0 | 0 | 0 | 0 | 0 | 0 | 3.06E-05 |
| k__Bacteria;p__Proteobacteria;c__Alphaproteobacteria;o__Rhizobiales;f__Methylocystaceae;g__Pleomorphomonas | | 0 | 0 | 0 | 0 | 0 | 0 | 0 |
| k__Bacteria;p__Proteobacteria;c__Alphaproteobacteria;o__Rhizobiales;f__Phyllobacteriaceae;g__ | | 1.39E-05 | 0 | 0 | 0.00017674 | 0.00014945 | 0 | 0 |
| k__Bacteria;p__Proteobacteria;c__Alphaproteobacteria;o__Rhizobiales;f__Phyllobacteriaceae;g__Chelativorans | | 0 | 0 | 0 | 0 | 0 | 0 | 0 |
| k__Bacteria;p__Proteobacteria;c__Alphaproteobacteria;o__Rhizobiales;f__Phyllobacteriaceae;g__Hoeflea | | 1.39E-05 | 7.12E-05 | 0 | 0 | 0 | 0 | 0 |
| k__Bacteria;p__Proteobacteria;c__Alphaproteobacteria;o__Rhizobiales;f__Phyllobacteriaceae;g__Mesorhizobium | | 0 | 0 | 0 | 0 | 0 | 0 | 0 |
| k__Bacteria;p__Proteobacteria;c__Alphaproteobacteria;o__Rhizobiales;f__Phyllobacteriaceae;g__Phyllobacterium | | 0 | 0 | 0 | 0 | 0 | 0 | 0 |
| k__Bacteria;p__Proteobacteria;c__Alphaproteobacteria;o__Rhizobiales;f__Rhizobiaceae;g__ | | 0 | 0 | 0 | 0 | 0 | 0 | 0 |
| k__Bacteria;p__Proteobacteria;c__Alphaproteobacteria;o__Rhizobiales;f__Rhizobiaceae;g__Agrobacterium | | 0 | 0 | 0 | 0 | 0 | 2.65E-05 | 0 |
| k__Bacteria;p__Proteobacteria;c__Alphaproteobacteria;o__Rhizobiales;f__Rhizobiaceae;g__Rhizobium | | 0 | 0 | 0 | 0 | 0 | 0 | 0 |
| k__Bacteria;p__Proteobacteria;c__Alphaproteobacteria;o__Rhizobiales;f__Rhizobiaceae;g__Shinella | | 0 | 0 | 0 | 0 | 0 | 0 | 0 |
| k__Bacteria;p__Proteobacteria;c__Alphaproteobacteria;o__Rhizobiales;f__Xanthobacteraceae;g__Xanthobacter | | 0 | 0 | 0 | 0 | 0 | 0 | 0 |
| k__Bacteria;p__Proteobacteria;c__Alphaproteobacteria;o__Rhodobacterales;f__Hyphomonadaceae;g__ | | 4.18E-05 | 8.55E-05 | 0.00104206 | 0.00231229 | 0.00322812 | 0.00352006 | 0.00379344 |
| k__Bacteria;p__Proteobacteria;c__Alphaproteobacteria;o__Rhodobacterales;f__Hyphomonadaceae;g__Maricaulis | | 2.79E-05 | 4.27E-05 | 0 | 0 | 0 | 0 | 0 |
| k__Bacteria;p__Proteobacteria;c__Alphaproteobacteria;o__Rhodobacterales;f__Hyphomonadaceae;g__Robiginitomaculum | | 0 | 0 | 0 | 0 | 0 | 3.97E-05 | 0 |
| k__Bacteria;p__Proteobacteria;c__Alphaproteobacteria;o__Rhodobacterales;f__Rhodobacteraceae;g__ | | 0.0532772 | 0.0684356 | 0.09063227 | 0.09568765 | 0.09433286 | 0.08384612 | 0.06555923 |
| k__Bacteria;p__Proteobacteria;c__Alphaproteobacteria;o__Rhodobacterales;f__Rhodobacteraceae;g__Amaricoccus | | 0 | 0 | 0 | 0 | 0 | 0 | 0 |
| k__Bacteria;p__Proteobacteria;c__Alphaproteobacteria;o__Rhodobacterales;f__Rhodobacteraceae;g__Anaerospora | | 1.39E-05 | 0 | 0 | 5.89E-05 | 0.00062769 | 0.000397 | 0 |
| k__Bacteria;p__Proteobacteria;c__Alphaproteobacteria;o__Rhodobacterales;f__Rhodobacteraceae;g__Celeribacter | | 0 | 0 | 1.35E-05 | 8.84E-05 | 0 | 0 | 0 |
| k__Bacteria;p__Proteobacteria;c__Alphaproteobacteria;o__Rhodobacterales;f__Rhodobacteraceae;g__Dinoroseobacter | | 9.76E-05 | 0.00051273 | 0 | 0.00011782 | 0.00047824 | 0.00033083 | 6.12E-05 |
| k__Bacteria;p__Proteobacteria;c__Alphaproteobacteria;o__Rhodobacterales;f__Rhodobacteraceae;g__Donghicola | | 0 | 1.42E-05 | 0 | 0 | 0 | 0 | 0 |
| k__Bacteria;p__Proteobacteria;c__Alphaproteobacteria;o__Rhodobacterales;f__Rhodobacteraceae;g__Jannaschia | | 1.39E-05 | 0 | 0 | 4.42E-05 | 0 | 0 | 0 |
| k__Bacteria;p__Proteobacteria;c__Alphaproteobacteria;o__Rhodobacterales;f__Rhodobacteraceae;g__Loktanella | | 0.00072486 | 0.0007691 | 0.00010827 | 0.0001031 | 0.00032879 | 0.00021173 | 0.00027533 |
| k__Bacteria;p__Proteobacteria;c__Alphaproteobacteria;o__Rhodobacterales;f__Rhodobacteraceae;g__Maribius | | 0 | 0 | 0 | 2.95E-05 | 0 | 0 | 0 |
| k__Bacteria;p__Proteobacteria;c__Alphaproteobacteria;o__Rhodobacterales;f__Rhodobacteraceae;g__Marivita | | 0 | 0 | 0 | 0 | 2.99E-05 | 3.97E-05 | 0 |
| k__Bacteria;p__Proteobacteria;c__Alphaproteobacteria;o__Rhodobacterales;f__Rhodobacteraceae;g__Nautella | | 0 | 0 | 0 | 0 | 0 | 0 | 0 |

| Taxon | | 2028 | 2029 | 1184 | 1196 | 1245 | 1246 | 2019 |
|---|---|---|---|---|---|---|---|---|
| | Copepod genus | Centropages | Centropages | Water | Water | Water | Water | Water |
| | Type of sample | Starved | Starved | Water | Water | Water | Water | Water |
| k__Bacteria;p__Proteobacteria;c__Alphaproteobacteria;o__Rhodobacterales;f__Rhodobacteraceae;g__Oceanicola | | 0 | 0 | 0 | 1.47E-05 | 0 | 0 | 0 |
| k__Bacteria;p__Proteobacteria;c__Alphaproteobacteria;o__Rhodobacterales;f__Rhodobacteraceae;g__Octadecabacter | | 0.02702891 | 0.05687062 | 0.03563309 | 0.06285899 | 0.21658297 | 0.15785991 | 0.0989048 |
| k__Bacteria;p__Proteobacteria;c__Alphaproteobacteria;o__Rhodobacterales;f__Rhodobacteraceae;g__Paracoccus | | 1.39E-05 | 0 | 0 | 0 | 0 | 1.32E-05 | 0 |
| k__Bacteria;p__Proteobacteria;c__Alphaproteobacteria;o__Rhodobacterales;f__Rhodobacteraceae;g__Phaeobacter | | 0.00165881 | 0.00132456 | 1.35E-05 | 2.95E-05 | 8.97E-05 | 0.00010587 | 0 |
| k__Bacteria;p__Proteobacteria;c__Alphaproteobacteria;o__Rhodobacterales;f__Rhodobacteraceae;g__Pseudoroseobacter | | 0.00022303 | 9.97E-05 | 0 | 4.42E-05 | 0 | 0 | 0 |
| k__Bacteria;p__Proteobacteria;c__Alphaproteobacteria;o__Rhodobacterales;f__Rhodobacteraceae;g__Pseudoruegeria | | 8.36E-05 | 0.00012818 | 0.0005278 | 0.00022092 | 0.00023912 | 0.0001191 | 0 |
| k__Bacteria;p__Proteobacteria;c__Alphaproteobacteria;o__Rhodobacterales;f__Rhodobacteraceae;g__Rhodobacter | | 0 | 0 | 2.71E-05 | 0 | 8.97E-05 | 7.94E-05 | 0 |
| k__Bacteria;p__Proteobacteria;c__Alphaproteobacteria;o__Rhodobacterales;f__Rhodobacteraceae;g__Roseicyclus | | 0 | 0 | 0 | 0 | 0 | 0 | 0 |
| k__Bacteria;p__Proteobacteria;c__Alphaproteobacteria;o__Rhodobacterales;f__Rhodobacteraceae;g__Roseobacter | | 0 | 0 | 0 | 1.47E-05 | 0 | 0 | 0 |
| k__Bacteria;p__Proteobacteria;c__Alphaproteobacteria;o__Rhodobacterales;f__Rhodobacteraceae;g__Rubellimicrobium | | 0 | 0 | 0 | 0 | 0 | 0 | 0 |
| k__Bacteria;p__Proteobacteria;c__Alphaproteobacteria;o__Rhodobacterales;f__Rhodobacteraceae;g__Shimia | | 0 | 0 | 0 | 0 | 0 | 0 | 0 |
| k__Bacteria;p__Proteobacteria;c__Alphaproteobacteria;o__Rhodobacterales;f__Rhodobacteraceae;g__Sulfitobacter | | 0.00182609 | 0.00457187 | 0 | 0 | 0 | 0 | 3.06E-05 |
| k__Bacteria;p__Proteobacteria;c__Alphaproteobacteria;o__Rhodobacterales;f__Rhodobacteraceae;g__Thalassobacter | | 0 | 0 | 1.35E-05 | 1.47E-05 | 0 | 0 | 6.12E-05 |
| k__Bacteria;p__Proteobacteria;c__Alphaproteobacteria;o__Rhodobacterales;f__Rhodobacteraceae;g__Thalassobius | | 0.00027879 | 0.00065516 | 0 | 0 | 0.00071736 | 0.00046317 | 9.18E-05 |
| k__Bacteria;p__Proteobacteria;c__Alphaproteobacteria;o__Rhodobacterales;f__Rhodobacteraceae;g__Tropicibacter | | 6.97E-05 | 5.70E-05 | 0 | 0 | 0 | 0 | 0 |
| k__Bacteria;p__Proteobacteria;c__Alphaproteobacteria;o__Rhodospirillales;f__;g__ | | 0 | 0 | 0 | 0 | 0 | 0 | 0 |
| k__Bacteria;p__Proteobacteria;c__Alphaproteobacteria;o__Rhodospirillales;f__Acetobacteraceae;g__ | | 0 | 0 | 0 | 0 | 0 | 0 | 0 |
| k__Bacteria;p__Proteobacteria;c__Alphaproteobacteria;o__Rhodospirillales;f__Acetobacteraceae;g__Roseococcus | | 0 | 0 | 0 | 0 | 0 | 0 | 0 |
| k__Bacteria;p__Proteobacteria;c__Alphaproteobacteria;o__Rhodospirillales;f__Rhodospirillaceae;g__ | | 0.00204912 | 0.00484248 | 0.00128566 | 0.00128133 | 0.0020923 | 0.00187913 | 0.00351811 |
| k__Bacteria;p__Proteobacteria;c__Alphaproteobacteria;o__Rhodospirillales;f__Rhodospirillaceae;g__Skermanella | | 0 | 0 | 0 | 0 | 0 | 0 | 0 |
| k__Bacteria;p__Proteobacteria;c__Alphaproteobacteria;o__Rickettsiales;f__;g__ | | 0.00054364 | 0.00069789 | 0.00320738 | 0.00334325 | 0.00080703 | 0.0011116 | 0.00403818 |
| k__Bacteria;p__Proteobacteria;c__Alphaproteobacteria;o__Rickettsiales;f__AEGEAN_112;g__ | | 2.79E-05 | 0 | 4.06E-05 | 5.89E-05 | 5.98E-05 | 0 | 0 |
| k__Bacteria;p__Proteobacteria;c__Alphaproteobacteria;o__Rickettsiales;f__Pelagibacteraceae;g__ | | 0.01201595 | 0.02441178 | 0.25584637 | 0.35207223 | 0.11223697 | 0.16365609 | 0.33048825 |
| k__Bacteria;p__Proteobacteria;c__Alphaproteobacteria;o__Rickettsiales;f__Rickettsiaceae;g__ | | 0 | 0 | 4.06E-05 | 2.95E-05 | 0.00014945 | 7.94E-05 | 0.00012237 |
| k__Bacteria;p__Proteobacteria;c__Alphaproteobacteria;o__Rickettsiales;f__mitochondria;g__ | | 0 | 0 | 0 | 7.36E-05 | 0 | 1.32E-05 | 0 |
| k__Bacteria;p__Proteobacteria;c__Alphaproteobacteria;o__Rickettsiales;f__mitochondria;g__Carludovica | | 0 | 0 | 0 | 0 | 0 | 0 | 0 |
| k__Bacteria;p__Proteobacteria;c__Alphaproteobacteria;o__Rickettsiales;f__mitochondria;g__Citrullus | | 0 | 0 | 0 | 0 | 0 | 0 | 0 |
| k__Bacteria;p__Proteobacteria;c__Alphaproteobacteria;o__Rickettsiales;f__mitochondria;g__Lupinus | | 0 | 0 | 0 | 0 | 0 | 0 | 0 |
| k__Bacteria;p__Proteobacteria;c__Alphaproteobacteria;o__Rickettsiales;f__mitochondria;g__Nageia | | 0 | 0 | 0 | 0 | 0 | 0 | 0 |
| k__Bacteria;p__Proteobacteria;c__Alphaproteobacteria;o__Rickettsiales;f__mitochondria;g__Nelumbo | | 0 | 0 | 0 | 0 | 0 | 0 | 0 |
| k__Bacteria;p__Proteobacteria;c__Alphaproteobacteria;o__Rickettsiales;f__mitochondria;g__Oenothera | | 0 | 0 | 0 | 0 | 0 | 0 | 0 |
| k__Bacteria;p__Proteobacteria;c__Alphaproteobacteria;o__Rickettsiales;f__mitochondria;g__Phyllocladus | | 0 | 0 | 0 | 0 | 0 | 0 | 0 |
| k__Bacteria;p__Proteobacteria;c__Alphaproteobacteria;o__Rickettsiales;f__mitochondria;g__Zea | | 0 | 0 | 4.06E-05 | 0 | 0 | 0 | 0 |
| k__Bacteria;p__Proteobacteria;c__Alphaproteobacteria;o__Sphingomonadales;f__;g__ | | 1.39E-05 | 1.42E-05 | 0 | 1.47E-05 | 0.00011956 | 0 | 3.06E-05 |
| k__Bacteria;p__Proteobacteria;c__Alphaproteobacteria;o__Sphingomonadales;f__Erythrobacteraceae;g__ | | 0 | 0 | 0 | 1.47E-05 | 2.99E-05 | 6.62E-05 | 0 |
| k__Bacteria;p__Proteobacteria;c__Alphaproteobacteria;o__Sphingomonadales;f__Erythrobacteraceae;g__Altererythrobacter | | 0 | 0 | 0 | 0 | 0 | 3.97E-05 | 0 |
| k__Bacteria;p__Proteobacteria;c__Alphaproteobacteria;o__Sphingomonadales;f__Erythrobacteraceae;g__Erythrobacter | | 0 | 0 | 0 | 0 | 0 | 2.65E-05 | 0 |
| k__Bacteria;p__Proteobacteria;c__Alphaproteobacteria;o__Sphingomonadales;f__Erythrobacteraceae;g__Lutibacterium | | 0 | 0 | 0 | 0 | 0 | 3.97E-05 | 0 |
| k__Bacteria;p__Proteobacteria;c__Alphaproteobacteria;o__Sphingomonadales;f__Sphingomonadaceae;g__ | | 2.79E-05 | 1.42E-05 | 2.71E-05 | 0 | 2.99E-05 | 5.29E-05 | 0 |
| k__Bacteria;p__Proteobacteria;c__Alphaproteobacteria;o__Sphingomonadales;f__Sphingomonadaceae;g__Kaistobacter | | 0 | 0 | 0 | 0 | 0 | 0 | 0 |
| k__Bacteria;p__Proteobacteria;c__Alphaproteobacteria;o__Sphingomonadales;f__Sphingomonadaceae;g__Novosphingobium | | 0 | 0 | 0 | 0 | 0 | 0 | 0 |
| k__Bacteria;p__Proteobacteria;c__Alphaproteobacteria;o__Sphingomonadales;f__Sphingomonadaceae;g__Sphingobium | | 0 | 0 | 1.35E-05 | 0 | 0 | 0 | 0 |
| k__Bacteria;p__Proteobacteria;c__Alphaproteobacteria;o__Sphingomonadales;f__Sphingomonadaceae;g__Sphingomonas | | 2.79E-05 | 1.42E-05 | 4.06E-05 | 0 | 2.99E-05 | 3.97E-05 | 0 |
| k__Bacteria;p__Proteobacteria;c__Alphaproteobacteria;o__Sphingomonadales;f__Sphingomonadaceae;g__Sphingopyxis | | 0 | 0 | 0 | 0 | 0 | 0 | 0 |
| k__Bacteria;p__Proteobacteria;c__Alphaproteobacteria;o__Sphingomonadales;f__Sphingomonadaceae;g__Zymomonas | | 0 | 0 | 0 | 0 | 0 | 0 | 0 |
| k__Bacteria;p__Proteobacteria;c__Betaproteobacteria;o__;f__;g__ | | 0.00051577 | 0.00156668 | 0.0004872 | 0.00058912 | 0.00215208 | 0.00228936 | 0.0027533 |
| k__Bacteria;p__Proteobacteria;c__Betaproteobacteria;o__Burkholderiales;Comamonadaceae;g__ | | 0 | 1.42E-05 | 1.35E-05 | 4.42E-05 | 0.00056791 | 0.00037053 | 0.00024474 |
| k__Bacteria;p__Proteobacteria;c__Betaproteobacteria;o__Burkholderiales;f__;g__ | | 0 | 0 | 0 | 0 | 0 | 0 | 0 |
| k__Bacteria;p__Proteobacteria;c__Betaproteobacteria;o__Burkholderiales;f__Alcaligenaceae;g__ | | 0 | 0 | 0 | 0 | 5.98E-05 | 2.65E-05 | 0 |
| k__Bacteria;p__Proteobacteria;c__Betaproteobacteria;o__Burkholderiales;f__Alcaligenaceae;g__Achromobacter | | 0 | 0 | 0 | 0 | 0 | 0 | 0 |
| k__Bacteria;p__Proteobacteria;c__Betaproteobacteria;o__Burkholderiales;f__Alcaligenaceae;g__Sutterella | | 0 | 0 | 0 | 0 | 5.98E-05 | 2.65E-05 | 0 |
| k__Bacteria;p__Proteobacteria;c__Betaproteobacteria;o__Burkholderiales;f__Burkholderiaceae;g__ | | 0 | 0 | 0 | 0 | 0 | 0 | 0 |
| k__Bacteria;p__Proteobacteria;c__Betaproteobacteria;o__Burkholderiales;f__Burkholderiaceae;g__Burkholderia | | 0 | 0 | 0 | 0 | 0 | 0 | 0 |
| k__Bacteria;p__Proteobacteria;c__Betaproteobacteria;o__Burkholderiales;f__Burkholderiaceae;g__Lautropia | | 0 | 0 | 0 | 0 | 0 | 0 | 0 |
| k__Bacteria;p__Proteobacteria;c__Betaproteobacteria;o__Burkholderiales;f__Comamonadaceae;g__Acidovorax | | 0 | 0 | 0 | 0 | 0 | 0 | 0 |
| k__Bacteria;p__Proteobacteria;c__Betaproteobacteria;o__Burkholderiales;f__Comamonadaceae;g__Comamonas | | 0 | 0 | 0 | 0 | 2.99E-05 | 0 | 0 |
| k__Bacteria;p__Proteobacteria;c__Betaproteobacteria;o__Burkholderiales;f__Comamonadaceae;g__Curvibacter | | 0 | 0 | 1.35E-05 | 0 | 0 | 0 | 0 |
| k__Bacteria;p__Proteobacteria;c__Betaproteobacteria;o__Burkholderiales;f__Comamonadaceae;g__Delftia | | 0 | 0 | 0 | 0 | 0 | 0 | 0 |
| k__Bacteria;p__Proteobacteria;c__Betaproteobacteria;o__Burkholderiales;f__Comamonadaceae;g__Hydrogenophaga | | 0 | 0 | 0 | 0 | 5.98E-05 | 1.32E-05 | 0 |
| k__Bacteria;p__Proteobacteria;c__Betaproteobacteria;o__Burkholderiales;f__Comamonadaceae;g__Leptothrix | | 0 | 0 | 0 | 0 | 0 | 0 | 0 |
| k__Bacteria;p__Proteobacteria;c__Betaproteobacteria;o__Burkholderiales;f__Comamonadaceae;g__Limnohabitans | | 0 | 0 | 0 | 0 | 0 | 3.97E-05 | 0 |
| k__Bacteria;p__Proteobacteria;c__Betaproteobacteria;o__Burkholderiales;f__Comamonadaceae;g__Methylibium | | 0 | 2.85E-05 | 0 | 0 | 0 | 0 | 0 |
| k__Bacteria;p__Proteobacteria;c__Betaproteobacteria;o__Burkholderiales;f__Comamonadaceae;g__Polaromonas | | 0 | 0 | 0 | 0 | 2.99E-05 | 0 | 0 |
| k__Bacteria;p__Proteobacteria;c__Betaproteobacteria;o__Burkholderiales;f__Comamonadaceae;g__RS62 | | 2.79E-05 | 0.00015667 | 2.71E-05 | 1.47E-05 | 0.00176351 | 0.00144243 | 0.00015296 |
| k__Bacteria;p__Proteobacteria;c__Betaproteobacteria;o__Burkholderiales;f__Comamonadaceae;g__Rhodoferax | | 0 | 0 | 0 | 0 | 0 | 0 | 0 |
| k__Bacteria;p__Proteobacteria;c__Betaproteobacteria;o__Burkholderiales;f__Comamonadaceae;g__Roseateles | | 0 | 0 | 0 | 0 | 0 | 0 | 0 |
| k__Bacteria;p__Proteobacteria;c__Betaproteobacteria;o__Burkholderiales;f__Comamonadaceae;g__Schlegelella | | 0 | 0 | 0 | 0 | 0 | 0 | 0 |
| k__Bacteria;p__Proteobacteria;c__Betaproteobacteria;o__Burkholderiales;f__Comamonadaceae;g__Tepidimonas | | 0 | 0 | 0 | 0 | 0 | 0 | 0 |
| k__Bacteria;p__Proteobacteria;c__Betaproteobacteria;o__Burkholderiales;f__Comamonadaceae;g__Variovorax | | 0 | 0 | 0 | 0 | 0 | 0 | 0 |
| k__Bacteria;p__Proteobacteria;c__Betaproteobacteria;o__Burkholderiales;f__Oxalobacteraceae;g__ | | 0 | 0 | 0.00010827 | 1.47E-05 | 0.00023912 | 9.26E-05 | 3.06E-05 |
| k__Bacteria;p__Proteobacteria;c__Betaproteobacteria;o__Burkholderiales;f__Oxalobacteraceae;g__Cupriavidus | | 0 | 0 | 0 | 0 | 0 | 0 | 0 |
| k__Bacteria;p__Proteobacteria;c__Betaproteobacteria;o__Burkholderiales;f__Oxalobacteraceae;g__Herbaspirillum | | 1.39E-05 | 0 | 0 | 0 | 0 | 0 | 0 |
| k__Bacteria;p__Proteobacteria;c__Betaproteobacteria;o__Burkholderiales;f__Oxalobacteraceae;g__Janthinobacterium | | 0 | 0 | 0 | 0 | 0 | 7.94E-05 | 0 |
| k__Bacteria;p__Proteobacteria;c__Betaproteobacteria;o__Burkholderiales;f__Oxalobacteraceae;g__Massilia | | 0 | 0 | 1.35E-05 | 0 | 0 | 0 | 0 |

| Taxon | 2028 | 2029 | 1184 | 1196 | 1245 | 1246 | 2019 |
|---|---|---|---|---|---|---|---|
| Copepod genus | Centropages | Centropages | Water | Water | Water | Water | Water |
| Type of sample | Starved | Starved | Water | Water | Water | Water | Water |
| k__Bacteria;p__Proteobacteria;c__Betaproteobacteria;o__Burkholderiales;f__Oxalobacteraceae;g__Polynucleobacter | 0 | 0 | 5.41E-05 | 0 | 2.99E-05 | 2.65E-05 | 0 |
| k__Bacteria;p__Proteobacteria;c__Betaproteobacteria;o__Burkholderiales;f__Oxalobacteraceae;g__Ralstonia | 0 | 0 | 0 | 0 | 0 | 0 | 0 |
| k__Bacteria;p__Proteobacteria;c__Betaproteobacteria;o__Ellin6067;f__;g__ | 0 | 0 | 0 | 0 | 0 | 0 | 0 |
| k__Bacteria;p__Proteobacteria;c__Betaproteobacteria;o__Methylophilales;f__Methylophilaceae;g__ | 0.00029273 | 0.00075486 | 0.00208412 | 0.0024743 | 0.0035868 | 0.00489632 | 0.00541483 |
| k__Bacteria;p__Proteobacteria;c__Betaproteobacteria;o__Methylophilales;f__Methylophilaceae;g__Methylotenera | 0 | 0 | 2.71E-05 | 0 | 8.97E-05 | 0.00013233 | 3.06E-05 |
| k__Bacteria;p__Proteobacteria;c__Betaproteobacteria;o__Neisseriales;f__Neisseriaceae;g__ | 0 | 0 | 0 | 0 | 0 | 0 | 0 |
| k__Bacteria;p__Proteobacteria;c__Betaproteobacteria;o__Neisseriales;f__Neisseriaceae;g__Kingella | 0 | 0 | 0 | 0 | 0 | 0 | 0 |
| k__Bacteria;p__Proteobacteria;c__Betaproteobacteria;o__Neisseriales;f__Neisseriaceae;g__Microvirgula | 0 | 0 | 0 | 0 | 0 | 2.65E-05 | 0 |
| k__Bacteria;p__Proteobacteria;c__Betaproteobacteria;o__Neisseriales;f__Neisseriaceae;g__Neisseria | 0 | 0 | 0 | 0 | 0 | 0 | 0 |
| k__Bacteria;p__Proteobacteria;c__Betaproteobacteria;o__Nitrosomonadales;f__Nitrosomonadaceae;g__ | 0 | 0 | 1.35E-05 | 0 | 0 | 0 | 3.06E-05 |
| k__Bacteria;p__Proteobacteria;c__Betaproteobacteria;o__Procabacteriales;f__Procabacteriaceae;g__ | 0 | 0 | 0 | 0 | 5.98E-05 | 0.00013233 | 0 |
| k__Bacteria;p__Proteobacteria;c__Betaproteobacteria;o__Rhodocyclales;f__Rhodocyclaceae;g__ | 0.00269035 | 0.00591067 | 0.00343745 | 0.00377036 | 0.0173362 | 0.01715034 | 0.00933064 |
| k__Bacteria;p__Proteobacteria;c__Betaproteobacteria;o__Rhodocyclales;f__Rhodocyclaceae;g__Dechloromonas | 0 | 0 | 0 | 0 | 0 | 1.32E-05 | 0 |
| k__Bacteria;p__Proteobacteria;c__Betaproteobacteria;o__Rhodocyclales;f__Rhodocyclaceae;g__Hydrogenophilus | 0 | 0 | 0 | 0 | 0 | 0 | 0 |
| k__Bacteria;p__Proteobacteria;c__Betaproteobacteria;o__Rhodocyclales;f__Rhodocyclaceae;g__Methyloversatilis | 0 | 0 | 0 | 0 | 0 | 0 | 0 |
| k__Bacteria;p__Proteobacteria;c__Betaproteobacteria;o__Rhodocyclales;f__Rhodocyclaceae;g__Propionivibrio | 0 | 0 | 0 | 0 | 0 | 0 | 0 |
| k__Bacteria;p__Proteobacteria;c__Betaproteobacteria;o__Rhodocyclales;f__Rhodocyclaceae;g__Uliginosibacterium | 0 | 0 | 0 | 0 | 0 | 0 | 0 |
| k__Bacteria;p__Proteobacteria;c__Betaproteobacteria;o__Rhodocyclales;f__Rhodocyclaceae;g__Zoogloea | 0 | 0 | 0 | 0 | 0 | 0 | 0 |
| k__Bacteria;p__Proteobacteria;c__Betaproteobacteria;o__SC-I-84;f__;g__ | 0 | 0 | 0 | 0 | 0 | 0 | 0 |
| k__Bacteria;p__Proteobacteria;c__Betaproteobacteria;o__Tremblayales;f__;g__ | 0 | 0 | 0 | 0 | 0 | 1.32E-05 | 0 |
| k__Bacteria;p__Proteobacteria;c__Deltaproteobacteria;o__;f__;g__ | 0 | 0 | 0 | 0 | 0 | 3.97E-05 | 0 |
| k__Bacteria;p__Proteobacteria;c__Deltaproteobacteria;o__Bdellovibrionales;f__Bacteriovoracaceae;g__ | 5.58E-05 | 0.00012818 | 9.47E-05 | 0 | 2.99E-05 | 0 | 0 |
| k__Bacteria;p__Proteobacteria;c__Deltaproteobacteria;o__Bdellovibrionales;f__Bacteriovoracaceae;g__Bacteriovorax | 1.39E-05 | 7.12E-05 | 1.35E-05 | 1.47E-05 | 0 | 2.65E-05 | 0 |
| k__Bacteria;p__Proteobacteria;c__Deltaproteobacteria;o__Bdellovibrionales;f__Bdellovibrionaceae;g__Bdellovibrio | 0 | 0 | 0 | 0 | 2.99E-05 | 0 | 0 |
| k__Bacteria;p__Proteobacteria;c__Deltaproteobacteria;o__Desulfarculales;f__Desulfarculaceae;g__ | 0 | 0 | 0 | 1.47E-05 | 0 | 0 | 0 |
| k__Bacteria;p__Proteobacteria;c__Deltaproteobacteria;o__Desulfobacterales;f__Desulfobacteraceae;g__ | 0 | 0 | 0 | 0 | 5.98E-05 | 1.32E-05 | 0 |
| k__Bacteria;p__Proteobacteria;c__Deltaproteobacteria;o__Desulfobacterales;f__Desulfobacteraceae;g__Desulfococcus | 0 | 0 | 0 | 0 | 8.97E-05 | 0.0001191 | 0 |
| k__Bacteria;p__Proteobacteria;c__Deltaproteobacteria;o__Desulfobacterales;f__Desulfobacteraceae;g__Desulfofrigus | 0 | 0 | 0 | 1.47E-05 | 0 | 0 | 0 |
| k__Bacteria;p__Proteobacteria;c__Deltaproteobacteria;o__Desulfobacterales;f__Desulfobacteraceae;g__Desulfosarcina | 0 | 0 | 0 | 1.47E-05 | 0 | 2.65E-05 | 0 |
| k__Bacteria;p__Proteobacteria;c__Deltaproteobacteria;o__Desulfobacterales;f__Desulfobulbaceae;g__ | 0 | 0 | 0 | 1.47E-05 | 5.98E-05 | 7.94E-05 | 0 |
| k__Bacteria;p__Proteobacteria;c__Deltaproteobacteria;o__Desulfobacterales;f__Nitrospinaceae;g__Nitrospina | 0 | 0 | 0 | 1.47E-05 | 0 | 0 | 0 |
| k__Bacteria;p__Proteobacteria;c__Deltaproteobacteria;o__Desulfovibrionales;f__Desulfovibrionaceae;g__ | 0 | 0 | 0 | 0 | 0 | 0 | 3.06E-05 |
| k__Bacteria;p__Proteobacteria;c__Deltaproteobacteria;o__Desulfovibrionales;f__Desulfovibrionaceae;g__Desulfovibrio | 0 | 0 | 0 | 0 | 0 | 3.97E-05 | 0 |
| k__Bacteria;p__Proteobacteria;c__Deltaproteobacteria;o__Desulfuromonadales;f__Desulfuromonadaceae;g__ | 0 | 0 | 0 | 0 | 5.98E-05 | 6.62E-05 | 0 |
| k__Bacteria;p__Proteobacteria;c__Deltaproteobacteria;o__Desulfuromonadales;f__Desulfuromonadaceae;g__Desulfuromusa | 0 | 0 | 0 | 0 | 0 | 1.32E-05 | 0 |
| k__Bacteria;p__Proteobacteria;c__Deltaproteobacteria;o__FAC87;f__;g__ | 0 | 0 | 0 | 0 | 0 | 0 | 0 |
| k__Bacteria;p__Proteobacteria;c__Deltaproteobacteria;o__MIZ46;f__;g__ | 0 | 0 | 0 | 0 | 0 | 0 | 0 |
| k__Bacteria;p__Proteobacteria;c__Deltaproteobacteria;o__Myxococcales;f__;g__ | 0 | 1.42E-05 | 0 | 0 | 0.00014945 | 0 | 0 |
| k__Bacteria;p__Proteobacteria;c__Deltaproteobacteria;o__Myxococcales;f__0319-6G20;g__ | 0 | 0 | 0 | 0 | 0 | 0 | 0 |
| k__Bacteria;p__Proteobacteria;c__Deltaproteobacteria;o__Myxococcales;f__OM27;g__ | 0 | 4.27E-05 | 0.00017593 | 0.00092786 | 0.00047824 | 0.0005161 | 0.00036711 |
| k__Bacteria;p__Proteobacteria;c__Deltaproteobacteria;o__NB1-j;f__;g__ | 0 | 0 | 1.35E-05 | 0 | 0 | 0 | 0 |
| k__Bacteria;p__Proteobacteria;c__Deltaproteobacteria;o__NB1-j;f__JTB38;g__ | 0 | 0 | 0.00010827 | 0.00039766 | 0.00044835 | 0.00022497 | 3.06E-05 |
| k__Bacteria;p__Proteobacteria;c__Deltaproteobacteria;o__PB19;f__;g__ | 0 | 0 | 5.41E-05 | 0.0002651 | 0 | 5.29E-05 | 0.00012237 |
| k__Bacteria;p__Proteobacteria;c__Deltaproteobacteria;o__Sva0853;f__;g__ | 0 | 0 | 0 | 0 | 0 | 0 | 0 |
| k__Bacteria;p__Proteobacteria;c__Deltaproteobacteria;o__Sva0853;f__S25_1238;g__ | 0 | 0 | 0 | 0 | 2.99E-05 | 1.32E-05 | 0 |
| k__Bacteria;p__Proteobacteria;c__Deltaproteobacteria;o__Sva0853;f__SAR324;g__ | 0 | 0 | 5.41E-05 | 1.47E-05 | 0 | 0 | 0 |
| k__Bacteria;p__Proteobacteria;c__Deltaproteobacteria;o__Syntrophobacterales;f__Syntrophobacteraceae;g__ | 0 | 0 | 0 | 0 | 0 | 2.65E-05 | 0 |
| k__Bacteria;p__Proteobacteria;c__Epsilonproteobacteria;o__Campylobacterales;f__Campylobacteraceae;g__Arcobacter | 0 | 8.55E-05 | 1.35E-05 | 0.00030929 | 0.00053802 | 0.0006352 | 0 |
| k__Bacteria;p__Proteobacteria;c__Epsilonproteobacteria;o__Campylobacterales;f__Campylobacteraceae;g__Campylobacter | 0 | 0 | 0 | 0 | 0 | 1.32E-05 | 0 |
| k__Bacteria;p__Proteobacteria;c__Epsilonproteobacteria;o__Campylobacterales;f__Campylobacteraceae;g__Sulfurospirillum | 0 | 0 | 0 | 1.47E-05 | 5.98E-05 | 2.65E-05 | 0 |
| k__Bacteria;p__Proteobacteria;c__Epsilonproteobacteria;o__Campylobacterales;f__Helicobacteraceae;g__ | 0 | 0 | 0 | 0.00023565 | 0.00032879 | 0.00014557 | 0 |
| k__Bacteria;p__Proteobacteria;c__Epsilonproteobacteria;o__Campylobacterales;f__Helicobacteraceae;g__Sulfurimonas | 0 | 0 | 0 | 5.89E-05 | 0.00020923 | 7.94E-05 | 0 |
| k__Bacteria;p__Proteobacteria;c__Epsilonproteobacteria;o__Campylobacterales;f__Helicobacteraceae;g__Wolinella | 0 | 0 | 0 | 0 | 0 | 0 | 0 |
| k__Bacteria;p__Proteobacteria;c__Gammaproteobacteria;o__;f__;g__ | 0.00015334 | 0.00011394 | 2.71E-05 | 0.00013255 | 5.98E-05 | 0.0001191 | 0.0003977 |
| k__Bacteria;p__Proteobacteria;c__Gammaproteobacteria;o__Aeromonadales;f__Aeromonadaceae;g__ | 0 | 0 | 0 | 0 | 0.00017934 | 0.00010587 | 3.06E-05 |
| k__Bacteria;p__Proteobacteria;c__Gammaproteobacteria;o__Aeromonadales;f__Aeromonadaceae;g__Aeromonas | 0 | 0 | 0 | 0 | 0 | 3.97E-05 | 0 |
| k__Bacteria;p__Proteobacteria;c__Gammaproteobacteria;o__Aeromonadales;f__Aeromonadaceae;g__Tolumonas | 0 | 0 | 0 | 0 | 8.97E-05 | 9.26E-05 | 0 |
| k__Bacteria;p__Proteobacteria;c__Gammaproteobacteria;o__Alteromonadales;f__;g__ | 2.79E-05 | 0 | 4.06E-05 | 8.84E-05 | 0.00035868 | 0.00021173 | 0.00027533 |
| k__Bacteria;p__Proteobacteria;c__Gammaproteobacteria;o__Alteromonadales;f__Alteromonadaceae;g__ | 0.02712649 | 0.0181308 | 6.77E-05 | 0.00016201 | 0.00020923 | 0.00013233 | 0.00116251 |
| k__Bacteria;p__Proteobacteria;c__Gammaproteobacteria;o__Alteromonadales;f__Alteromonadaceae;g__Alteromonas | 0.01598874 | 0.01008375 | 0 | 0 | 0 | 1.32E-05 | 0.00520069 |
| k__Bacteria;p__Proteobacteria;c__Gammaproteobacteria;o__Alteromonadales;f__Alteromonadaceae;g__BD2-13 | 0 | 0 | 0 | 0 | 0 | 2.65E-05 | 0 |
| k__Bacteria;p__Proteobacteria;c__Gammaproteobacteria;o__Alteromonadales;f__Alteromonadaceae;g__Candidatus Endobugula | 0 | 0 | 0 | 2.95E-05 | 0 | 0 | 0 |
| k__Bacteria;p__Proteobacteria;c__Gammaproteobacteria;o__Alteromonadales;f__Alteromonadaceae;g__Cellvibrio | 0 | 0 | 0 | 0 | 0 | 0 | 0 |
| k__Bacteria;p__Proteobacteria;c__Gammaproteobacteria;o__Alteromonadales;f__Alteromonadaceae;g__Glaciecola | 0.06741197 | 0.16159631 | 0.00023007 | 0.0001031 | 0.0002989 | 0.0002382 | 0.00030592 |
| k__Bacteria;p__Proteobacteria;c__Gammaproteobacteria;o__Alteromonadales;f__Alteromonadaceae;g__HTCC2207 | 0.00022303 | 0.00042728 | 0.00860716 | 0.021061 | 0.00884744 | 0.00816494 | 0.0031816 |
| k__Bacteria;p__Proteobacteria;c__Gammaproteobacteria;o__Alteromonadales;f__Alteromonadaceae;g__Marinobacter | 0.00365218 | 0.00843161 | 0 | 0 | 0 | 1.32E-05 | 3.06E-05 |
| k__Bacteria;p__Proteobacteria;c__Gammaproteobacteria;o__Alteromonadales;f__Alteromonadaceae;g__Porticoccus | 0 | 0 | 0 | 0 | 8.97E-05 | 1.32E-05 | 0 |
| k__Bacteria;p__Proteobacteria;c__Gammaproteobacteria;o__Alteromonadales;f__Alteromonadaceae;g__Spongiibacter | 0 | 0 | 0 | 1.47E-05 | 0 | 0 | 0 |
| k__Bacteria;p__Proteobacteria;c__Gammaproteobacteria;o__Alteromonadales;f__Alteromonadaceae;g__ZD0117 | 0.00057152 | 0.00243548 | 0.000609 | 0.00011782 | 8.97E-05 | 0.0002382 | 0.00100954 |
| k__Bacteria;p__Proteobacteria;c__Gammaproteobacteria;o__Alteromonadales;f__Alteromonadaceae;g__nsmpVI18 | 0 | 0 | 0 | 0 | 0 | 0 | 0 |
| k__Bacteria;p__Proteobacteria;c__Gammaproteobacteria;o__Alteromonadales;f__Colwelliaceae;g__ | 0.01763361 | 0.03251581 | 0.00086613 | 0.0002651 | 8.97E-05 | 0.0002382 | 6.12E-05 |
| k__Bacteria;p__Proteobacteria;c__Gammaproteobacteria;o__Alteromonadales;f__Colwelliaceae;g__Colwellia | 2.79E-05 | 1.42E-05 | 2.71E-05 | 1.47E-05 | 0 | 1.32E-05 | 0 |
| k__Bacteria;p__Proteobacteria;c__Gammaproteobacteria;o__Alteromonadales;f__Colwelliaceae;g__Thalassomonas | 1.39E-05 | 0 | 1.35E-05 | 0 | 0 | 2.65E-05 | 6.12E-05 |
| k__Bacteria;p__Proteobacteria;c__Gammaproteobacteria;o__Alteromonadales;f__HTCC2188;g__ | 0 | 1.42E-05 | 0 | 0.00053021 | 0.00295911 | 0.00250109 | 0.00036711 |

| Taxon | 2028 | 2029 | 1184 | 1196 | 1245 | 1246 | 2019 |
|---|---|---|---|---|---|---|---|
| Copepod genus | Centropages | Centropages | Water | Water | Water | Water | Water |
| Type of sample | Starved | Starved | Water | Water | Water | Water | Water |
| k__Bacteria;p__Proteobacteria;c__Gammaproteobacteria;o__Alteromonadales;f__HTCC2188;g__HTCC | 0.00023697 | 0.00047001 | 0.0006902 | 0.00013255 | 0.00035868 | 0.00030437 | 0.00030592 |
| k__Bacteria;p__Proteobacteria;c__Gammaproteobacteria;o__Alteromonadales;f__Idiomarinaceae;g__Idiomarina | 9.76E-05 | 0.00021364 | 0 | 0 | 0 | 0 | 0 |
| k__Bacteria;p__Proteobacteria;c__Gammaproteobacteria;o__Alteromonadales;f__Moritellaceae;g__Moritella | 1.39E-05 | 0 | 0 | 4.42E-05 | 2.99E-05 | 0 | 0 |
| k__Bacteria;p__Proteobacteria;c__Gammaproteobacteria;o__Alteromonadales;f__OM60;g__ | 0.00034849 | 0.0007691 | 0.00320738 | 0.00726089 | 0.01629005 | 0.01220109 | 0.02123103 |
| k__Bacteria;p__Proteobacteria;c__Gammaproteobacteria;o__Alteromonadales;f__OM60;g__Congregibacter | 0 | 0 | 0 | 0 | 5.98E-05 | 0 | 0 |
| k__Bacteria;p__Proteobacteria;c__Gammaproteobacteria;o__Alteromonadales;f__Psychromonadaceae;g__Psychromonas | 1.39E-05 | 0 | 1.35E-05 | 0.00029456 | 0.00020923 | 0.00010587 | 3.06E-05 |
| k__Bacteria;p__Proteobacteria;c__Gammaproteobacteria;o__Alteromonadales;f__Shewanellaceae;g__Shewanella | 0.00030667 | 7.12E-05 | 2.71E-05 | 7.36E-05 | 5.98E-05 | 0.00010587 | 0 |
| k__Bacteria;p__Proteobacteria;c__Gammaproteobacteria;o__Alteromonadales;f__[Chromatiaceae];g__ | 0 | 0 | 0 | 0 | 2.99E-05 | 0 | 0 |
| k__Bacteria;p__Proteobacteria;c__Gammaproteobacteria;o__Alteromonadales;f__[Chromatiaceae];g__Rheinheimera | 0 | 0 | 0 | 0 | 0 | 0 | 0 |
| k__Bacteria;p__Proteobacteria;c__Gammaproteobacteria;o__Cardiobacteriales;f__Cardiobacteriaceae;g__Cardiobacterium | 0 | 1.42E-05 | 0 | 0 | 0 | 0 | 0 |
| k__Bacteria;p__Proteobacteria;c__Gammaproteobacteria;o__Chromatiales;f__;g__ | 0 | 0 | 0.00014887 | 5.89E-05 | 0.00014945 | 0.00013233 | 0.00018355 |
| k__Bacteria;p__Proteobacteria;c__Gammaproteobacteria;o__Chromatiales;f__Chromatiaceae;g__ | 0 | 0 | 0 | 0 | 0.00011956 | 0 | 0 |
| k__Bacteria;p__Proteobacteria;c__Gammaproteobacteria;o__Enterobacteriales;f__Enterobacteriaceae;g__ | 0 | 0 | 0 | 0 | 5.98E-05 | 6.62E-05 | 0 |
| k__Bacteria;p__Proteobacteria;c__Gammaproteobacteria;o__Enterobacteriales;f__Enterobacteriaceae;g__Enterobacter | 0 | 0 | 0 | 0 | 0 | 0 | 0 |
| k__Bacteria;p__Proteobacteria;c__Gammaproteobacteria;o__Enterobacteriales;f__Enterobacteriaceae;g__Erwinia | 0 | 0 | 0 | 0 | 0 | 0 | 0 |
| k__Bacteria;p__Proteobacteria;c__Gammaproteobacteria;o__Enterobacteriales;f__Enterobacteriaceae;g__Escherichia | 0 | 0 | 0 | 0 | 0 | 0 | 0 |
| k__Bacteria;p__Proteobacteria;c__Gammaproteobacteria;o__Enterobacteriales;f__Enterobacteriaceae;g__Ewingella | 0 | 0 | 0 | 0.00027983 | 5.98E-05 | 3.97E-05 | 3.06E-05 |
| k__Bacteria;p__Proteobacteria;c__Gammaproteobacteria;o__Enterobacteriales;f__Enterobacteriaceae;g__Gluconacetobacter | 0 | 0 | 0 | 0 | 0 | 0 | 0 |
| k__Bacteria;p__Proteobacteria;c__Gammaproteobacteria;o__Enterobacteriales;f__Enterobacteriaceae;g__Klebsiella | 0 | 0 | 0 | 0 | 0 | 0 | 0 |
| k__Bacteria;p__Proteobacteria;c__Gammaproteobacteria;o__Enterobacteriales;f__Enterobacteriaceae;g__Pantoea | 0 | 1.42E-05 | 0 | 0 | 8.97E-05 | 0 | 0 |
| k__Bacteria;p__Proteobacteria;c__Gammaproteobacteria;o__Enterobacteriales;f__Enterobacteriaceae;g__Salmonella | 0 | 0 | 0 | 0 | 0 | 0 | 0 |
| k__Bacteria;p__Proteobacteria;c__Gammaproteobacteria;o__Enterobacteriales;f__Enterobacteriaceae;g__Serratia | 0 | 0 | 0 | 0 | 0 | 1.32E-05 | 0 |
| k__Bacteria;p__Proteobacteria;c__Gammaproteobacteria;o__HTCC2188;f__HTCC2089;g__ | 0.00027879 | 0.00018515 | 0.00063606 | 0.00094259 | 0.0017934 | 0.00247463 | 0.00137665 |
| k__Bacteria;p__Proteobacteria;c__Gammaproteobacteria;o__HTCC2188;f__HTCC2089;g__Acinetobacter | 0 | 2.85E-05 | 0 | 0 | 0 | 0 | 0 |
| k__Bacteria;p__Proteobacteria;c__Gammaproteobacteria;o__Legionellales;f__;g__ | 0 | 0 | 0 | 0 | 0 | 2.65E-05 | 0 |
| k__Bacteria;p__Proteobacteria;c__Gammaproteobacteria;o__Legionellales;f__Coxiellaceae;g__ | 0 | 0 | 1.35E-05 | 0 | 0 | 0 | 0 |
| k__Bacteria;p__Proteobacteria;c__Gammaproteobacteria;o__Legionellales;f__Francisellaceae;g__ | 0 | 5.70E-05 | 0 | 1.47E-05 | 0 | 1.32E-05 | 6.12E-05 |
| k__Bacteria;p__Proteobacteria;c__Gammaproteobacteria;o__Legionellales;f__Legionellaceae;g__ | 1.39E-05 | 1.42E-05 | 0 | 0 | 2.99E-05 | 0 | 0 |
| k__Bacteria;p__Proteobacteria;c__Gammaproteobacteria;o__Legionellales;f__Legionellaceae;g__Legionella | 0 | 0 | 0 | 0 | 0 | 1.32E-05 | 0 |
| k__Bacteria;p__Proteobacteria;c__Gammaproteobacteria;o__Methylococcales;f__;g__ | 0 | 0 | 0 | 0 | 0 | 0 | 0 |
| k__Bacteria;p__Proteobacteria;c__Gammaproteobacteria;o__Oceanospirillales;f__;g__ | 8.36E-05 | 0.00015667 | 0.00801169 | 0.00201773 | 0.0008967 | 0.00133656 | 0.00061185 |
| k__Bacteria;p__Proteobacteria;c__Gammaproteobacteria;o__Oceanospirillales;f__Alcanivoracaceae;g__Alcanivorax | 8.36E-05 | 0.00018515 | 0 | 0 | 0 | 0 | 3.06E-05 |
| k__Bacteria;p__Proteobacteria;c__Gammaproteobacteria;o__Oceanospirillales;f__Endozoicimonaceae;g__Endozoicomonas | 0 | 0 | 0 | 0 | 5.98E-05 | 0 | 0 |
| k__Bacteria;p__Proteobacteria;c__Gammaproteobacteria;o__Oceanospirillales;f__Halomonadaceae;g__ | 0.011542 | 0.02069447 | 0 | 0 | 5.98E-05 | 0 | 0 |
| k__Bacteria;p__Proteobacteria;c__Gammaproteobacteria;o__Oceanospirillales;f__Halomonadaceae;g__Candidatus Portiera | 0.00260671 | 0.00666553 | 0.09615385 | 0.05978085 | 0.03293879 | 0.04234653 | 0.02581987 |
| k__Bacteria;p__Proteobacteria;c__Gammaproteobacteria;o__Oceanospirillales;f__Halomonadaceae;g__Cobetia | 0 | 0 | 0 | 0 | 0 | 0 | 0 |
| k__Bacteria;p__Proteobacteria;c__Gammaproteobacteria;o__Oceanospirillales;f__Halomonadaceae;g__Haererehalobacter | 0 | 0 | 0 | 0 | 0 | 0 | 0 |
| k__Bacteria;p__Proteobacteria;c__Gammaproteobacteria;o__Oceanospirillales;f__Halomonadaceae;g__Halomonas | 0.00206306 | 0.00246397 | 0 | 0 | 0.00053802 | 0.00026467 | 3.06E-05 |
| k__Bacteria;p__Proteobacteria;c__Gammaproteobacteria;o__Oceanospirillales;f__Oceanospirillaceae;g__ | 0.00011152 | 0.00024212 | 0.00454718 | 0.00191464 | 0.00092659 | 0.0013498 | 0.00107073 |
| k__Bacteria;p__Proteobacteria;c__Gammaproteobacteria;o__Oceanospirillales;f__Oceanospirillaceae;g__Amphritea | 0 | 0 | 0 | 0 | 0 | 1.32E-05 | 0 |
| k__Bacteria;p__Proteobacteria;c__Gammaproteobacteria;o__Oceanospirillales;f__Oceanospirillaceae;g__Marinobacterium | 0 | 0 | 0 | 0 | 5.98E-05 | 0 | 0 |
| k__Bacteria;p__Proteobacteria;c__Gammaproteobacteria;o__Oceanospirillales;f__Oceanospirillaceae;g__Marinomonas | 0.03218657 | 0.00830342 | 0 | 8.84E-05 | 0 | 9.26E-05 | 6.12E-05 |
| k__Bacteria;p__Proteobacteria;c__Gammaproteobacteria;o__Oceanospirillales;f__Oceanospirillaceae;g__Neptunomonas | 0 | 0 | 0 | 2.95E-05 | 5.98E-05 | 1.32E-05 | 0 |
| k__Bacteria;p__Proteobacteria;c__Gammaproteobacteria;o__Oceanospirillales;f__Oceanospirillaceae;g__Oceaniserpentilla | 0 | 0 | 0 | 0 | 0 | 0 | 0.00027533 |
| k__Bacteria;p__Proteobacteria;c__Gammaproteobacteria;o__Oceanospirillales;f__Oceanospirillaceae;g__Oleibacter | 0.00129638 | 0.00139577 | 0 | 0 | 0 | 0 | 0.00045888 |
| k__Bacteria;p__Proteobacteria;c__Gammaproteobacteria;o__Oceanospirillales;f__Oceanospirillaceae;g__Oleispira | 5.58E-05 | 0.00012818 | 0.00041953 | 0 | 2.99E-05 | 2.65E-05 | 0.0007954 |
| k__Bacteria;p__Proteobacteria;c__Gammaproteobacteria;o__Oceanospirillales;f__Oceanospirillaceae;g__Spongiispira | 0 | 0 | 0 | 0 | 0 | 0 | 3.06E-05 |
| k__Bacteria;p__Proteobacteria;c__Gammaproteobacteria;o__Oceanospirillales;f__Oleiphilaceae;g__ | 2.79E-05 | 1.42E-05 | 0 | 0 | 0 | 0 | 0 |
| k__Bacteria;p__Proteobacteria;c__Gammaproteobacteria;o__Oceanospirillales;f__SUP05;g__ | 5.58E-05 | 0 | 0.01847291 | 0.00428584 | 0.00047824 | 0.00074106 | 0.00027533 |
| k__Bacteria;p__Proteobacteria;c__Gammaproteobacteria;o__Oceanospirillales;f__Saccharospirillaceae;g__Reinekea | 0 | 0 | 0 | 0 | 0 | 2.65E-05 | 0 |
| k__Bacteria;p__Proteobacteria;c__Gammaproteobacteria;o__Oceanospirillales;f__Saccharospirillaceae;g__Saccharospirillum | 0 | 1.42E-05 | 0 | 0 | 0 | 0 | 0 |
| k__Bacteria;p__Proteobacteria;c__Gammaproteobacteria;o__Pasteurellales;f__Pasteurellaceae;g__Actinobacillus | 0 | 0 | 0 | 0 | 0 | 0 | 0 |
| k__Bacteria;p__Proteobacteria;c__Gammaproteobacteria;o__Pasteurellales;f__Pasteurellaceae;g__Aggregatibacter | 0 | 0 | 0 | 0 | 0 | 0 | 0 |
| k__Bacteria;p__Proteobacteria;c__Gammaproteobacteria;o__Pasteurellales;f__Pasteurellaceae;g__Haemophilus | 0 | 1.42E-05 | 0 | 0 | 0 | 0 | 0 |
| k__Bacteria;p__Proteobacteria;c__Gammaproteobacteria;o__Pseudomonadales;f__Moraxellaceae;g__ | 0.03091806 | 0.02607816 | 0.00033833 | 2.95E-05 | 2.99E-05 | 7.94E-05 | 9.18E-05 |
| k__Bacteria;p__Proteobacteria;c__Gammaproteobacteria;o__Pseudomonadales;f__Moraxellaceae;g__Acinetobacter | 0 | 0 | 0 | 0 | 0.00014945 | 3.97E-05 | 3.06E-05 |
| k__Bacteria;p__Proteobacteria;c__Gammaproteobacteria;o__Pseudomonadales;f__Moraxellaceae;g__Alkanindiges | 0 | 0 | 0 | 0 | 0 | 5.29E-05 | 0 |
| k__Bacteria;p__Proteobacteria;c__Gammaproteobacteria;o__Pseudomonadales;f__Moraxellaceae;g__Enhydrobacter | 0 | 0 | 0 | 0 | 0 | 0 | 0 |
| k__Bacteria;p__Proteobacteria;c__Gammaproteobacteria;o__Pseudomonadales;f__Moraxellaceae;g__Perlucidibaca | 0 | 0 | 0 | 0 | 0 | 0 | 0 |
| k__Bacteria;p__Proteobacteria;c__Gammaproteobacteria;o__Pseudomonadales;f__Moraxellaceae;g__Psychrobacter | 0.00844741 | 0.00840312 | 6.77E-05 | 0 | 5.98E-05 | 1.32E-05 | 0 |
| k__Bacteria;p__Proteobacteria;c__Gammaproteobacteria;o__Pseudomonadales;f__Pseudomonadaceae;g__ | 0 | 0 | 0 | 0 | 0 | 0 | 0 |
| k__Bacteria;p__Proteobacteria;c__Gammaproteobacteria;o__Pseudomonadales;f__Pseudomonadaceae;g__Pseudomonas | 0.00114305 | 0.00021364 | 0 | 0 | 0 | 1.32E-05 | 0 |
| k__Bacteria;p__Proteobacteria;c__Gammaproteobacteria;o__Salinisphaerales;f__Salinisphaeraceae;g__Salinisphaera | 1.39E-05 | 0 | 0 | 0 | 0 | 0 | 0 |
| k__Bacteria;p__Proteobacteria;c__Gammaproteobacteria;o__Thiohalorhabdales;f__;g__ | 0 | 0 | 5.41E-05 | 0.0001031 | 0.0002989 | 0.0002382 | 0.00015296 |
| k__Bacteria;p__Proteobacteria;c__Gammaproteobacteria;o__Thiohalorhabdales;f__Thiohalorhabdaceae;g__ | 0 | 0 | 0 | 2.95E-05 | 0 | 0 | 0 |
| k__Bacteria;p__Proteobacteria;c__Gammaproteobacteria;o__Thiotrichales;f__Piscirickettsiaceae;g__ | 5.58E-05 | 0.00015667 | 0.00295025 | 0.00167899 | 0.00068747 | 0.0007146 | 0.00330396 |
| k__Bacteria;p__Proteobacteria;c__Gammaproteobacteria;o__Thiotrichales;f__Piscirickettsiaceae;g__Methylophaga | 0 | 0.00024212 | 0 | 1.47E-05 | 0 | 0 | 0 |
| k__Bacteria;p__Proteobacteria;c__Gammaproteobacteria;o__Thiotrichales;f__Thiotrichaceae;g__ | 0 | 0 | 0 | 0 | 0 | 3.97E-05 | 0 |
| k__Bacteria;p__Proteobacteria;c__Gammaproteobacteria;o__Thiotrichales;f__Thiotrichaceae;g__Cocleimonas | 0 | 0 | 0 | 0 | 5.98E-05 | 2.65E-05 | 0 |
| k__Bacteria;p__Proteobacteria;c__Gammaproteobacteria;o__Thiotrichales;f__Thiotrichaceae;g__E8 | 0 | 0 | 0 | 0 | 0 | 0 | 0 |
| k__Bacteria;p__Proteobacteria;c__Gammaproteobacteria;o__Thiotrichales;f__Thiotrichaceae;g__Leucothrix | 0 | 0 | 0 | 0.00054494 | 0.00020923 | 0.00010587 | 0 |
| k__Bacteria;p__Proteobacteria;c__Gammaproteobacteria;o__Thiotrichales;f__Thiotrichaceae;g__Thiothrix | 0 | 0 | 0 | 0.00025038 | 2.99E-05 | 2.65E-05 | 0 |
| k__Bacteria;p__Proteobacteria;c__Gammaproteobacteria;o__Vibrionales;f__Pseudoalteromonadaceae;g__ | 0.00255095 | 0.00378853 | 0.00033833 | 0.00039766 | 5.98E-05 | 9.26E-05 | 0.0023862 |
| k__Bacteria;p__Proteobacteria;c__Gammaproteobacteria;o__Vibrionales;f__Pseudoalteromonadaceae;g__Pseudoalteromonas | 0.58893473 | 0.36355609 | 0.00776809 | 0.00053021 | 0.00352702 | 0.00207763 | 0.02306657 |

| Taxon | | 2028 | 2029 | 1184 | 1196 | 1245 | 1246 | 2019 |
|---|---|---|---|---|---|---|---|---|
| | Copepod genus | Centropages | Centropages | Water | Water | Water | Water | Water |
| | Type of sample | Starved | Starved | Water | Water | Water | Water | Water |
| k__Bacteria;p__Proteobacteria;c__Gammaproteobacteria;o__Vibrionales;f__Vibrionaceae;g__ | | 0.00150548 | 0.00196548 | 0.000203 | 0.00014728 | 2.99E-05 | 9.26E-05 | 0.00152961 |
| k__Bacteria;p__Proteobacteria;c__Gammaproteobacteria;o__Vibrionales;f__Vibrionaceae;g__Aliivibrio | | 0.00046001 | 0.00091153 | 4.06E-05 | 0.00014728 | 0.00011956 | 0.0001191 | 0.00033651 |
| k__Bacteria;p__Proteobacteria;c__Gammaproteobacteria;o__Vibrionales;f__Vibrionaceae;g__Enterovibrio | | 1.39E-05 | 0 | 0 | 0 | 5.98E-05 | 0 | 3.06E-05 |
| k__Bacteria;p__Proteobacteria;c__Gammaproteobacteria;o__Vibrionales;f__Vibrionaceae;g__Listonella | | 0 | 4.27E-05 | 0 | 0 | 0 | 0 | 0 |
| k__Bacteria;p__Proteobacteria;c__Gammaproteobacteria;o__Vibrionales;f__Vibrionaceae;g__Photobacterium | | 1.39E-05 | 0.00011394 | 1.35E-05 | 8.84E-05 | 0 | 9.26E-05 | 3.06E-05 |
| k__Bacteria;p__Proteobacteria;c__Gammaproteobacteria;o__Vibrionales;f__Vibrionaceae;g__Vibrio | | 0.00011152 | 1.42E-05 | 0 | 5.89E-05 | 5.98E-05 | 3.97E-05 | 0.00015296 |
| k__Bacteria;p__Proteobacteria;c__Gammaproteobacteria;o__Xanthomonadales;f__Sinobacteraceae;g__ | | 0 | 0 | 0 | 0 | 5.98E-05 | 0 | 0 |
| k__Bacteria;p__Proteobacteria;c__Gammaproteobacteria;o__Xanthomonadales;f__Xanthomonadaceae;g__ | | 1.39E-05 | 2.85E-05 | 0 | 0 | 2.99E-05 | 0 | 0 |
| k__Bacteria;p__Proteobacteria;c__Gammaproteobacteria;o__Xanthomonadales;f__Xanthomonadaceae;g__Aspromonas | | 0 | 0 | 0 | 0 | 0 | 0 | 0 |
| k__Bacteria;p__Proteobacteria;c__Gammaproteobacteria;o__Xanthomonadales;f__Xanthomonadaceae;g__Ignatzschineria | | 0 | 0 | 0 | 1.47E-05 | 0 | 0 | 0 |
| k__Bacteria;p__Proteobacteria;c__Gammaproteobacteria;o__Xanthomonadales;f__Xanthomonadaceae;g__Lysobacter | | 0 | 0 | 0 | 0 | 0 | 1.32E-05 | 0 |
| k__Bacteria;p__Proteobacteria;c__Gammaproteobacteria;o__Xanthomonadales;f__Xanthomonadaceae;g__Pseudoxanthomonas | | 0 | 0 | 0 | 0 | 0 | 0 | 0 |
| k__Bacteria;p__Proteobacteria;c__Gammaproteobacteria;o__Xanthomonadales;f__Xanthomonadaceae;g__Stenotrophomonas | | 0 | 0 | 0 | 0 | 0 | 0 | 0 |
| k__Bacteria;p__Proteobacteria;c__Gammaproteobacteria;o__Xanthomonadales;f__Xanthomonadaceae;g__Thermomonas | | 0 | 0 | 0 | 0 | 0 | 0 | 0 |
| k__Bacteria;p__Proteobacteria;c__Gammaproteobacteria;o__[Marinicellales];f__[Marinicellaceae];g__ | | 0 | 0 | 2.71E-05 | 0 | 0.00011956 | 5.29E-05 | 0 |
| k__Bacteria;p__Proteobacteria;c__Gammaproteobacteria;o__[Marinicellales];f__[Marinicellaceae];g__Marinicella | | 0 | 0 | 0.00010827 | 2.95E-05 | 0 | 2.65E-05 | 0 |
| k__Bacteria;p__Proteobacteria;c__TA18;o__CV90;f__;g__ | | 0 | 0 | 0 | 0 | 0 | 1.32E-05 | 0 |
| k__Bacteria;p__Proteobacteria;c__Zetaproteobacteria;o__Mariprofundales;f__Mariprofundaceae;g__Mariprofundus | | 0 | 0 | 0 | 0 | 0 | 1.32E-05 | 0 |
| k__Bacteria;p__SAR406;c__AB16;o__Arctic96B-7;f__A714017;g__SGSH944 | | 0 | 0 | 0.00035186 | 0.0001031 | 0 | 2.65E-05 | 6.12E-05 |
| k__Bacteria;p__SAR406;c__AB16;o__Arctic96B-7;f__A714017;g__SargSea-WGS | | 0 | 0 | 5.41E-05 | 4.42E-05 | 0 | 0 | 0 |
| k__Bacteria;p__SAR406;c__AB16;o__Arctic96B-7;f__A714017;g__ZA3312c | | 0 | 0 | 0.0002842 | 0.00014728 | 0 | 0 | 0 |
| k__Bacteria;p__SAR406;c__AB16;o__ZA3648c;f__AEGEAN_185;g__ | | 0 | 0 | 0 | 0 | 0 | 0 | 0 |
| k__Bacteria;p__SBR1093;c__A712011;o__;f__;g__ | | 0 | 0 | 0 | 0 | 0 | 0 | 0 |
| k__Bacteria;p__SR1;c__;o__;f__;g__ | | 0 | 0 | 0 | 0 | 0 | 0 | 0 |
| k__Bacteria;p__Spirochaetes;c__Spirochaetes;o__Sphaerochaetales;f__Sphaerochaetaceae;g__wall-less | | 0 | 0 | 0 | 0 | 2.99E-05 | 0 | 0 |
| k__Bacteria;p__Spirochaetes;c__Spirochaetes;o__Spirochaetales;f__Spirochaetaceae;g__Spirochaeta | | 0 | 0 | 0 | 0 | 0 | 0 | 0 |
| k__Bacteria;p__Spirochaetes;c__Spirochaetes;o__Spirochaetales;f__Spirochaetaceae;g__Treponema | | 0 | 0 | 0 | 0 | 2.99E-05 | 0 | 0 |
| k__Bacteria;p__TM6;c__SJA-4;o__;f__;g__ | | 0 | 0 | 0 | 0 | 0 | 0 | 3.06E-05 |
| k__Bacteria;p__TM7;c__TM7-1;o__;f__;g__ | | 0 | 0 | 0 | 0 | 0 | 0 | 0 |
| k__Bacteria;p__TM7;c__TM7-3;o__;f__;g__ | | 0 | 0 | 0 | 0 | 0 | 0 | 0 |
| k__Bacteria;p__TM7;c__TM7-3;o__CW040;f__F16;g__ | | 0 | 0 | 0 | 0 | 0 | 0 | 0 |
| k__Bacteria;p__TM7;c__TM7-3;o__EW055;f__;g__ | | 0 | 4.27E-05 | 0 | 0 | 0 | 1.32E-05 | 0 |
| k__Bacteria;p__TM7;c__TM7-3;o__I025;f__Rs-045;g__ | | 0 | 0 | 0 | 0 | 0 | 0 | 0 |
| k__Bacteria;p__Tenericutes;c__Mollicutes;o__Acholeplasmatales;f__Acholeplasmataceae;g__Acholeplasma | | 0 | 0 | 0 | 0 | 0 | 0 | 0 |
| k__Bacteria;p__Tenericutes;c__Mollicutes;o__Mycoplasmatales;f__Mycoplasmataceae;g__Mycoplasma | | 0 | 0 | 0 | 1.47E-05 | 0 | 0 | 0 |
| k__Bacteria;p__Thermotogae;c__Thermotogae;o__Thermotogales;f__Thermotogaceae;g__Fervidobacterium | | 0 | 0 | 0 | 0 | 0 | 0 | 0 |
| k__Bacteria;p__Verrucomicrobia;c__Opitutae;o__Opitutales;f__Opitutaceae;g__Opitutus | | 1.39E-05 | 0 | 0 | 0 | 0 | 3.97E-05 | 0 |
| k__Bacteria;p__Verrucomicrobia;c__Opitutae;o__Puniceicoccales;f__Puniceicoccaceae;g__Coraliomargarita | | 4.18E-05 | 0 | 4.06E-05 | 0.00011782 | 0.00017934 | 0.00025143 | 0.00015296 |
| k__Bacteria;p__Verrucomicrobia;c__Opitutae;o__Puniceicoccales;f__Puniceicoccaceae;g__MB11C04 | | 0.00012546 | 0.00014243 | 0.00037893 | 0.00041238 | 0.00080703 | 0.0009925 | 0.00204968 |
| k__Bacteria;p__Verrucomicrobia;c__Verrucomicrobiae;o__Verrucomicrobiales;f__Verrucomicrobiaceae;g__ | | 0.00032061 | 0.00054122 | 0.00098793 | 0.00366727 | 0.00050813 | 0.0007543 | 0.0023556 |
| k__Bacteria;p__Verrucomicrobia;c__Verrucomicrobiae;o__Verrucomicrobiales;f__Verrucomicrobiaceae;g__Akkermansia | | 0 | 0 | 0 | 0 | 0 | 0 | 0 |
| k__Bacteria;p__Verrucomicrobia;c__Verrucomicrobiae;o__Verrucomicrobiales;f__Verrucomicrobiaceae;g__Luteolibacter | | 0 | 0 | 0 | 1.47E-05 | 0.00011956 | 5.29E-05 | 0 |
| k__Bacteria;p__Verrucomicrobia;c__Verrucomicrobiae;o__Verrucomicrobiales;f__Verrucomicrobiaceae;g__MSBL3 | | 0 | 0 | 0 | 0 | 2.99E-05 | 0 | 0 |
| k__Bacteria;p__Verrucomicrobia;c__Verrucomicrobiae;o__Verrucomicrobiales;f__Verrucomicrobiaceae;g__Persicirhabdus | | 9.76E-05 | 0.00018515 | 4.06E-05 | 0.00191464 | 0.0005978 | 0.0010719 | 0.00012237 |
| k__Bacteria;p__Verrucomicrobia;c__Verrucomicrobiae;o__Verrucomicrobiales;f__Verrucomicrobiaceae;g__Prosthecobacter | | 0 | 1.42E-05 | 0 | 0 | 0 | 1.32E-05 | 0 |
| k__Bacteria;p__Verrucomicrobia;c__Verrucomicrobiae;o__Verrucomicrobiales;f__Verrucomicrobiaceae;g__Rubritalea | | 0 | 2.85E-05 | 4.06E-05 | 0.00076585 | 0.00263032 | 0.00279222 | 0.00052007 |
| k__Bacteria;p__Verrucomicrobia;c__Verrucomicrobiae;o__Verrucomicrobiales;f__Verrucomicrobiaceae;g__Verrucomicrobium | | 0.00040425 | 0.0005697 | 1.35E-05 | 8.84E-05 | 0.00017934 | 1.32E-05 | 9.18E-05 |
| k__Bacteria;p__Verrucomicrobia;c__[Spartobacteria];o__[Chthoniobacterales];f__[Chthoniobacteraceae];g__ | | 0 | 0 | 0 | 0 | 0 | 0 | 0 |
| k__Bacteria;p__Verrucomicrobia;c__[Spartobacteria];o__[Chthoniobacterales];f__[Chthoniobacteraceae];g__Candidatus Xiphinematobacter | | 0 | 0 | 0 | 0 | 0 | 1.32E-05 | 0 |
| k__Bacteria;p__Verrucomicrobia;c__[Spartobacteria];o__[Chthoniobacterales];f__[Chthoniobacteraceae];g__DA101 | | 0 | 0 | 0 | 0 | 0 | 0 | 0 |
| k__Bacteria;p__Verrucomicrobia;c__[Spartobacteria];o__[Chthoniobacterales];f__[Chthoniobacteraceae];g__Ellin506 | | 0 | 0 | 0 | 0 | 0 | 1.32E-05 | 0 |
| k__Bacteria;p__WS3;c__PRR-12;o__GN03;f__KSB4;g__ | | 0 | 0 | 0 | 0 | 0 | 2.65E-05 | 0 |
| k__Bacteria;p__WS3;c__PRR-12;o__Sediment-1;f__;g__ | | 0 | 0 | 0 | 0 | 2.99E-05 | 0 | 0 |
| k__Bacteria;p__ZB3;c__BS119;o__;f__;g__ | | 0 | 0 | 8.12E-05 | 0 | 0 | 1.32E-05 | 6.12E-05 |
| k__Bacteria;p__[Thermi];c__Deinococci;o__Deinococcales;f__Deinococcaceae;g__Deinococcus | | 0 | 0 | 0 | 0 | 0 | 2.65E-05 | 0 |
| k__Bacteria;p__[Thermi];c__Deinococci;o__Deinococcales;f__Trueperaceae;g__Truepera | | 0 | 5.70E-05 | 0 | 0 | 0 | 0 | 0 |
| k__Bacteria;p__[Thermi];c__Deinococci;o__Thermales;f__Thermaceae;g__Thermus | | 0 | 0 | 0 | 0 | 0 | 0 | 0 |
