# Supplementary material for: Stable Associations Masked by Temporal Variability in the Marine Copepod Microbiome
Source: PLoS One. 2015 Sep 22;10(9):e0138967. doi: 10.1371/journal.pone.0138967 (PMC4579122; doi:10.1371/journal.pone.0138967)
Supplement: S1 Text — (PDF) [file pone.0138967.s005.pdf]

Supplementary Methods

DNA extraction and Polymerase Chain Reaction

For DNA extraction, the samples were thawed at 65ºC, and 180 μl of ATL buffer from the Qiagen Blood and Tissue kit (Qiagen, Germantown, MD) was added. The samples were then treated with 3 freeze-thaw cycles by placing them into -80ºC for 5-8 min (until frozen) then incubating them at 65ºC for 2-3 min (until thawed). Samples were next agitated in a bead beater (Mini-Beadbeater-8, Biospec, Bartlesville, OK) for 2 min (full speed), then pulse centrifuged. Proteinase K (20 μl) (IBI Scientific, Peosta, IA) was added, and the tubes were incubated at 55ºC for 1 h. Samples were pulse centrifuged, 10 μl RNase A was added, and the tubes were then incubated at 65ºC for 10 min, vortexing periodically. 200 μl of buffer AL from the kit was added to tubes, mixing thoroughly. Solution was then pipetted away from the glass beads into a tube containing 200 μl of molecular grade ethanol. The tubes were vortexed and the contents pipetted to spin columns from the kit. The spin and wash protocols, and final elution to 50 μl AE buffer followed the manufacturer's protocol. The DNA was stored at -20ºC.

The samples were processed for amplification of the microbial 16S rRNA as follows. The reactions included 2.5 μl 10X buffer, 1-1.5 μl of 50-mM $MgCl_2$, 0.125 μl of 16S rRNA gene overhang primers 341F_OH and 785R_OH each (25 μM stock) [1] (S2 Table), 0.625 μl dNTPs (10 mM stock), 0.375 μl BSA (20 mg mL^{-1} stock), 0.1 μl of Platinum Taq (Invitrogen, Life Technologies, Grand Island, NY), and one μl of DNA template. The reactions were adjusted to 25 μl with nuclease free water. Amplification consisted of an initial 95ºC for 5 min, followed by 30 cycles of 95ºC for 40 s, 55ºC for 2

min, and 72ºC for 1 min, and a final 72ºC for 7 min on a ABI 2720 thermocycler. The 16S rRNA overhang primers used in PCR were the Illumina sequencing primers fused with the 16S rRNA gene primers (S2 Table). The PCR amplicons were purified following the Agencourt AMPure XP magnetic bead protocol with a 50-μL elution (Beckman Coulter, Brea, CA). Illumina indexes were then attached to the PCR amplicons using the Nextera XT indexing kit (Illumina, San Diego, CA). The indexing reactions contained 5 μl 10X buffer, 2.5 μl $MgCl_2$ (50 mM stock), 5 μl primers P1 and P2 each, 1.5 μl dNTPs, and 0.2 μl Platinum Taq, and 5 μl of purified product from the 16S rRNA PCR with overhang primers, adjusted to 50 μl final volume. The indexing thermocycling conditions included an initial denaturation at 95ºC for 3 min, followed by 8 cycles at 95ºC for 30 s, 55ºC for 30 s, and 72ºC for 30 s, and a final extension at 72ºC for 5 min. The indexed PCR amplicons were purified via the AMPure protocol using a 25-μl final elution. The samples were quantified by the PicoGreen method using a SpectraMax M2 plate reader (Molecular Devices, Sunnyvale, CA) and pooled at uniform concentrations to a final concentration of approximately 4 nM. The samples were sequenced in two runs, each with a total of 96 samples multiplexed (each including additional samples from our other studies).

For amplification of COI for confirming copepod identification, the reactions contained 5 μl of 10X buffer, 5 μl of 50 mM $MgCl_2$, 1 μl of 10 mM dNTPs, 2.5 μl of reverse and forward primers at 20 μM, 0.2 μl Platinum Taq, and 2 μl of DNA from the starved copepod samples as a template, adjusted to 50 μl final volume with nuclease free water. PCR included an initial 94ºC for 5 min, followed by 35 cycles of 60 s at 94ºC, 60 s

at 52ºC, and 90 s at 72ºC, and final extension at 72ºC for 7 min using a ABI 2720 Thermocycler (Life Technologies).

Treatment of negatives in the amplicon sequencing analysis

Negative controls that had no DNA template were sequenced in parallel with the samples, and the sequence data from these samples were used to determine the influence of contamination on the samples that occurred from PCR or sequencing reagents. Detection of amplified sequences from the controls containing no DNA template is a phenomenon commonly encountered in high sensitivity analyses [2]. Presence of such sequences has been attributed to contamination that can stem from genomic DNA in commercial reagents [3]. The majority of the contaminants fell in Sphingomonadaceae within Alphaproteobacteria (16-33% of sequences) and Burkholderiales (mostly Comamonadaceae) within Betaproteobacteria (44-48% of sequences in the negatives). In a few cases, a very few sequences (forming <1% of all sequences in the negatives) representing environmentally relevant groups that were found at high abundances in some samples (such as SAR11), were also detected in the negatives. It is highly unlikely that such sequences would have originated from PCR reagents, but suggests a very minor cross-sample contamination during sequencing, sample handling, or analysis. Based on these observations, OTUs that formed <1% of the sequences in the negatives were left in the sample data, while OTUs that formed >1% of the sequences in the negatives were removed from the sample data, assuming any sequences representing these OTUs in the samples originated from reagent contamination.

COI cloning, sequencing, and sequence analysis

The COI amplicons were cloned and sequenced using pGEM-t vector system with *E. coli* JM109. Sequencing was done at the Massachusetts General Hospital sequencing facility (Cambridge, MA). BLASTn was conducted for the trimmed sequences to identify them.

References

1. Klindworth A, Pruesse E, Schweer T, Peplies J, Quast C, Horn M, et al. Evaluation of general 16S ribosomal RNA gene PCR primers for classical and next-generation sequencing-based diversity studies. Nucleic Ac Res. 2013; 41:e1.

2. Zehr JP, Crumbliss LL, Church MJ, Omoregie EO, Jenkins BD. Nitrogenase genes in PCR and RT-PCR reagents: implications for studies of diversity of functional genes. Biotechniques. 2003; 35:996-+.

3. Woyke T, Tighe D, Mavromatis K, Clum A, Copeland A, Schackwitz W, et al. One bacterial cell, one complete genome. PLoS One. 2010; 5: e10314.
